# The effect of numerical aperture on quantitative use-wear studies and its implication on reproducibility

**DOI:** 10.1038/s41598-019-42713-w

**Published:** 2019-04-19

**Authors:** Ivan Calandra, Lisa Schunk, Konstantin Bob, Walter Gneisinger, Antonella Pedergnana, Eduardo Paixao, Andreas Hildebrandt, Joao Marreiros

**Affiliations:** 1 TraCEr, Laboratory for Traceology and Controlled Experiments at MONREPOS Archaeological Research Centre and Museum for Human Behavioural Evolution, RGZM, Schloss Monrepos, 56567 Neuwied, Germany; 2 Institute for Prehistoric and Protohistoric Archaeology, Johannes Gutenberg University, Schönborner Hof, Schillerstraße 11, 55116 Mainz, Germany; 3 Scientific Computing and Bioinformatics, Institute of Computer Science, Johannes Gutenberg University, Staudingerweg 9, 55128 Mainz, Germany; 4 ICArEHB, Interdisciplinary Center for Archaeology and Evolution of Human Behaviour, University of Algarve, Campus de Gambelas, 8005-139 Faro, Portugal

**Keywords:** Archaeology, Archaeology

## Abstract

Many archeologists are skeptical about the capabilities of use-wear analysis to infer on the function of archeological tools, mainly because the method is seen as subjective, not standardized and not reproducible. Quantitative methods in particular have been developed and applied to address these issues. However, the importance of equipment, acquisition and analysis settings remains underestimated. One of those settings, the numerical aperture of the objective, has the potential to be one of the major factors leading to reproducibility issues. Here, experimental flint and quartzite tools were imaged using laser-scanning confocal microscopy with two objectives having the same magnification but different numerical apertures. The results demonstrate that 3D surface texture ISO 25178 parameters differ significantly when the same surface is measured with objectives having different numerical apertures. It is, however, unknown whether this property would blur or mask information related to use of the tools. Other acquisition and analyses settings are also discussed. We argue that to move use-wear analysis toward standardization, repeatability and reproducibility, the first step is to report all acquisition and analysis settings. This will allow the reproduction of use-wear studies, as well as tracing the differences between studies to given settings.

## Introduction

Investigating how artifacts were produced and used in the past by humans is one of the key research areas in the study of human behavioral evolution. Although use-wear analysis has the clear potential to significantly contribute, a lot of criticism has been raised against it, mainly due to a lack of standardization during experiments and analyses, compromising in turn its repeatability and reproducibility^1–3^.

In these discussions, the importance of equipment and analysis settings is often overlooked and underestimated. For example, different pieces of equipment, objectives (see Supplementary Material 1 for definitions and details), as well as light and analysis settings have been shown or are expected to yield different results^4,5^. Quantitative use-wear analyses^6–10^ are likely to be more sensitive to such acquisition and analysis settings. As more emphasis has been put on quantitative analyses in recent years, it is now important to define which settings play a role and should therefore be standardized, if possible.

Furthermore, it is well known that a surface –be it from an engineered tool, an animal tooth or an archeological artifact– appears differently when observing it at different scales, or magnifications^11^. The application of both high and low power approaches to use-wear analyses^12–15^ demonstrates that traceologists recognize the importance of scale. Yet, the magnification and resolution of acquisition and analysis (see Supplementary Material 1 for definitions and details) are rarely unambiguously reported in archeological studies. We argue that this is, at least partly, due to the recent developments in digital microscopy.

In the context of an experiment, *repeatability* measures the variation in measurements taken by a single instrument or person under the same conditions, while *reproducibility* measures whether a study or experiment can be reproduced in its entirety. *Preproducibility* is a neologism that Philip B. Stark defined as follows (p. 613): “An experiment or analysis is preproducible if it has been described in adequate detail for others to undertake it. Preproducibility is a prerequisite for reproducibility”^16^.

In the present study, we list and discuss the relevant hardware and software settings that should be reported if the research is to be preproducible. This list is by no means exhaustive, but it represents a solid starting point. As an example of such settings, we tested whether the numerical aperture (NA) of the objective can and does influence the results of archeological quantitative use-wear analyses.

From a theoretical point of view, the NA should have an impact on the way an image is acquired, because it dictates the optical lateral and axial resolutions, as well as the steepest slopes that can be measured (see e.g. refs^17,18^ and Supplementary Material 1). The NA has already been shown to have an effect on the image acquired^19,20^, but, to our knowledge, this effect has not been measured on surface topographies acquired with confocal microscopy. Furthermore, it is currently unknown how variations in NA would affect the results of quantitative use-wear analysis. Therefore, we acquired quantitative surface texture data of experimental tools at high magnification with two objectives having different numerical apertures. This represents one of the first steps toward comparability, repeatability and reproducibility in use-wear analyses.

Hereafter, following Leach^21^, the term *surface topography* will be used to describe the overall surface structure, while *surface form* is defined as the shape of the object, and *surface texture* is what remains when the form is removed from the topography. These definitions differ from Evans et al^6^., where texture describes the *roughness* and topography the *waviness* (both included in Leach’s^21^ texture), the distinction between roughness and waviness being based on wavelength filters (see below).

The 3D images referred to below are representations of the surface topography, form and texture of the samples. These 3D images, or 3D surface data, can be processed so that the surface topography and/or texture are measured quantitatively. Many parameters describe specific attributes of the topography and/or texture.

## Results

**Figure 1:**
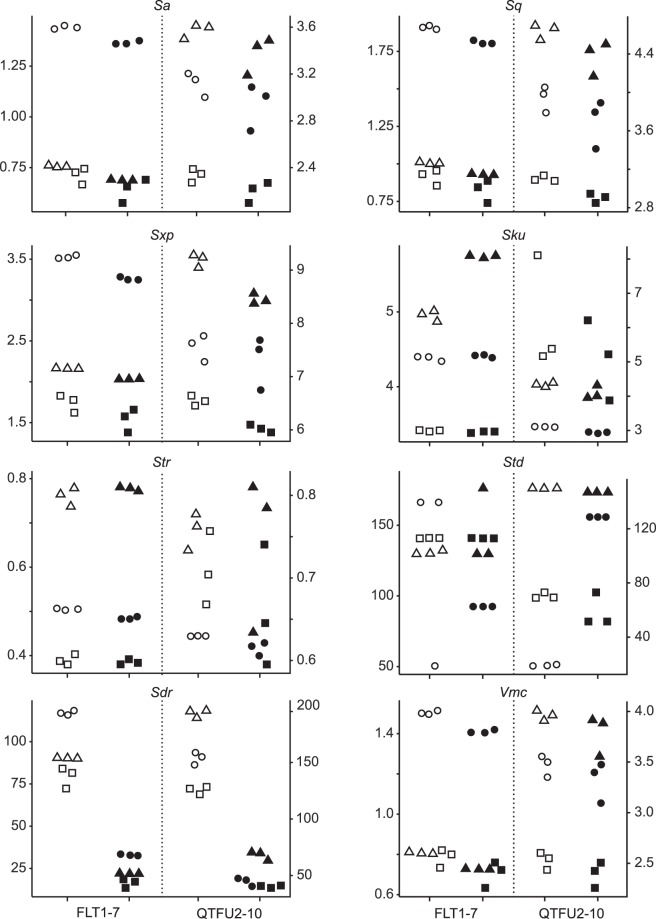
Scatter plots of the selected ISO 25178 parameters: *Sa*, *Sq*, *Sxp*, *Sku*, *Str*, *Std*, *Sdr* and *Vmc*. For each plot, the left y-axis relates to FLT1-7 (flint) and the right y-axis corresponds to QTFU2-10 (quartzite). Symbols differentiate the three locations on each sample (○ = location 1, Δ = location 2 and □ = location 3), empty symbols represent data acquired with the 50×/0.75 objective, and filled symbols correspond to data from the 50×/0.95 objective. See Supplementary Table S1 for details on parameters.

**Figure 2:**
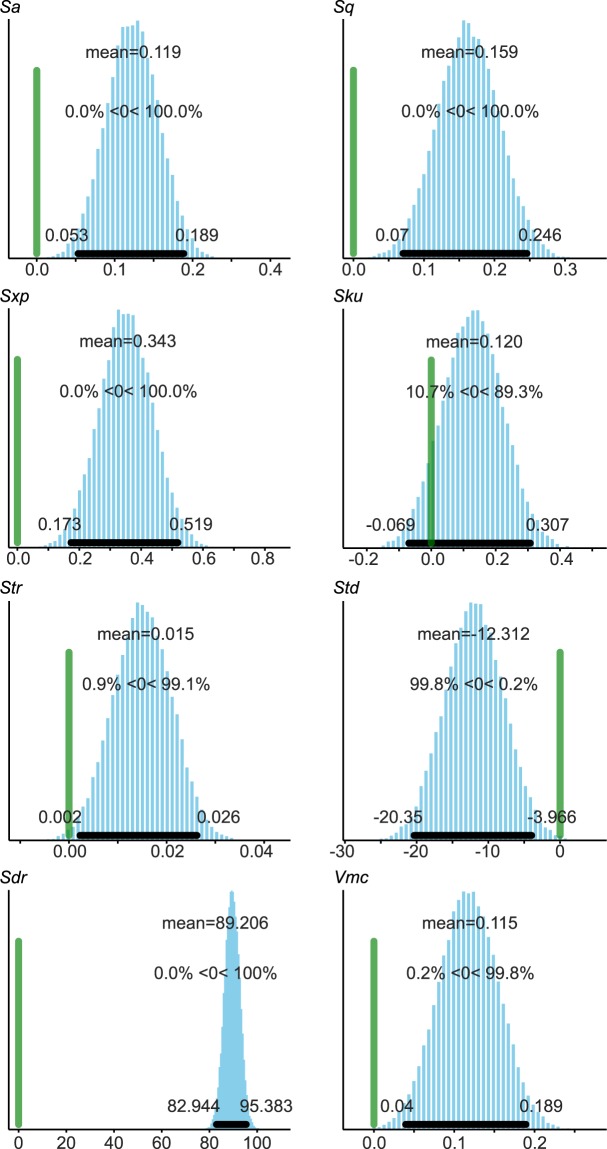
Contrast plots between the two objectives of the selected ISO 25178 parameters: *Sa*, *Sq*, *Sxp*, *Sku*, *Str*, *Std*, *Sdr* and *Vmc*. The green vertical line marks the 0 effect strength, while the black horizontal line and the values given on each side represent the 95% high probability density interval.

Twenty nine ISO 25178-2 parameters were calculated on each surface of the flint and quartzite samples (Supplementary Material 2 and Table S1). Three of them (*Spq*, *Svq* and *Smq*) could not be calculated on most surfaces (Supplementary Table S2) so they were not included in the inferential statistics. Out of the 26 analyzed (Supplementary Materials 3, 4 and Table S3), eight parameters, spanning the different categories of field parameters, were selected for figures (Figs 1 and 2). The *Sa* and *Sq* parameters are different measures of surface roughness^22^. *Sxp* is the height difference between the average height of the surface (*p* = 50% material ratio) and the highest peak, excluding the 2.5% highest points (*q* = 97.5% material ratio). *Sku* is the kurtosis of the height distribution of the surface texture. *Str* is a measure of isotropy; it varies between 0 (anisotropic surface) and 1 (isotropic surface). *Std* calculates the main direction of the surface, but is obviously only relevant for anisotropic surfaces (*Str* < 0.5). *Vmc* is the volume of material (i.e. below the surface), excluding the 10% lowest (*p* = 10%) and 20% highest (*q* = 80%) points. *Sdr* is a measure of surface complexity.

There are significant differences for 25 parameters (all but *Sku*) between the height maps acquired with objectives having different NA values (Figs 1 and 2, Supplementary Materials 3, 4 and Table S3). The standard deviations are more often larger with the 50×/0.75 than with the 50×/0.95 objective, but this depends a lot on the parameter considered (Supplementary Table S3).

**Figure 3:**
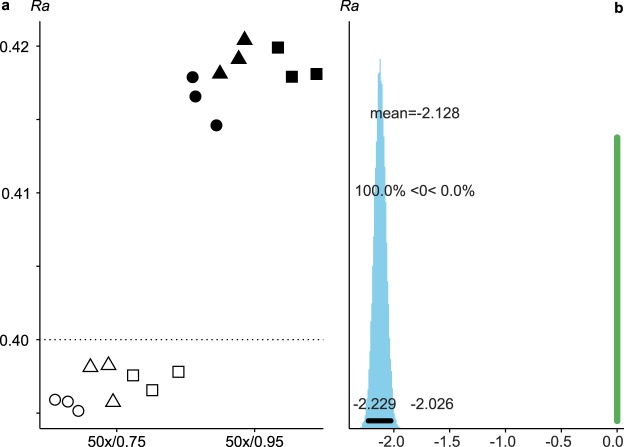
Scatter (**a**) and contrast (**b**) plots of ISO 4287 *Ra* calculated on the surfaces from each objective on the roughness standard. The dotted line in (**a**) highlights the nominal *Ra* value of the roughness standard (0.40 µm). Note that values in (**b**) are given in units of 0.01 µm. See Fig. 1 for details on symbols.

Both objectives produced results within the tolerance range of the nominal *Ra* value of the roughness standard (*Ra* = 0.40 ± 0.05 µm; Fig. 3a, Supplementary Material 3 and Tables S2 and S3). However, it should be stressed that the values from each objective are significantly different (Fig. 3b, Supplementary Material 4), the 50×/0.75 objective producing values closer to the nominal value (Supplementary Table S3).

## Discussion

Both objectives used here yield data within the certified tolerance of the roughness standard, although they are significantly different from each other (Fig. 3). The values calculated on surfaces acquired with the 50×/0.75 objective are closer to the nominal *Ra* value than those acquired with the 50×/0.95 objective. This is surprising, as the higher numerical aperture (NA) objective should theoretically produce the most accurate results. The real *Ra* value of the roughness standard is unknown, though; it could be that the real *Ra* value is closer to 0.42 µm than to the nominal *Ra* value 0.40 µm.

Nevertheless, the results demonstrate that the NA of an objective influences the way the surface topography is acquired on both quartzite and flint (Figs 1 and 2), two of the most common raw materials in the archeological record. This, in turn, implies that quantitative use-wear analyses have the potential to produce different results depending on the objective used on most archeological samples. Previous research in microscopy^19,20^, and the role of NA on resolution in general (see Supplementary Material 1), have shown that this influence of the objective’s NA was to be expected. However, this effect had not been measured before in archeological use-wear studies. Unfortunately, this property is not always reported in quantitative use-wear research.

Objective manufacturers offer a wide range of objectives, with different combinations of magnification, numerical aperture and working distance, to cover numerous applications. To our knowledge, however, the 50×/0.95 objective is the only 50× objective produced by all manufacturers. This is likely because 0.95 is the highest numerical aperture for non-immersion (i.e. air) objectives. Therefore, this objective appears to be the best candidate for standardization in use-wear studies. Nevertheless, having the highest possible numerical aperture also means that this objective has the smallest working distance. This could be problematic for samples made of coarse-grained materials, such as quartzite. Indeed, the sample used here (QTFU2-10) proved challenging to image with this objective (working distance = 0.22 mm): the sample had to be very precisely oriented and only the highest locations could be imaged without the objective touching the sample. Still, 0.22 mm is a minute distance that is challenging even to the experienced user.

These results are highly relevant in the growing field of quantitative use-wear analyses. Indeed, using quantitative methods is often seen as a way to improve standardization and, in turn, repeatability and reproducibility^1^. It was demonstrated here that this is only true if the same acquisition parameters are used. The objective used (magnification and numerical aperture) is a critical component of a microscope, but it is not the only one. Different types of imaging equipment are known to produce results that are not quantitatively comparable^5^. Furthermore, resolution, which is based on the objective’s NA, on the light source and on the size of camera/detector (see Supplementary Material 1), surely plays a role in the way wear features are measured. As this was beyond the scope of this paper, it was not tested. Other acquisition settings, concerning both hardware and software, might also have an impact on the measurement of surface textures.

The processing workflow and filter cut-off values are likely to have a major influence on the topography of the surface that will be quantified, although the magnitude has not been measured yet on archeological samples (but see refs^4,23^ for a discussion of analysis protocols in dental microwear texture analysis). This post-processing can also be used to compare surface data produced by different types of equipment^4,23^. Therefore, analysis settings should also be reported as exhaustively as possible. As surfaces can be processed many times with different settings by different researchers, we urge all archeologists to provide access to the unprocessed data, for example by using repositories. The present raw data, including the acquired surfaces and the whole processing workflow, are available as *.mnt (MountainsMap) files on Zenodo (10.5281/zenodo.1479117).

Table 1 and Supplementary Material 2 list all hardware and software settings related to both acquisition and analysis used here. Nevertheless, there might be settings that are not accessible in this system and/or software packages but that are still relevant to data acquisition and analysis. Furthermore, other systems might have different settings and it is likely that some have different names.

**Table 1:** Acquisition settings.

Setting		FLT1-7	QTFU2-10	Roughness standard
Microscope	Manufacturer	Carl Zeiss Microscopy GmbH		
	Model	Axio Imager.Z2 Vario + LSM 800 MAT		
Location	Laboratory	TraCEr laboratory, MONREPOS, Germany		
	Floor	−1 (basement)		
	Anti-vibration table	Passive		
Acquisition	Software	ZEN blue 2.3 with Shuttle&Find module		
	Mode	LSM (laser scanning confocal microscopy)		
Objective	Manufacturer	Carl Zeiss Microscopy GmbH		
	Objective 1	EC Epiplan 50×/NA = 0.75/WD = 1 mm		
	Objective 2	C Epiplan-Apochromat 50×/NA = 0.95/WD = 0.22 mm		
Illumination	Source	Laser		
	Wavelength	405 nm		
	Intensity	4%		
Settings	Scanning direction	Both ways (no correction, line step = 1)		
	Scanning speed	8 (max)		
	Bit depth	16 bits		
	Master Gain	260 V		195 V
	Pinhole diameter objective 1	73 µm (=1 AU lateral optical resolution)		
	Pinhole diameter objective 2	54 µm (=1 AU lateral optical resolution)		
Size and resolution	Zoom	0.5×		
	Field of view	255.56 × 255.56 µm		945.62 × 255.56 μm (4 × 1 tiles)
	Frame size	3000 × 3000 pixels		7578 × 2048 pixels
	X/Y pixel size	0.0852 µm		0.125 µm
	Step size	0.25 µm		
	Data quality	No noise cut (0–65335 levels, post-processing)		
Measurement conditions	Duration	≈5–10 min	≈15–20 min	≈5–6 min
	Vertical (z) measuring range	20–37 µm	60–82 µm	10 µm
	Temperature	25.4 to 26.2 ± 0.5 °C	24.5 to 26.1 ± 0.5 °C	25.1 to 26.4 ± 0.5 °C
	Relative humidity	46.2 to 55.2 ± 3%rH	51.9 to 54.9 ± 3%rH	48.5 to 53.6 ± 3%rH

AU = Airy Unit, NA = numerical aperture, WD = working distance.

While the influence of at least some of these settings is critical when quantifying use-wear, their influence on qualitative use-wear has not been considered. However, a conservative approach would be to be as cautious about acquisition and analysis settings in qualitative studies as in quantitative ones.

It is currently still unknown how to best define these settings for use-wear analyses on experimental and archeological samples (lithics made of different raw materials, bone, antler, shells…), so it is currently impossible to define standards. In the meantime, we therefore recommend that every use-wear study reports all the settings used so that, at least, the studies are preproducible and the source of variation between studies can be traced to one or several acquisition settings.

In this study, we tested whether using objectives with different numerical apertures affects the results of quantitative 3D surface texture analysis. It appears that the surfaces of experimental flint and quartzite tools, as well as those of a roughness standard, are significantly different when acquired with different objectives and analyzed quantitatively. The numerical aperture is only one of the many acquisition and analysis settings that could influence the results of use-wear analyses.

The present results have implications on how to move use-wear analysis toward a reproducible science. This goal can only be achieved if all relevant acquisition and analysis settings are standardized. As it is still unknown which settings are relevant and which values should be used for these settings, this ultimate goal remains out of reach. Nevertheless, a first step would be to report all hardware and software settings that can vary between studies and that can be adjusted by the users of the piece(s) of equipment. Listing all these parameters can be done very quickly and easily; for example, Table 1 was prepared in a few minutes and Supplementary Material 2 was created automatically in batch. The potential benefit of doing this is significant and therefore largely exceeds the minimal costs. Eventually, standardization will help us in exchanging data as well as comparing, reproducing and replicating use-wear studies^1^, lending more weight to our archeological interpretations.

## Methods

### Samples

**Figure 4:**
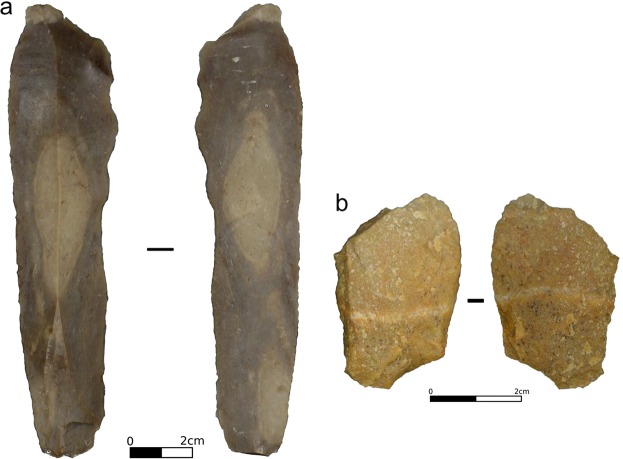
Photos of the two experimental tools used in this study, FLT1-7 (**a**) and QTFU2–10 (**b**). Photos were taken with a Nikon DSLR camera D610 with a Nikon AF-S VR Micro-Nikkor 105 mm f/2.8G IF-ED lens.

We selected two experimental tools displaying use-wear. The first tool (FLT1–7; Fig. 4a) is a blade knapped from flint from the French Pyrenees (Narbonne-Sigean Basin). It was used in mechanical bi-directional linear (cutting-like) action on dry wood (*Pinus* sp.) boards. It performed 250 strokes of 2 × 30 cm at 0.5 m.s^−1^ with a 4.5 kg load applied onto the tool (Pereira *et al*. in prep.). The second tool (QTFU2–10; Fig. 4b) is an unretouched metaquartzite flake manually used to cut a Giant cane’s stem (*Arundo donax*) for 2 × 15 min (see ref.^24^ for details).

The samples have been cleaned thoroughly previously (see refs^24,25^). The measured areas (around the edge) were cleaned again with 2-propanol 70%v/v and lens cleaning tissues just before acquisition.

100–200 µm ceramic beads were adhered onto the samples with epoxy resin to provide reference points for the coordinate system (see ref.^25^ for details). This allows us to find the same spot again for future analyses.

Even though the objective with the highest NA should yield the results closest to reality, the real, expected results for these rock samples are unknown. Therefore, a roughness standard with nominal *Ra* = 0.40 ± 0.05 μm was measured with each of the two objectives. The measured *Ra* values were then compared to the nominal value.

### Data acquisition

**Figure 5:**
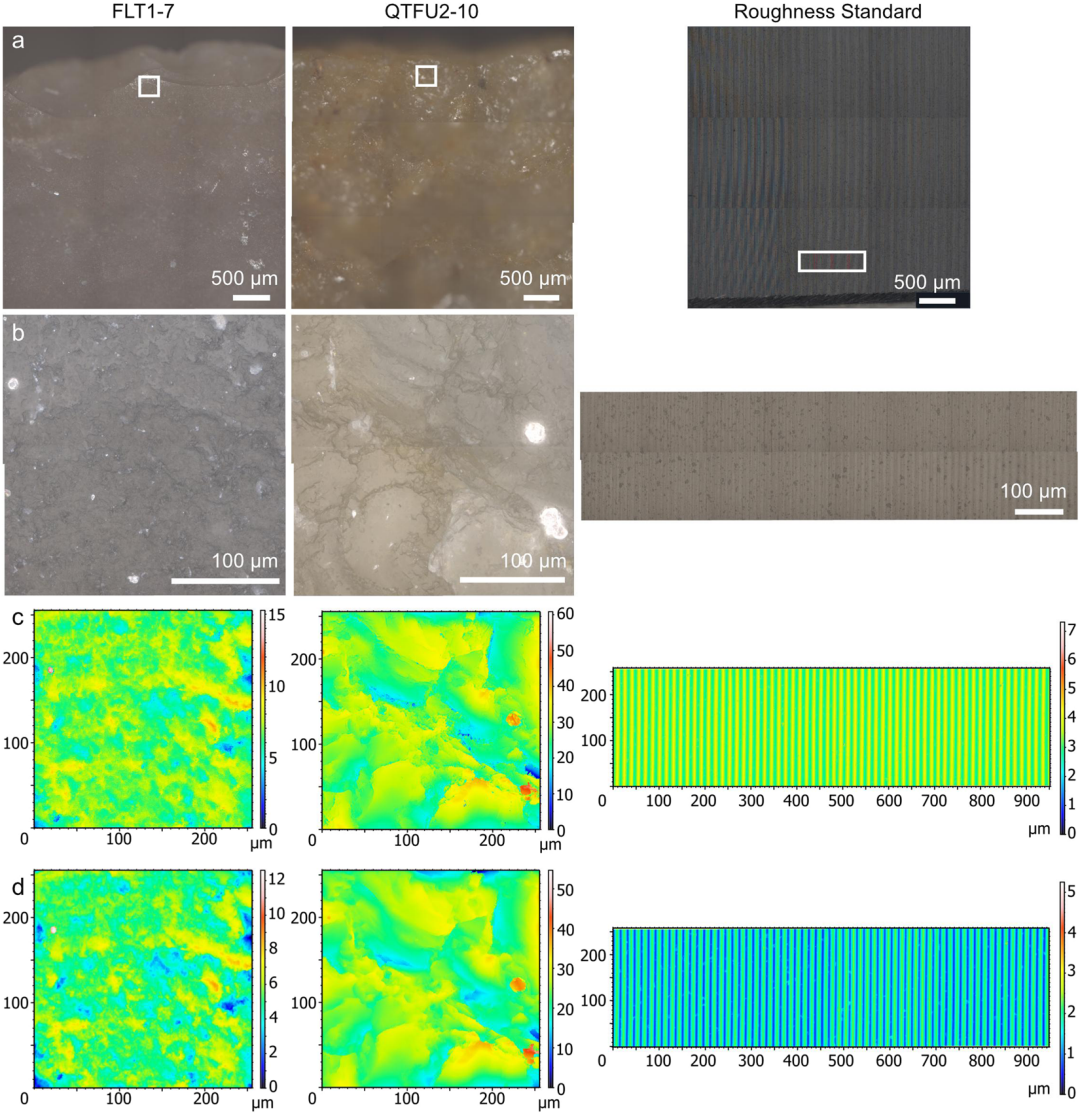
Locations 2 and replicas 1 on all three samples: (left) FLT1-7, (middle) QTFU2–10 and (right) roughness standard 0.4 µm. **(a)** Stitched 3 × 3 overview image acquired with the C Epiplan-Apochromat 5×/0.20 objective. (**b**) Stitched 2 × 2 (FLT1–7 and QTFU2–10) or 8 × 2 (roughness standard) wide field image acquired with the 50×/0.95 objective. **(c-d)** S-L surfaces (FLT1–7 and QTFU2–10) or leveled surfaces (roughness standard, stitched 4 × 1) acquired with the 50×/0.75 and 50×/0.95 objectives, respectively.

We acquired 3D surface data on the samples (Fig. 5c,d) with an upright light microscope Axio Imager.Z2 Vario coupled to laser-scanning confocal microscope (LSCM) LSM 800 MAT, manufactured by Carl Zeiss Microscopy GmbH. The system was turned on at least one hour before starting acquisition, so that all components were warmed up to limit thermic drift. The LSCM was equipped with an EC Epiplan 50×/0.75 (Fig. 5c) objective and a C Epiplan-Apochromat 50×/0.95 objective (Fig. 5d) on a motorized revolver (Carl Zeiss Microscopy GmbH). The numerical apertures of the objectives are 0.75 and 0.95, respectively, as written after the slash in the description of the objectives above.

All relevant information and acquisition settings are listed in Table 1. On the flint and quartzite samples, the field of view (FOV) was 255.6 × 255.6 µm. The pixel size was calculated following as closely as possible the ISO 4287/4288 norms^26,27^: L = FOV/2 = 127.8 µm, S_1_ = L/300 = 0.426 µm, and pixel size = S_1_/5 = 0.0852 µm. The frame size was then defined as the field of view divided by the pixel size, i.e. 3000 × 3000 pixels. The field of view on the roughness standard was set to 945.62 × 255.56 μm, with a 4 × 1 stitched image, representing a frame size of 7578 × 2048 px. In doing so, evaluation length (4.56 mm ≥ 4 mm), sampling length (0.927 ≥ 0.8 mm), and point spacing (0.125 ≤ 0.5 μm) are according to ISO 4287/4288, so that the measured values can be compared to the nominal value. The pinhole diameter was adjusted so that it corresponds to 1 Airy Unit for each objective: 54 µm for the 50×/0.95 objective and 73 µm for the 50×/0.75 objective. This means that the optical lateral resolution of the objectives was kept constant throughout the experiment. The Shannon-Nyquist criterion (pixel size less than half the optical lateral resolution; see Supplementary Material 1) is met on all samples.

The samples were positioned with the measured area as horizontal as possible to minimize the vertical (z-axis) measuring range. Temperature and humidity were measured constantly. Three locations were measured on each sample. Each location was scanned three times (i.e. technical replicas) with each objective. In order to compensate for unknown confounding factors that could influence the results, we randomized the acquisition as follows:

1. Sample 1/location 1/replica 1 with objective 1, then objective 2
2. Repeat step #1 on sample 1/location 1/replicas 2-3.
3. Repeat steps #1-2 on location 1 of samples 2-3.
4. Repeat steps #1-3 for locations 2-3 of samples 1-3.

To gain a better representation of the imaged areas, wide field overview and extended depth of focus (EDF) images were also acquired at each location. The overview images (Fig. 5a) were acquired with the following settings: C Epiplan-Apochromat 5×/0.20 objective and 3 × 3 tile region. The EDF images (Fig. 5b) were acquired with the following settings: the 50×/0.95 objective described above, step size = 1 µm, 2 × 2 tile regions for flint and quartzite, and 8 × 2 tile regions for the roughness standard in order to cover the same area.

### Data processing

The resulting 3D surface data were processed in batch with templates in ConfoMap v7.4.8633 (a derivative of MountainsMap Imaging Topography developed by Digital Surf, Besançon, France).

The template for the roughness standard (Supplementary Material 2) followed the ISO 4287/4288 norms, in order to compute values that are comparable to the nominal value: (1) level by least squares plane subtraction (Fig. 5c,d), (2) extract a 4.56 mm-long profile, (3) apply a Gaussian microroughness low-pass filter (λ_s_ = 2.5 μm) to filter out the noise and keep the primary profile, (4) apply a Gaussian roughness high-pass filter (λ_c_ = 0.8 mm, end effects not managed) to filter out the waviness and keep the roughness profile, and (5) compute ISO 4287 *Ra* (Supplementary Table S1).

The template for flint and quartzite samples performs the following procedure on each 3D surface (Supplementary Material 2): (1) apply a Gaussian low-pass S-filter (S_1_ nesting index = 0.425 µm, end effects managed) to remove noise and keep the primary surface, (2) apply an F operator (polynomial of degree 3) to remove the form and keep the SF surface, (3) apply a Gaussian high-pass L-filter (L nesting index = 127 µm, end effects managed) to filter out the waviness and keep the SL surface (Fig. 5c,d), and (4) compute 29 ISO 25178-2 parameters^28^ (Supplementary Table S1). This template follows Digital Surf’s Metrology Guide (accessible at https://guide.digitalsurf.com/en/guide.html) as closely as possible, but it should not be expected that lithic tool surfaces require the exact same processing as dictated by the ISO norms defined for industrial applications. We therefore adapted the cut-off values for the filters based on field of view, frame size and pixel size, as detailed above. Much more work is needed to define the most appropriate way to analyze surfaces of archeological tools but this task is beyond the scope of the present study. The processing workflow was performed consistently to enable the comparison, which was the goal. It is not intended as a general recommendation on how to measure surfaces of experimental or archeological samples.

### Statistical procedure

All descriptive analyses (summary statistics and scatter plots) were performed in the open-source software R v. 3.5.1 (ref.^29^) through RStudio (v. 1.1.456; RStudio Inc., Boston, USA) for Microsoft Windows 10. The following packages were used: doBy v. 4.6-1 (ref.^30^), ggplot2 v. 3.0.0 (ref.^31^), openxlsx v. 4.1.0 (ref.^32^), R.utils v. 2.7.0 (ref.^33^). Reports of the analyses in HTML format, created with knitr v. 1.20 (refs^34–36^) and rmarkdown v. 1.10 (ref.^37^), as well as raw data, scripts and RStudio project, are available as Supplementary Material 3.

To evaluate whether the numerical aperture significantly changes the measured value of the surface parameters, a Bayesian Multi-factor ANOVA was applied. This method computes the amount of variances that can be attributed to a single factor or a combination of two factors using Bayesian inference.

There are several advantages to this approach compared to the traditional null hypothesis testing procedure^38^. First, this method does not rely on assumptions other than the ones stated below and is therefore more transparent. Second, by using the full posterior distribution for the significance testing, the certainty of the results can also be assessed. Finally, regarding the practical component of the analysis, the availability of steadily increasing computational power and user friendly software libraries makes the greater complexity of the computation not a serious drawback compared to the gain in insight.

The whole analysis was performed in Python with the package PyMC3 (ref.^39^).

The change in numerical aperture is considered here as the first factor, *x*_1_. The combination of the two other settings, the type of raw material (quartzite or flint) and the location on the sample, is considered as the second factor, *x*_2_. For every single measured surface parameter, the measurement outcome *y* is related to the factors by a linear model:

$$y={\beta }_{0}+{\beta }_{1}\cdot {x}_{1}+{\beta }_{2}\cdot {x}_{2}+{x}_{1}\cdot M\cdot {x}_{2}$$

The terms of the equation can be understood as follows: *β*_0_ is a real number that indicates the overall order of magnitude of the measured values. *β*_1_ is a vector of length 2 that contains the effect strengths of choosing the numerical aperture, while *x*_1_ is a vector that indicates the level of factor 1, i.e. *x*_1_ is [1, 0] when choosing the first level of factor 1 and [0,1] in the other case. The same applies to *β*_2_ and *x*_2_, but here with 6 different levels (2 levels for raw material × 3 levels for location). M is a matrix where the entry *M*_*i,j*_ indicates the effect strength of the particular combination of the two factors.

In order to check for a significant effect, the unknown parameters *β*_0_, *β*_1_, *β*_2_ and *M* must be inferred from the data and the prior knowledge on the measurement process. The detailed model is chosen as

$${\beta }_{0} \sim N(m,s)$$

$${\beta }_{1} \sim N({0},{\sigma }_{1})$$

$${\beta }_{2} \sim N({0},{\sigma }_{2})$$

$$M \sim N({0},{\sigma }_{M})$$

$${\mu }={\beta }_{0}+{\beta }_{1}\cdot {x}_{1}+{\beta }_{2}\cdot {x}_{2}+{x}_{1}\cdot M\cdot {x}_{2}$$

$$\varepsilon  \sim U({0},Erro{r}_{Max})$$

for the priors, where ‘~’ means ‘is distributed as’ and *N(a, b)* denotes a normal distribution with mean *a* and standard deviation *b* and *U(a, b)* a uniform distribution between *a* and *b*.

The hyperparameter are chosen as follows: *m* denotes the estimated mean of the measured data and *s* the estimated standard deviation. *σ*_1_ and *σ*_2_ are calculated as the maximum observed effect strength when varying factor 1 or 2, respectively. *σ*_*M*_ is computed as 5% of the combined effect strength $\sqrt{{\sigma }_{1}^{2}+{\sigma }_{2}^{2}}$ as, from a priori knowledge, there is no interaction between the numerical aperture and the location and sample type. *Error*_*Max*_, which is a strict upper bound on the measurement error for stabilization of the computation, is chosen as 20% of the minimum of *σ*_*1*_ and *σ*_*2*_, although the measurement process itself is far more precise. Lastly the likelihood is modeled as *y* ~ *N(µ,ε)*.

The posterior distribution is now accessed by sampling using a special variant of Markov Chain Monte Carlo, the Hamiltonian Monte Carlo algorithm^40^ in the implementation by Salvatier *et al*.^39^. When performing the sampling, the results have to be checked for consistency based on the trace plots and on the energy plots of Hamiltonian Monte Carlo (see Supplementary Material 4 for details).

After having computed the samples from the posterior, the so-called contrast, i.e. the distribution of the differences between *β*_*1,0*_ and *β*_*1,1*_, can be analyzed. To decide whether there is a significant effect in changing the numerical aperture, the 95% high probability density interval of 2.5% to 97.5% cumulated probability of the contrast is considered. If zero effect strength is not within that interval, the effect is considered significant.

## Supplementary information

Supplementary Material 1

Supplementary Material 2

Supplementary Material 3

Supplementary Material 4

Supplementary Table S1

Supplementary Table S2

Supplementary Table S3

## Data Availability

All data generated and/or analyzed during the current study are included in this published article and its Supplementary Information files, or are available on Zenodo (see Supplementary Materials 2, 3 and 4).
