# Supplementary material for: The effect of numerical aperture on quantitative use-wear studies and its implication on reproducibility
Source: Sci Rep. 2019 Apr 19;9:6313. doi: 10.1038/s41598-019-42713-w (PMC6474883; doi:10.1038/s41598-019-42713-w)
Supplement: Supplementary file 1 — Supplementary Material 1 [file 41598_2019_42713_MOESM1_ESM.pdf]

The effect of numerical aperture on quantitative use-wear studies and its implication on reproducibility

Ivan Calandra, Lisa Schunk, Konstantin Bob, Walter Gneisinger, Antonella Pedergnana, Eduardo Paixao, Andreas Hildebrandt, Joao Marreiros

Scientific Reports

Supplementary Material 1. Definitions and details on the properties of objectives, magnification and resolution.

Properties of objectives

The *objective magnification* is the "nominal lateral imaging scale of an objective" (Fair Data Sheet [FDS] §2.2.4, ref. [1]) and will define, together with the optical/digital zoom factor, the measuring area (see also ref. [2]).

Other important properties of an objective are its *numerical aperture* (NA; FDS §2.2.5) and *working distance* (WD; FDS §2.2.2). The numerical aperture defines the maximum angle (α) that can be received by the objective: $NA = n\,.\ \sin\alpha$. Because the refractive index of air can be assumed to be equal to 1, the maximum theoretical value of the NA for a non-immersion objective is 1 too, although the maximum practical value is 0.95.

The working distance is a crucial property too, especially when observing non-flat, non-horizontal samples, common in archeology. Unfortunately, the laws of optics dictate that NA and WD are roughly inversely correlated. Therefore, there is a trade-off between NA and WD. The most appropriate combination depends on the samples observed.

It is worth noting that the NA and WD of an objective can vary, even for a given magnification. For example, Carl Zeiss Microscopy GmbH produces 50× objectives with $0.55 \le NA \le 0.95$ and $0.22 \le WD \le 9.1$ mm. Because all these properties can be combined in many ways, it is important to report all of them.

Magnification

Many archeologists observe their samples through the oculars of a microscope. In this case, the *magnification of observation* is the magnification of the objective multiplied by the magnification of the oculars, multiplied by the optical zoom factor (if applicable), e.g. a 100× objective combined with 10× oculars and 2× zoom factor result in a magnification of observation equal to 2000×.

However, in order to share observations with the archeological community, oral presentations, posters and publications include digital photographs, either made with a mounted DSLR camera or with an integrated CCD/CMOS camera. Calculating the magnification of a digital image, what we refer to as *digital magnification*, is less straightforward. It still depends on the objective magnification and on the optical zoom factor (if applicable), but the magnification of the camera adaptor, the digital zoom (if applicable), the screen diagonal and the camera sensor (chip) diagonal also play a role:

$$Digital\ mag. = \frac{objective\ mag. \times optical\ zoom \times camera\ adaptor \times screen\ diagonal \times digital\ zoom}{camera\ sensor\ diagonal}$$

This implies that the digital magnification is necessarily different from the magnification of observation when looking through the oculars. Therefore, the magnifications of observation and reporting differ. In other words, the researcher doing the work and the community looking at the reported digital images might observe the samples at different scales, and therefore observe different aspects of the samples.

On digital microscopes, that is, microscopes without oculars on which the user observes directly on a computer screen, the magnification of observation is equal to the on-screen magnification and therefore to the digital magnification of the image produced. Hence, one of the advantages of such microscopes is that the observation of the sample is performed at the scale at which the digital image is acquired. Unfortunately, a lack of standards for

specifications means that it is difficult to compare the magnifications of different digital microscopes without detailed knowledge of all the parameters listed above.

In summary, the term ‘magnification’ can be confusing and precise definitions should thus be used. However, reporting the magnification correctly is valid only as long as the image is not enlarged or reduced, e.g. during publication, printing or projection. Therefore, adding a scale bar to every digital image is necessary too.

Resolution

Resolution also plays a role in defining the scale of observation because it defines the size of the smallest observable features. Firstly, it is important to explain what the resolution is. In 3D imaging, resolution is split into horizontal or lateral (XY), and vertical or axial (Z) resolutions.

Lateral resolution can be defined in two ways: *optical lateral resolution* and *measuring point spacing*. The former is the “minimum theoretical distance between two adjacent, barely distinguishable features of an object” (FDS §2.2.8). The optical lateral resolution is based on the point-spread function and is calculated as follows [2,3]:

$$\delta_L = \frac{K \,.\, \lambda}{NA}$$

with K = Rayleigh resolution (K = 0.51 at 1 Airy Unit pinhole diameter, as used here), λ = wavelength of the light source and NA = numerical aperture of the objective (see below).

The Airy Unit (AU) is used to standardize resolution relative to the light source and numerical aperture of the objective [3]. It is defined as follows:

$$1\,AU = \frac{1.22\,\lambda}{NA}$$

The optical lateral resolution therefore only depends on the light source and the objective used. Using an objective with a larger NA or a light source with a shorter wavelength will both decrease the optical lateral resolution (what is usually referred to as “higher resolution”). When observing through the oculars, only this optical definition of resolution is relevant.

However, the resolution of a digital image can complementarily be defined as the measuring point spacing, also known as pixel size. It is defined as the “sampling interval of measuring points in the measuring volume, both in X and in Y direction” (FDS §2.2.7), and is calculated by dividing the measuring area (or field of view; FDS §2.2.1) by the maximum number of measuring points in a single measurement (or number of pixels, or frame size; FDS §2.1.2). Thus, it depends on the objective, optical zoom and number of pixels on the camera sensor. Higher optical magnification (i.e. smaller measuring area) and more pixels on the camera sensors will both decrease the measuring point spacing and, in turn, increase the digital resolution.

A digital zoom will proportionately decrease the measuring area and the frame size, and will therefore have no effect on the digital resolution. However, most of the time, the image on the screen is not scaled down with the measuring area; rather, the size of the image on the screen stays constant. This means that the total magnification increases, which is the goal of the digital zoom. Pixels might become so enlarged that they are apparent, giving the impression that resolution decreases, even though it is constant.

The distinction between optical lateral resolution and measuring point spacing is crucial because they relate to different parts of the system: optical or digital components. As such, they are complementary and both should be reported when applicable. The Shannon-Nyquist sampling theorem states that the measuring point spacing should be lower than or equal to half of the optical lateral resolution [3–5]. In other words, there should be at least 2 pixels to image in a satisfying manner the transition between (optical) features.

The *axial resolution* measures the vertical distance between barely distinguishable features. For light microscopy, the axial resolution is equal to the depth of field [3]:

$$d_{axial(Light)} = \frac{\lambda \, . \, n}{NA^2}$$

with *n* being the refractive index of the surrounding medium.

For confocal microscopy, the axial resolution at 1 AU pinhole diameter is calculated as follows, as used here [3,6]:

$$d_{axial(Confocal)} = \frac{0.88 \, . \, \lambda}{n - \sqrt{n^2 - NA^2}}$$

The *optical slice thickness* in confocal microscopy at 1 AU pinhole diameter, corresponding to the *depth of field* in light microscopy, is given by [3,6]:

$$Optical\ slice\ thickness = \sqrt{\left(\frac{0.88\,\lambda}{n - \sqrt{n^2 - NA^2}}\right)^2 + \left(\frac{n \, . \, PH . \sqrt{2}}{NA}\right)^2}$$

References

1. Bauer, W., Helmli, F., Seewig, J., Krüger-Sehm, R. & Wiora, G. Fair Data Sheet: Definition of a Comparable Data Sheet for Optical Surface Measurement Devices, version 1.2α. (2016). Available at: http://www.optassyst.de/fairesdatenblatt/.
2. Leach, R. Some Common Terms and Definitions. in *Optical Measurement of Surface Topography* (ed. Leach, R.) 15–22 (Springer Berlin Heidelberg, 2011). doi:10.1007/978-3-642-12012-1_2
3. Wilhelm, S. *Confocal Laser Scanning Microscopy*. (Carl Zeiss MicroImaging GmbH, 2010).
4. Liutkus, A. *et al.* Imaging With Nature: Compressive Imaging Using a Multiply Scattering Medium. *Scientific Reports* **4**, 5552 (2014).
5. Unser, M. Sampling—50 Years After Shannon. *Proceedings of the IEEE* **88**, 569–587 (2000).
6. Artigas, R. Imaging Confocal Microscopy. in *Optical Measurement of Surface Topography* (ed. Leach, R.) 237–286 (Springer Berlin Heidelberg, 2011). doi:10.1007/978-3-642-12012-1_11
