# Supplementary material for: The effect of numerical aperture on quantitative use-wear studies and its implication on reproducibility
Source: Sci Rep. 2019 Apr 19;9:6313. doi: 10.1038/s41598-019-42713-w (PMC6474883; doi:10.1038/s41598-019-42713-w)

The effect of numerical aperture on quantitative use-wear studies and its implication on reproducibility

Ivan Calandra, Lisa Schunk, Konstantin Bob, Walter Gneisinger, Antonella Pedergnana, Eduardo Paixao, Andreas Hildebrandt, Joao Marreiros

Scientific Reports

Supplementary Material 2. PDF export of the results of the ConfoMap template applied to all analyzed surfaces. Editable *.mnt files (which include the unprocessed surfaces) are available on Zenodo (DOI: https://doi.org/10.5281/zenodo.1479117).

Flint FLT1-7
50x objective, NA = 0.75 or 0.95

Identity card - FLT1-7_location1_LSM_50x-...		
Name:	FLT1-7_location1_LSM_50x-0.75_replica1_2018...	
Created on:	8/15/2018 4:30:26 PM	
**Axis:**	**X**	
Length:	255	µm
Size:	3000	points
Spacing:	85.2	nm
**Axis:**	**Y**	
Length:	255	µm
Size:	3000	points
Spacing:	85.2	nm
**Axis:**	**Z**	
Length:	28.1	µm
Size:	65532	digits
Spacing:	0.428	nm
NMP ratio:	0.00 % (0 Pts)	

Extracted surface

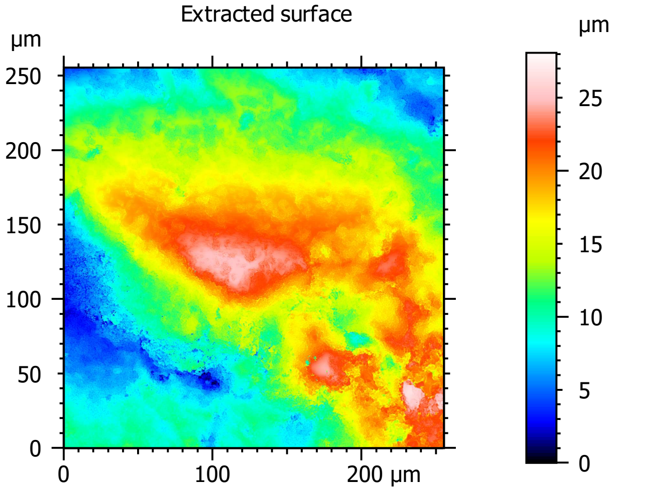

Primary surface (S Filter: Gaussian, S_1 nesting index = 0.425 µm, manage end effects)

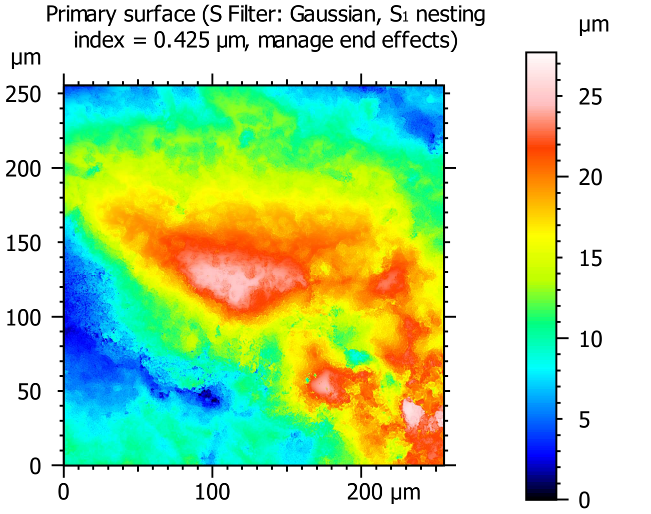

SF surface (F operator: polynomial of degree 3)

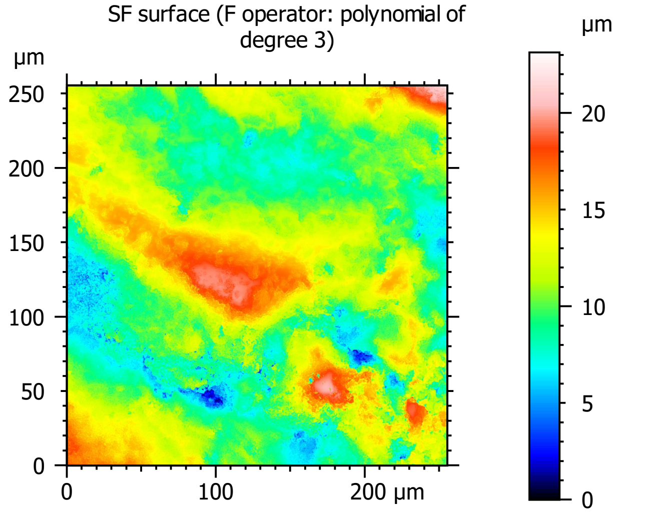

S-L surface (L filter: Gaussian, L nesting index = 127 µm, manage end effects)

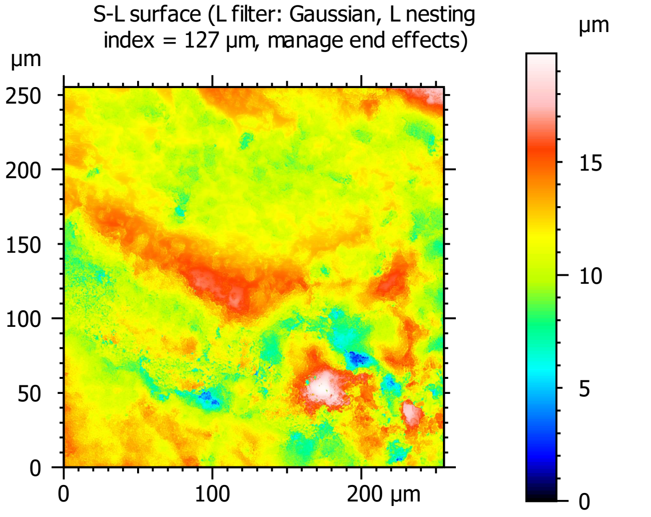

S-L surface (L filter: Gaussian, L nesting index = 127 µm, manage end effects)

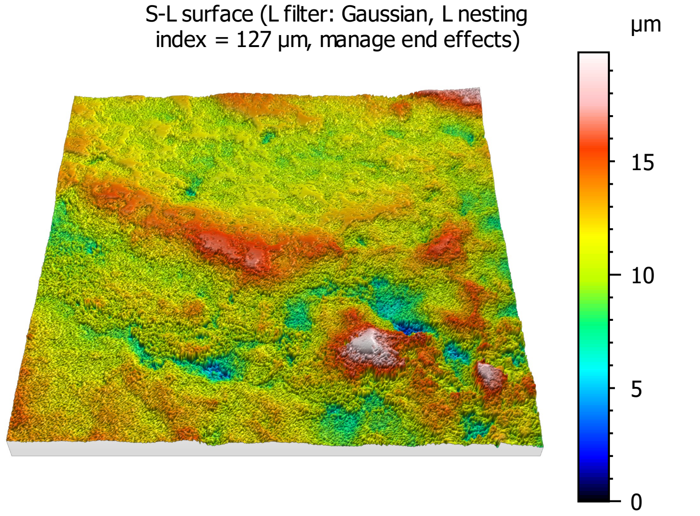

ISO 25178		
**Height Parameters**		
Sq	1.92	µm
Ssk	0.251	
Sku	4.41	
Sp	8.40	µm
Sv	11.4	µm
Sz	19.8	µm
Sa	1.45	µm
**Functional Parameters**		
Smr	0.154	%
Smc	2.42	µm
Sxp	3.56	µm
**Spatial Parameters**		
Sal	17.2	µm
Str	0.504	
Std	166	°
**Hybrid Parameters**		
Sdq	2.72	
Sdr	119	%
**Functional Parameters (V...**		
Vm	0.125	µm³/µm²
Vv	2.54	µm³/µm²
Vmp	0.125	µm³/µm²
Vmc	1.52	µm³/µm²
Vvc	2.32	µm³/µm²
Vvv	0.220	µm³/µm²
**Functional Parameters (S...**		
Sk	2.71	µm
Spk	1.74	µm
Svk	2.03	µm
Smr1	12.1	%
Smr2	87.6	%
Spq	0.442	
Svq	3.71	
Smq	55.8	

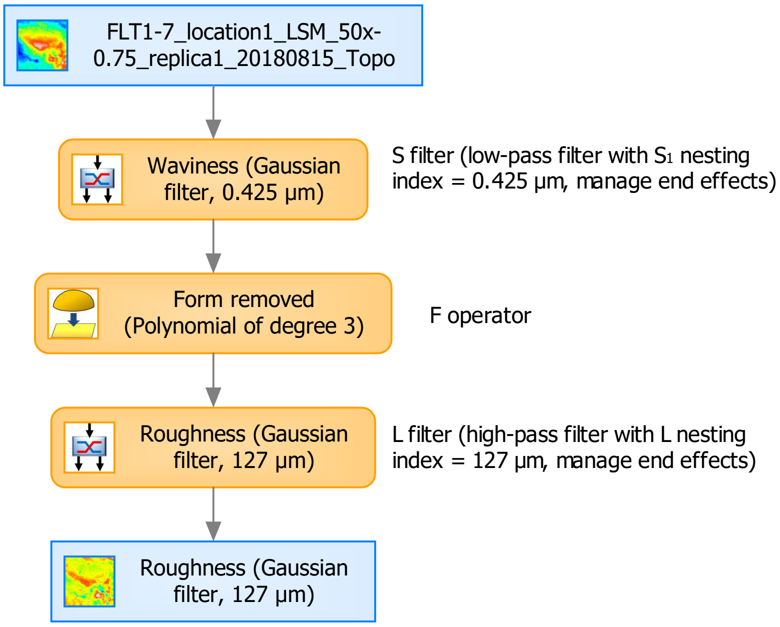

Flint FLT1-7
50x objective, NA = 0.75 or 0.95

Extracted surface

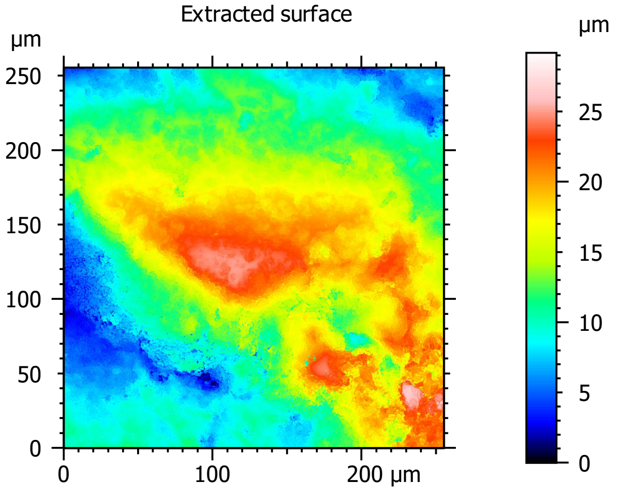

Identity card - FLT1-7_location1_LSM_50x-...		
Name:	FLT1-7_location1_LSM_50x-0.75_replica2_2018...	
Created on:	8/15/2018 4:56:32 PM	
**Axis:**	**X**	
Length:	255	µm
Size:	3000	points
Spacing:	85.2	nm
**Axis:**	**Y**	
Length:	255	µm
Size:	3000	points
Spacing:	85.2	nm
**Axis:**	**Z**	
Length:	29.2	µm
Size:	65531	digits
Spacing:	0.445	nm
NMP ratio:	0.00 % (0 Pts)	

Primary surface (S Filter: Gaussian, S_1 nesting index = 0.425 µm, manage end effects)

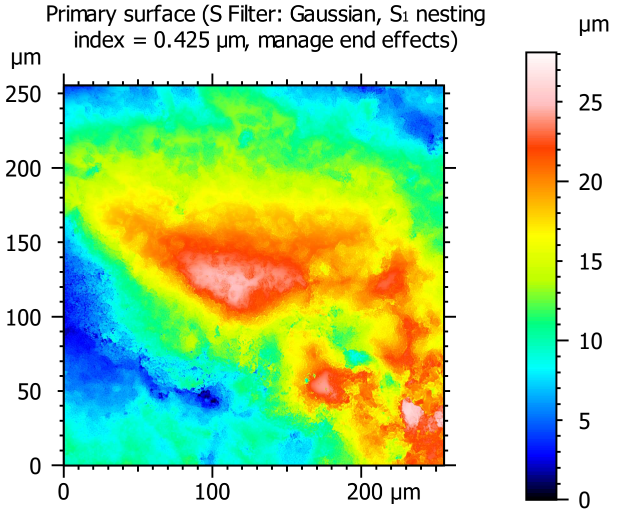

SF surface (F operator: polynomial of degree 3)

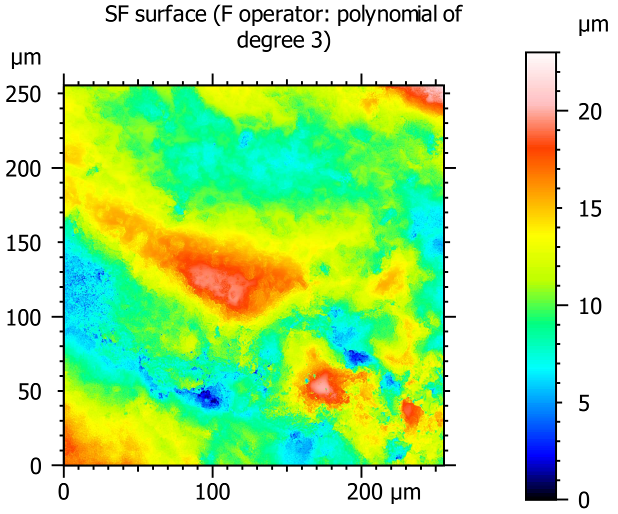

S-L surface (L filter: Gaussian, L nesting index = 127 µm, manage end effects)

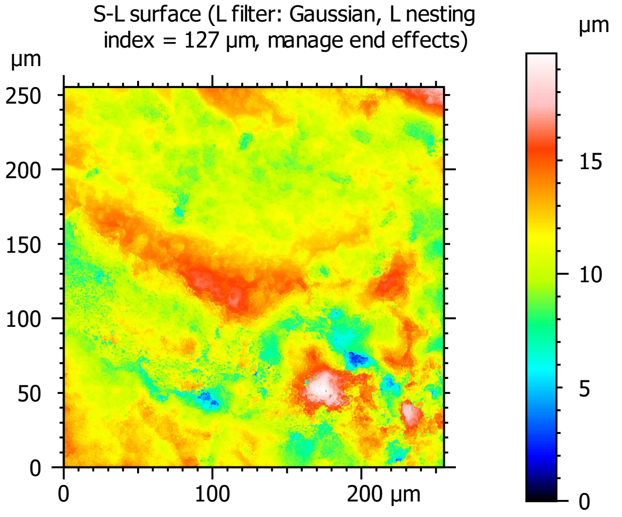

S-L surface (L filter: Gaussian, L nesting index = 127 µm, manage end effects)

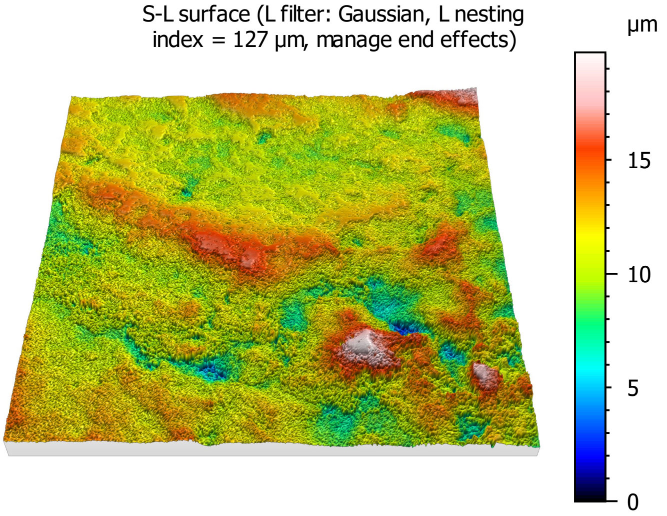

ISO 25178		
**Height Parameters**		
Sq	1.91	µm
Ssk	0.260	
Sku	4.40	
Sp	8.51	µm
Sv	11.2	µm
Sz	19.7	µm
Sa	1.44	µm
**Functional Parameters**		
Smr	0.130	%
Smc	2.40	µm
Sxp	3.52	µm
**Spatial Parameters**		
Sal	17.2	µm
Str	0.506	
Std	50.7	°
**Hybrid Parameters**		
Sdq	2.69	
Sdr	117	%
**Functional Parameters (V...**		
Vm	0.125	µm³/µm²
Vv	2.53	µm³/µm²
Vmp	0.125	µm³/µm²
Vmc	1.50	µm³/µm²
Vvc	2.31	µm³/µm²
Vvv	0.218	µm³/µm²
**Functional Parameters (S...**		
Sk	2.68	µm
Spk	1.74	µm
Svk	1.99	µm
Smr1	12.2	%
Smr2	87.5	%
Spq	1.41	
Svq	3.66	
Smq	98.1	

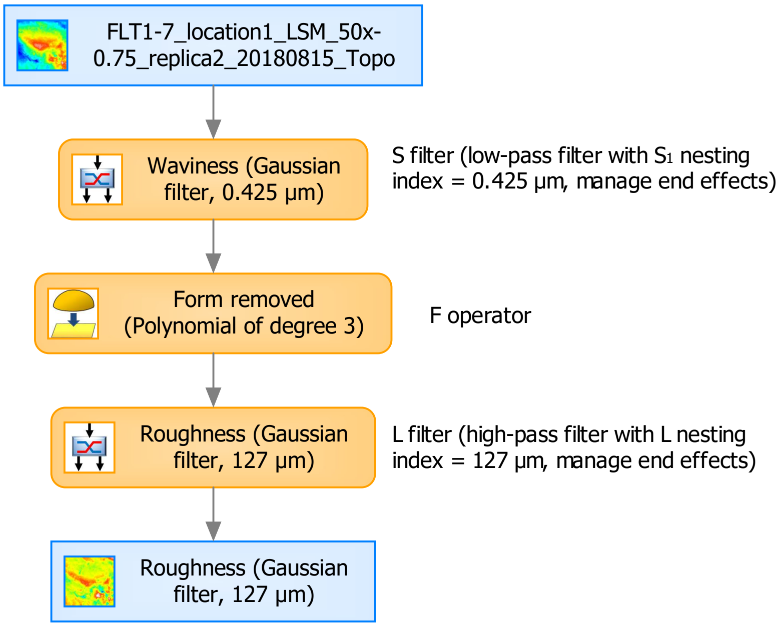

Flint FLT1-7
50x objective, NA = 0.75 or 0.95

Extracted surface

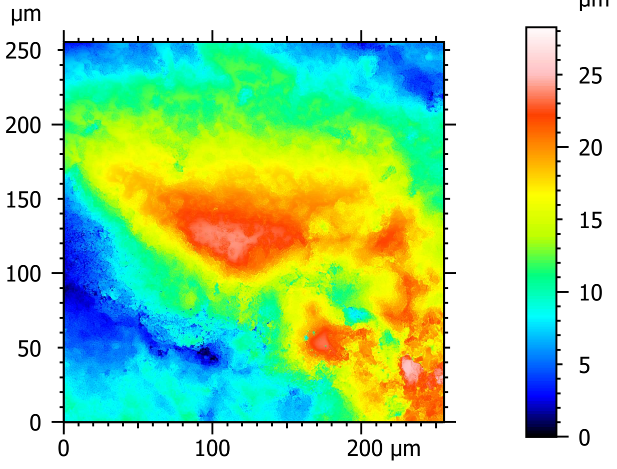

**Identity card - FLT1-7_location1_LSM_50x-...**			
Name:	FLT1-7_location1_LSM_50x-0.75_replica3_2018...		
Created on:	8/15/2018 5:17:01 PM		
**Axis:**	**X**		
Length:	255	µm	
Size:	3000	points	
Spacing:	85.2	nm	
**Axis:**	**Y**		
Length:	255	µm	
Size:	3000	points	
Spacing:	85.2	nm	
**Axis:**	**Z**		
Length:	28.3	µm	
Size:	65532	digits	
Spacing:	0.431	nm	
NMP ratio:	0.00 % (0 Pts)		

Primary surface (S Filter: Gaussian, S_1 nesting index = 0.425 µm, manage end effects)

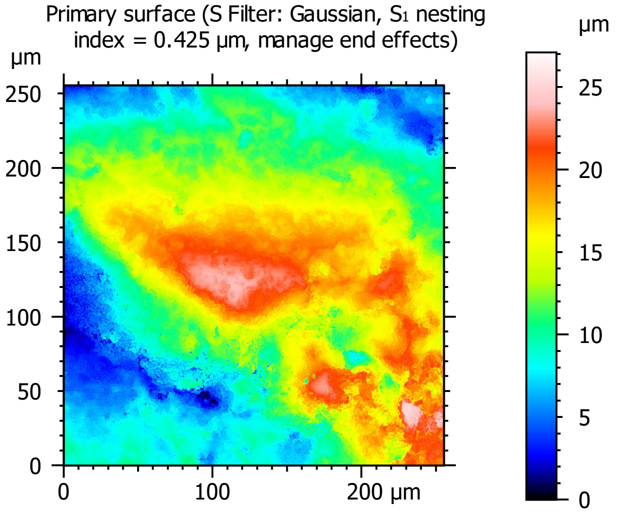

SF surface (F operator: polynomial of degree 3)

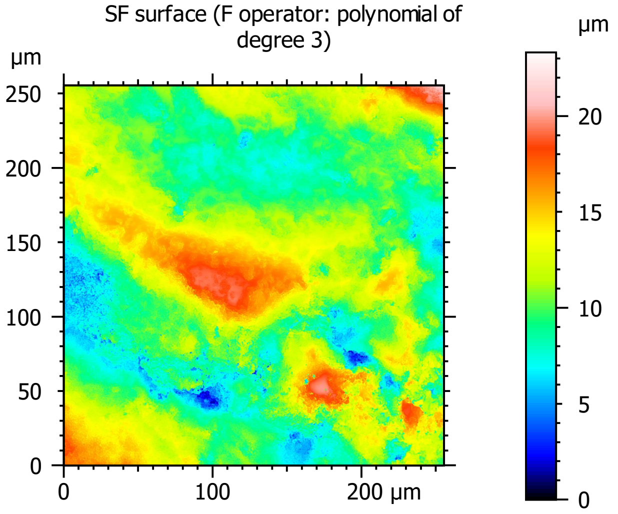

S-L surface (L filter: Gaussian, L nesting index = 127 µm, manage end effects)

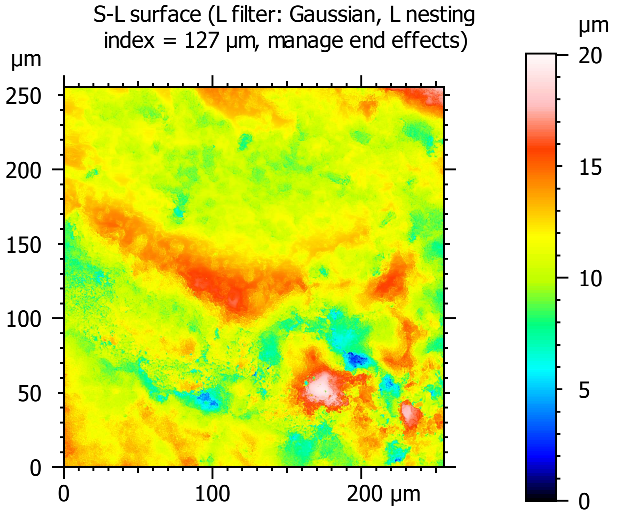

S-L surface (L filter: Gaussian, L nesting index = 127 µm, manage end effects)

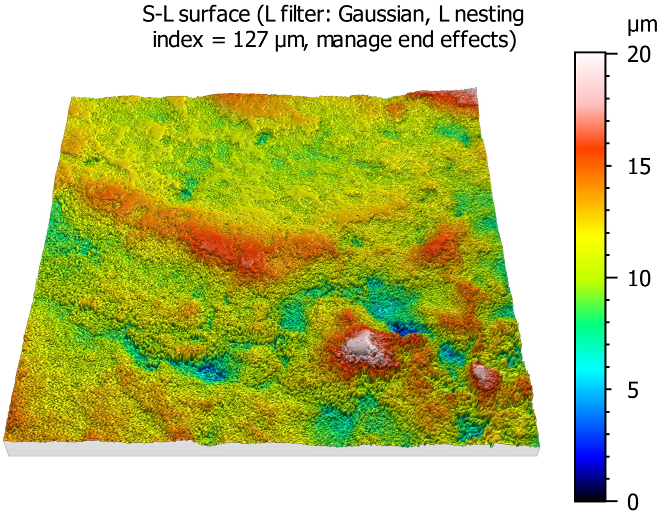

ISO 25178		
**Height Parameters**		
Sq	1.90	µm
Ssk	0.251	
Sku	4.35	
Sp	8.83	µm
Sv	11.2	µm
Sz	20.1	µm
Sa	1.43	µm
**Functional Parameters**		
Smr	0.0643	%
Smc	2.39	µm
Sxp	3.52	µm
**Spatial Parameters**		
Sal	17.2	µm
Str	0.508	
Std	167	°
**Hybrid Parameters**		
Sdq	2.67	
Sdr	116	%
**Functional Parameters (V...**		
Vm	0.123	µm³/µm²
Vv	2.51	µm³/µm²
Vmp	0.123	µm³/µm²
Vmc	1.50	µm³/µm²
Vvc	2.30	µm³/µm²
Vvv	0.218	µm³/µm²
**Functional Parameters (S...**		
Sk	2.67	µm
Spk	1.71	µm
Svk	1.98	µm
Smr1	12.2	%
Smr2	87.5	%
Spq	1.27	
Svq	3.51	
Smq	95.5	

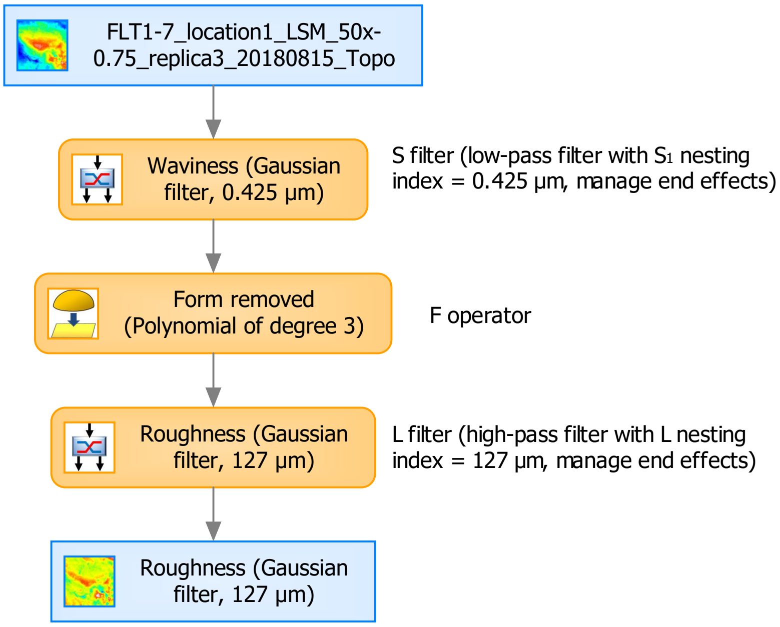

Flint FLT1-7
50x objective, NA = 0.75 or 0.95

Identity card - FLT1-7_location1_LSM_50x-...		
Name:	FLT1-7_location1_LSM_50x-0.95_replica1_2018...	
Created on:	8/15/2018 4:17:30 PM	
**Axis:**	**X**	
Length:	255	µm
Size:	3000	points
Spacing:	85.2	nm
**Axis:**	**Y**	
Length:	255	µm
Size:	3000	points
Spacing:	85.2	nm
**Axis:**	**Z**	
Length:	26.1	µm
Size:	65532	digits
Spacing:	0.398	nm
NMP ratio:	0.00 % (0 Pts)	

Extracted surface

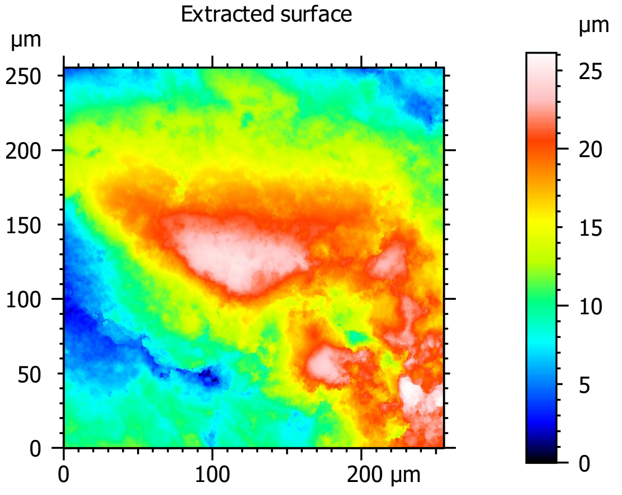

Primary surface (S Filter: Gaussian, S1 nesting index = 0.425 µm, manage end effects)

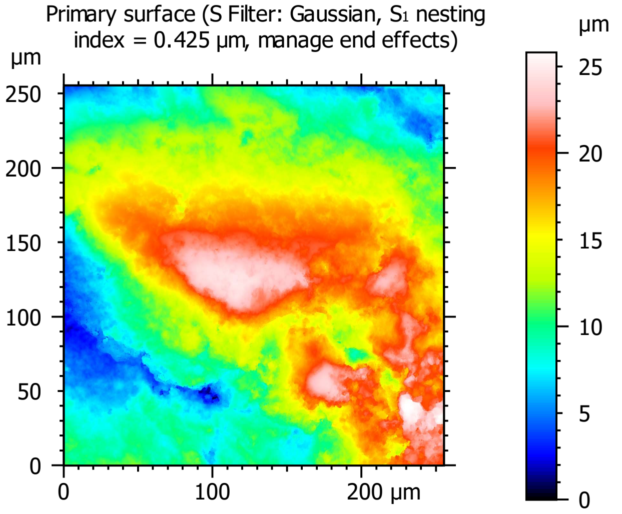

SF surface (F operator: polynomial of degree 3)

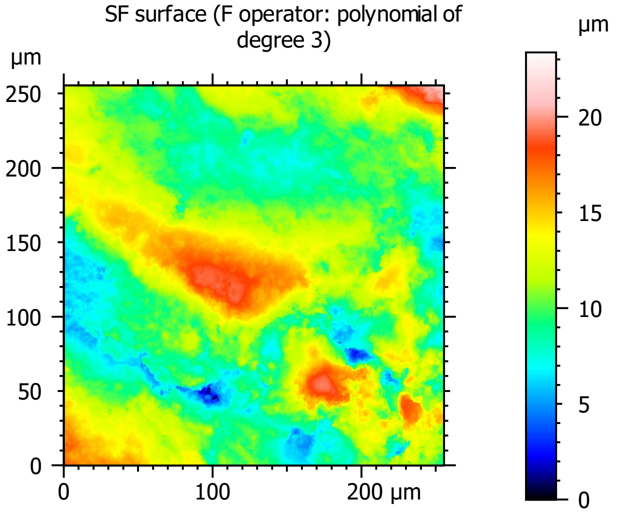

S-L surface (L filter: Gaussian, L nesting index = 127 µm, manage end effects)

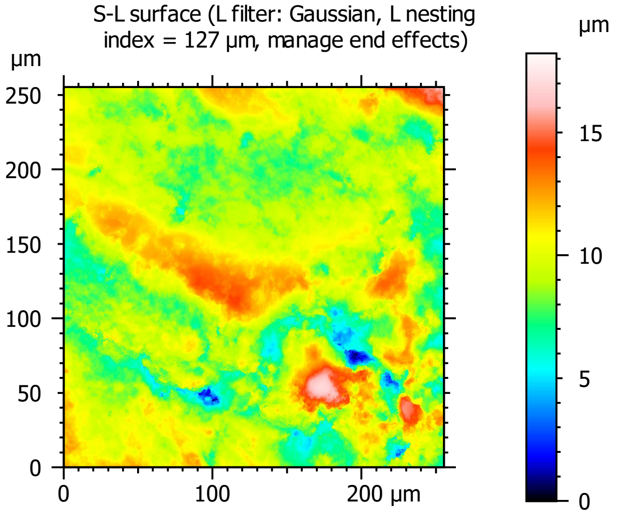

S-L surface (L filter: Gaussian, L nesting index = 127 µm, manage end effects)

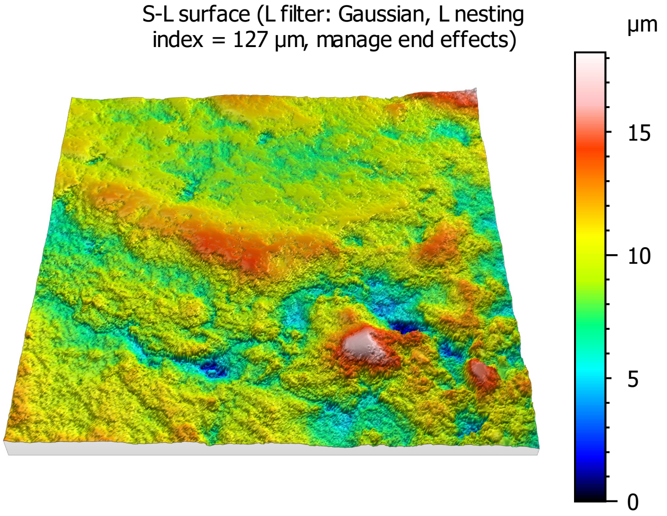

ISO 25178		
**Height Parameters**		
Sq	1.83	µm
Ssk	0.301	
Sku	4.43	
Sp	8.80	µm
Sv	9.43	µm
Sz	18.2	µm
Sa	1.38	µm
**Functional Parameters**		
Smr	0.00289	%
Smc	2.34	µm
Sxp	3.29	µm
**Spatial Parameters**		
Sal	17.5	µm
Str	0.489	
Std	92.8	°
**Hybrid Parameters**		
Sdq	1.26	
Sdr	33.7	%
**Functional Parameters (Vol...**		
Vm	0.117	µm³/µm²
Vv	2.46	µm³/µm²
Vmp	0.117	µm³/µm²
Vmc	1.42	µm³/µm²
Vvc	2.26	µm³/µm²
Vvv	0.204	µm³/µm²
**Functional Parameters (Str...**		
Sk	2.35	µm
Spk	1.65	µm
Svk	1.83	µm
Smr1	13.2	%
Smr2	87.0	%
Spq	0.201	
Svq	3.59	
Smq	53.2	

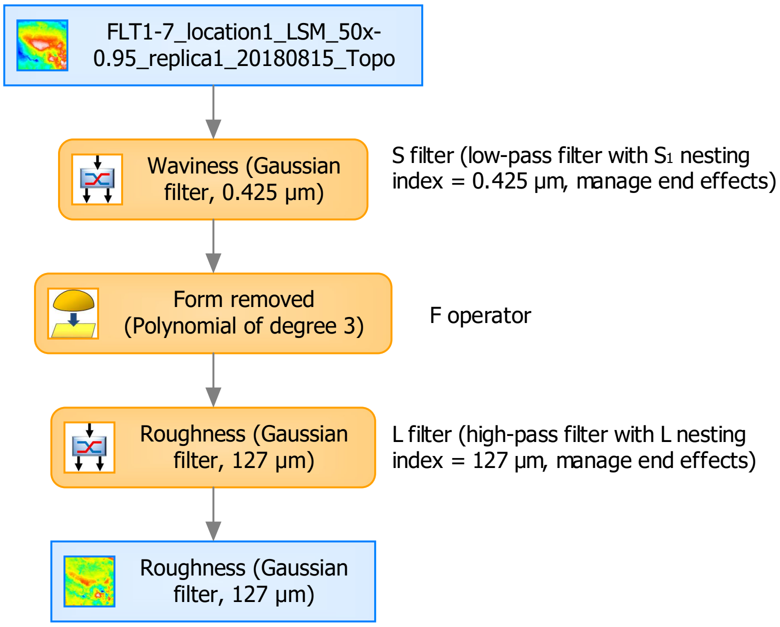

Flint FLT1-7
50x objective, NA = 0.75 or 0.95

**Identity card - FLT1-7_location1_LSM_50x-...**		
Name:	FLT1-7_location1_LSM_50x-0.95_replica2_2018...	
Created on:	8/15/2018 4:44:00 PM	
**Axis:**	**X**	
Length:	255	µm
Size:	3000	points
Spacing:	85.2	nm
**Axis:**	**Y**	
Length:	255	µm
Size:	3000	points
Spacing:	85.2	nm
**Axis:**	**Z**	
Length:	26.4	µm
Size:	65532	digits
Spacing:	0.403	nm
NMP ratio:	0.00 % (0 Pts)	

Extracted surface

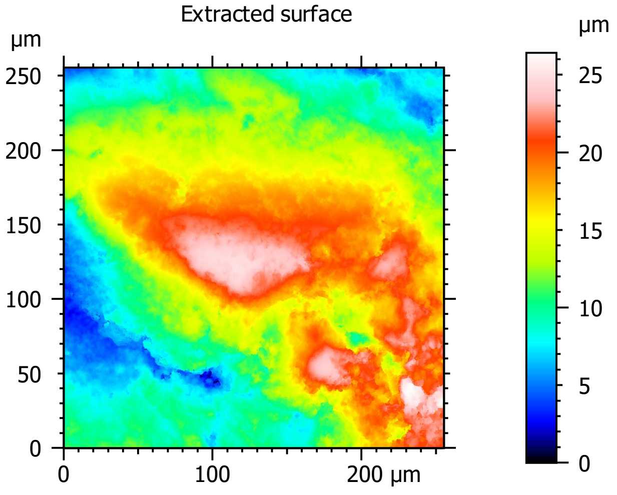

Primary surface (S Filter: Gaussian, S1 nesting index = 0.425 µm, manage end effects)

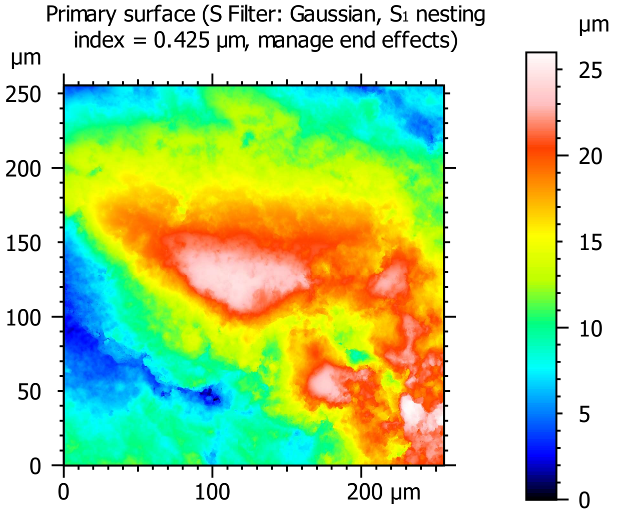

SF surface (F operator: polynomial of degree 3)

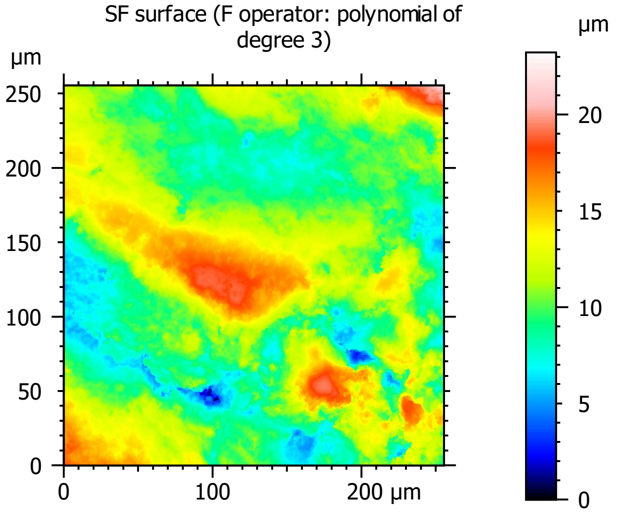

S-L surface (L filter: Gaussian, L nesting index = 127 µm, manage end effects)

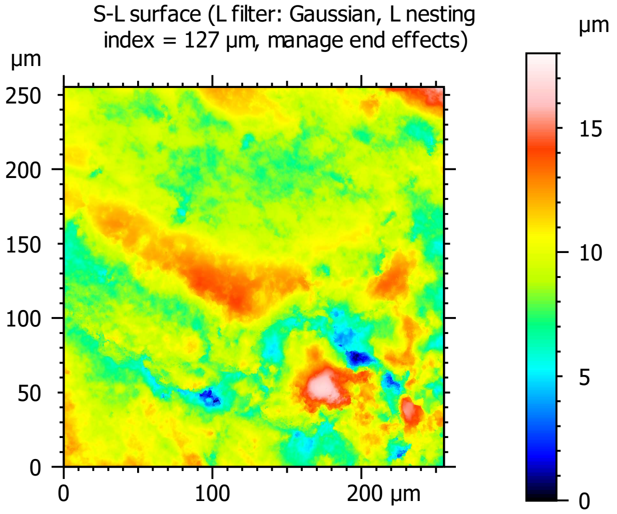

S-L surface (L filter: Gaussian, L nesting index = 127 µm, manage end effects)

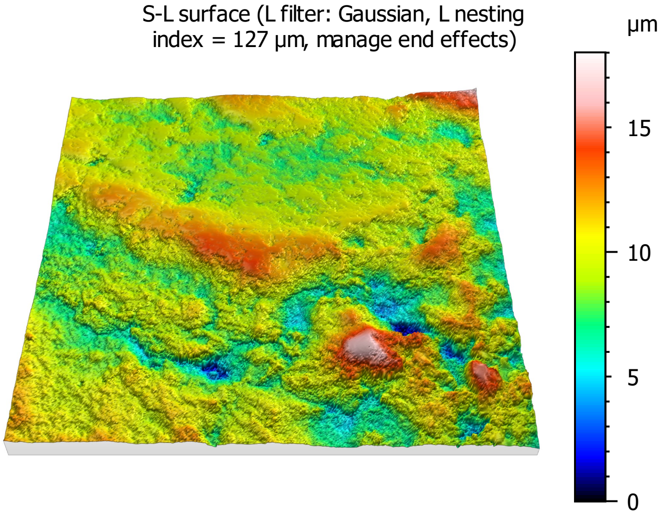

ISO 25178		
**Height Parameters**		
Sq	1.80	µm
Ssk	0.289	
Sku	4.42	
Sp	8.68	µm
Sv	9.33	µm
Sz	18.0	µm
Sa	1.36	µm
**Functional Parameters**		
Smr	0.00222	%
Smc	2.32	µm
Sxp	3.25	µm
**Spatial Parameters**		
Sal	17.6	µm
Str	0.484	
Std	92.8	°
**Hybrid Parameters**		
Sdq	1.24	
Sdr	33.1	%
**Functional Parameters (Vol...**		
Vm	0.115	µm³/µm²
Vv	2.43	µm³/µm²
Vmp	0.115	µm³/µm²
Vmc	1.41	µm³/µm²
Vvc	2.23	µm³/µm²
Vvv	0.202	µm³/µm²
**Functional Parameters (Str...**		
Sk	2.34	µm
Spk	1.64	µm
Svk	1.82	µm
Smr1	12.8	%
Smr2	87.1	%
Spq	0.0523	
Svq	3.61	
Smq	46.8	

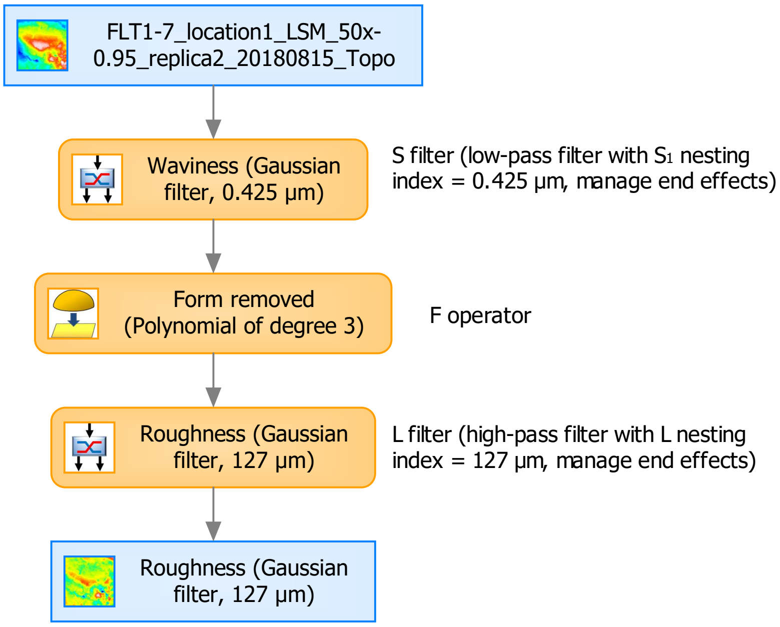

Flint FLT1-7
50x objective, NA = 0.75 or 0.95

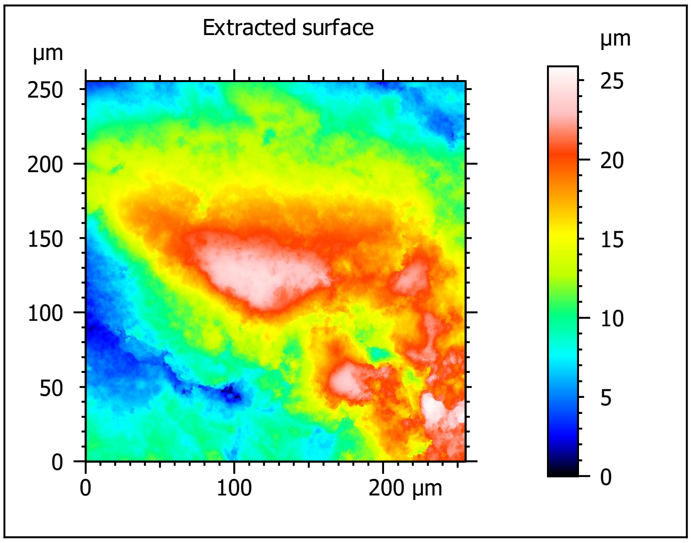

Identity card - FLT1-7_location1_LSM_50x-...

Name:	FLT1-7_location1_LSM_50x-0.95_replica3_2018...	
Created on:	8/15/2018 5:06:24 PM	
**Axis:**	**X**	
Length:	255	µm
Size:	3000	points
Spacing:	85.2	nm
**Axis:**	**Y**	
Length:	255	µm
Size:	3000	points
Spacing:	85.2	nm
**Axis:**	**Z**	
Length:	25.9	µm
Size:	65532	digits
Spacing:	0.395	nm
NMP ratio:	0.00 % (0 Pts)	

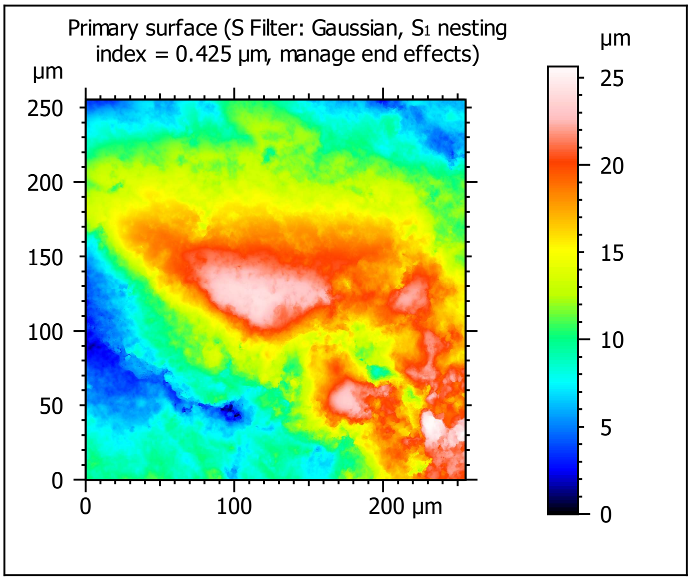

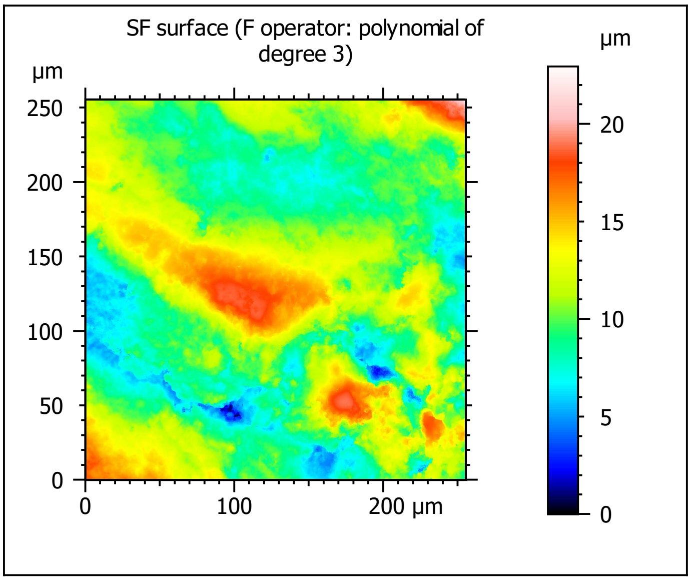

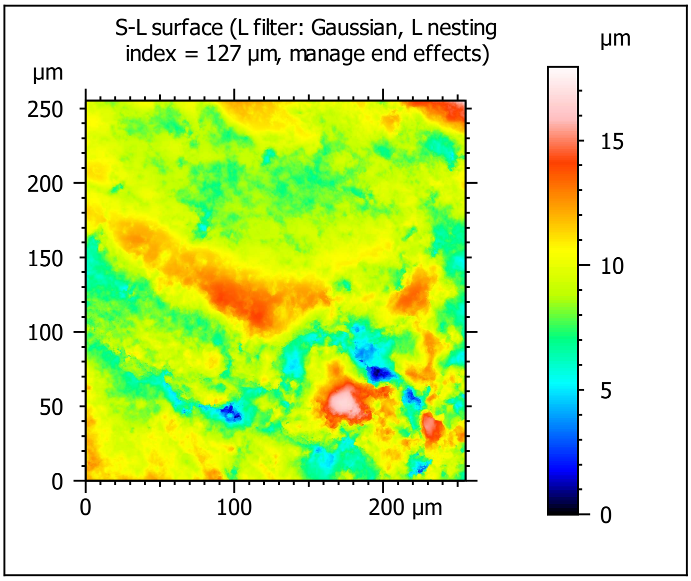

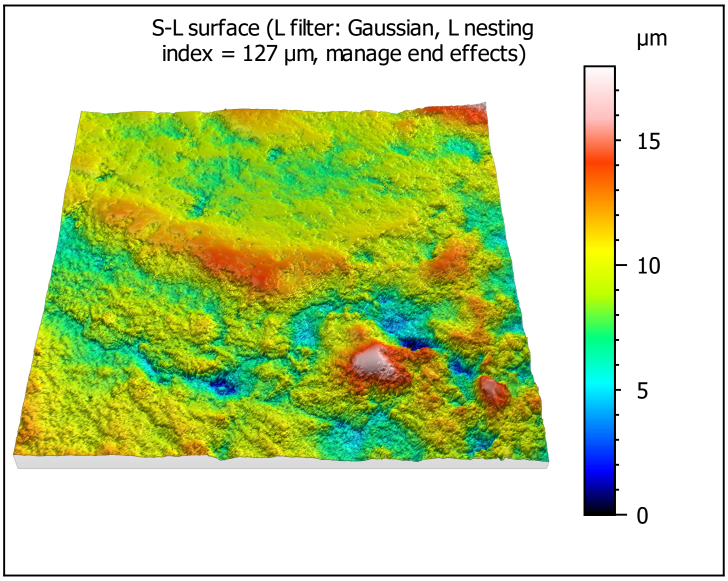

ISO 25178		
**Height Parameters**		
Sq	1.80	µm
Ssk	0.284	
Sku	4.39	
Sp	8.70	µm
Sv	9.26	µm
Sz	18.0	µm
Sa	1.36	µm
**Functional Parameters**		
Smr	0.00444	%
Smc	2.32	µm
Sxp	3.25	µm
**Spatial Parameters**		
Sal	17.6	µm
Str	0.484	
Std	92.8	°
**Hybrid Parameters**		
Sdq	1.24	
Sdr	32.8	%
**Functional Parameters (Vol...**		
Vm	0.115	µm³/µm²
Vv	2.43	µm³/µm²
Vmp	0.115	µm³/µm²
Vmc	1.41	µm³/µm²
Vvc	2.23	µm³/µm²
Vvv	0.202	µm³/µm²
**Functional Parameters (Str...**		
Sk	2.34	µm
Spk	1.63	µm
Svk	1.82	µm
Smr1	12.8	%
Smr2	87.1	%
Spq	0.0791	
Svq	3.58	
Smq	47.3	

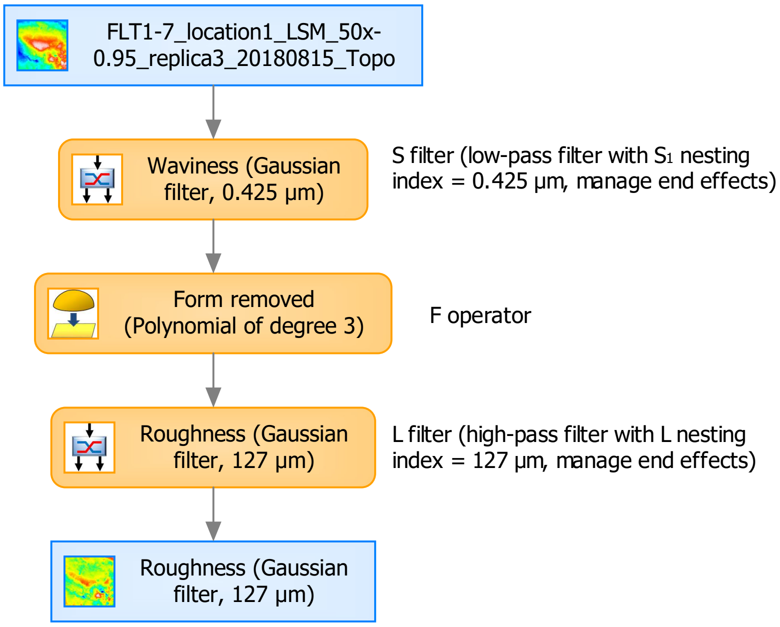

Flint FLT1-7
50x objective, NA = 0.75 or 0.95

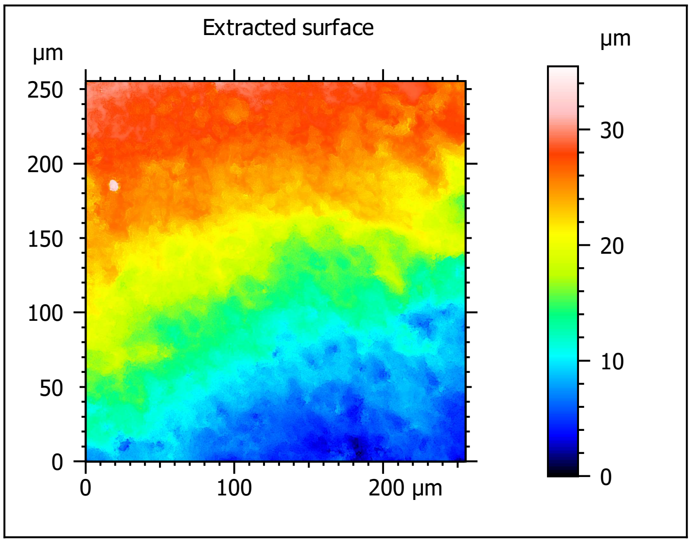

Identity card - FLT1-7_location2_LSM_50x-...			
Name:	FLT1-7_location2_LSM_50x-0.75_replica1_2018...		
Created on:	8/16/2018 3:35:16 PM		
**Axis:**	**X**		
Length:	255	µm	
Size:	3000	points	
Spacing:	85.2	nm	
**Axis:**	**Y**		
Length:	255	µm	
Size:	3000	points	
Spacing:	85.2	nm	
**Axis:**	**Z**		
Length:	35.5	µm	
Size:	65532	digits	
Spacing:	0.541	nm	
NMP ratio:	0.00 % (0 Pts)		

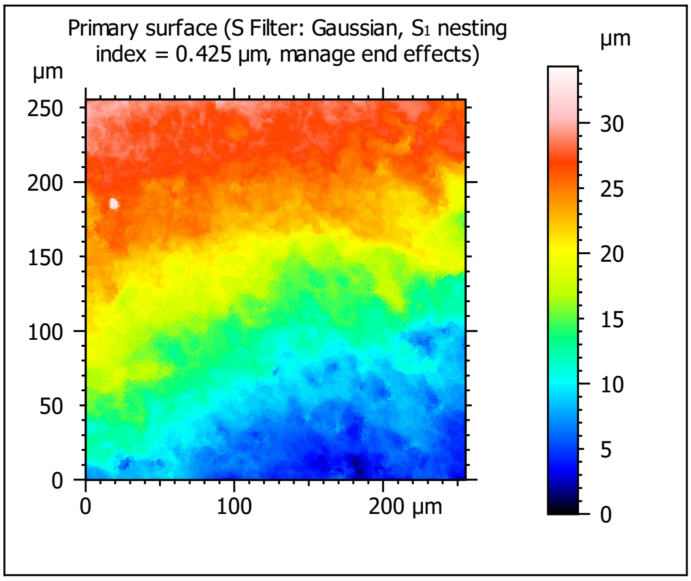

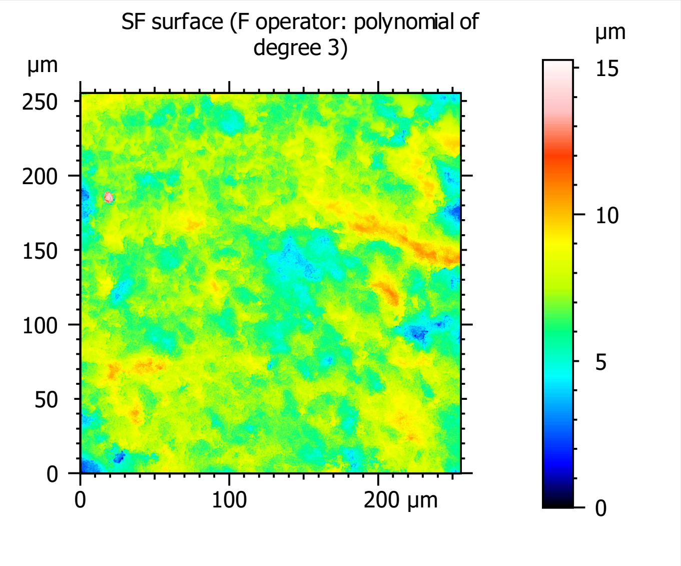

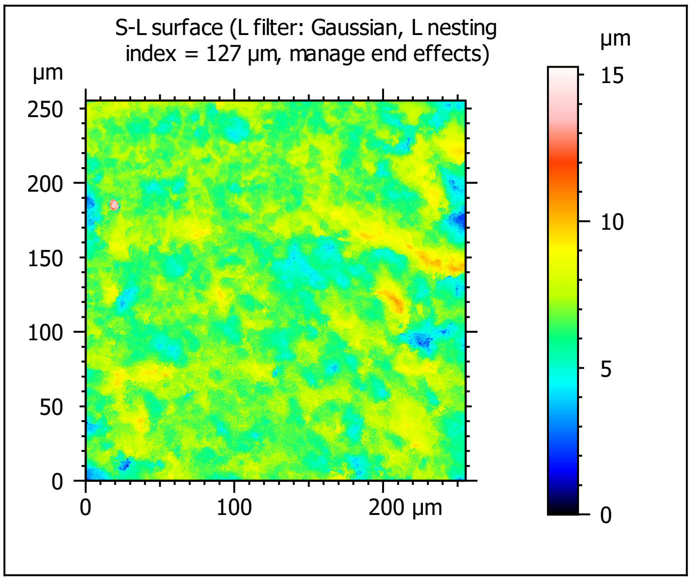

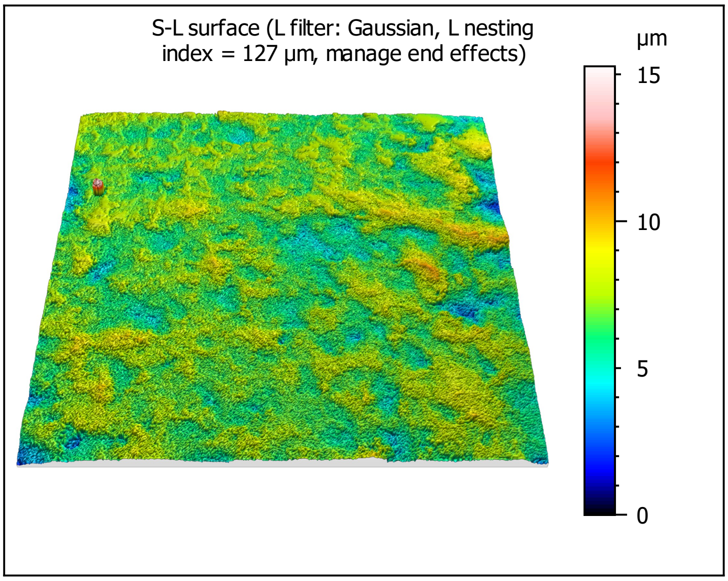

ISO 25178		
**Height Parameters**		
Sq	1.00	µm
Ssk	-0.144	
Sku	5.02	
Sp	8.52	µm
Sv	6.75	µm
Sz	15.3	µm
Sa	0.754	µm
**Functional Parameters**		
Smr	0.000783	%
Smc	1.17	µm
Sxp	2.17	µm
**Spatial Parameters**		
Sal	10.9	µm
Str	0.780	
Std	132	°
**Hybrid Parameters**		
Sdq	1.95	
Sdr	90.4	%
**Functional Parameters (Vol...**		
Vm	0.0561	µm^3/µm^2
Vv	1.23	µm^3/µm^2
Vmp	0.0561	µm^3/µm^2
Vmc	0.805	µm^3/µm^2
Vvc	1.09	µm^3/µm^2
Vvv	0.139	µm^3/µm^2
**Functional Parameters (Str...**		
Sk	1.98	µm
Spk	0.990	µm
Svk	1.12	µm
Smr1	10.0	%
Smr2	87.9	%
Spq	*****	
Svq	*****	
Smq	*****	

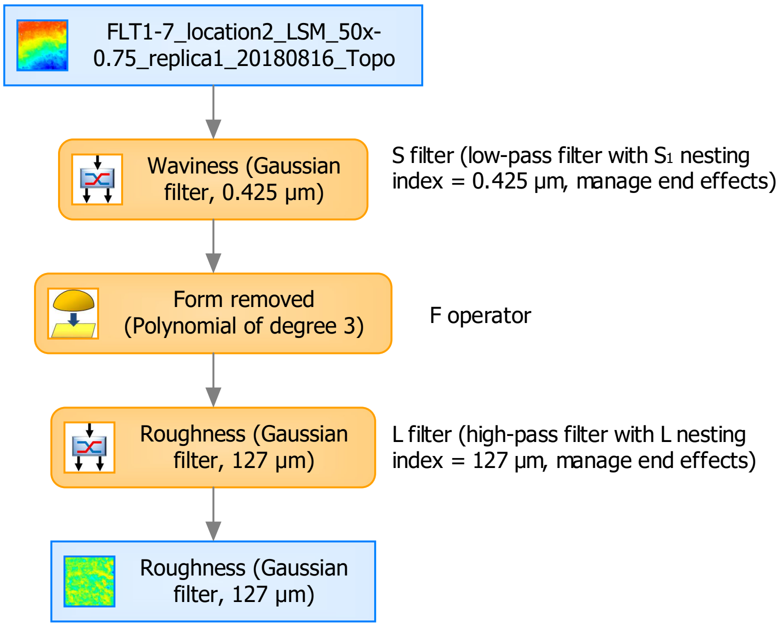

Flint FLT1-7
50x objective, NA = 0.75 or 0.95

Identity card - FLT1-7_location2_LSM_50x-...		
Name:	FLT1-7_location2_LSM_50x-0.75_replica2_2018...	
Created on:	8/16/2018 4:01:40 PM	
**Axis:**	**X**	
Length:	255	µm
Size:	3000	points
Spacing:	85.2	nm
**Axis:**	**Y**	
Length:	255	µm
Size:	3000	points
Spacing:	85.2	nm
**Axis:**	**Z**	
Length:	34.6	µm
Size:	65532	digits
Spacing:	0.528	nm
NMP ratio:	0.00 % (0 Pts)	

Extracted surface

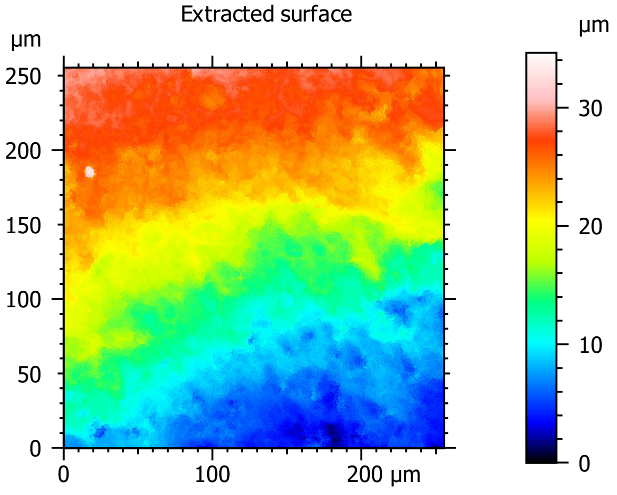

Primary surface (S Filter: Gaussian, S_1 nesting index = 0.425 µm, manage end effects)

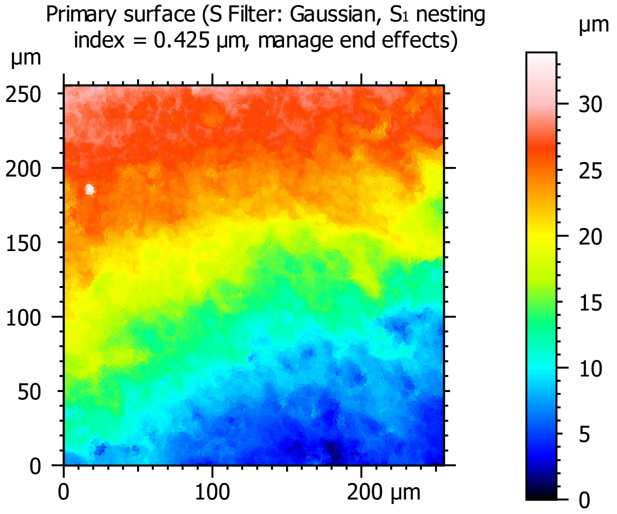

SF surface (F operator: polynomial of degree 3)

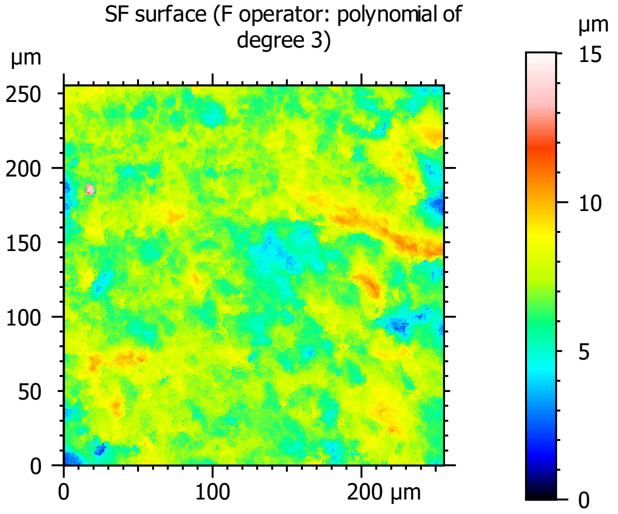

S-L surface (L filter: Gaussian, L nesting index = 127 µm, manage end effects)

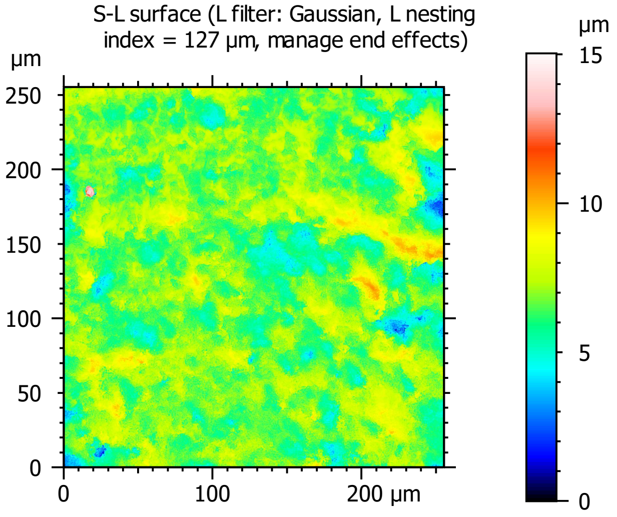

S-L surface (L filter: Gaussian, L nesting index = 127 µm, manage end effects)

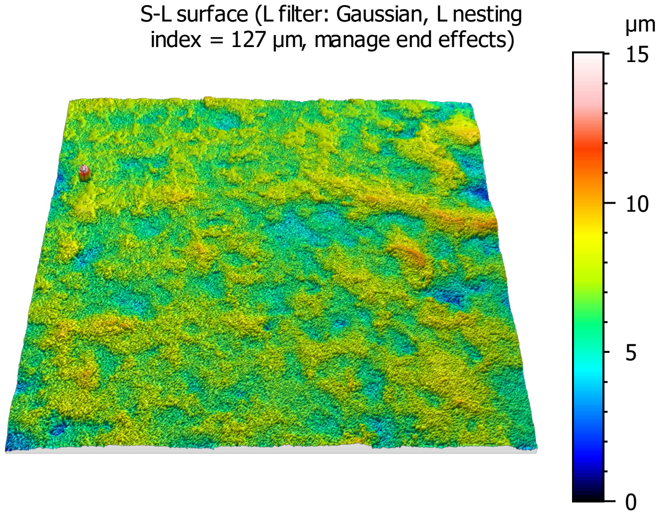

ISO 25178		
**Height Parameters**		
Sq	1.01	µm
Ssk	-0.129	
Sku	4.98	
Sp	8.29	µm
Sv	6.75	µm
Sz	15.0	µm
Sa	0.757	µm
**Functional Parameters**		
Smr	0.0035	%
Smc	1.18	µm
Sxp	2.17	µm
**Spatial Parameters**		
Sal	10.9	µm
Str	0.766	
Std	130	°
**Hybrid Parameters**		
Sdq	1.94	
Sdr	90.3	%
**Functional Parameters (V...**		
Vm	0.057	µm^3/µm^2
Vv	1.24	µm^3/µm^2
Vmp	0.057	µm^3/µm^2
Vmc	0.807	µm^3/µm^2
Vvc	1.10	µm^3/µm^2
Vvv	0.138	µm^3/µm^2
**Functional Parameters (S...**		
Sk	1.99	µm
Spk	0.998	µm
Svk	1.12	µm
Smr1	10.0	%
Smr2	88.1	%
Spq	*****	
Svq	*****	
Smq	*****	

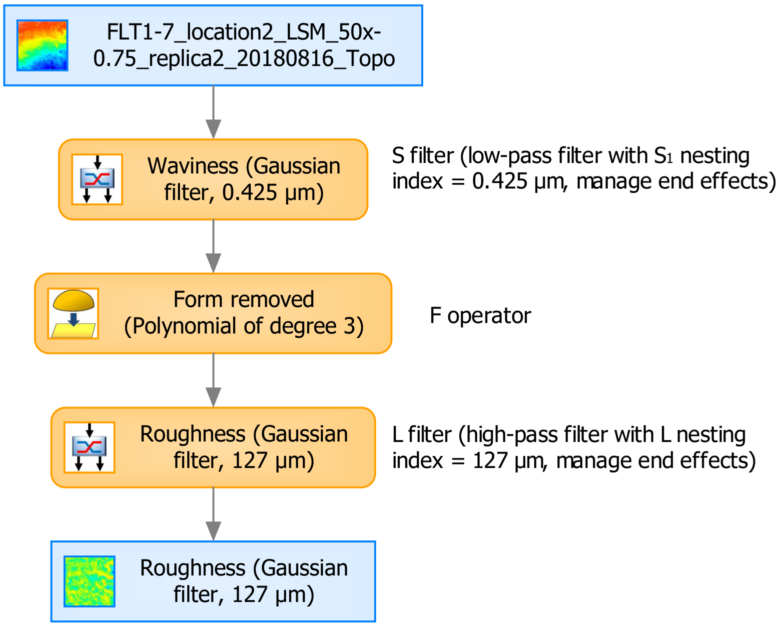

Flint FLT1-7
50x objective, NA = 0.75 or 0.95

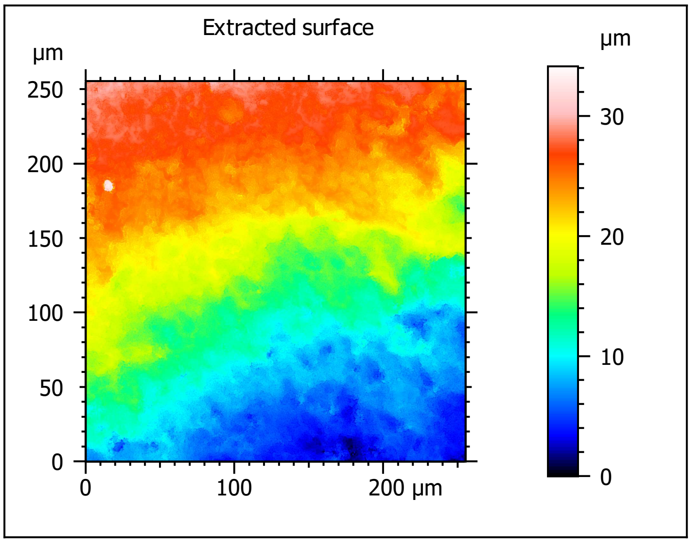

Identity card - FLT1-7_location2_LSM_50x-...		
Name:	FLT1-7_location2_LSM_50x-0.75_replica3_2018...	
Created on:	8/16/2018 4:28:37 PM	
**Axis:**	**X**	
Length:	255	µm
Size:	3000	points
Spacing:	85.2	nm
**Axis:**	**Y**	
Length:	255	µm
Size:	3000	points
Spacing:	85.2	nm
**Axis:**	**Z**	
Length:	34.1	µm
Size:	65532	digits
Spacing:	0.521	nm
NMP ratio:	0.00 % (0 Pts)	

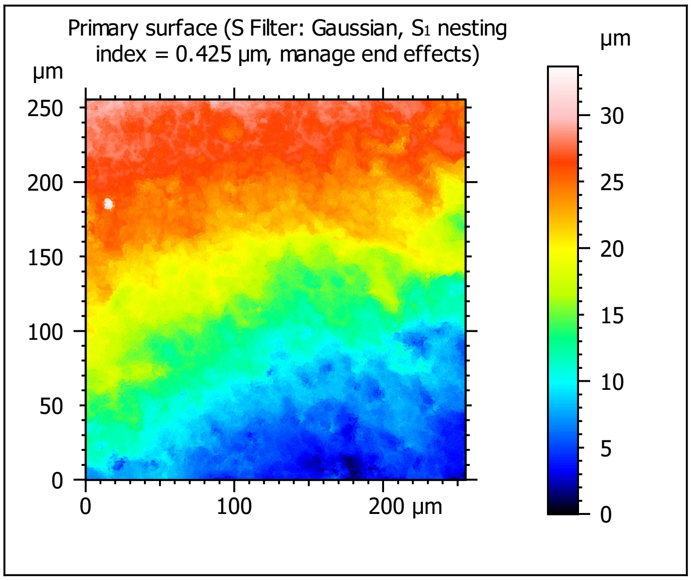

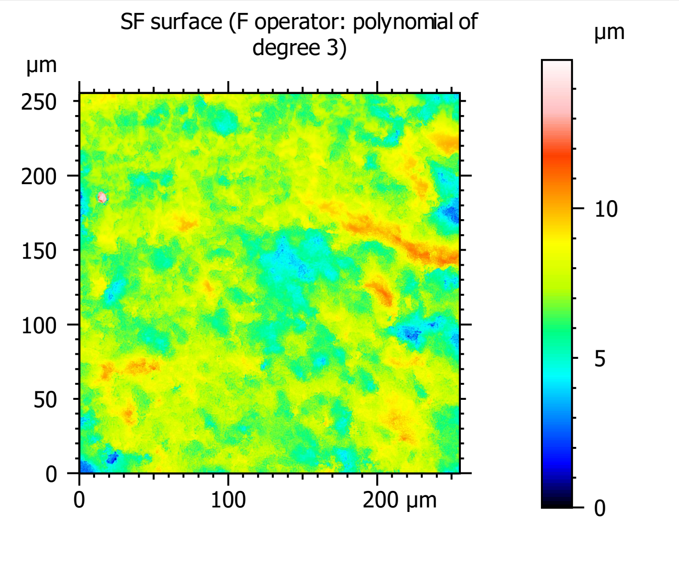

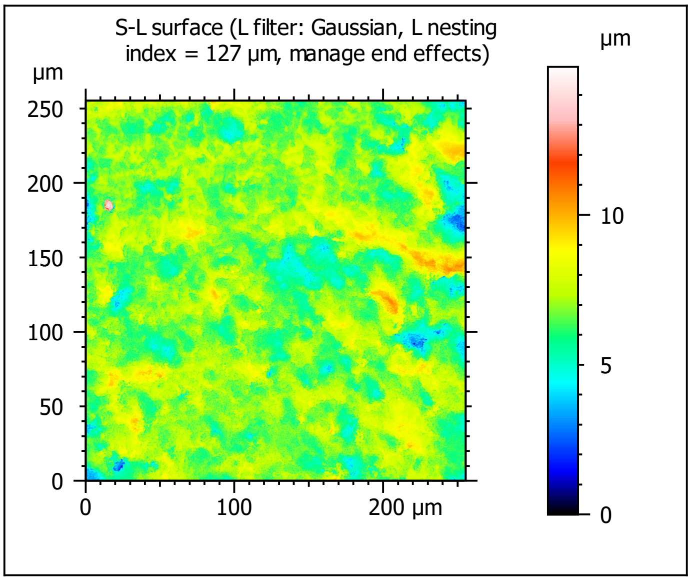

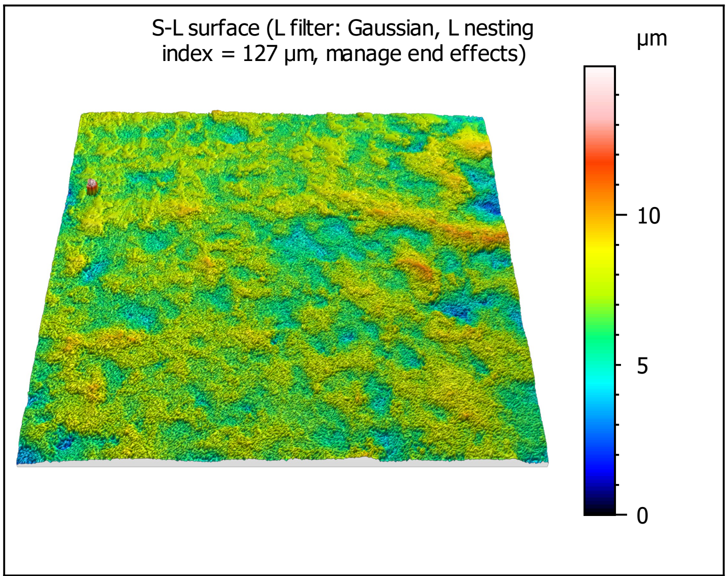

ISO 25178		
**Height Parameters**		
Sq	1.01	µm
Ssk	-0.109	
Sku	4.87	
Sp	8.09	µm
Sv	6.86	µm
Sz	14.9	µm
Sa	0.763	µm
**Functional Parameters**		
Smr	0.00638	%
Smc	1.19	µm
Sxp	2.17	µm
**Spatial Parameters**		
Sal	10.9	µm
Str	0.738	
Std	130	°
**Hybrid Parameters**		
Sdq	1.94	
Sdr	90.7	%
**Functional Parameters (Vol...**		
Vm	0.0583	µm³/µm²
Vv	1.25	µm³/µm²
Vmp	0.0583	µm³/µm²
Vmc	0.814	µm³/µm²
Vvc	1.11	µm³/µm²
Vvv	0.138	µm³/µm²
**Functional Parameters (Str...**		
Sk	2.01	µm
Spk	1.02	µm
Svk	1.13	µm
Smr1	9.96	%
Smr2	88.2	%
Spq	*****	
Svq	*****	
Smq	*****	

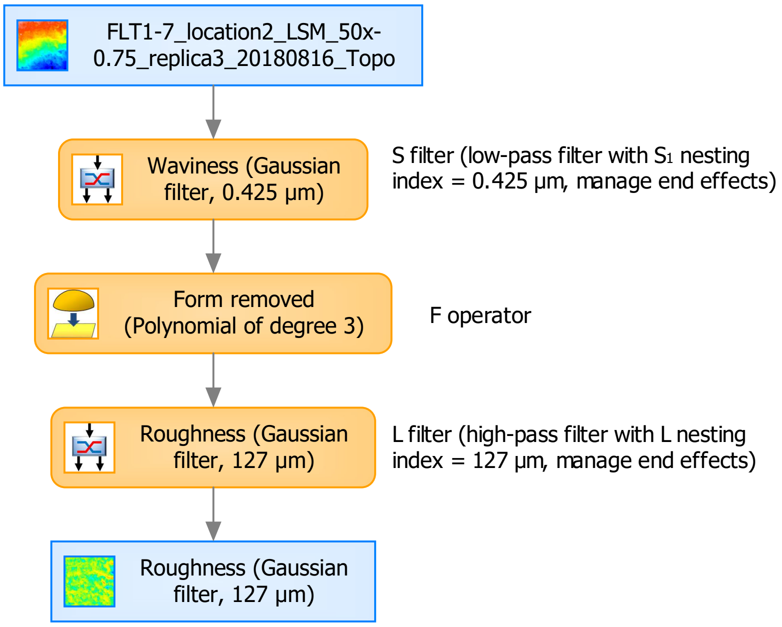

Flint FLT1-7
50x objective, NA = 0.75 or 0.95

Identity card - FLT1-7_location2_LSM_50x-...		
Name:	FLT1-7_location2_LSM_50x-0.95_replica1_2018...	
Created on:	8/16/2018 3:22:20 PM	
**Axis:**	**X**	
Length:	255	µm
Size:	3000	points
Spacing:	85.2	nm
**Axis:**	**Y**	
Length:	255	µm
Size:	3000	points
Spacing:	85.2	nm
**Axis:**	**Z**	
Length:	33.6	µm
Size:	65532	digits
Spacing:	0.512	nm
NMP ratio:	0.00 % (0 Pts)	

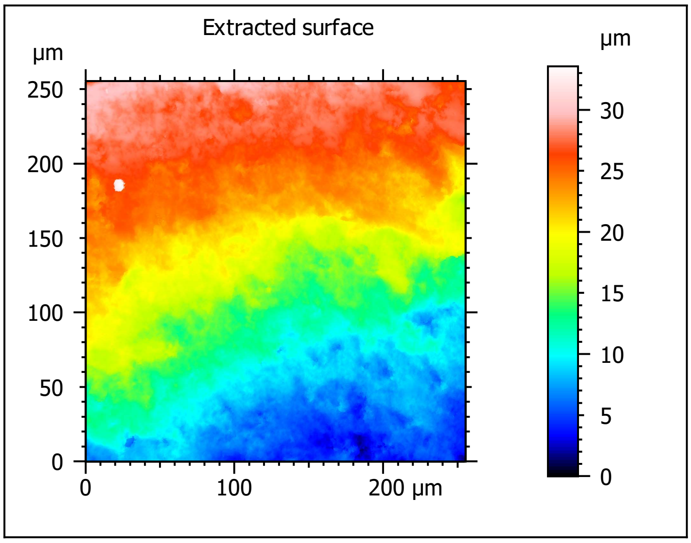

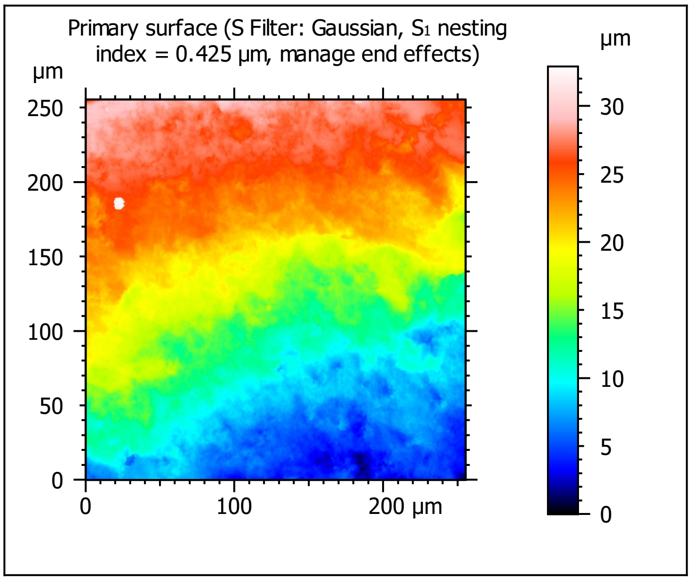

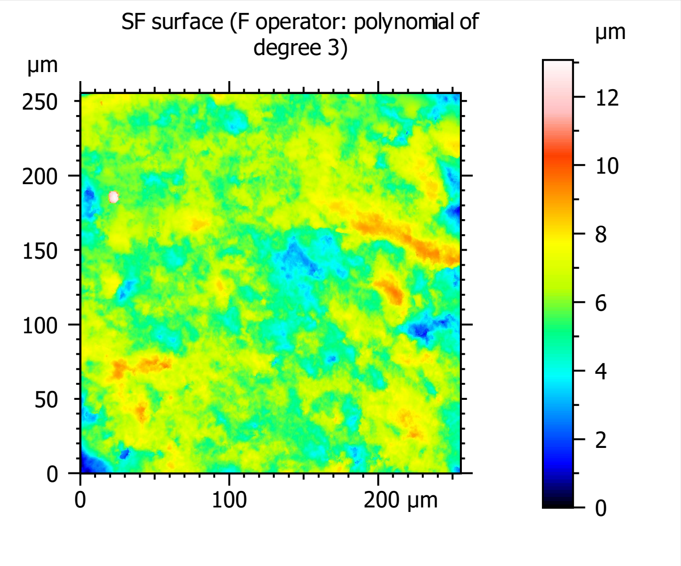

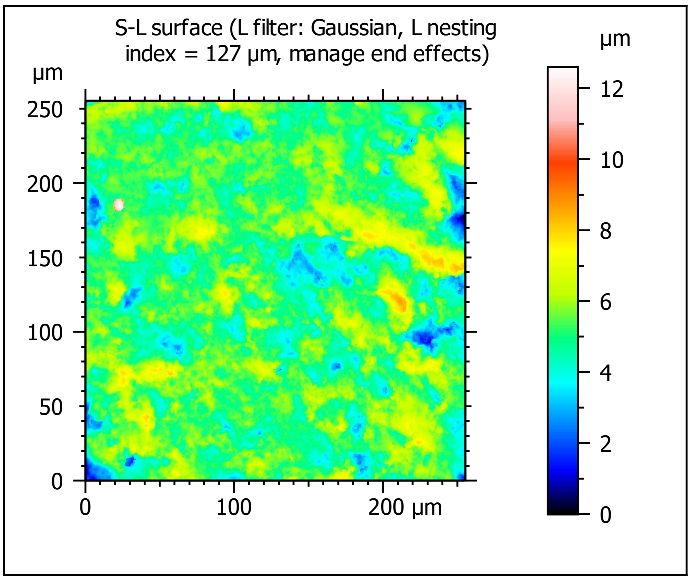

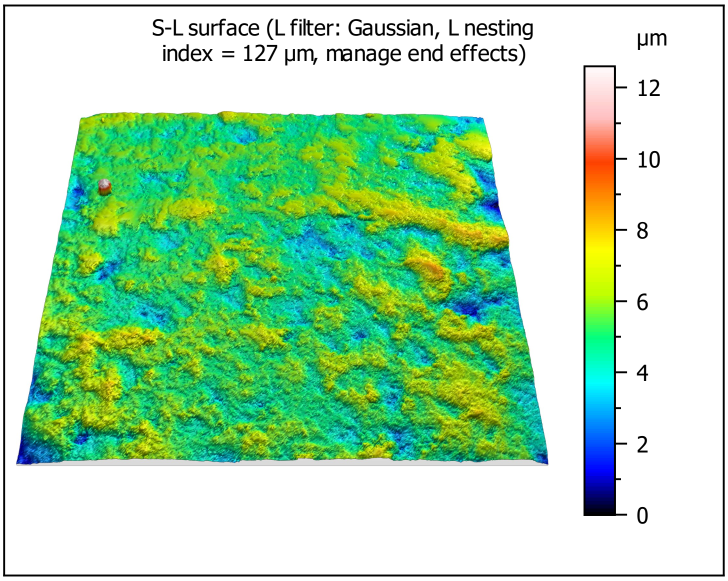

ISO 25178		
**Height Parameters**		
Sq	0.930	µm
Ssk	-0.0849	
Sku	5.76	
Sp	7.38	µm
Sv	5.22	µm
Sz	12.6	µm
Sa	0.689	µm
**Functional Parameters**		
Smr	0.0454	%
Smc	1.08	µm
Sxp	2.04	µm
**Spatial Parameters**		
Sal	11.4	µm
Str	0.780	
Std	176	°
**Hybrid Parameters**		
Sdq	0.858	
Sdr	22.2	%
**Functional Parameters (V...**		
Vm	0.0537	µm³/µm²
Vv	1.13	µm³/µm²
Vmp	0.0537	µm³/µm²
Vmc	0.728	µm³/µm²
Vvc	0.999	µm³/µm²
Vvv	0.132	µm³/µm²
**Functional Parameters (S...**		
Sk	1.72	µm
Spk	0.962	µm
Svk	1.03	µm
Smr1	10.5	%
Smr2	87.3	%
Spq	*****	
Svq	*****	
Smq	*****	

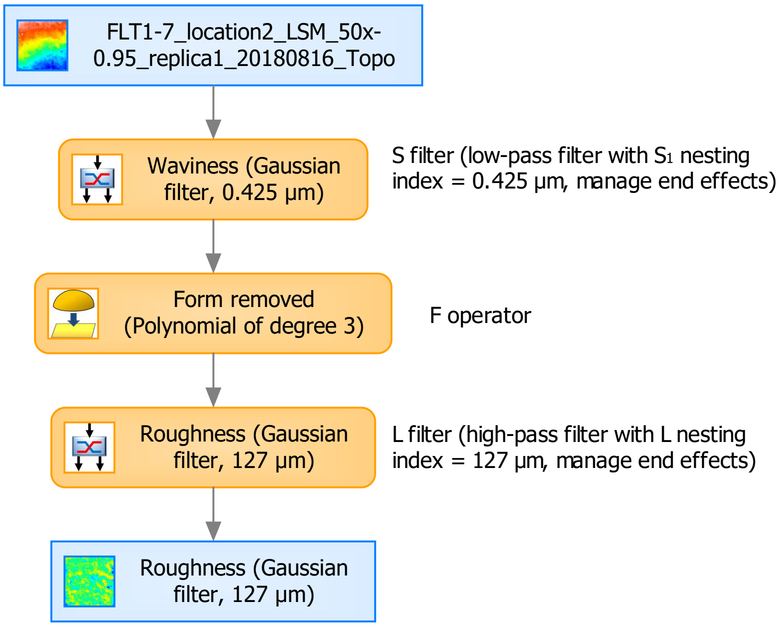

Flint FLT1-7
50x objective, NA = 0.75 or 0.95

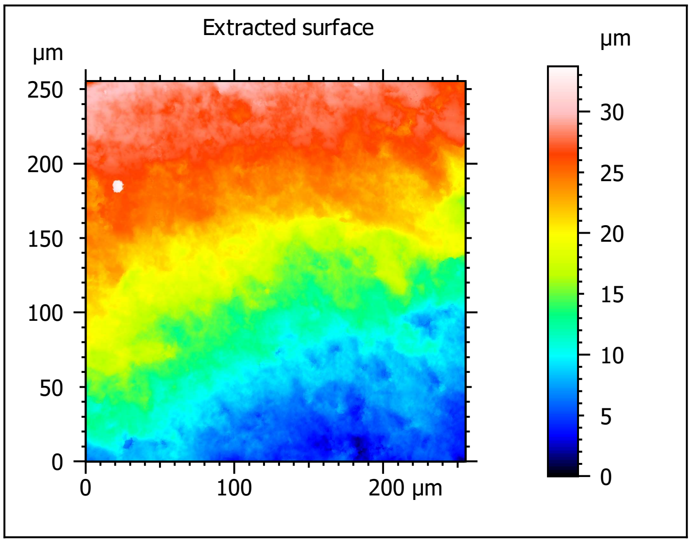

Identity card - FLT1-7_location2_LSM_50x-...		
Name:	FLT1-7_location2_LSM_50x-0.95_replica2_2018...	
Created on:	8/16/2018 3:49:58 PM	
**Axis:**	**X**	
Length:	255	µm
Size:	3000	points
Spacing:	85.2	nm
**Axis:**	**Y**	
Length:	255	µm
Size:	3000	points
Spacing:	85.2	nm
**Axis:**	**Z**	
Length:	33.7	µm
Size:	65532	digits
Spacing:	0.514	nm
NMP ratio:	0.00 % (0 Pts)	

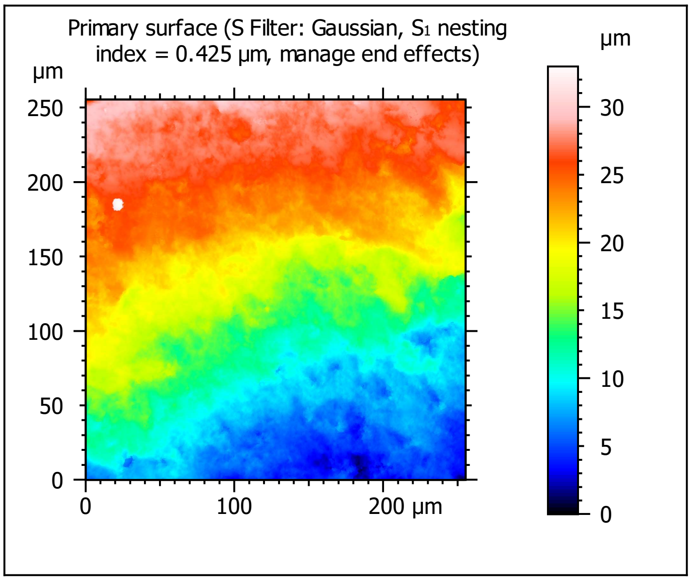

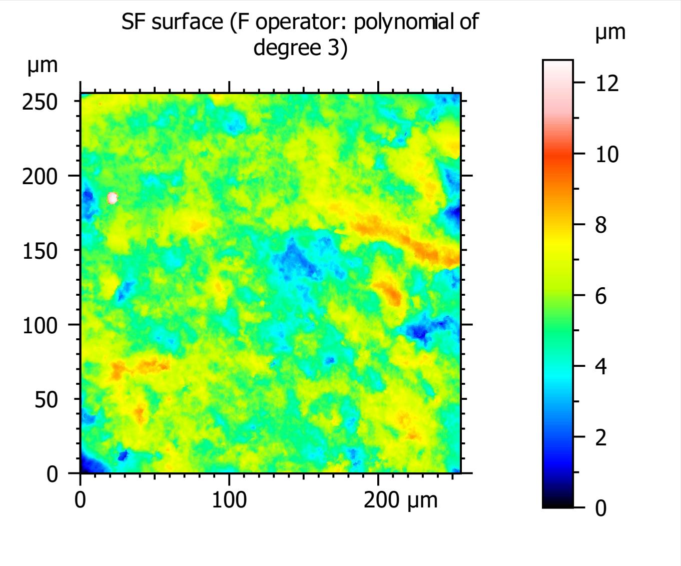

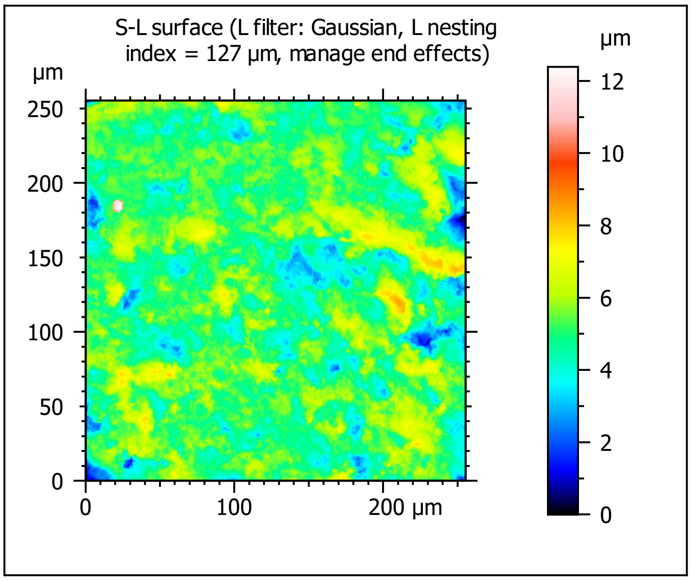

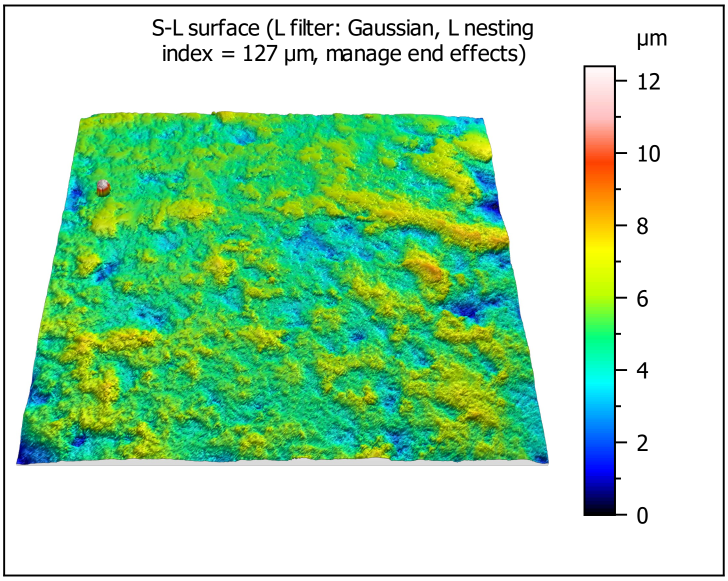

ISO 25178		
**Height Parameters**		
Sq	0.931	µm
Ssk	-0.0815	
Sku	5.73	
Sp	7.31	µm
Sv	5.09	µm
Sz	12.4	µm
Sa	0.689	µm
**Functional Parameters**		
Smr	0.046	%
Smc	1.08	µm
Sxp	2.04	µm
**Spatial Parameters**		
Sal	11.5	µm
Str	0.782	
Std	130	°
**Hybrid Parameters**		
Sdq	0.852	
Sdr	22.1	%
**Functional Parameters (V...**		
Vm	0.0543	µm³/µm²
Vv	1.13	µm³/µm²
Vmp	0.0543	µm³/µm²
Vmc	0.728	µm³/µm²
Vvc	1.00	µm³/µm²
Vvv	0.132	µm³/µm²
**Functional Parameters (S...**		
Sk	1.72	µm
Spk	0.965	µm
Svk	1.03	µm
Smr1	10.5	%
Smr2	87.3	%
Spq	*****	
Svq	*****	
Smq	*****	

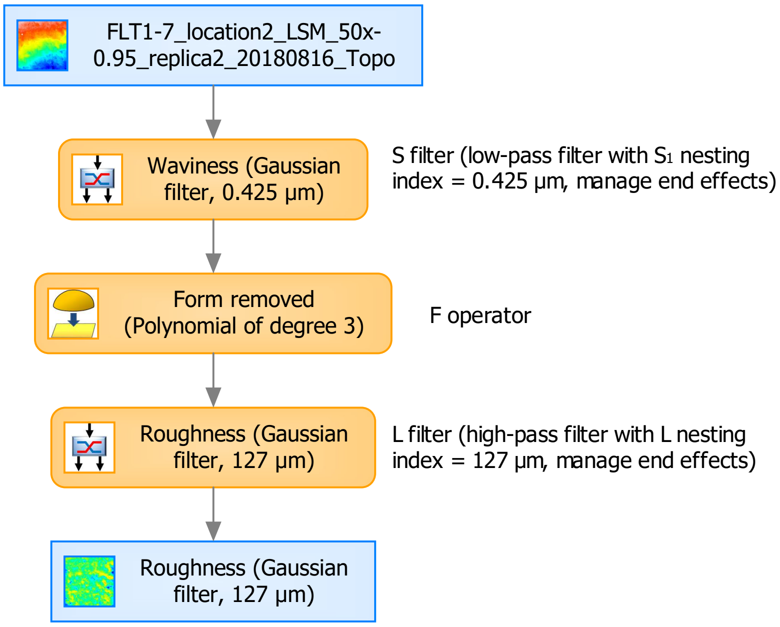

Flint FLT1-7
50x objective, NA = 0.75 or 0.95

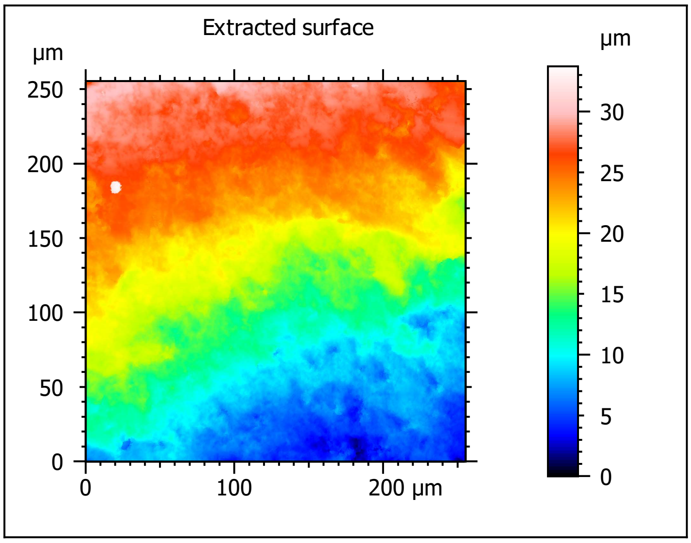

**Identity card - FLT1-7_location2_LSM_50x-...**		
Name:	FLT1-7_location2_LSM_50x-0.95_replica3_2018...	
Created on:	8/16/2018 4:17:09 PM	
**Axis:**	**X**	
Length:	255	µm
Size:	3000	points
Spacing:	85.2	nm
**Axis:**	**Y**	
Length:	255	µm
Size:	3000	points
Spacing:	85.2	nm
**Axis:**	**Z**	
Length:	33.7	µm
Size:	65532	digits
Spacing:	0.515	nm
NMP ratio:	0.00 % (0 Pts)	

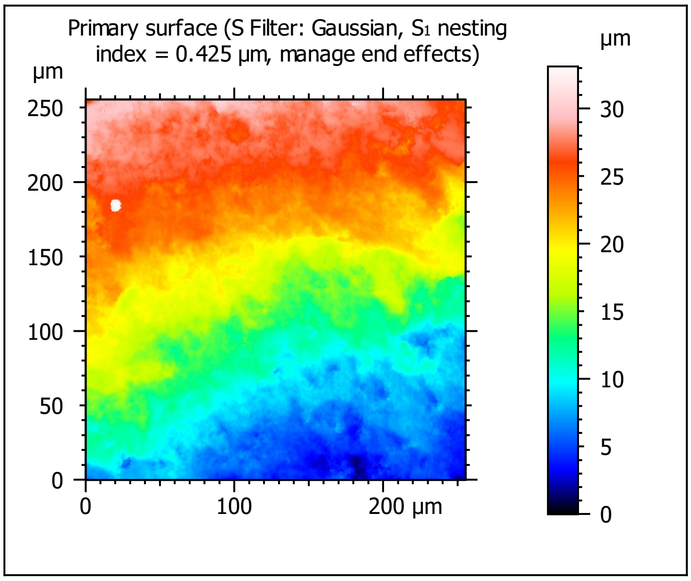

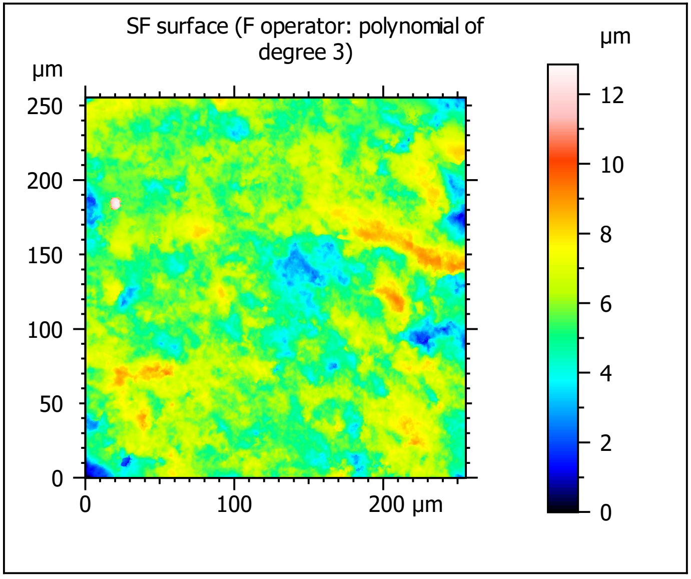

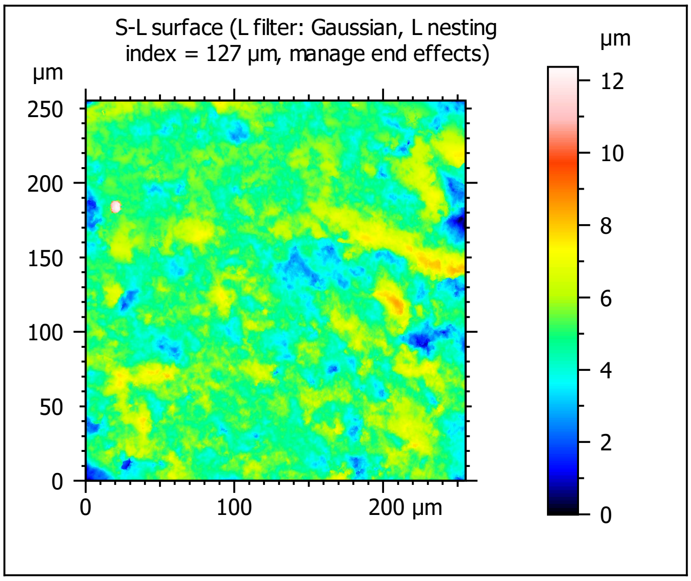

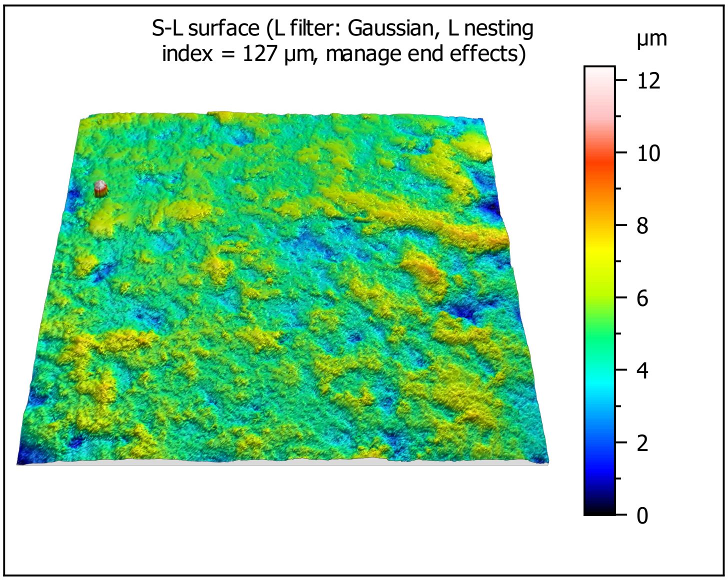

ISO 25178		
**Height Parameters**		
Sq	0.937	µm
Ssk	-0.0634	
Sku	5.76	
Sp	7.37	µm
Sv	5.01	µm
Sz	12.4	µm
Sa	0.693	µm
**Functional Parameters**		
Smr	0.0458	%
Smc	1.09	µm
Sxp	2.04	µm
**Spatial Parameters**		
Sal	11.5	µm
Str	0.773	
Std	130	°
**Hybrid Parameters**		
Sdq	0.860	
Sdr	22.3	%
**Functional Parameters (V...**		
Vm	0.0554	µm³/µm²
Vv	1.14	µm³/µm²
Vmp	0.0554	µm³/µm²
Vmc	0.730	µm³/µm²
Vvc	1.01	µm³/µm²
Vvv	0.132	µm³/µm²
**Functional Parameters (S...**		
Sk	1.73	µm
Spk	0.980	µm
Svk	1.04	µm
Smr1	10.4	%
Smr2	87.5	%
Spq	*****	
Svq	*****	
Smq	*****	

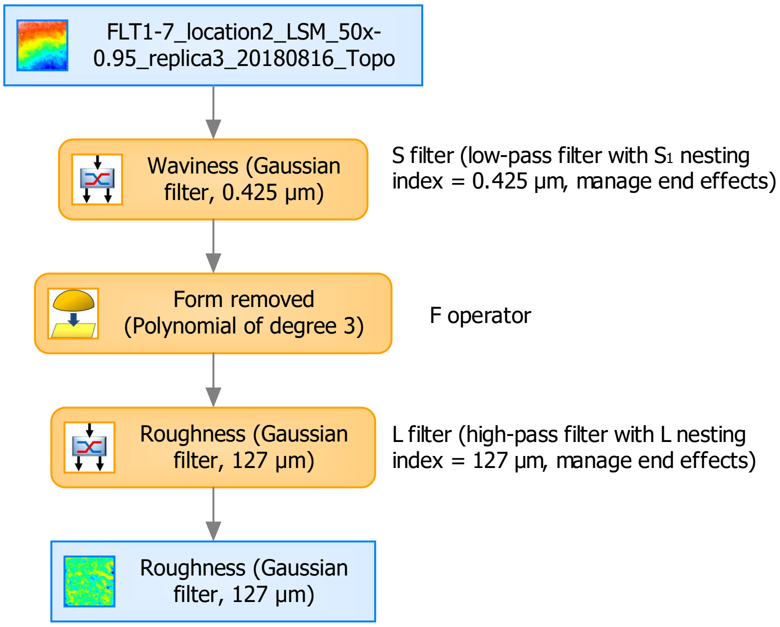

Flint FLT1-7
50x objective, NA = 0.75 or 0.95

**Identity card - FLT1-7_location3_LSM_50x-...**		
Name:	FLT1-7_location3_LSM_50x-0.75_replica1_2018...	
Created on:	8/20/2018 11:31:12 AM	
**Axis:**	**X**	
Length:	255	µm
Size:	3000	points
Spacing:	85.2	nm
**Axis:**	**Y**	
Length:	255	µm
Size:	3000	points
Spacing:	85.2	nm
**Axis:**	**Z**	
Length:	15.9	µm
Size:	65532	digits
Spacing:	0.243	nm
NMP ratio:	0.00 % (0 Pts)	

Extracted surface

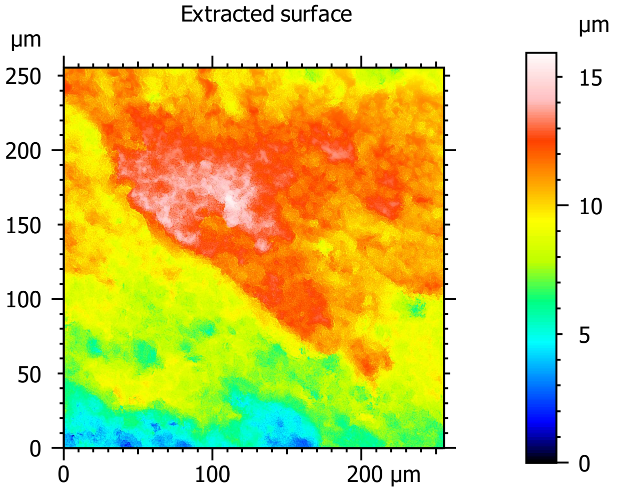

Primary surface (S Filter: Gaussian, S1 nesting index = 0.425 µm, manage end effects)

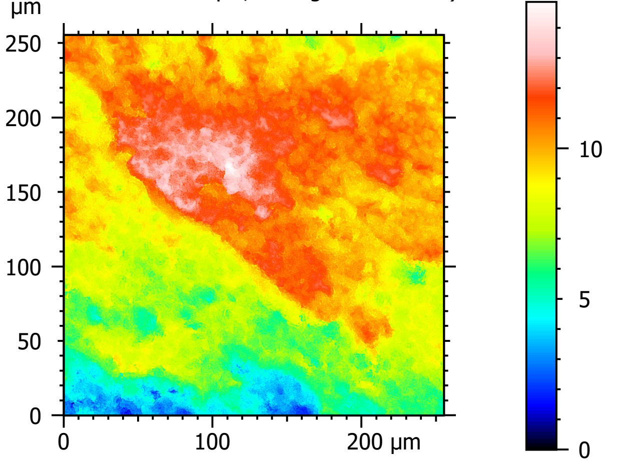

SF surface (F operator: polynomial of degree 3)

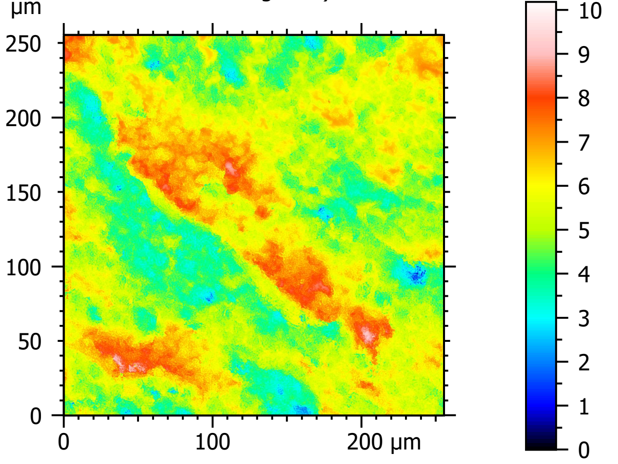

S-L surface (L filter: Gaussian, L nesting index = 127 µm, manage end effects)

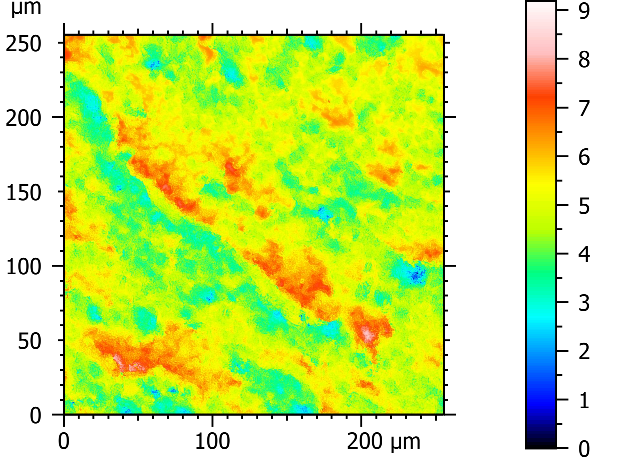

S-L surface (L filter: Gaussian, L nesting index = 127 µm, manage end effects)

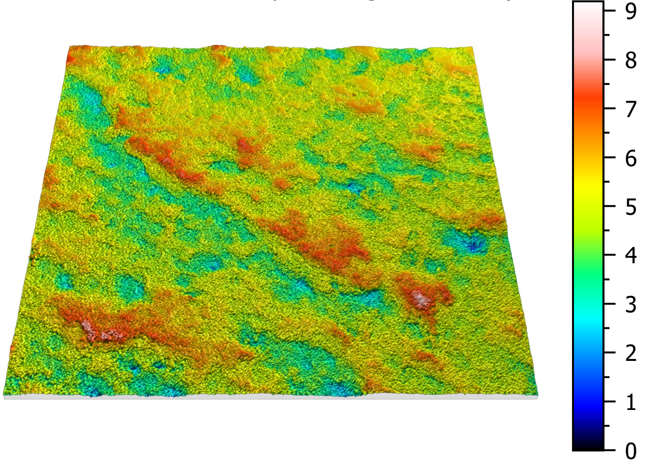

ConfoMap ST 7.4.8633

ISO 25178		
**Height Parameters**		
Sq	0.855	µm
Ssk	0.123	
Sku	3.40	
Sp	4.32	µm
Sv	4.87	µm
Sz	9.19	µm
Sa	0.667	µm
**Functional Parameters**		
Smr	0.0111	%
Smc	1.10	µm
Sxp	1.62	µm
**Spatial Parameters**		
Sal	11.8	µm
Str	0.403	
Std	141	°
**Hybrid Parameters**		
Sdq	1.64	
Sdr	72.3	%
**Functional Parameters (V...**		
Vm	0.0469	µm³/µm²
Vv	1.15	µm³/µm²
Vmp	0.0469	µm³/µm²
Vmc	0.734	µm³/µm²
Vvc	1.05	µm³/µm²
Vvv	0.096	µm³/µm²
**Functional Parameters (S...**		
Sk	1.62	µm
Spk	0.781	µm
Svk	0.808	µm
Smr1	11.1	%
Smr2	89.0	%
Spq	*****	
Svq	*****	
Smq	*****	

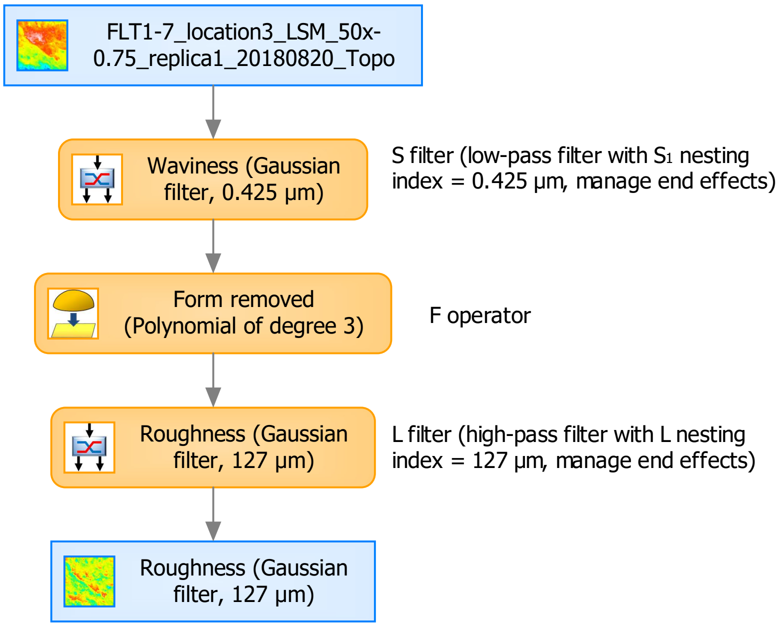

Flint FLT1-7
50x objective, NA = 0.75 or 0.95

**Identity card - FLT1-7_location3_LSM_50x-...**		
Name:	FLT1-7_location3_LSM_50x-0.75_replica2_2018...	
Created on:	8/20/2018 11:50:58 AM	
**Axis:**	**X**	
Length:	255	µm
Size:	3000	points
Spacing:	85.2	nm
**Axis:**	**Y**	
Length:	255	µm
Size:	3000	points
Spacing:	85.2	nm
**Axis:**	**Z**	
Length:	16.1	µm
Size:	65532	digits
Spacing:	0.246	nm
NMP ratio:	0.00 % (0 Pts)	

Extracted surface

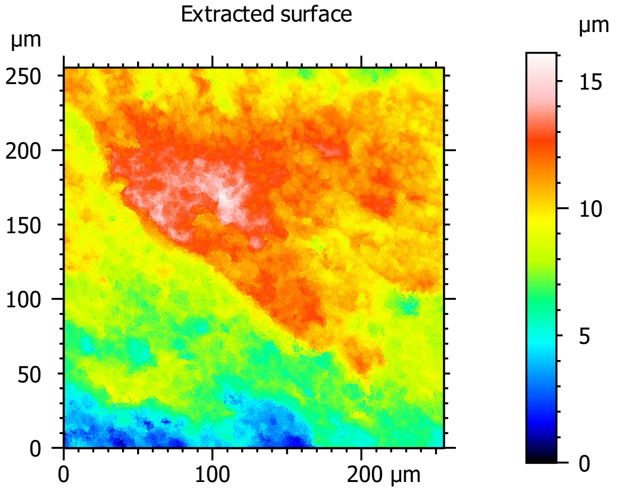

Primary surface (S Filter: Gaussian, S1 nesting index = 0.425 µm, manage end effects)

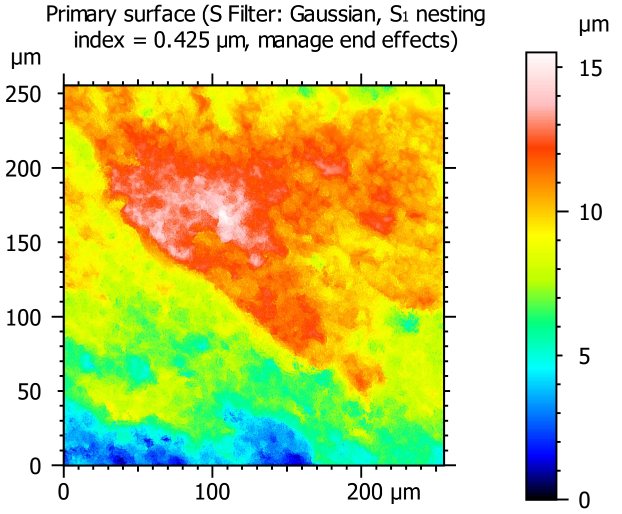

SF surface (F operator: polynomial of degree 3)

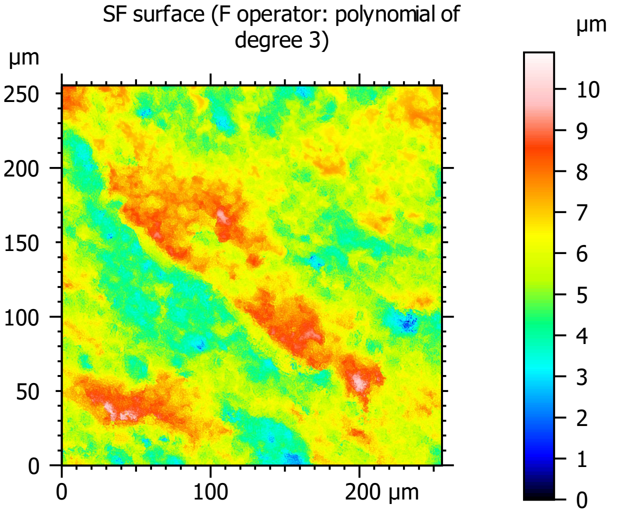

S-L surface (L filter: Gaussian, L nesting index = 127 µm, manage end effects)

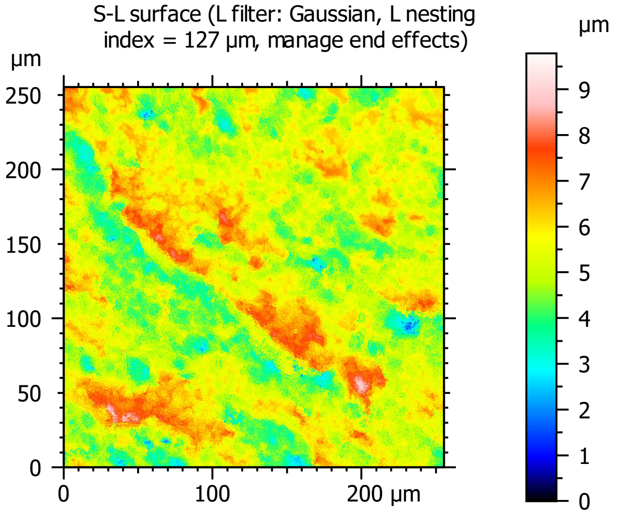

S-L surface (L filter: Gaussian, L nesting index = 127 µm, manage end effects)

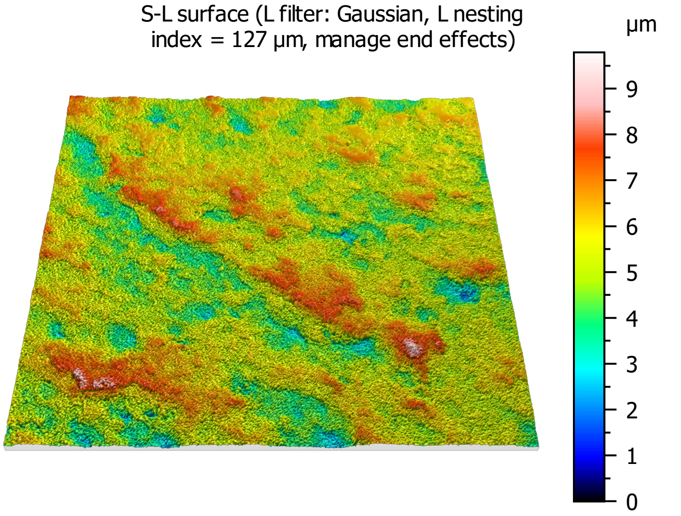

ISO 25178		
**Height Parameters**		
Sq	0.932	µm
Ssk	0.112	
Sku	3.42	
Sp	4.47	µm
Sv	5.32	µm
Sz	9.79	µm
Sa	0.727	µm
**Functional Parameters**		
Smr	0.0339	%
Smc	1.19	µm
Sxp	1.78	µm
**Spatial Parameters**		
Sal	11.9	µm
Str	0.388	
Std	141	°
**Hybrid Parameters**		
Sdq	1.78	
Sdr	81.6	%
**Functional Parameters (V...**		
Vm	0.0516	µm³/µm²
Vv	1.25	µm³/µm²
Vmp	0.0516	µm³/µm²
Vmc	0.800	µm³/µm²
Vvc	1.14	µm³/µm²
Vvv	0.106	µm³/µm²
**Functional Parameters (S...**		
Sk	1.76	µm
Spk	0.852	µm
Svk	0.884	µm
Smr1	10.9	%
Smr2	88.9	%
Spq	*****	
Svq	*****	
Smq	*****	

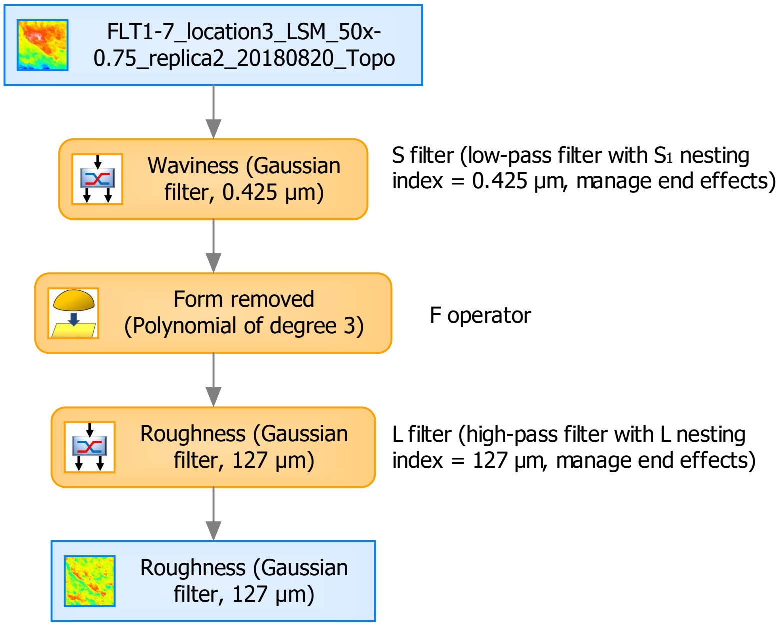

Flint FLT1-7
50x objective, NA = 0.75 or 0.95

Extracted surface

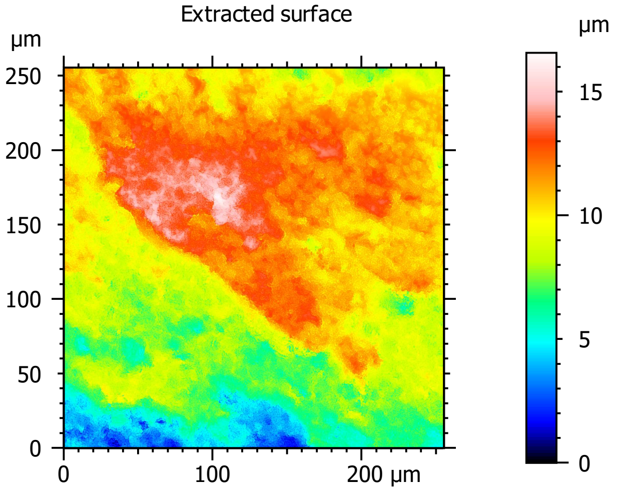

Identity card - FLT1-7_location3_LSM_50x-...		
Name:	FLT1-7_location3_LSM_50x-0.75_replica3_2018...	
Created on:	8/20/2018 12:05:22 PM	
**Axis:**	**X**	
Length:	255	µm
Size:	3000	points
Spacing:	85.2	nm
**Axis:**	**Y**	
Length:	255	µm
Size:	3000	points
Spacing:	85.2	nm
**Axis:**	**Z**	
Length:	16.6	µm
Size:	65532	digits
Spacing:	0.253	nm
NMP ratio:	0.00 % (0 Pts)	

Primary surface (S Filter: Gaussian, S_1 nesting index = 0.425 µm, manage end effects)

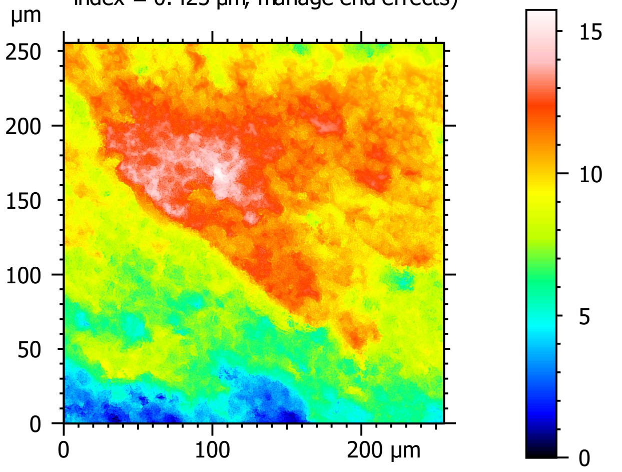

SF surface (F operator: polynomial of degree 3)

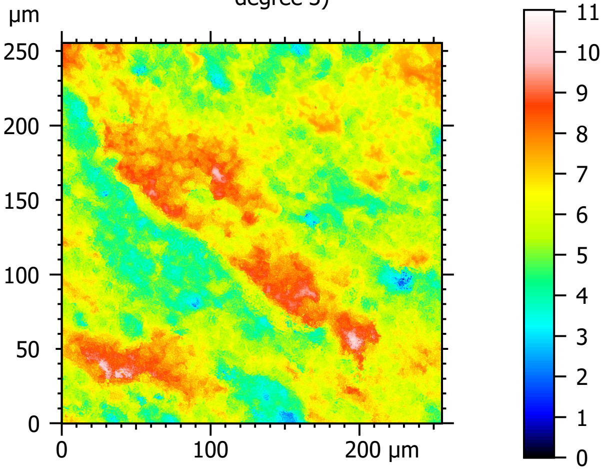

S-L surface (L filter: Gaussian, L nesting index = 127 µm, manage end effects)

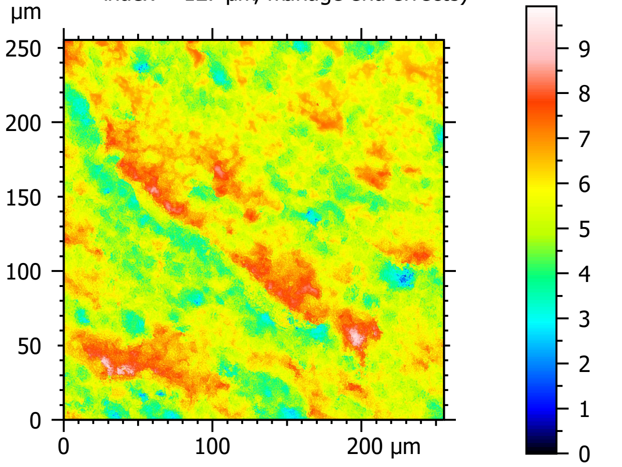

S-L surface (L filter: Gaussian, L nesting index = 127 µm, manage end effects)

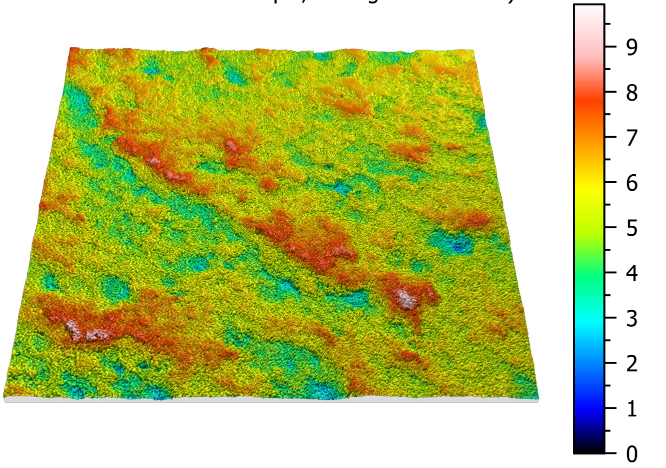

ISO 25178		
**Height Parameters**		
Sq	0.955	µm
Ssk	0.108	
Sku	3.42	
Sp	4.41	µm
Sv	5.53	µm
Sz	9.93	µm
Sa	0.745	µm
**Functional Parameters**		
Smr	0.0677	%
Smc	1.22	µm
Sxp	1.83	µm
**Spatial Parameters**		
Sal	11.9	µm
Str	0.380	
Std	141	°
**Hybrid Parameters**		
Sdq	1.82	
Sdr	84.1	%
**Functional Parameters (V...**		
Vm	0.053	µm³/µm²
Vv	1.27	µm³/µm²
Vmp	0.053	µm³/µm²
Vmc	0.821	µm³/µm²
Vvc	1.16	µm³/µm²
Vvv	0.108	µm³/µm²
**Functional Parameters (S...**		
Sk	1.79	µm
Spk	0.868	µm
Svk	0.909	µm
Smr1	11.0	%
Smr2	88.7	%
Spq	*****	
Svq	*****	
Smq	*****	

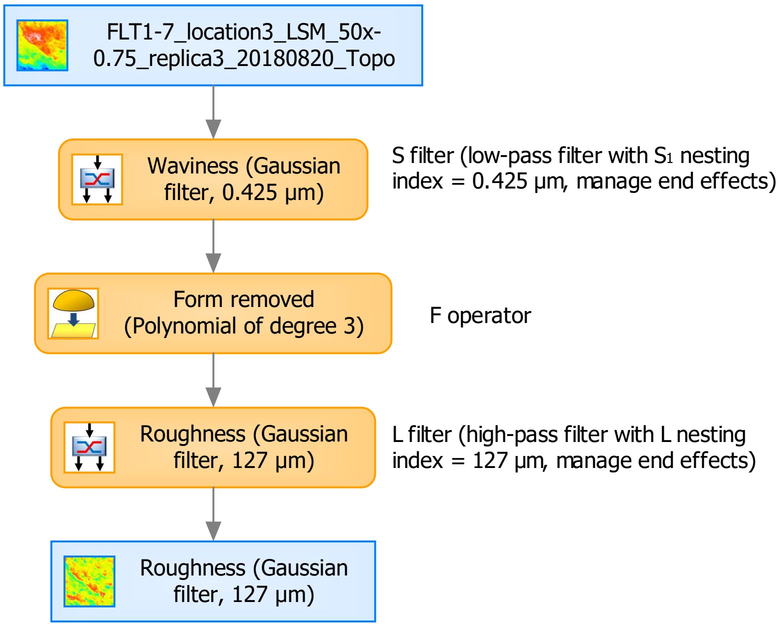

Flint FLT1-7
50x objective, NA = 0.75 or 0.95

Extracted surface

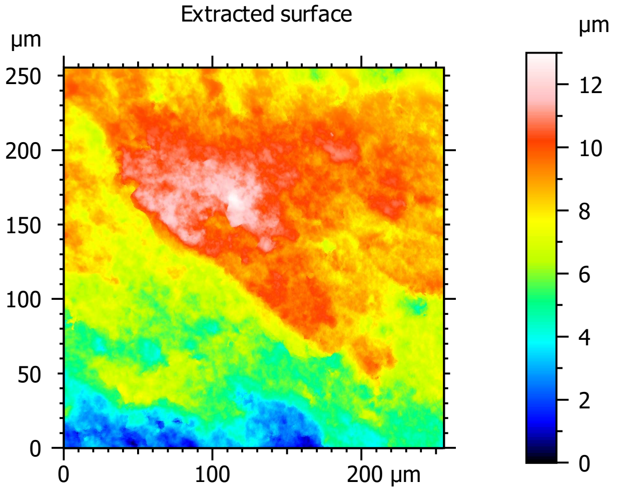

Identity card - FLT1-7_location3_LSM_50x-...		
Name:	FLT1-7_location3_LSM_50x-0.95_replica1_2018...	
Created on:	8/20/2018 11:23:03 AM	
**Axis:**	**X**	
Length:	255	µm
Size:	3000	points
Spacing:	85.2	nm
**Axis:**	**Y**	
Length:	255	µm
Size:	3000	points
Spacing:	85.2	nm
**Axis:**	**Z**	
Length:	13.0	µm
Size:	65532	digits
Spacing:	0.198	nm
NMP ratio:	0.00 % (0 Pts)	

Primary surface (S Filter: Gaussian, S1 nesting index = 0.425 µm, manage end effects)

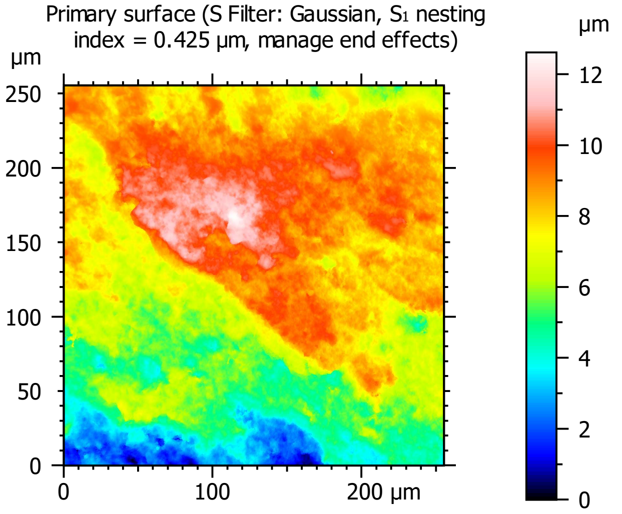

SF surface (F operator: polynomial of degree 3)

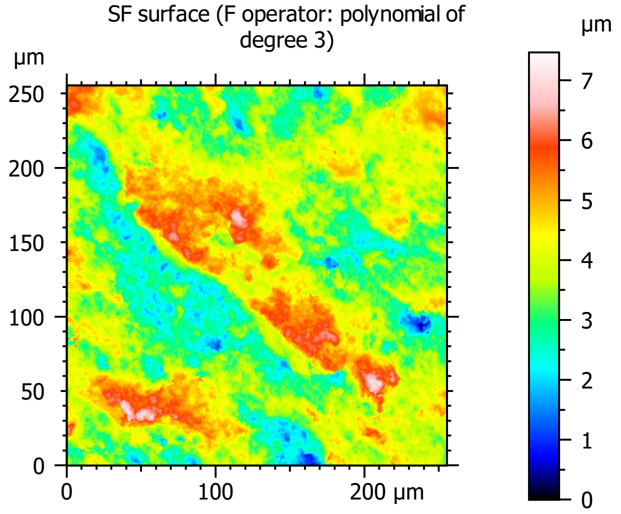

S-L surface (L filter: Gaussian, L nesting index = 127 µm, manage end effects)

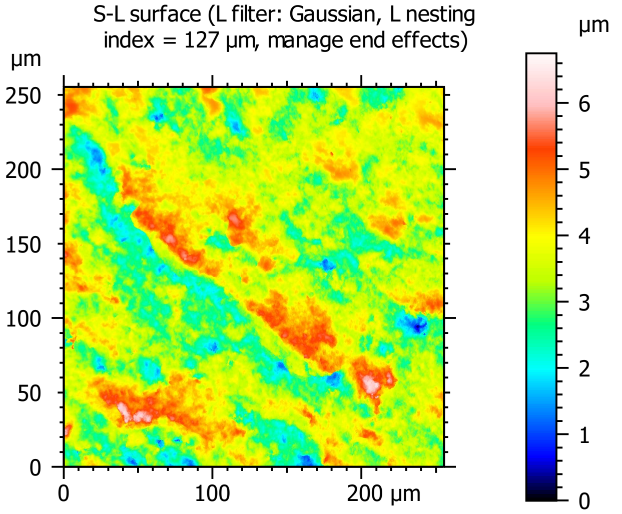

S-L surface (L filter: Gaussian, L nesting index = 127 µm, manage end effects)

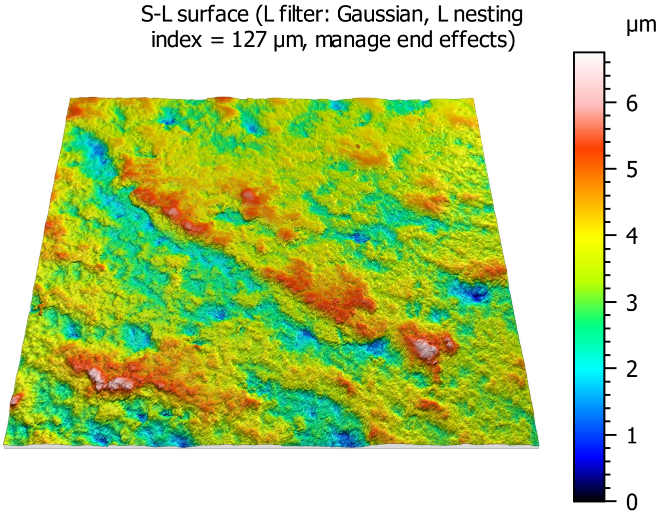

ISO 25178		
**Height Parameters**		
Sq	0.740	µm
Ssk	0.175	
Sku	3.38	
Sp	3.26	µm
Sv	3.50	µm
Sz	6.75	µm
Sa	0.576	µm
**Functional Parameters**		
Smr	0.306	%
Smc	0.958	µm
Sxp	1.38	µm
**Spatial Parameters**		
Sal	12.4	µm
Str	0.392	
Std	141	°
**Hybrid Parameters**		
Sdq	0.611	
Sdr	13.5	%
**Functional Parameters (V...**		
Vm	0.0419	$\mu m^3/\mu m^2$
Vv	1.00	$\mu m^3/\mu m^2$
Vmp	0.0419	$\mu m^3/\mu m^2$
Vmc	0.634	$\mu m^3/\mu m^2$
Vvc	0.918	$\mu m^3/\mu m^2$
Vvv	0.0819	$\mu m^3/\mu m^2$
**Functional Parameters (S...**		
Sk	1.33	µm
Spk	0.690	µm
Svk	0.679	µm
Smr1	11.8	%
Smr2	88.9	%
Spq	*****	
Svq	*****	
Smq	*****	

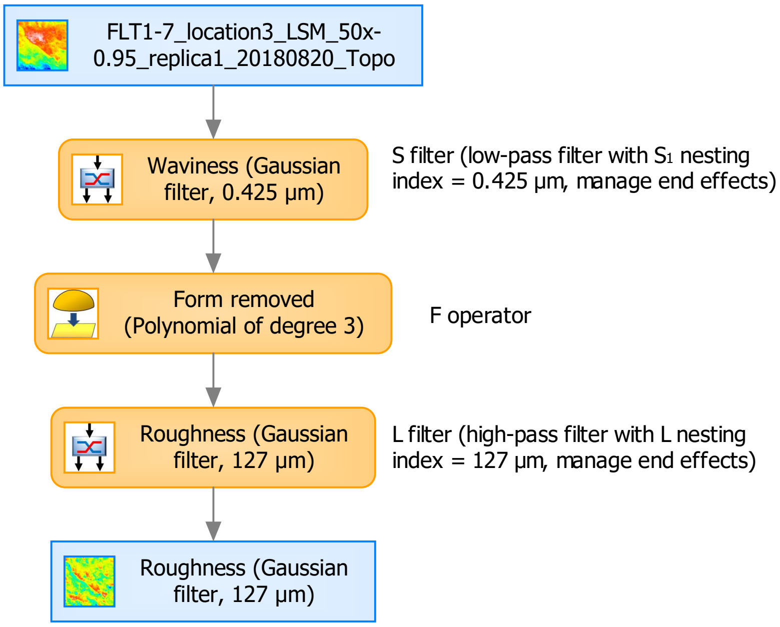

Flint FLT1-7
50x objective, NA = 0.75 or 0.95

Identity card - FLT1-7_location3_LSM_50x-...		
Name:	FLT1-7_location3_LSM_50x-0.95_replica2_2018...	
Created on:	8/20/2018 11:40:53 AM	
**Axis:**	**X**	
Length:	255	µm
Size:	3000	points
Spacing:	85.2	nm
**Axis:**	**Y**	
Length:	255	µm
Size:	3000	points
Spacing:	85.2	nm
**Axis:**	**Z**	
Length:	14.8	µm
Size:	65532	digits
Spacing:	0.227	nm
NMP ratio:	0.00 % (0 Pts)	

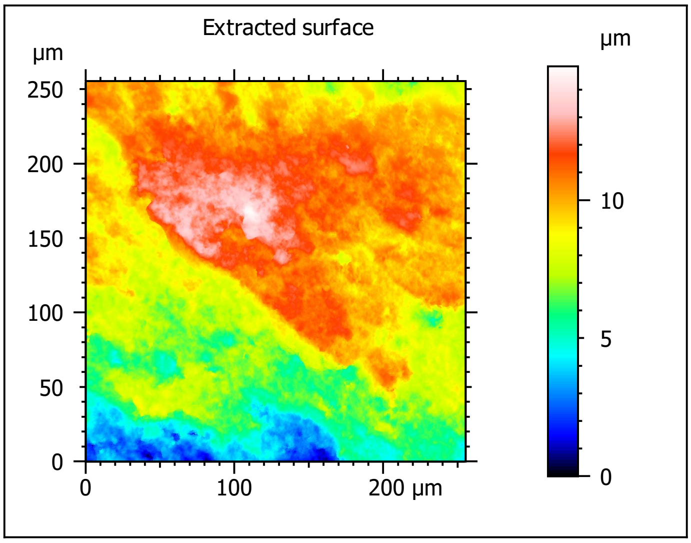

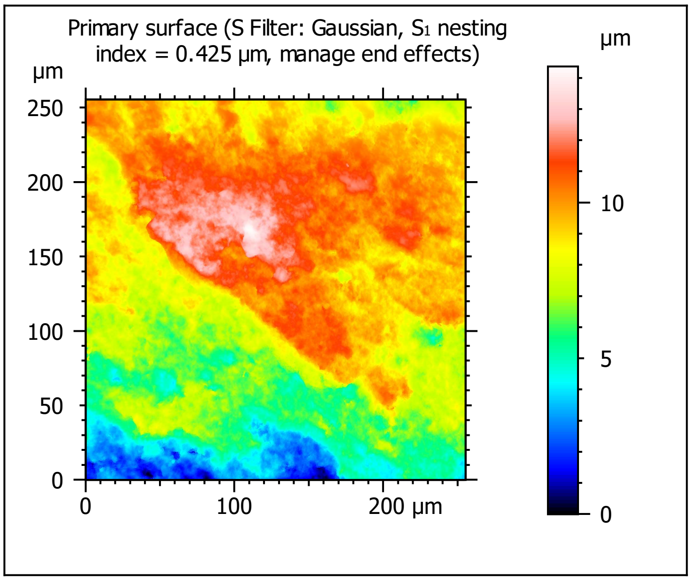

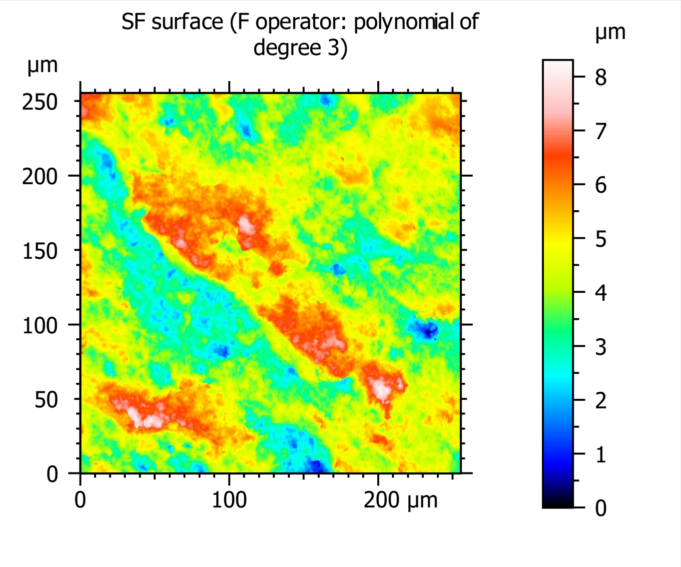

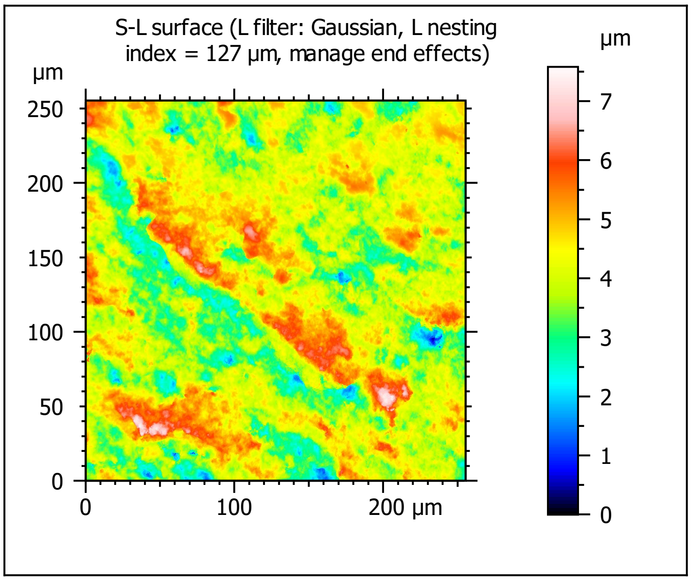

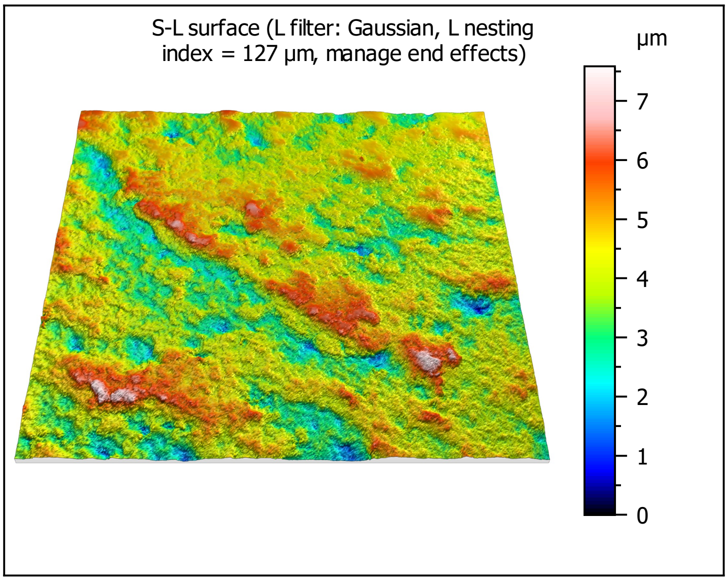

ISO 25178		
**Height Parameters**		
Sq	0.844	µm
Ssk	0.170	
Sku	3.40	
Sp	3.50	µm
Sv	4.09	µm
Sz	7.58	µm
Sa	0.657	µm
**Functional Parameters**		
Smr	0.363	%
Smc	1.09	µm
Sxp	1.58	µm
**Spatial Parameters**		
Sal	12.4	µm
Str	0.384	
Std	141	°
**Hybrid Parameters**		
Sdq	0.705	
Sdr	17.2	%
**Functional Parameters (V...**		
Vm	0.0481	µm^3/µm^2
Vv	1.14	µm^3/µm^2
Vmp	0.0481	µm^3/µm^2
Vmc	0.723	µm^3/µm^2
Vvc	1.04	µm^3/µm^2
Vvv	0.0939	µm^3/µm^2
**Functional Parameters (S...**		
Sk	1.52	µm
Spk	0.783	µm
Svk	0.773	µm
Smr1	11.5	%
Smr2	88.9	%
Spq	*****	
Svq	*****	
Smq	*****	

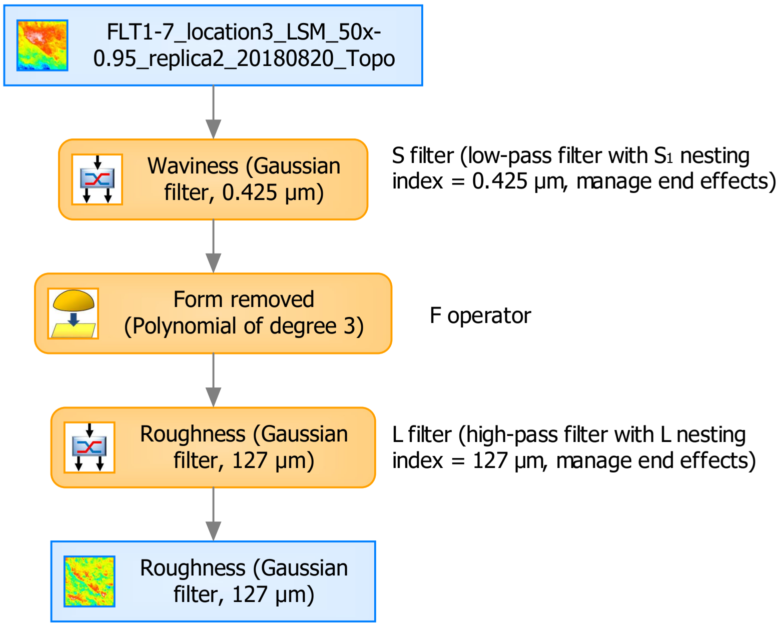

Flint FLT1-7
50x objective, NA = 0.75 or 0.95

Identity card - FLT1-7_location3_LSM_50x-...		
Name:	FLT1-7_location3_LSM_50x-0.95_replica3_2018...	
Created on:	8/20/2018 11:58:10 AM	
**Axis:**	**X**	
Length:	255	µm
Size:	3000	points
Spacing:	85.2	nm
**Axis:**	**Y**	
Length:	255	µm
Size:	3000	points
Spacing:	85.2	nm
**Axis:**	**Z**	
Length:	15.9	µm
Size:	65532	digits
Spacing:	0.243	nm
NMP ratio:	0.00 % (0 Pts)	

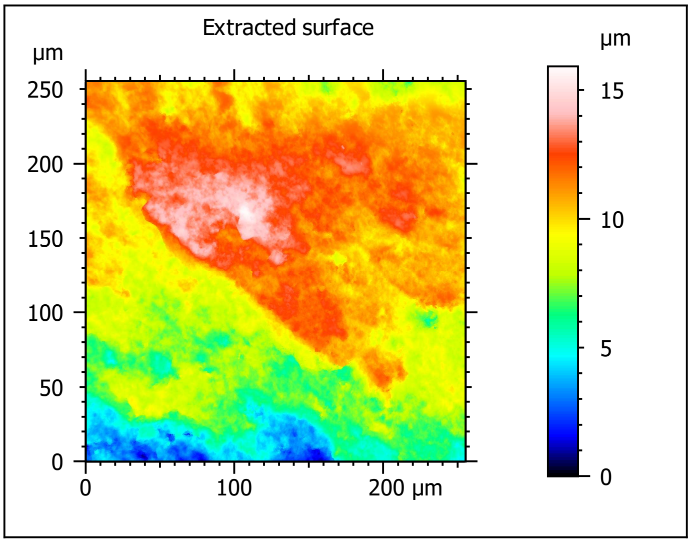

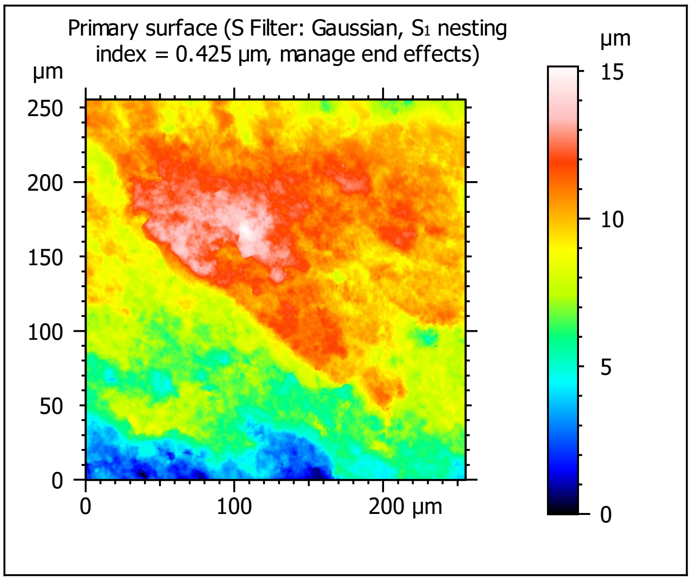

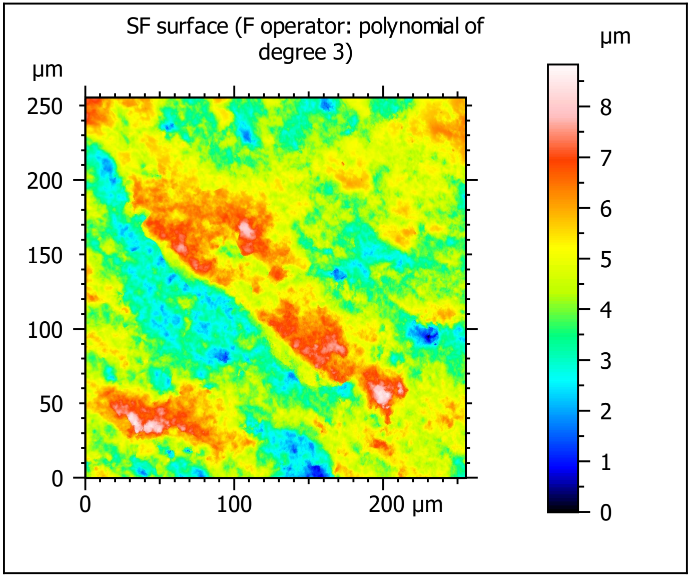

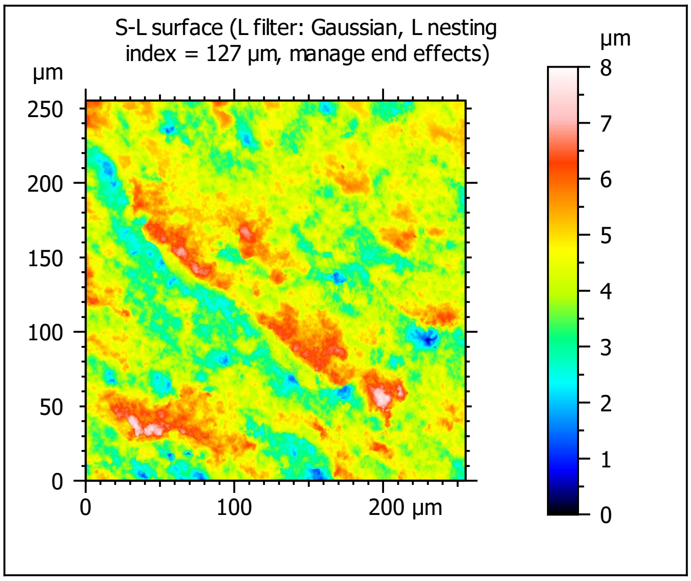

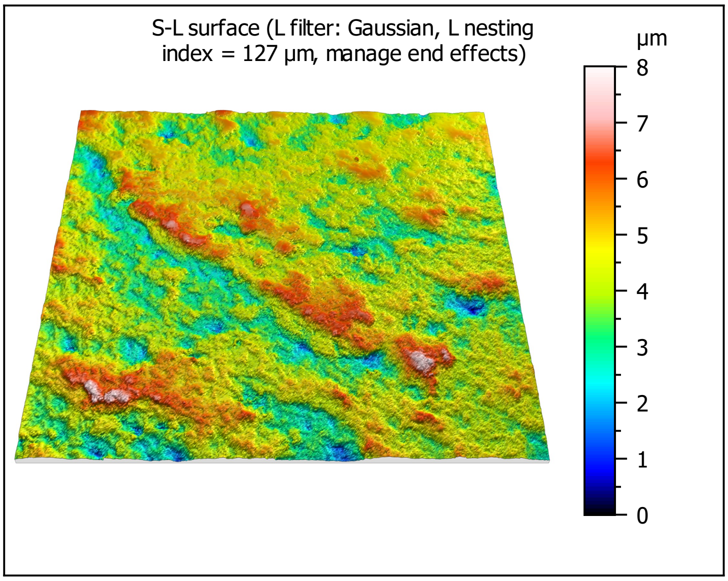

ISO 25178		
**Height Parameters**		
Sq	0.887	µm
Ssk	0.165	
Sku	3.40	
Sp	3.77	µm
Sv	4.23	µm
Sz	8.00	µm
Sa	0.691	µm
**Functional Parameters**		
Smr	0.268	%
Smc	1.14	µm
Sxp	1.66	µm
**Spatial Parameters**		
Sal	12.5	µm
Str	0.380	
Std	141	°
**Hybrid Parameters**		
Sdq	0.735	
Sdr	18.6	%
**Functional Parameters (V...**		
Vm	0.0507	µm³/µm²
Vv	1.19	µm³/µm²
Vmp	0.0507	µm³/µm²
Vmc	0.760	µm³/µm²
Vvc	1.09	µm³/µm²
Vvv	0.0992	µm³/µm²
**Functional Parameters (S...**		
Sk	1.60	µm
Spk	0.825	µm
Svk	0.809	µm
Smr1	11.5	%
Smr2	88.8	%
Spq	*****	
Svq	*****	
Smq	*****	

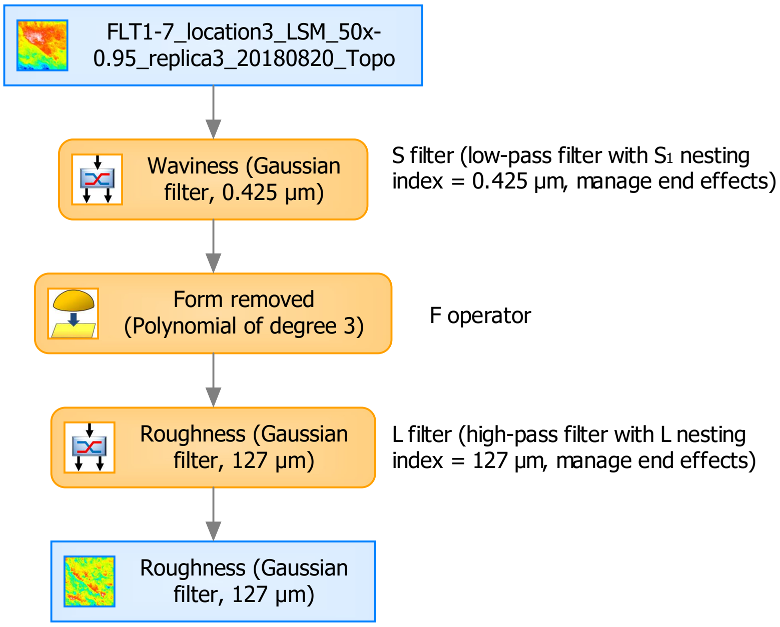

Quartzite QTFU2-10
50x objective, NA = 0.75 or 0.95

Extracted surface

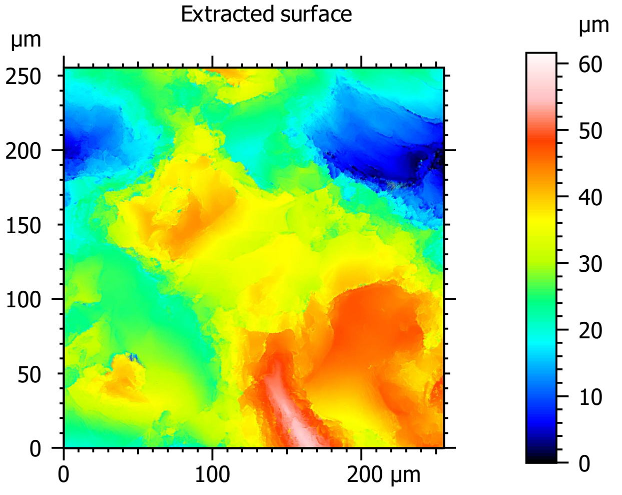

Identity card - QTFU2-10_location1_LSM_5...		
Name:	QTFU2-10_location1_LSM_50x-0.75_replica1_20...	
Created on:	8/16/2018 10:53:03 AM	
**Axis:**	**X**	
Length:	255	µm
Size:	3000	points
Spacing:	85.2	nm
**Axis:**	**Y**	
Length:	255	µm
Size:	3000	points
Spacing:	85.2	nm
**Axis:**	**Z**	
Length:	61.6	µm
Size:	65532	digits
Spacing:	0.940	nm
NMP ratio:	0.00 % (0 Pts)	

Primary surface (S Filter: Gaussian, S_1 nesting index = 0.425 µm, manage end effects)

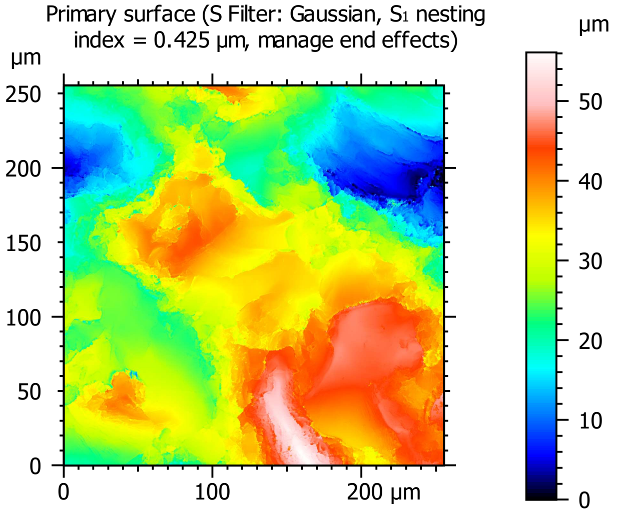

SF surface (F operator: polynomial of degree 3)

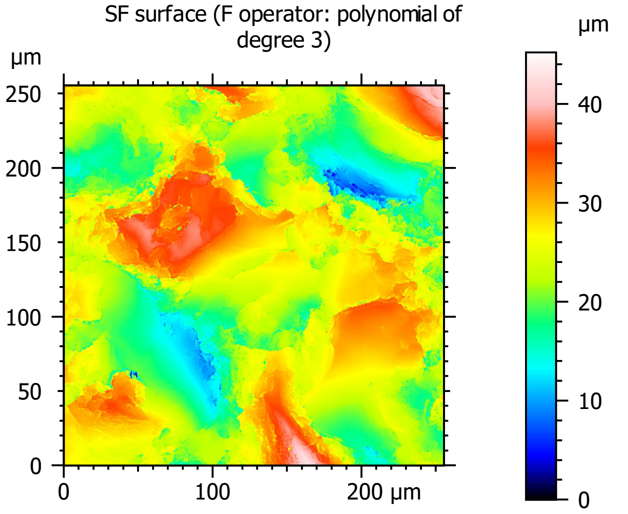

S-L surface (L filter: Gaussian, L nesting index = 127 µm, manage end effects)

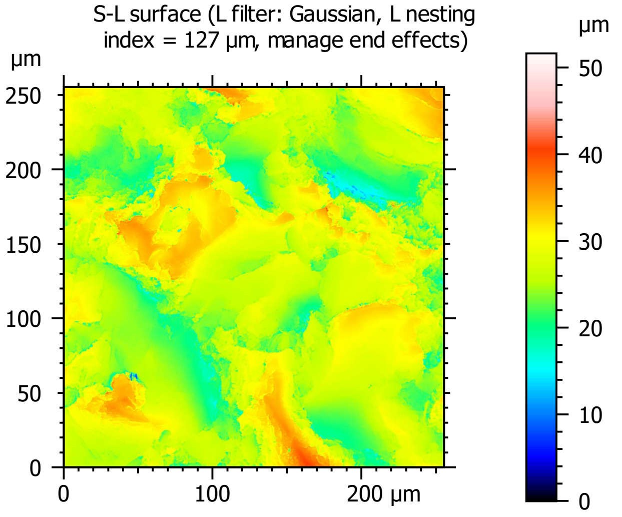

S-L surface (L filter: Gaussian, L nesting index = 127 µm, manage end effects)

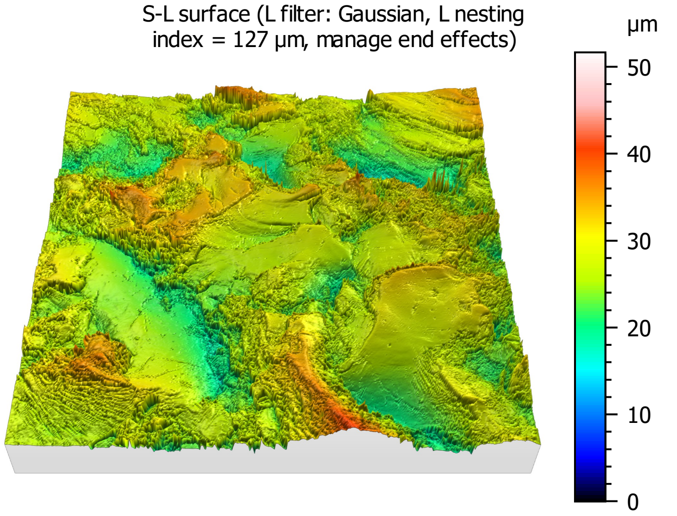

ISO 25178		
**Height Parameters**		
Sq	3.79	µm
Ssk	-0.0178	
Sku	3.12	
Sp	24.9	µm
Sv	26.8	µm
Sz	51.7	µm
Sa	3.00	µm
**Functional Parameters**		
Smr	1.11e-05	%
Smc	4.97	µm
Sxp	7.29	µm
**Spatial Parameters**		
Sal	20.0	µm
Str	0.630	
Std	19.0	°
**Hybrid Parameters**		
Sdq	4.82	
Sdr	148	%
**Functional Parameters (Vol...**		
Vm	0.174	µm³/µm²
Vv	5.14	µm³/µm²
Vmp	0.174	µm³/µm²
Vmc	3.35	µm³/µm²
Vvc	4.71	µm³/µm²
Vvv	0.440	µm³/µm²
**Functional Parameters (Str...**		
Sk	5.45	µm
Spk	2.78	µm
Svk	3.47	µm
Smr1	12.0	%
Smr2	87.1	%
Spq	*****	
Svq	*****	
Smq	*****	

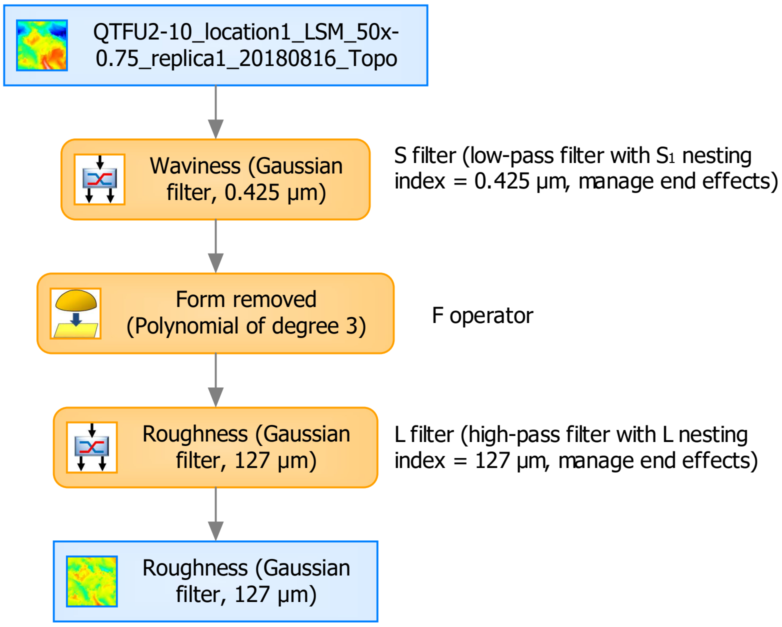

Quartzite QTFU2-10
50x objective, NA = 0.75 or 0.95

Extracted surface

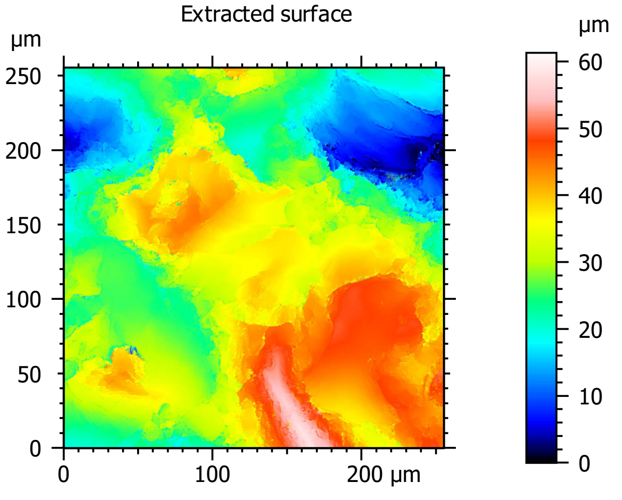

Identity card - QTFU2-10_location1_LSM_5...			
Name:	QTFU2-10_location1_LSM_50x-0.75_replica2_20...		
Created on:	8/16/2018 11:34:47 AM		
**Axis:**	**X**		
Length:	255	µm	
Size:	3000	points	
Spacing:	85.2	nm	
**Axis:**	**Y**		
Length:	255	µm	
Size:	3000	points	
Spacing:	85.2	nm	
**Axis:**	**Z**		
Length:	61.3	µm	
Size:	65532	digits	
Spacing:	0.935	nm	
NMP ratio:	0.00 % (0 Pts)		

Primary surface (S Filter: Gaussian, S_1 nesting index = 0.425 µm, manage end effects)

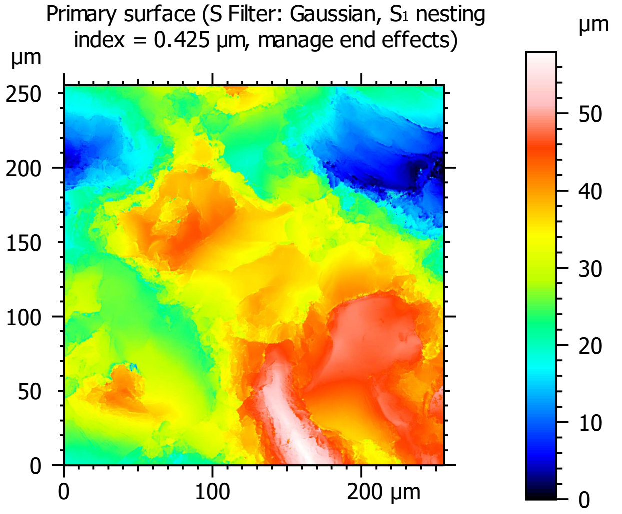

SF surface (F operator: polynomial of degree 3)

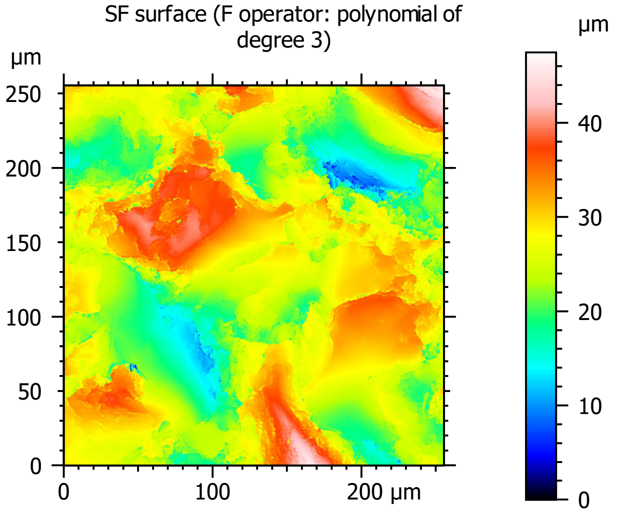

S-L surface (L filter: Gaussian, L nesting index = 127 µm, manage end effects)

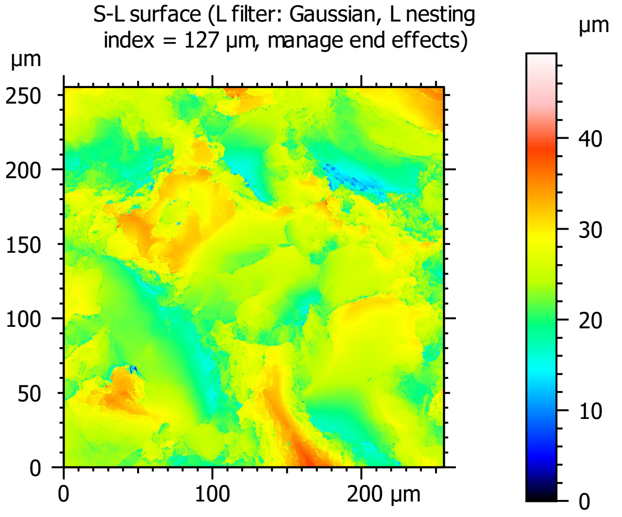

S-L surface (L filter: Gaussian, L nesting index = 127 µm, manage end effects)

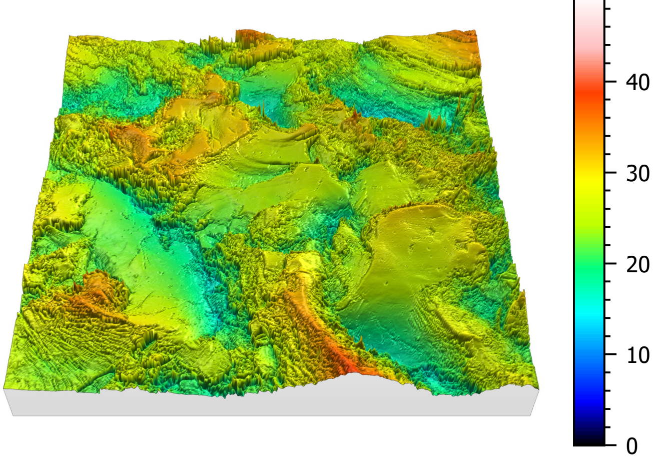

ISO 25178		
**Height Parameters**		
Sq	3.99	µm
Ssk	-0.000955	
Sku	3.11	
Sp	24.8	µm
Sv	24.6	µm
Sz	49.4	µm
Sa	3.16	µm
**Functional Parameters**		
Smr	3.33e-05	%
Smc	5.22	µm
Sxp	7.64	µm
**Spatial Parameters**		
Sal	19.8	µm
Str	0.630	
Std	19.3	°
**Hybrid Parameters**		
Sdq	4.92	
Sdr	155	%
**Functional Parameters (Vol...**		
Vm	0.185	µm³/µm²
Vv	5.41	µm³/µm²
Vmp	0.185	µm³/µm²
Vmc	3.50	µm³/µm²
Vvc	4.94	µm³/µm²
Vvv	0.467	µm³/µm²
**Functional Parameters (Str...**		
Sk	5.80	µm
Spk	2.86	µm
Svk	3.56	µm
Smr1	11.6	%
Smr2	87.5	%
Spq	*****	
Svq	*****	
Smq	*****	

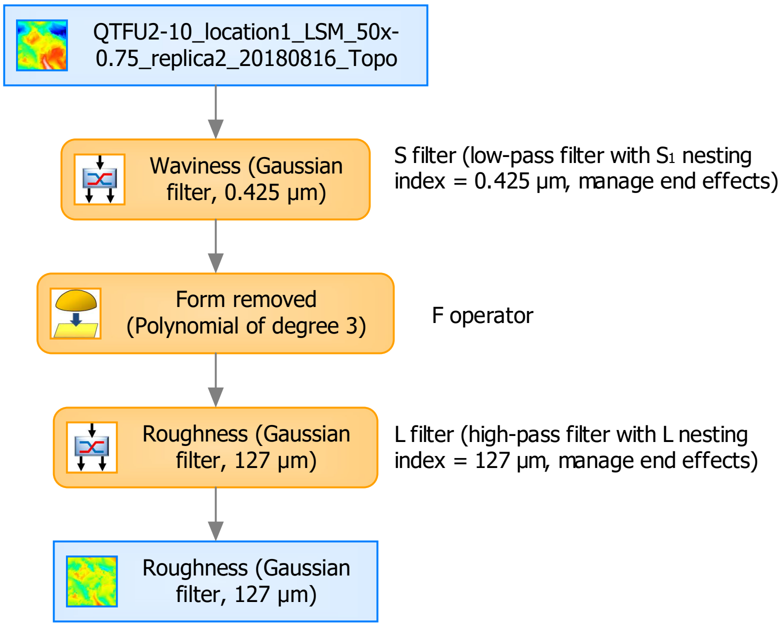

Quartzite QTFU2-10
50x objective, NA = 0.75 or 0.95

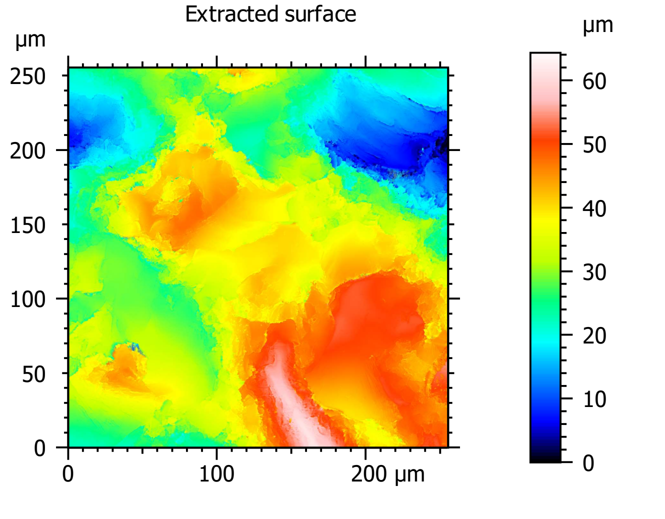

Identity card - QTFU2-10_location1_LSM_5...		
Name:	QTFU2-10_location1_LSM_50x-0.75_replica3_20...	
Created on:	8/16/2018 12:16:49 PM	
**Axis:**	**X**	
Length:	255	µm
Size:	3000	points
Spacing:	85.2	nm
**Axis:**	**Y**	
Length:	255	µm
Size:	3000	points
Spacing:	85.2	nm
**Axis:**	**Z**	
Length:	64.3	µm
Size:	65532	digits
Spacing:	0.981	nm
NMP ratio:	0.00 % (0 Pts)	

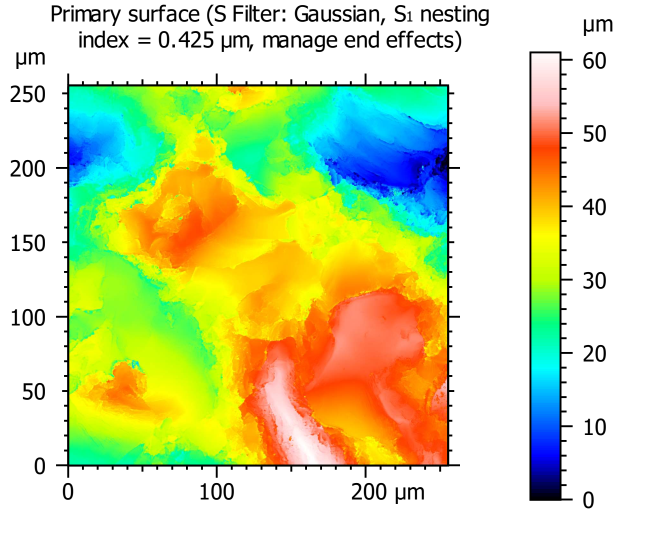

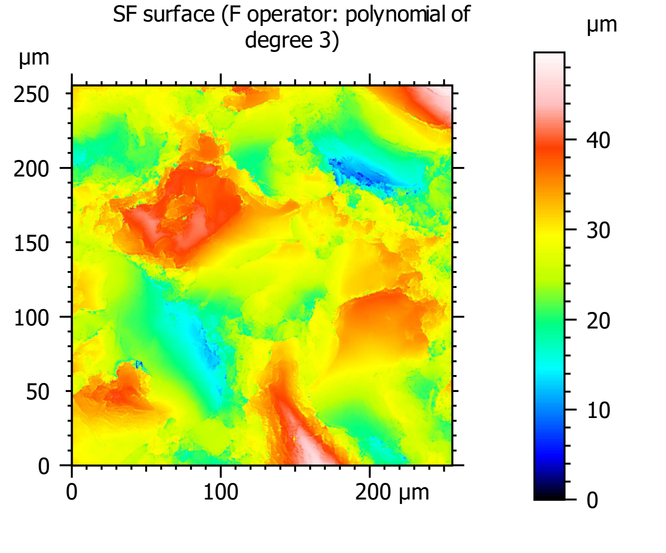

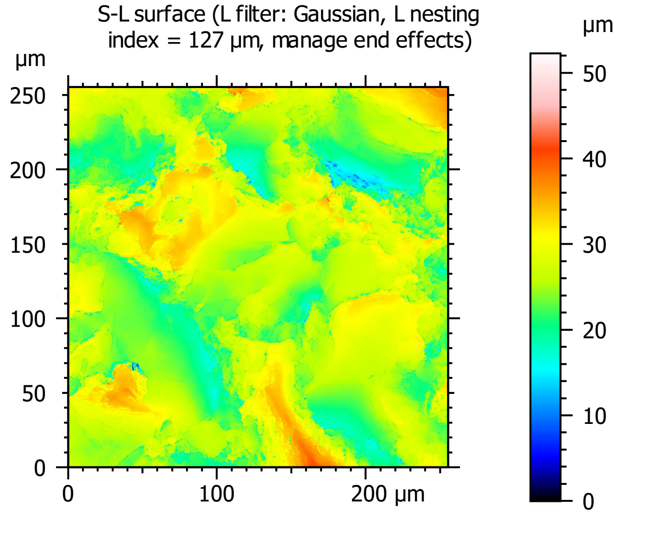

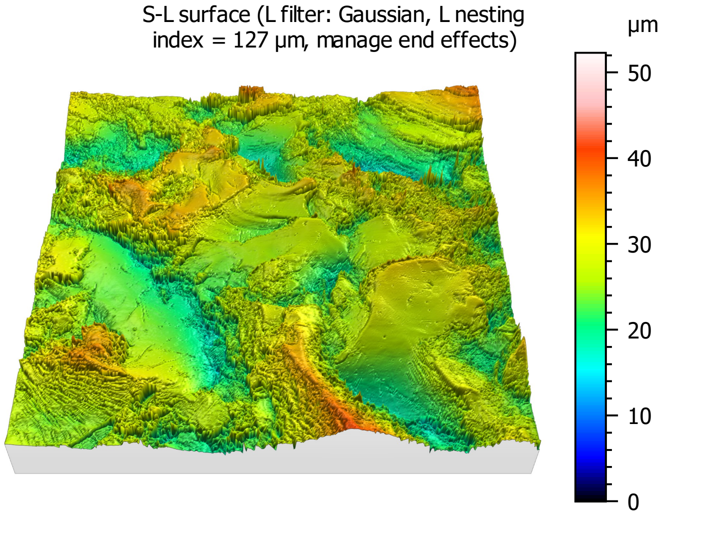

ISO 25178		
**Height Parameters**		
Sq	4.06	µm
Ssk	-0.00287	
Sku	3.12	
Sp	26.3	µm
Sv	26.0	µm
Sz	52.3	µm
Sa	3.21	µm
**Functional Parameters**		
Smr	1.11e-05	%
Smc	5.34	µm
Sxp	7.77	µm
**Spatial Parameters**		
Sal	19.8	µm
Str	0.630	
Std	20.0	°
**Hybrid Parameters**		
Sdq	5.02	
Sdr	159	%
**Functional Parameters (Vol...**		
Vm	0.187	µm^3/µm^2
Vv	5.52	µm^3/µm^2
Vmp	0.187	µm^3/µm^2
Vmc	3.56	µm^3/µm^2
Vvc	5.05	µm^3/µm^2
Vvv	0.478	µm^3/µm^2
**Functional Parameters (Str...**		
Sk	5.91	µm
Spk	2.90	µm
Svk	3.68	µm
Smr1	11.6	%
Smr2	87.7	%
Spq	*****	
Svq	*****	
Smq	*****	

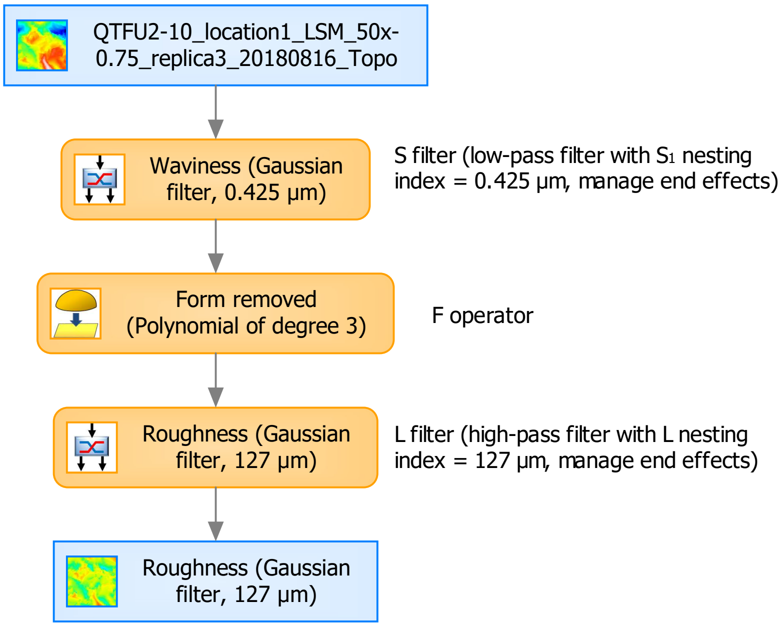

Quartzite QTFU2-10
50x objective, NA = 0.75 or 0.95

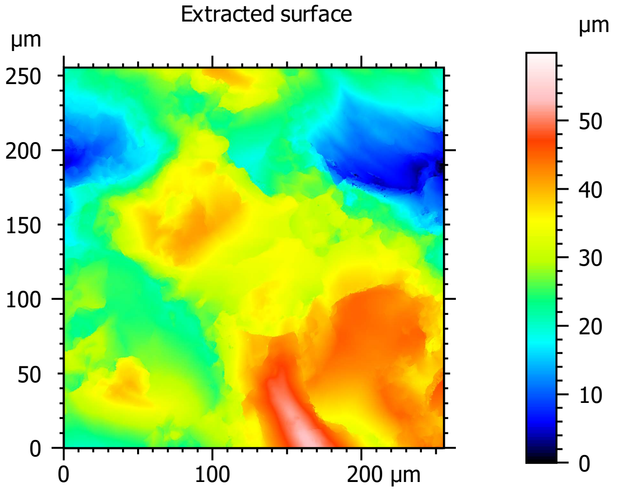

**Identity card - QTFU2-10_location1_LSM_5...**		
Name:	QTFU2-10_location1_LSM_50x-0.95_replica1_20...	
Created on:	8/16/2018 10:33:48 AM	
**Axis:**	**X**	
Length:	255	µm
Size:	3000	points
Spacing:	85.2	nm
**Axis:**	**Y**	
Length:	255	µm
Size:	3000	points
Spacing:	85.2	nm
**Axis:**	**Z**	
Length:	59.9	µm
Size:	65532	digits
Spacing:	0.914	nm
NMP ratio:	0.00 % (0 Pts)	

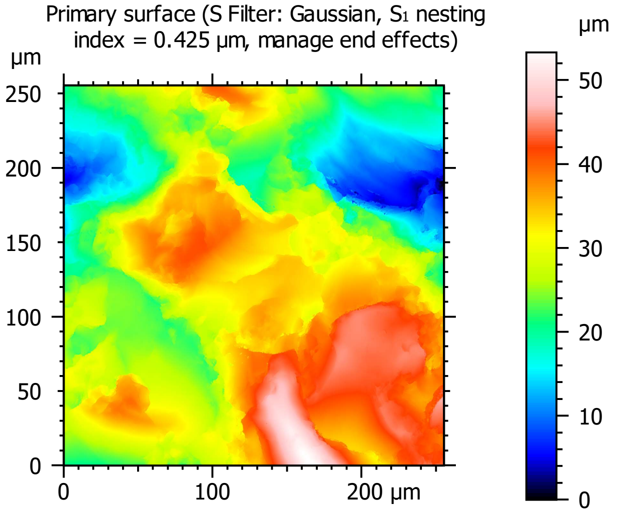

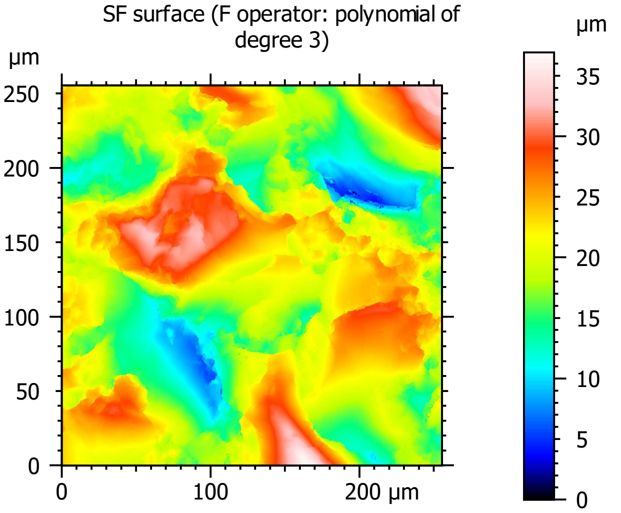

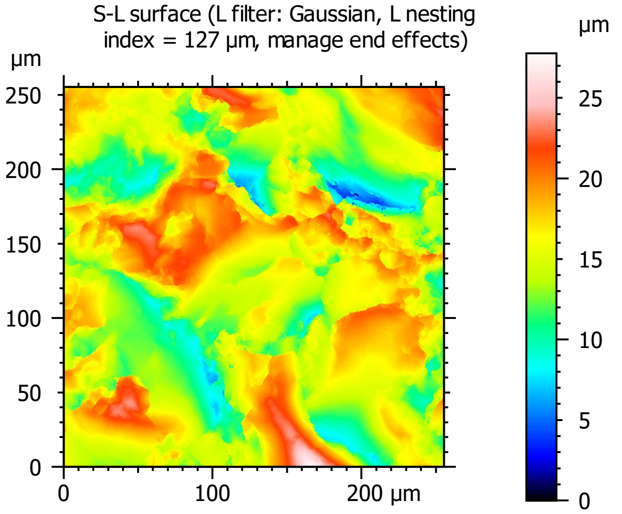

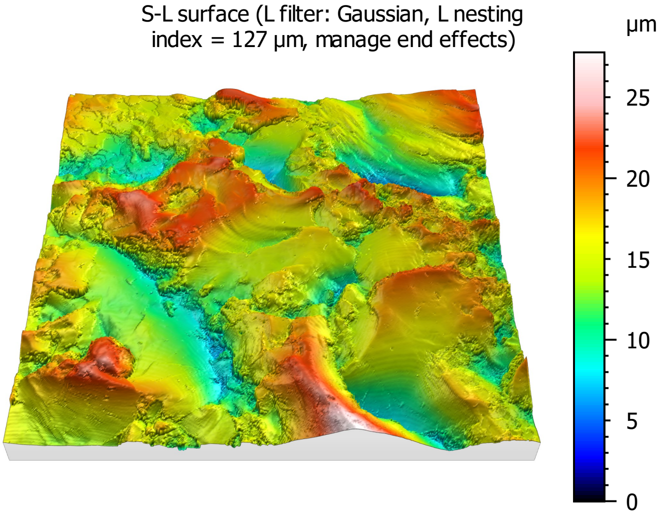

ISO 25178		
**Height Parameters**		
Sq	3.42	µm
Ssk	-0.0409	
Sku	2.93	
Sp	12.4	µm
Sv	15.3	µm
Sz	27.8	µm
Sa	2.72	µm
**Functional Parameters**		
Smr	0.0435	%
Smc	4.56	µm
Sxp	6.76	µm
**Spatial Parameters**		
Sal	20.2	µm
Str	0.606	
Std	129	°
**Hybrid Parameters**		
Sdq	1.93	
Sdr	40.8	%
**Functional Parameters (V...**		
Vm	0.140	µm³/µm²
Vv	4.70	µm³/µm²
Vmp	0.140	µm³/µm²
Vmc	3.10	µm³/µm²
Vvc	4.31	µm³/µm²
Vvv	0.394	µm³/µm²
**Functional Parameters (S...**		
Sk	4.70	µm
Spk	2.17	µm
Svk	2.77	µm
Smr1	11.6	%
Smr2	86.5	%
Spq	*****	
Svq	*****	
Smq	*****	

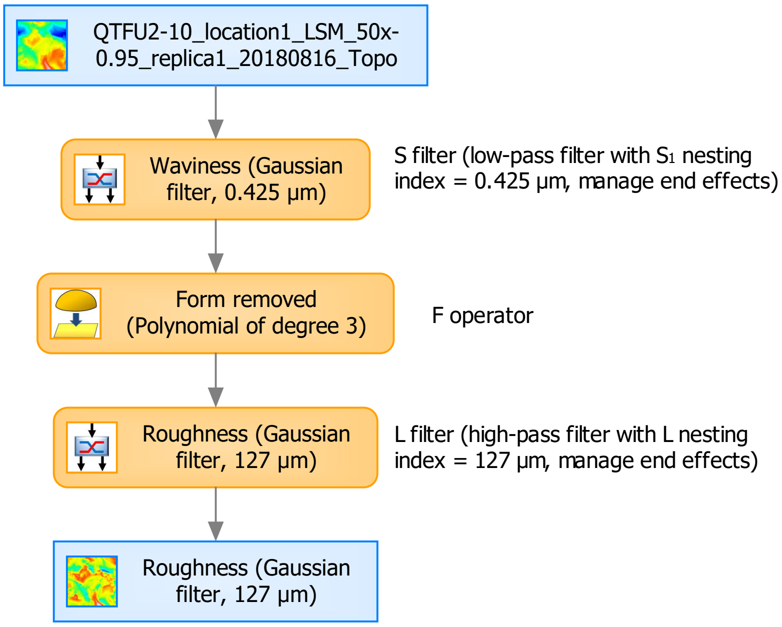

Quartzite QTFU2-10
50x objective, NA = 0.75 or 0.95

Identity card - QTFU2-10_location1_LSM_5...		
Name:	QTFU2-10_location1_LSM_50x-0.95_replica2_20...	
Created on:	8/16/2018 11:12:49 AM	
**Axis:**	**X**	
Length:	255	µm
Size:	3000	points
Spacing:	85.2	nm
**Axis:**	**Y**	
Length:	255	µm
Size:	3000	points
Spacing:	85.2	nm
**Axis:**	**Z**	
Length:	57.6	µm
Size:	65532	digits
Spacing:	0.879	nm
NMP ratio:	0.00 % (0 Pts)	

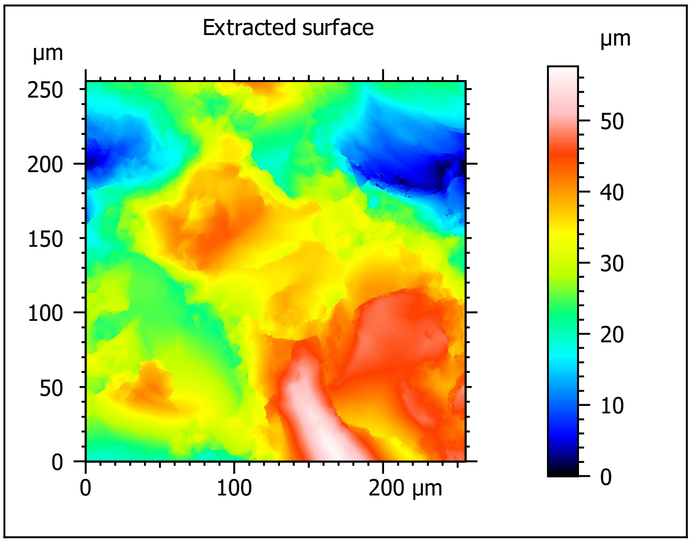

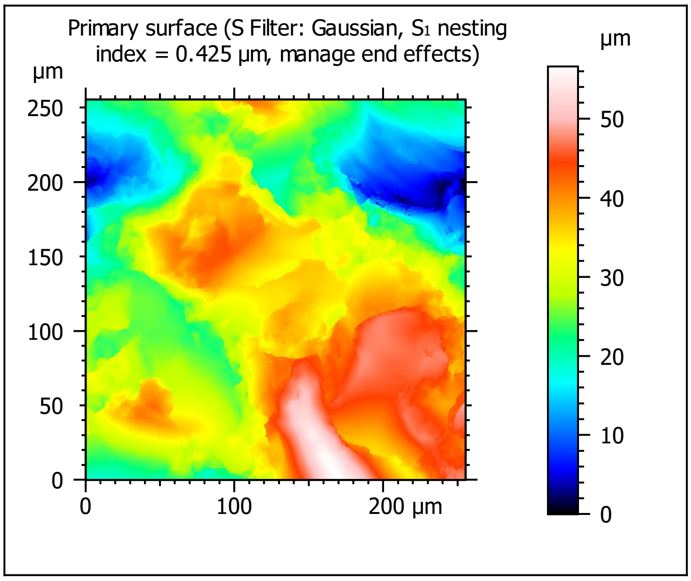

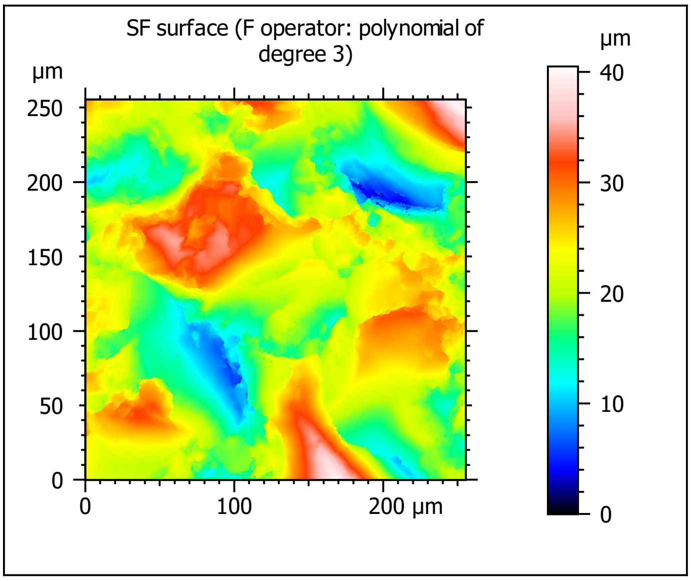

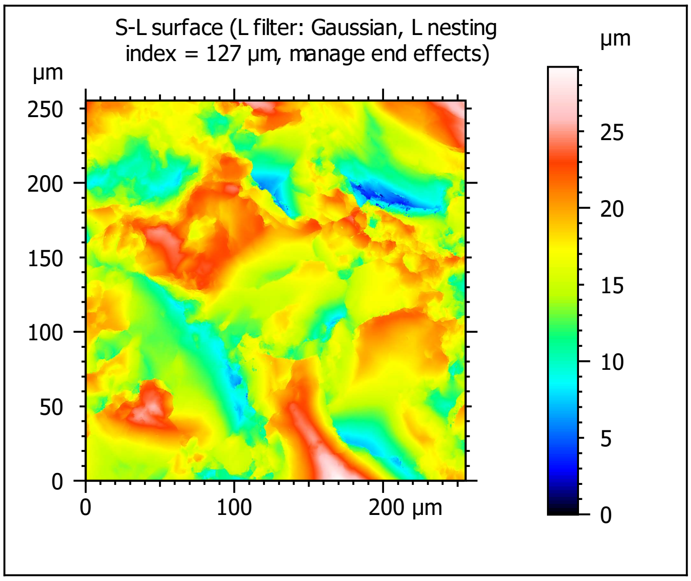

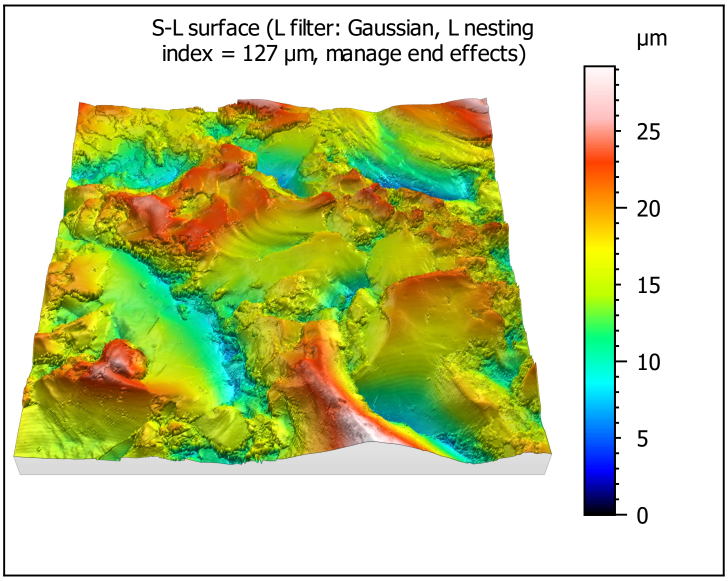

ISO 25178		
**Height Parameters**		
Sq	3.80	µm
Ssk	-0.0243	
Sku	2.96	
Sp	13.0	µm
Sv	16.2	µm
Sz	29.2	µm
Sa	3.01	µm
**Functional Parameters**		
Smr	0.0446	%
Smc	4.99	µm
Sxp	7.52	µm
**Spatial Parameters**		
Sal	20.2	µm
Str	0.618	
Std	129	°
**Hybrid Parameters**		
Sdq	2.09	
Sdr	46.3	%
**Functional Parameters (V...**		
Vm	0.168	µm³/µm²
Vv	5.16	µm³/µm²
Vmp	0.168	µm³/µm²
Vmc	3.40	µm³/µm²
Vvc	4.71	µm³/µm²
Vvv	0.445	µm³/µm²
**Functional Parameters (S...**		
Sk	5.34	µm
Spk	2.24	µm
Svk	2.97	µm
Smr1	11.0	%
Smr2	87.0	%
Spq	*****	
Svq	*****	
Smq	*****	

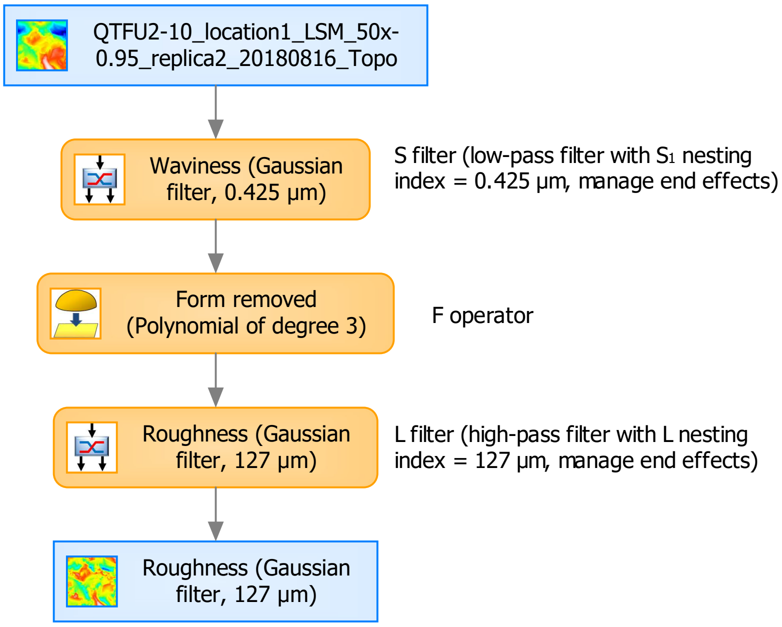

Quartzite QTFU2-10
50x objective, NA = 0.75 or 0.95

Identity card - QTFU2-10_location1_LSM_5...		
Name:	QTFU2-10_location1_LSM_50x-0.95_replica3_20...	
Created on:	8/16/2018 11:55:19 AM	
**Axis:**	**X**	
Length:	255	µm
Size:	3000	points
Spacing:	85.2	nm
**Axis:**	**Y**	
Length:	255	µm
Size:	3000	points
Spacing:	85.2	nm
**Axis:**	**Z**	
Length:	58.6	µm
Size:	65532	digits
Spacing:	0.895	nm
NMP ratio:	0.00 % (0 Pts)	

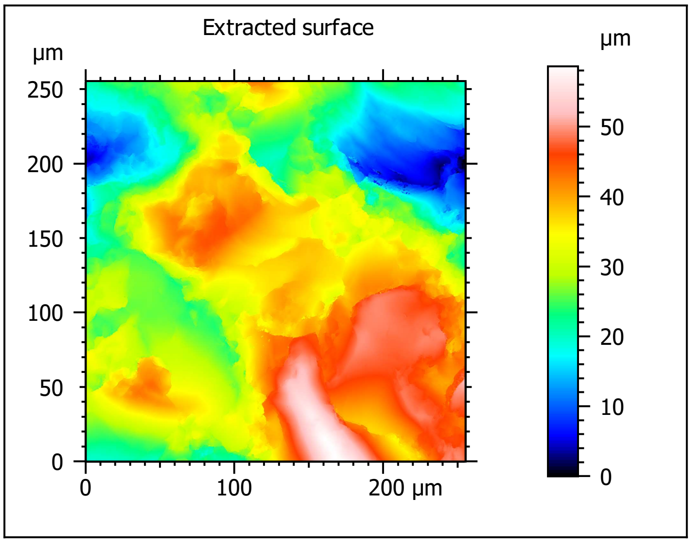

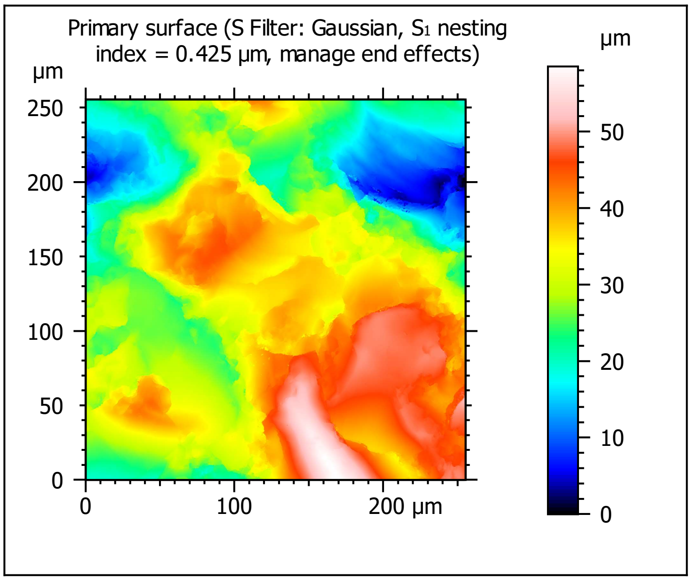

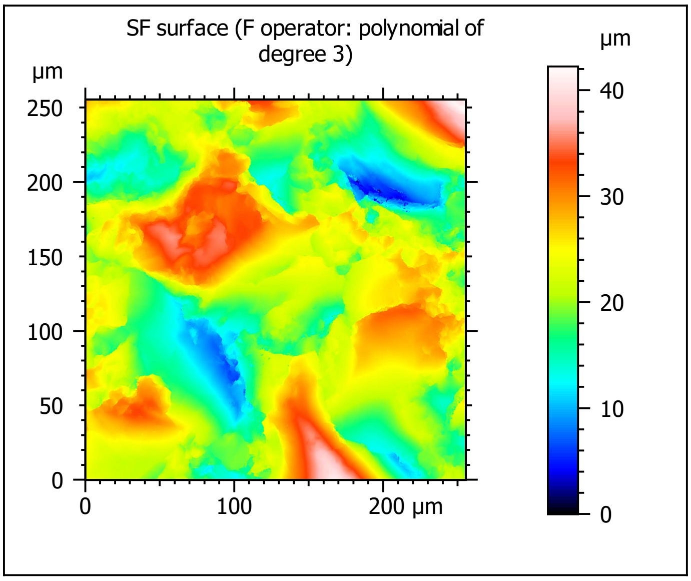

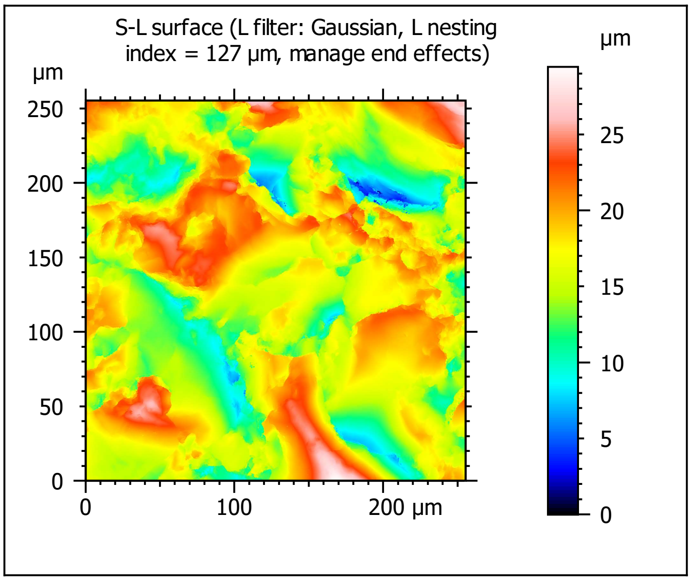

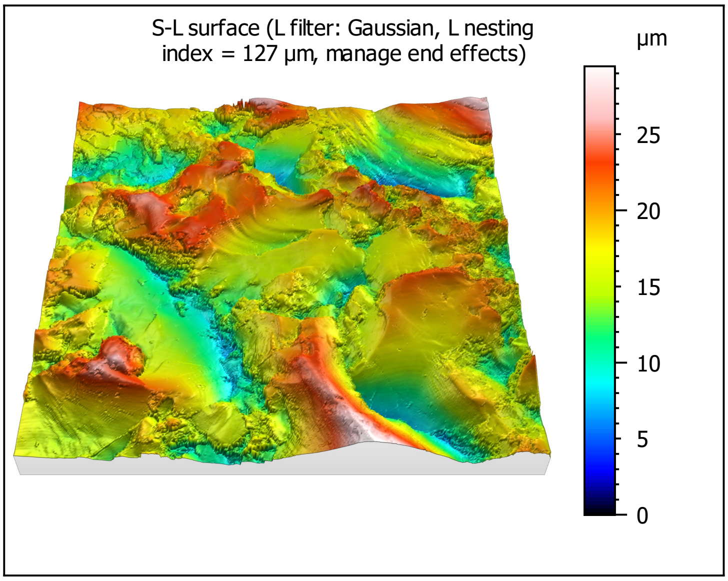

ISO 25178		
**Height Parameters**		
Sq	3.90	µm
Ssk	-0.0236	
Sku	2.97	
Sp	13.0	µm
Sv	16.5	µm
Sz	29.5	µm
Sa	3.09	µm
**Functional Parameters**		
Smr	0.0595	%
Smc	5.13	µm
Sxp	7.69	µm
**Spatial Parameters**		
Sal	20.2	µm
Str	0.622	
Std	129	°
**Hybrid Parameters**		
Sdq	2.13	
Sdr	47.8	%
**Functional Parameters (V...**		
Vm	0.171	µm³/µm²
Vv	5.30	µm³/µm²
Vmp	0.171	µm³/µm²
Vmc	3.48	µm³/µm²
Vvc	4.84	µm³/µm²
Vvv	0.458	µm³/µm²
**Functional Parameters (S...**		
Sk	5.58	µm
Spk	2.26	µm
Svk	2.99	µm
Smr1	10.7	%
Smr2	87.4	%
Spq	*****	
Svq	*****	
Smq	*****	

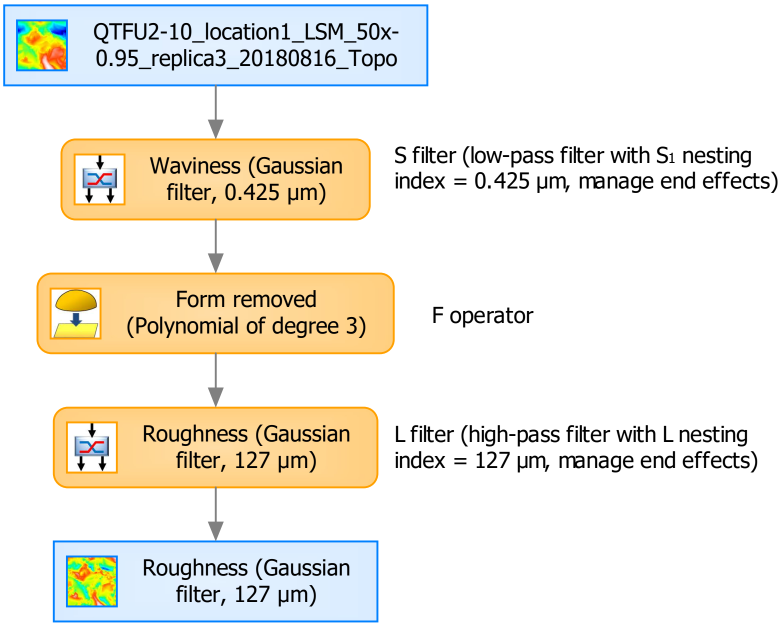

Quartzite QTFU2-10
50x objective, NA = 0.75 or 0.95

**Identity card - QTFU2-10_location2_LSM_5...**		
Name:	QTFU2-10_location2_LSM_50x-0.75_replica1_20...	
Created on:	8/17/2018 10:48:46 AM	
**Axis:**	**X**	
Length:	255	µm
Size:	3000	points
Spacing:	85.2	nm
**Axis:**	**Y**	
Length:	255	µm
Size:	3000	points
Spacing:	85.2	nm
**Axis:**	**Z**	
Length:	82.5	µm
Size:	65532	digits
Spacing:	1.26	nm
NMP ratio:	0.00 % (0 Pts)	

Extracted surface

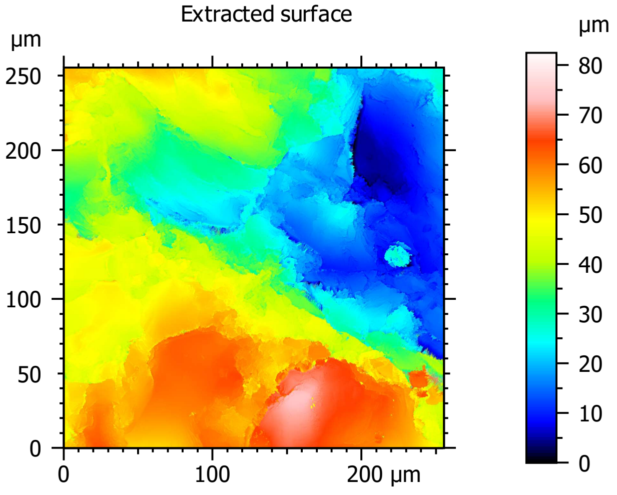

Primary surface (S Filter: Gaussian, S1 nesting index = 0.425 µm, manage end effects)

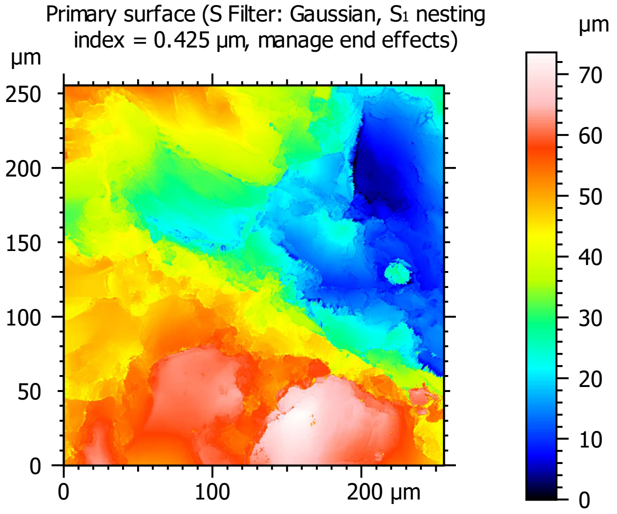

SF surface (F operator: polynomial of degree 3)

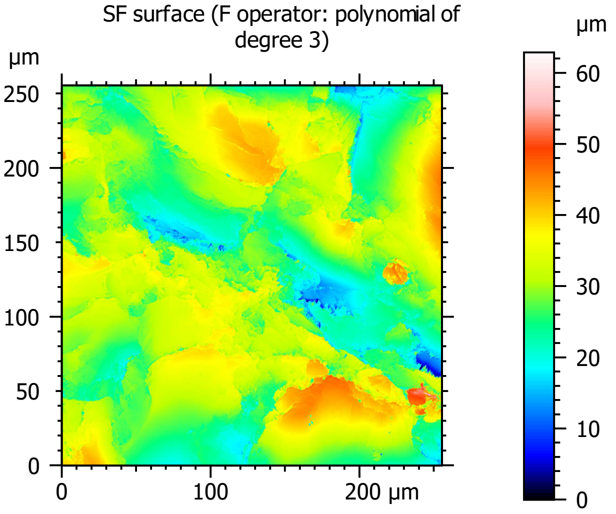

S-L surface (L filter: Gaussian, L nesting index = 127 µm, manage end effects)

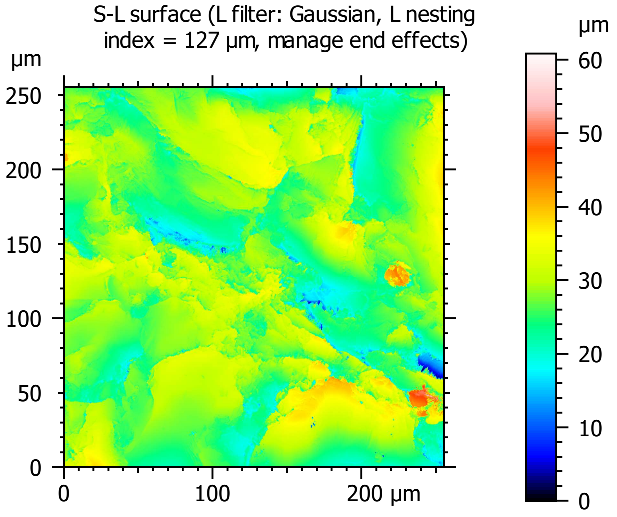

S-L surface (L filter: Gaussian, L nesting index = 127 µm, manage end effects)

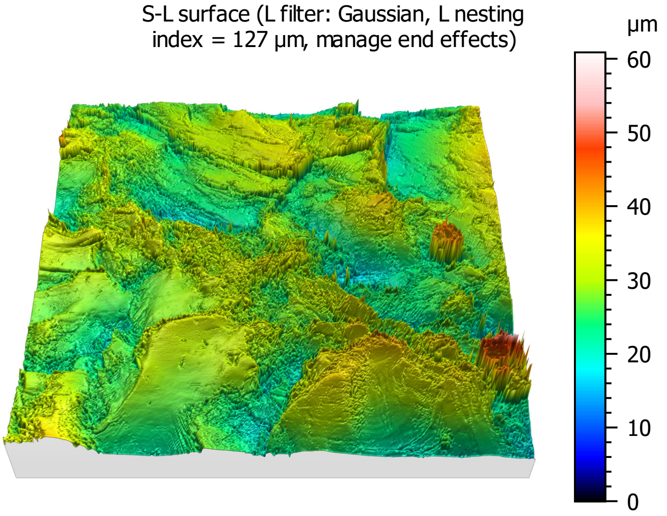

ISO 25178		
**Height Parameters**		
Sq	4.55	µm
Ssk	-0.0139	
Sku	4.29	
Sp	33.3	µm
Sv	27.6	µm
Sz	60.9	µm
Sa	3.50	µm
**Functional Parameters**		
Smr	7.78e-05	%
Smc	5.70	µm
Sxp	9.05	µm
**Spatial Parameters**		
Sal	19.4	µm
Str	0.734	
Std	150	°
**Hybrid Parameters**		
Sdq	6.63	
Sdr	190	%
**Functional Parameters (Vol...**		
Vm	0.231	µm³/µm²
Vv	5.94	µm³/µm²
Vmp	0.231	µm³/µm²
Vmc	3.91	µm³/µm²
Vvc	5.39	µm³/µm²
Vvv	0.548	µm³/µm²
**Functional Parameters (Str...**		
Sk	6.35	µm
Spk	4.27	µm
Svk	4.30	µm
Smr1	11.9	%
Smr2	86.6	%
Spq	*****	
Svq	*****	
Smq	*****	

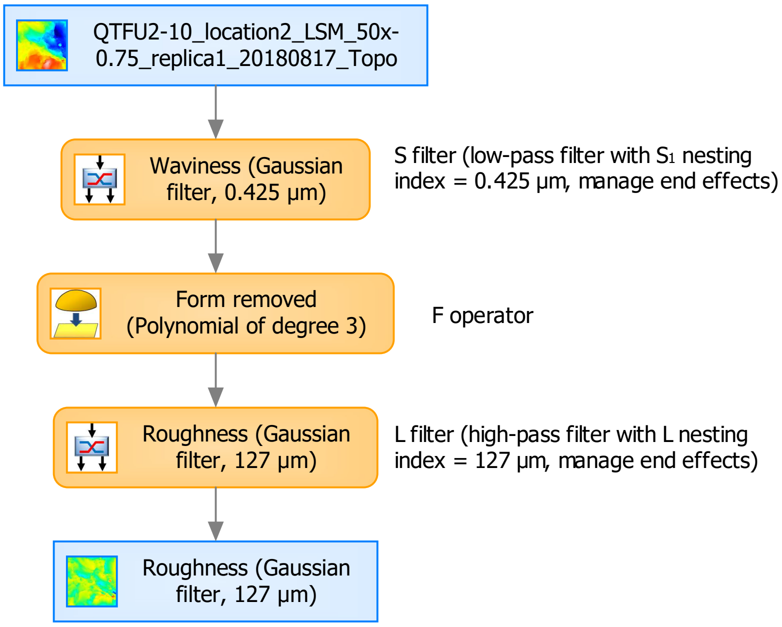

Quartzite QTFU2-10
50x objective, NA = 0.75 or 0.95

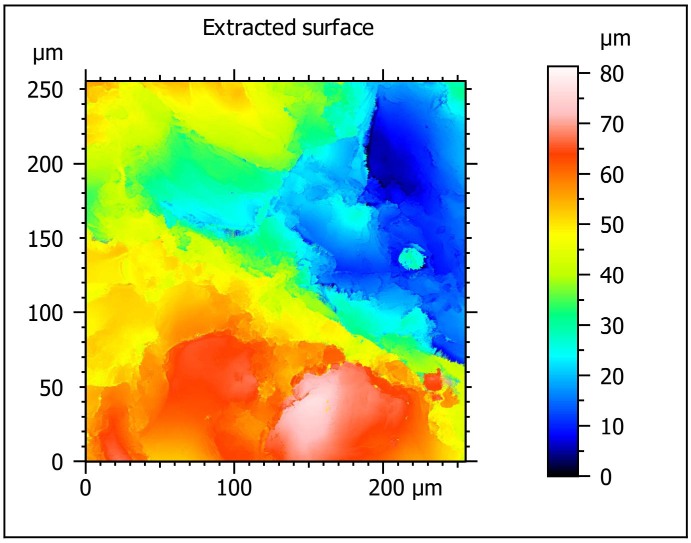

Identity card - QTFU2-10_location2_LSM_5...		
Name:	QTFU2-10_location2_LSM_50x-0.75_replica2_20...	
Created on:	8/17/2018 11:37:03 AM	
**Axis:**	**X**	
Length:	255	µm
Size:	3000	points
Spacing:	85.2	nm
**Axis:**	**Y**	
Length:	255	µm
Size:	3000	points
Spacing:	85.2	nm
**Axis:**	**Z**	
Length:	81.3	µm
Size:	65532	digits
Spacing:	1.24	nm
NMP ratio:	0.00 % (0 Pts)	

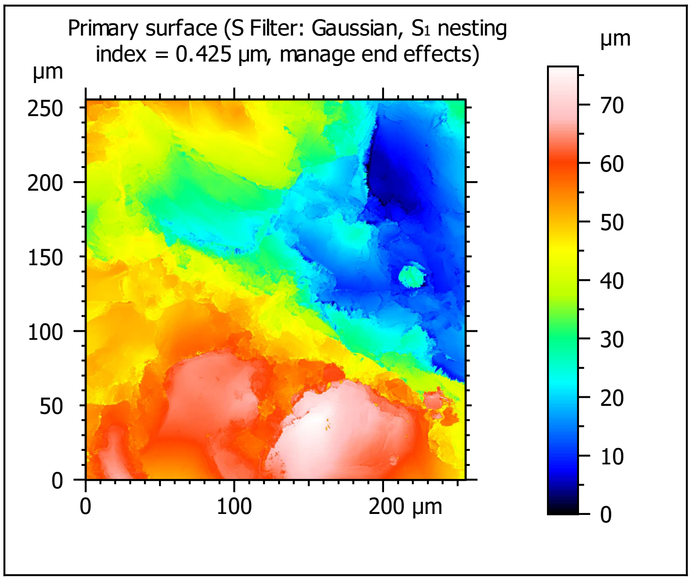

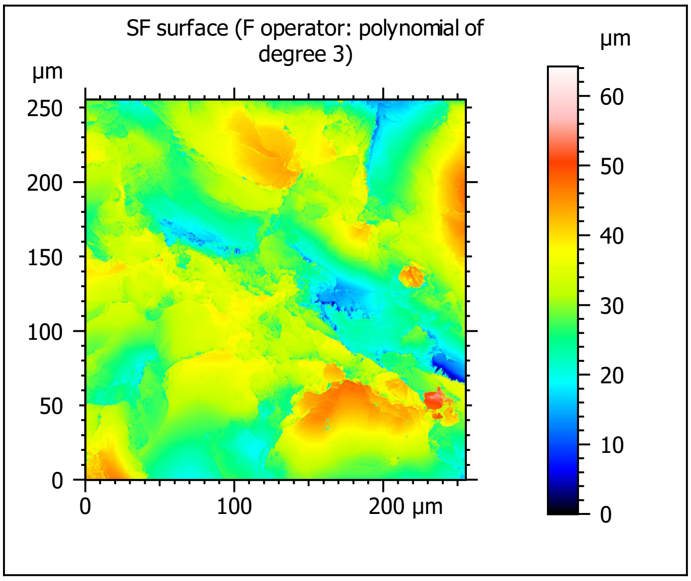

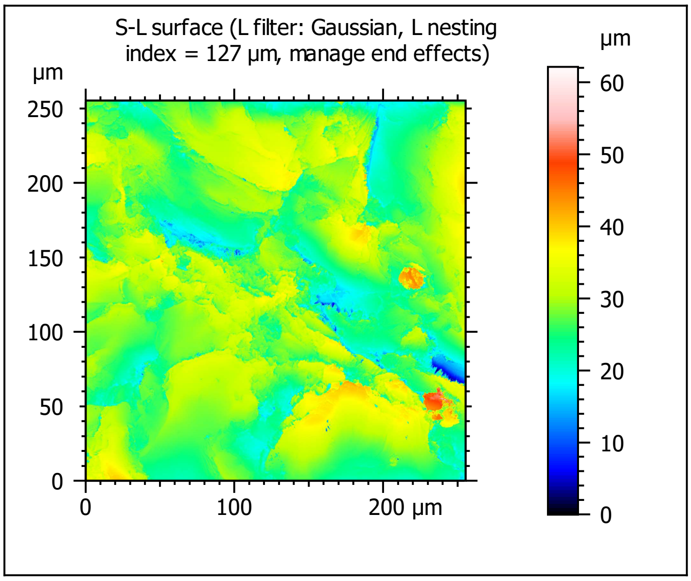

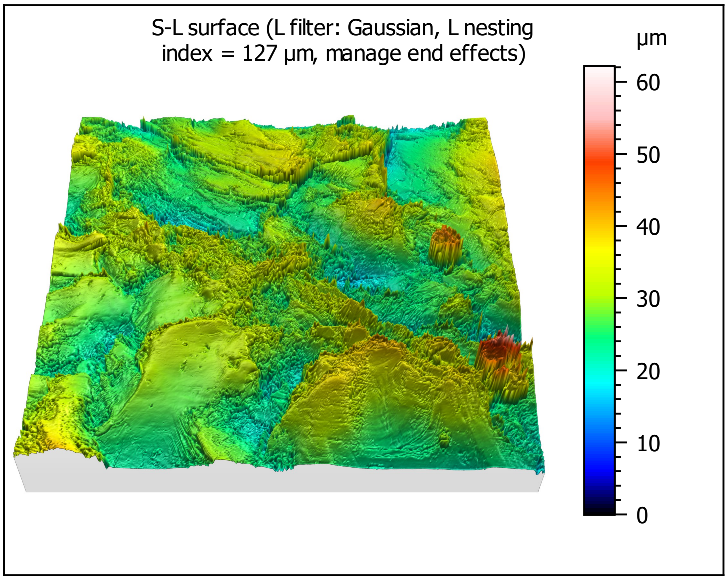

ISO 25178		
**Height Parameters**		
Sq	4.68	µm
Ssk	-0.00798	
Sku	4.35	
Sp	34.2	µm
Sv	27.9	µm
Sz	62.2	µm
Sa	3.60	µm
**Functional Parameters**		
Smr	5.65e-05	%
Smc	5.81	µm
Sxp	9.24	µm
**Spatial Parameters**		
Sal	19.8	µm
Str	0.778	
Std	150	°
**Hybrid Parameters**		
Sdq	6.76	
Sdr	196	%
**Functional Parameters (Vol...**		
Vm	0.244	µm³/µm²
Vv	6.05	µm³/µm²
Vmp	0.244	µm³/µm²
Vmc	4.01	µm³/µm²
Vvc	5.50	µm³/µm²
Vvv	0.550	µm³/µm²
**Functional Parameters (Str...**		
Sk	6.56	µm
Spk	4.47	µm
Svk	4.48	µm
Smr1	11.4	%
Smr2	86.9	%
Spq	*****	
Svq	*****	
Smq	*****	

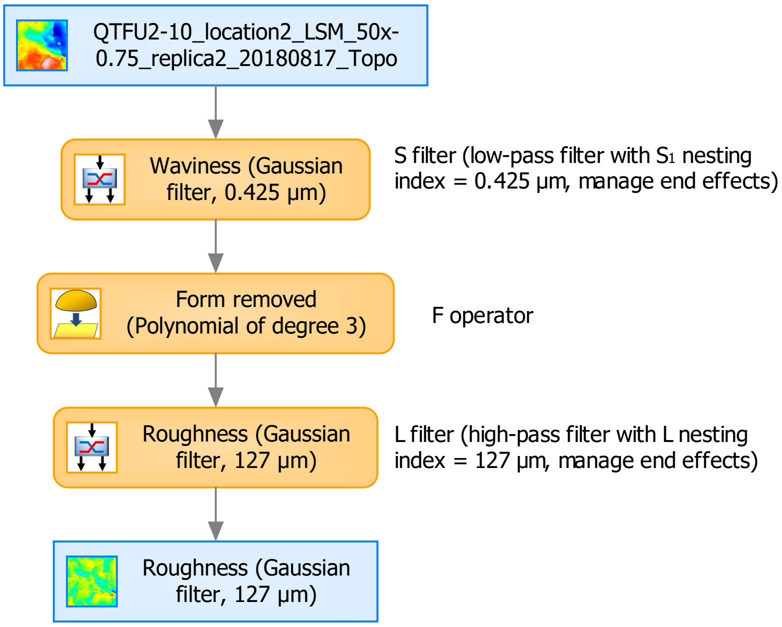

Quartzite QTFU2-10
50x objective, NA = 0.75 or 0.95

Extracted surface

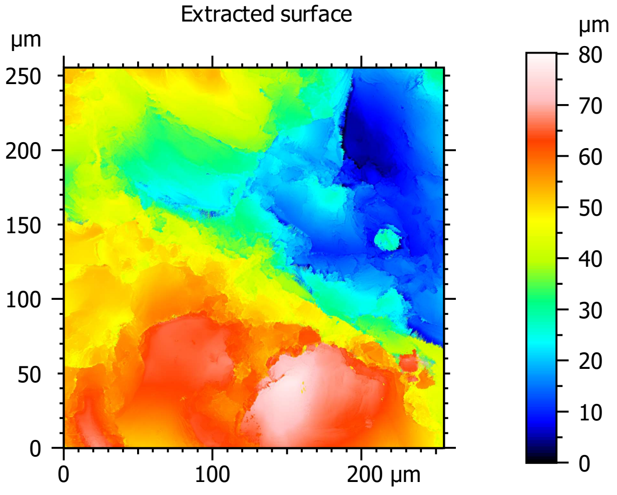

Identity card - QTFU2-10_location2_LSM_5...		
Name:	QTFU2-10_location2_LSM_50x-0.75_replica3_20...	
Created on:	8/17/2018 12:27:24 PM	
**Axis:**	**X**	
Length:	255	µm
Size:	3000	points
Spacing:	85.2	nm
**Axis:**	**Y**	
Length:	255	µm
Size:	3000	points
Spacing:	85.2	nm
**Axis:**	**Z**	
Length:	80.2	µm
Size:	65532	digits
Spacing:	1.22	nm
NMP ratio:	0.00 % (0 Pts)	

Primary surface (S Filter: Gaussian, S_1 nesting index = 0.425 µm, manage end effects)

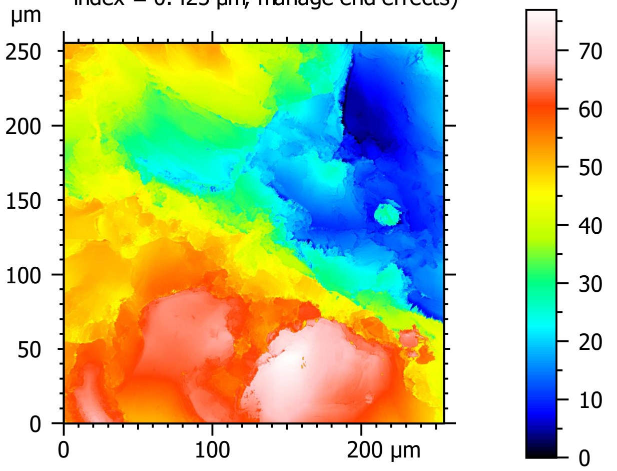

SF surface (F operator: polynomial of degree 3)

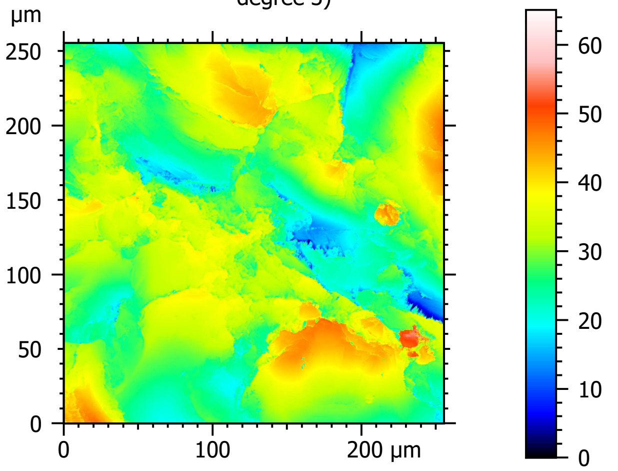

S-L surface (L filter: Gaussian, L nesting index = 127 µm, manage end effects)

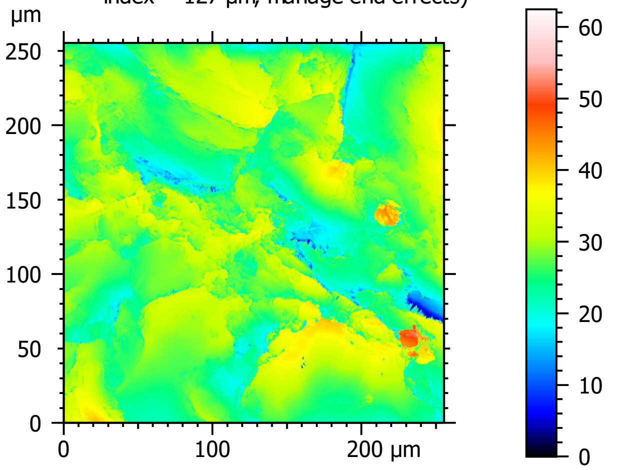

S-L surface (L filter: Gaussian, L nesting index = 127 µm, manage end effects)

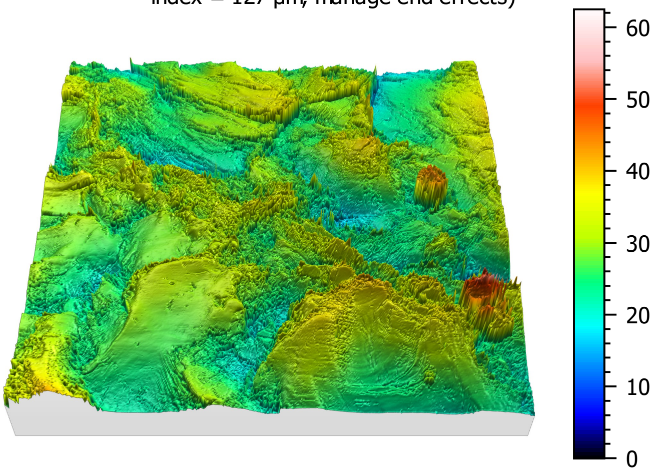

ISO 25178		
**Height Parameters**		
Sq	4.70	µm
Ssk	-0.00636	
Sku	4.41	
Sp	34.9	µm
Sv	27.6	µm
Sz	62.5	µm
Sa	3.62	µm
**Functional Parameters**		
Smr	4.44e-05	%
Smc	5.82	µm
Sxp	9.29	µm
**Spatial Parameters**		
Sal	19.7	µm
Str	0.763	
Std	150	°
**Hybrid Parameters**		
Sdq	6.75	
Sdr	196	%
**Functional Parameters (Vol...**		
Vm	0.251	$\mu m^3/\mu m^2$
Vv	6.07	$\mu m^3/\mu m^2$
Vmp	0.251	$\mu m^3/\mu m^2$
Vmc	3.97	$\mu m^3/\mu m^2$
Vvc	5.51	$\mu m^3/\mu m^2$
Vvv	0.563	$\mu m^3/\mu m^2$
**Functional Parameters (Str...**		
Sk	6.44	µm
Spk	4.56	µm
Svk	4.53	µm
Smr1	11.8	%
Smr2	86.5	%
Spq	*****	
Svq	*****	
Smq	*****	

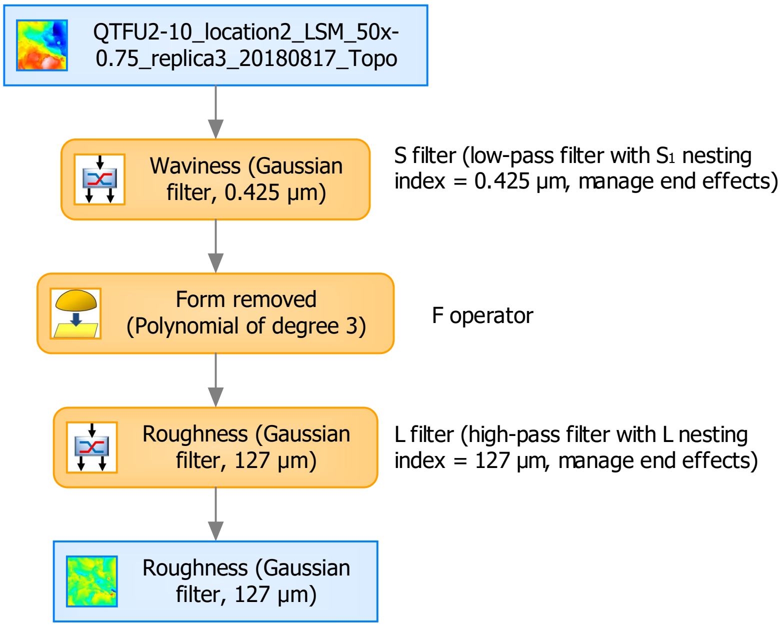

Quartzite QTFU2-10
50x objective, NA = 0.75 or 0.95

**Identity card - QTFU2-10_location2_LSM_5...**		
Name:	QTFU2-10_location2_LSM_50x-0.95_replica1_20...	
Created on:	8/17/2018 10:22:20 AM	
**Axis:**	**X**	
Length:	255	µm
Size:	3000	points
Spacing:	85.2	nm
**Axis:**	**Y**	
Length:	255	µm
Size:	3000	points
Spacing:	85.2	nm
**Axis:**	**Z**	
Length:	77.0	µm
Size:	65532	digits
Spacing:	1.18	nm
NMP ratio:	0.00 % (0 Pts)	

Extracted surface

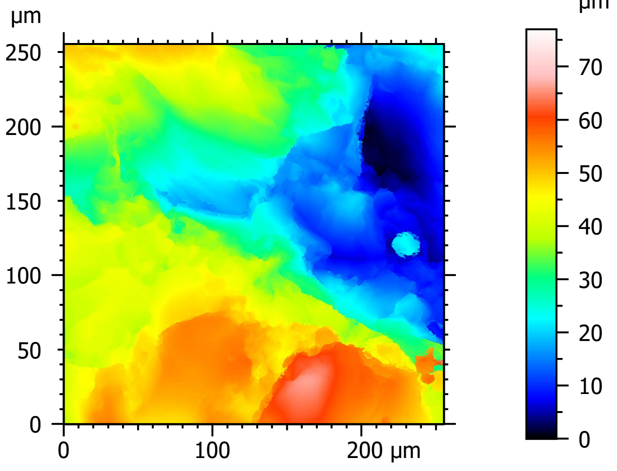

Primary surface (S Filter: Gaussian, S1 nesting index = 0.425 µm, manage end effects)

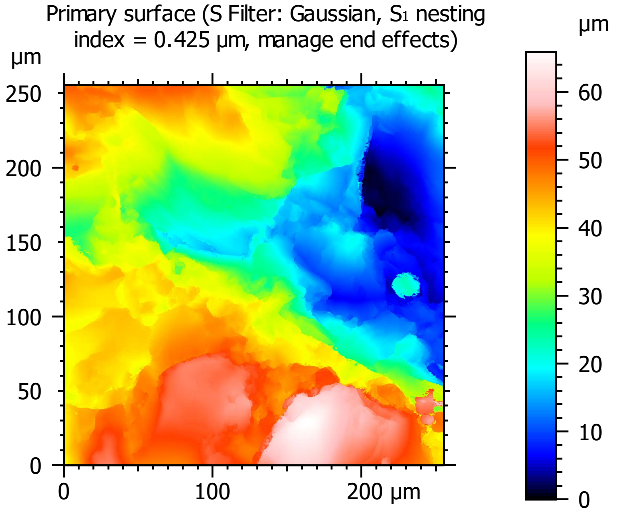

SF surface (F operator: polynomial of degree 3)

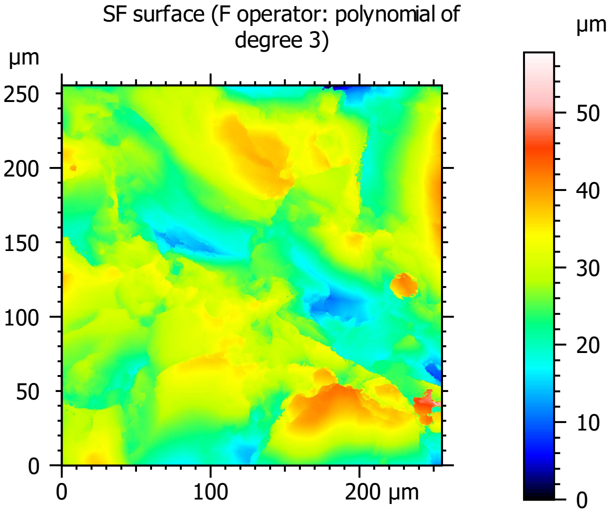

S-L surface (L filter: Gaussian, L nesting index = 127 µm, manage end effects)

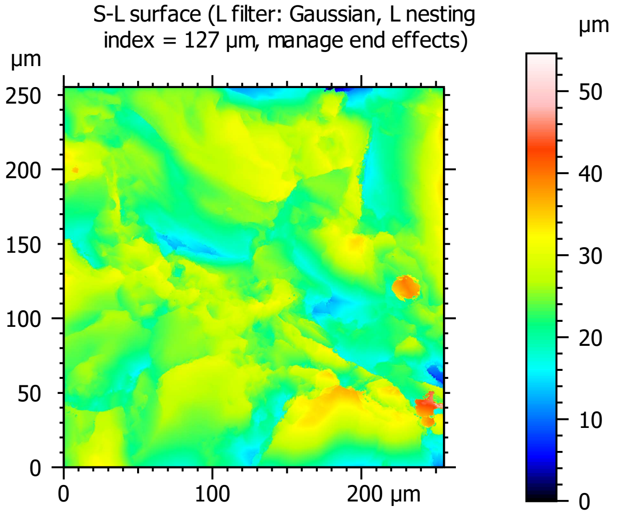

S-L surface (L filter: Gaussian, L nesting index = 127 µm, manage end effects)

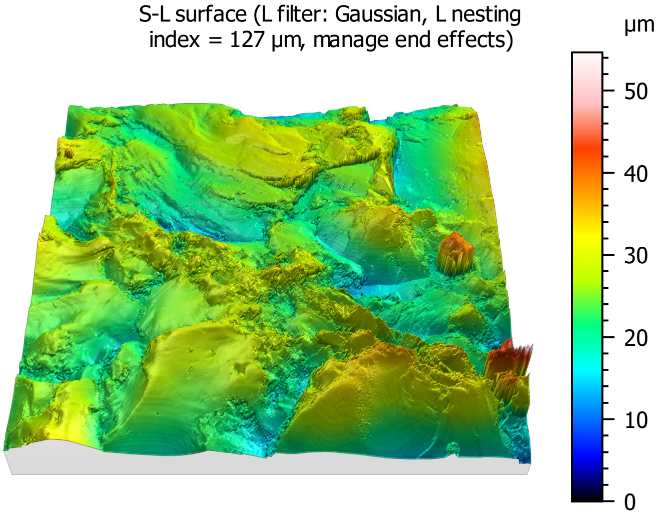

ISO 25178		
**Height Parameters**		
Sq	4.17	µm
Ssk	0.0218	
Sku	4.33	
Sp	30.2	µm
Sv	24.5	µm
Sz	54.7	µm
Sa	3.19	µm
**Functional Parameters**		
Smr	2.55e-05	%
Smc	5.16	µm
Sxp	8.43	µm
**Spatial Parameters**		
Sal	19.1	µm
Str	0.634	
Std	147	°
**Hybrid Parameters**		
Sdq	3.39	
Sdr	63.6	%
**Functional Parameters (Vol...**		
Vm	0.214	µm³/µm²
Vv	5.38	µm³/µm²
Vmp	0.214	µm³/µm²
Vmc	3.56	µm³/µm²
Vvc	4.86	µm³/µm²
Vvv	0.516	µm³/µm²
**Functional Parameters (Str...**		
Sk	5.44	µm
Spk	3.84	µm
Svk	3.74	µm
Smr1	13.1	%
Smr2	86.1	%
Spq	*****	
Svq	*****	
Smq	*****	

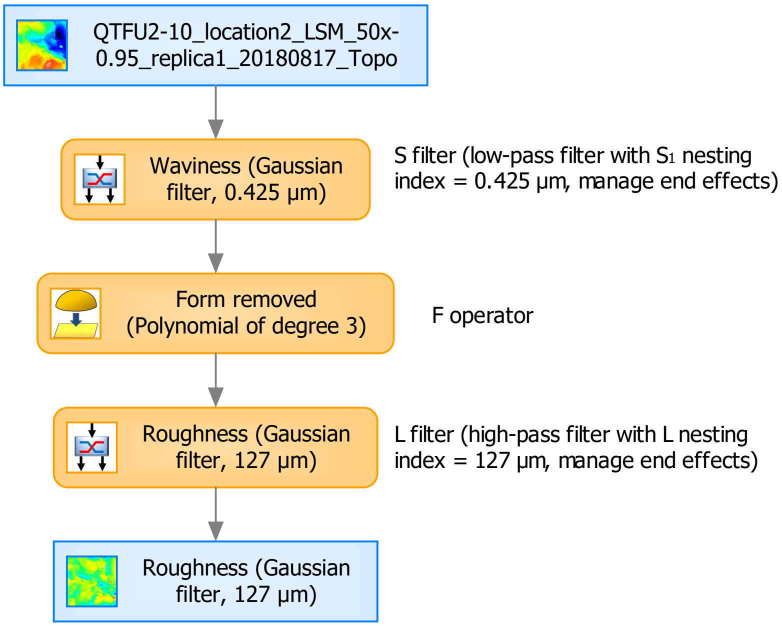

Quartzite QTFU2-10
50x objective, NA = 0.75 or 0.95

Identity card - QTFU2-10_location2_LSM_5...		
Name:	QTFU2-10_location2_LSM_50x-0.95_replica2_20...	
Created on:	8/17/2018 11:12:17 AM	
**Axis:**	**X**	
Length:	255	µm
Size:	3000	points
Spacing:	85.2	nm
**Axis:**	**Y**	
Length:	255	µm
Size:	3000	points
Spacing:	85.2	nm
**Axis:**	**Z**	
Length:	76.4	µm
Size:	65531	digits
Spacing:	1.17	nm
NMP ratio:	0.00 % (0 Pts)	

Extracted surface

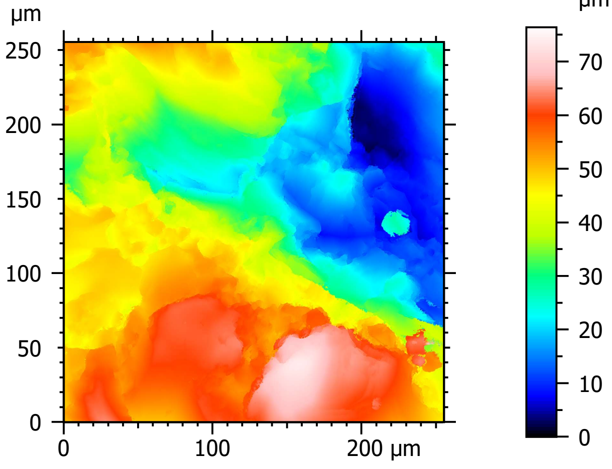

Primary surface (S Filter: Gaussian, S1 nesting index = 0.425 µm, manage end effects)

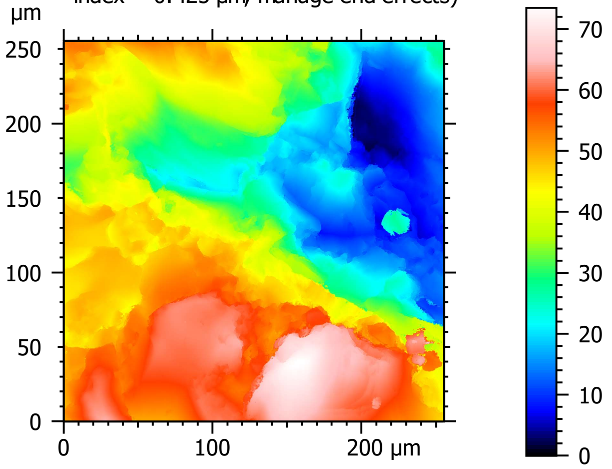

SF surface (F operator: polynomial of degree 3)

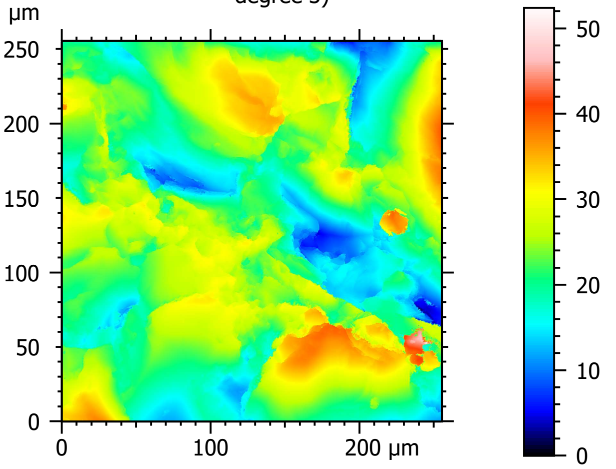

S-L surface (L filter: Gaussian, L nesting index = 127 µm, manage end effects)

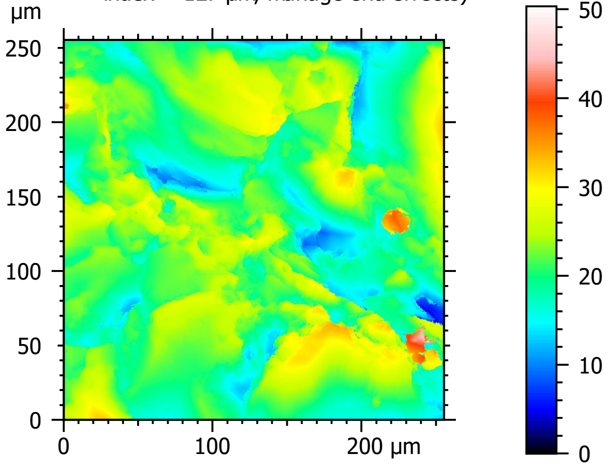

S-L surface (L filter: Gaussian, L nesting index = 127 µm, manage end effects)

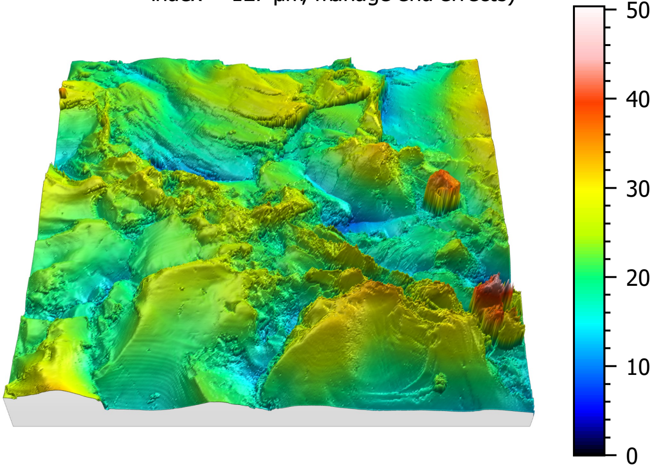

ISO 25178		
**Height Parameters**		
Sq	4.45	µm
Ssk	0.203	
Sku	3.97	
Sp	28.8	µm
Sv	21.6	µm
Sz	50.4	µm
Sa	3.44	µm
**Functional Parameters**		
Smr	0.000605	%
Smc	5.64	µm
Sxp	8.38	µm
**Spatial Parameters**		
Sal	20.4	µm
Str	0.785	
Std	147	°
**Hybrid Parameters**		
Sdq	3.68	
Sdr	70.1	%
**Functional Parameters (Vol...**		
Vm	0.239	µm³/µm²
Vv	5.88	µm³/µm²
Vmp	0.239	µm³/µm²
Vmc	3.89	µm³/µm²
Vvc	5.38	µm³/µm²
Vvv	0.496	µm³/µm²
**Functional Parameters (Str...**		
Sk	6.23	µm
Spk	4.13	µm
Svk	3.65	µm
Smr1	11.9	%
Smr2	87.7	%
Spq	*****	
Svq	*****	
Smq	*****	

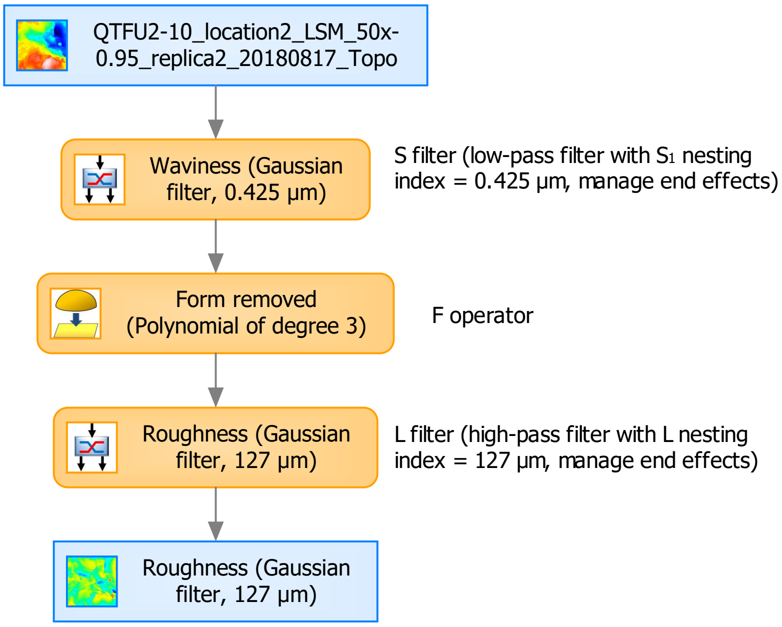

Quartzite QTFU2-10
50x objective, NA = 0.75 or 0.95

Identity card - QTFU2-10_location2_LSM_5...		
Name:	QTFU2-10_location2_LSM_50x-0.95_replica3_20...	
Created on:	8/17/2018 12:00:25 PM	
**Axis:**	**X**	
Length:	255	µm
Size:	3000	points
Spacing:	85.2	nm
**Axis:**	**Y**	
Length:	255	µm
Size:	3000	points
Spacing:	85.2	nm
**Axis:**	**Z**	
Length:	76.5	µm
Size:	65532	digits
Spacing:	1.17	nm
NMP ratio:	0.00 % (0 Pts)	

Extracted surface

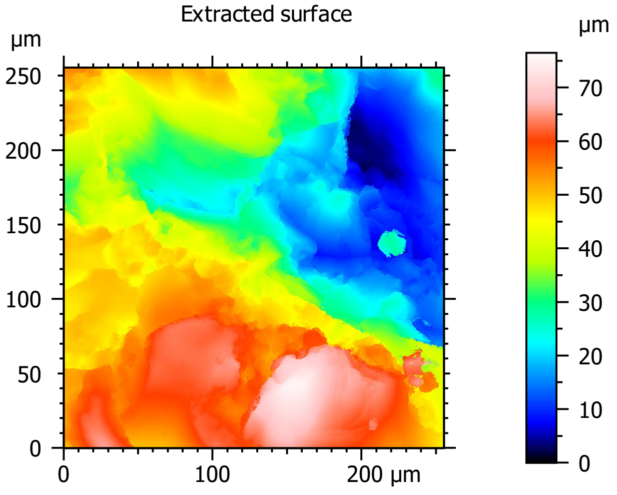

Primary surface (S Filter: Gaussian, S1 nesting index = 0.425 µm, manage end effects)

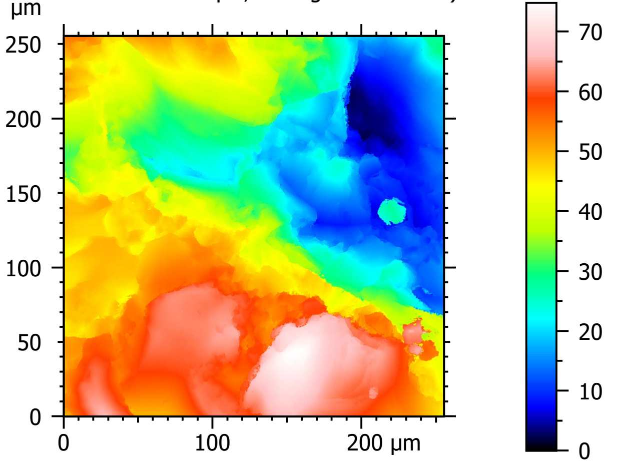

SF surface (F operator: polynomial of degree 3)

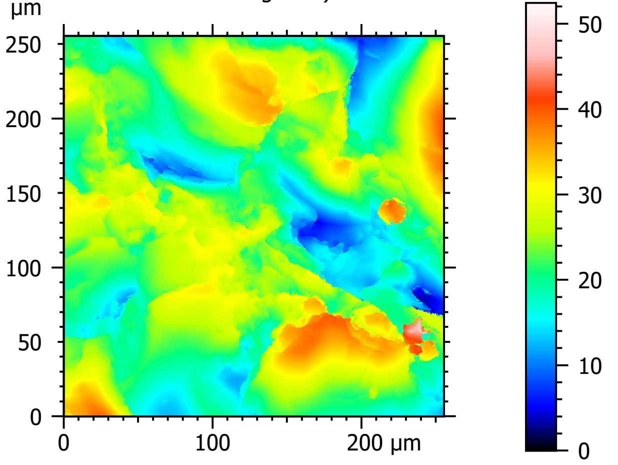

S-L surface (L filter: Gaussian, L nesting index = 127 µm, manage end effects)

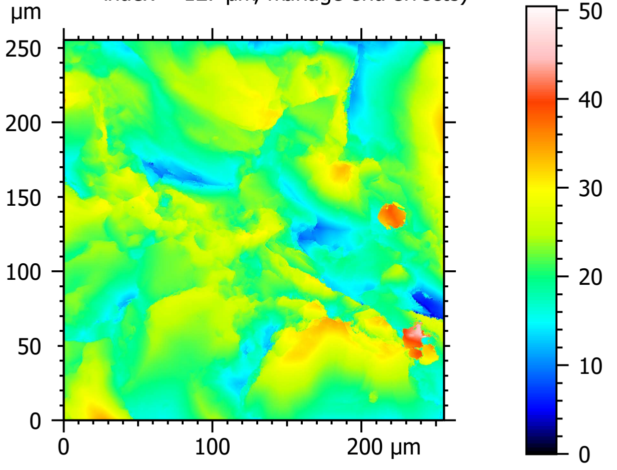

S-L surface (L filter: Gaussian, L nesting index = 127 µm, manage end effects)

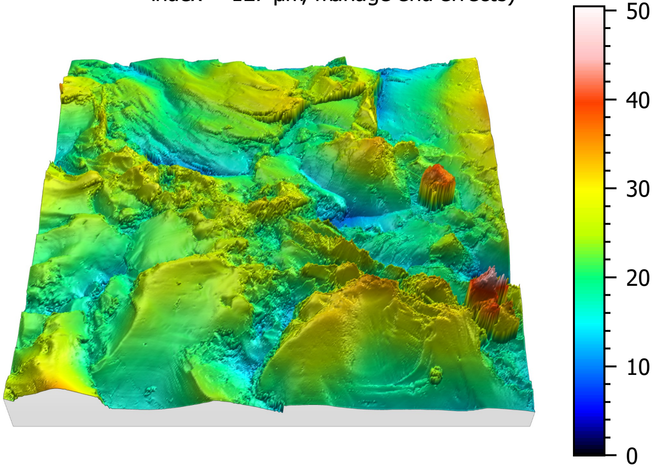

ISO 25178		
**Height Parameters**		
Sq	4.51	µm
Ssk	0.205	
Sku	4.01	
Sp	28.6	µm
Sv	21.9	µm
Sz	50.5	µm
Sa	3.49	µm
**Functional Parameters**		
Smr	0.000474	%
Smc	5.68	µm
Sxp	8.57	µm
**Spatial Parameters**		
Sal	20.5	µm
Str	0.811	
Std	147	°
**Hybrid Parameters**		
Sdq	3.73	
Sdr	70.8	%
**Functional Parameters (Vol...**		
Vm	0.247	$\mu m^3/\mu m^2$
Vv	5.93	$\mu m^3/\mu m^2$
Vmp	0.247	$\mu m^3/\mu m^2$
Vmc	3.92	$\mu m^3/\mu m^2$
Vvc	5.43	$\mu m^3/\mu m^2$
Vvv	0.501	$\mu m^3/\mu m^2$
**Functional Parameters (Str...**		
Sk	6.23	µm
Spk	4.28	µm
Svk	3.74	µm
Smr1	11.8	%
Smr2	87.4	%
Spq	*****	
Svq	*****	
Smq	*****	

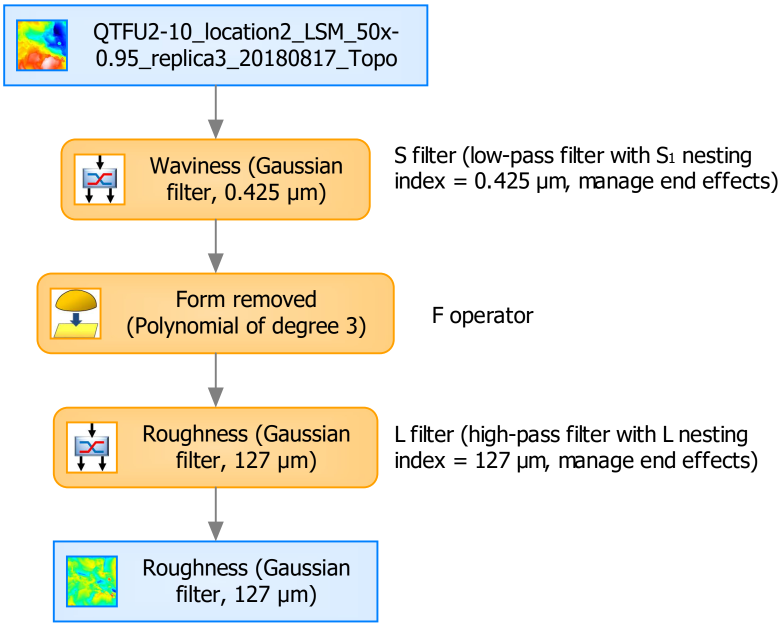

Quartzite QTFU2-10
50x objective, NA = 0.75 or 0.95

Extracted surface

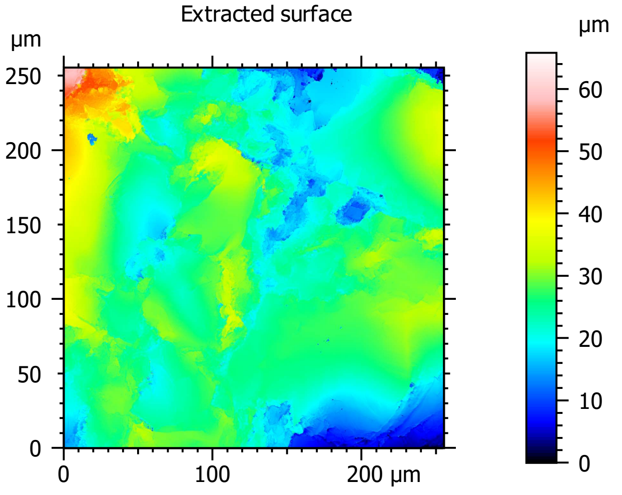

Identity card - QTFU2-10_location3_LSM_5...		
Name:	QTFU2-10_location3_LSM_50x-0.75_replica1_20...	
Created on:	8/20/2018 1:59:54 PM	
**Axis:**	**X**	
Length:	255	µm
Size:	3000	points
Spacing:	85.2	nm
**Axis:**	**Y**	
Length:	255	µm
Size:	3000	points
Spacing:	85.2	nm
**Axis:**	**Z**	
Length:	65.8	µm
Size:	65532	digits
Spacing:	1.00	nm
NMP ratio:	0.00 % (0 Pts)	

Primary surface (S Filter: Gaussian, S_1 nesting index = 0.425 µm, manage end effects)

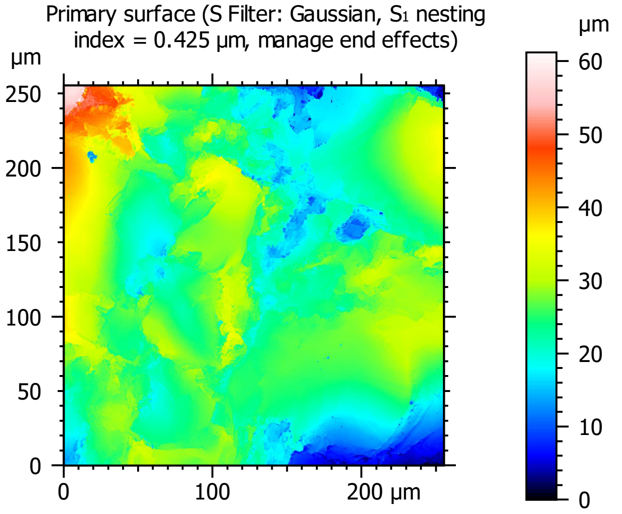

SF surface (F operator: polynomial of degree 3)

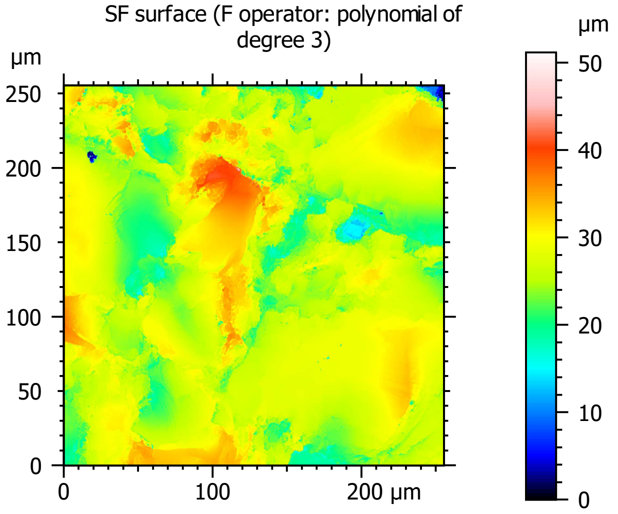

S-L surface (L filter: Gaussian, L nesting index = 127 µm, manage end effects)

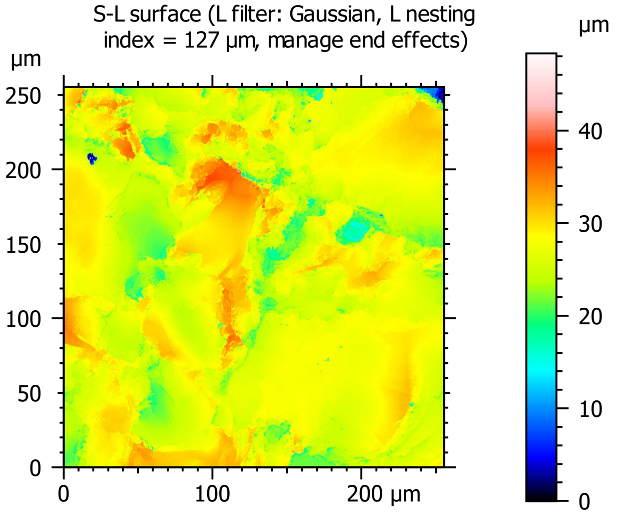

S-L surface (L filter: Gaussian, L nesting index = 127 µm, manage end effects)

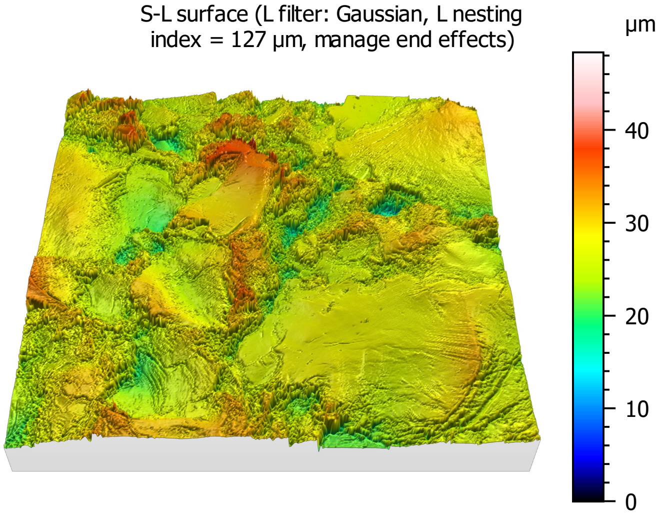

ISO 25178		
**Height Parameters**		
Sq	3.08	µm
Ssk	-0.811	
Sku	8.11	
Sp	21.4	µm
Sv	27.0	µm
Sz	48.3	µm
Sa	2.27	µm
**Functional Parameters**		
Smr	2.22e-05	%
Smc	3.49	µm
Sxp	6.45	µm
**Spatial Parameters**		
Sal	15.6	µm
Str	0.757	
Std	69.0	°
**Hybrid Parameters**		
Sdq	4.06	
Sdr	122	%
**Functional Parameters (Vol...**		
Vm	0.160	µm³/µm²
Vv	3.65	µm³/µm²
Vmp	0.160	µm³/µm²
Vmc	2.44	µm³/µm²
Vvc	3.22	µm³/µm²
Vvv	0.436	µm³/µm²
**Functional Parameters (Str...**		
Sk	4.06	µm
Spk	2.80	µm
Svk	3.61	µm
Smr1	13.6	%
Smr2	84.2	%
Spq	2.48	
Svq	17.3	
Smq	99.3	

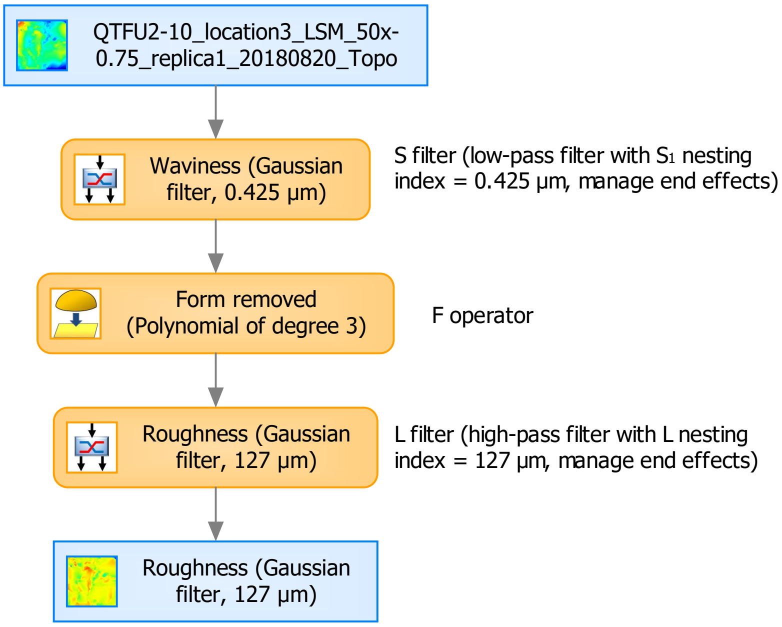

Quartzite QTFU2-10
50x objective, NA = 0.75 or 0.95

Identity card - QTFU2-10_location3_LSM_5...		
Name:	QTFU2-10_location3_LSM_50x-0.75_replica2_20...	
Created on:	8/20/2018 2:44:00 PM	
**Axis:**	**X**	
Length:	255	µm
Size:	3000	points
Spacing:	85.2	nm
**Axis:**	**Y**	
Length:	255	µm
Size:	3000	points
Spacing:	85.2	nm
**Axis:**	**Z**	
Length:	64.1	µm
Size:	65531	digits
Spacing:	0.978	nm
NMP ratio:	0.00 % (0 Pts)	

Extracted surface

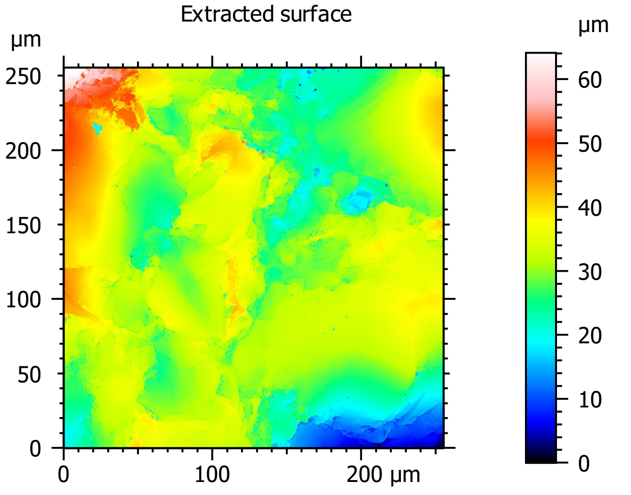

Primary surface (S Filter: Gaussian, S1 nesting index = 0.425 µm, manage end effects)

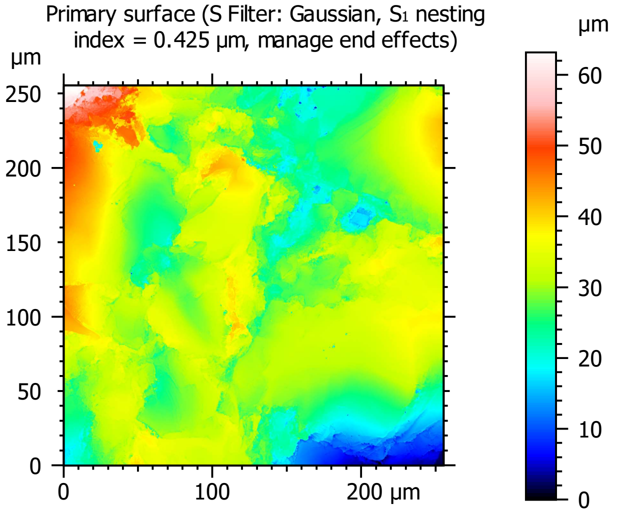

SF surface (F operator: polynomial of degree 3)

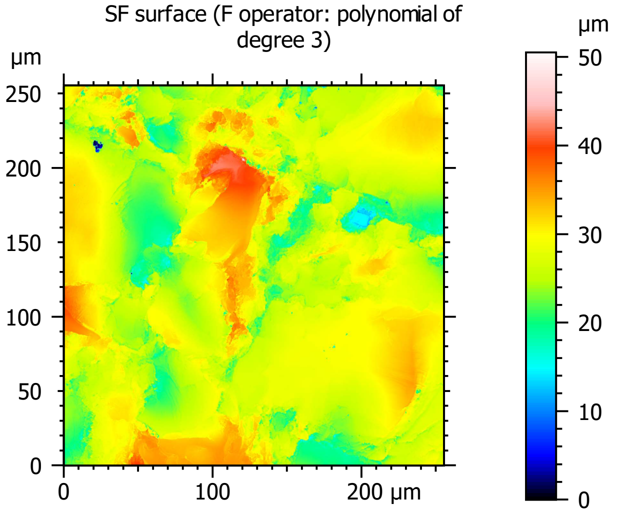

S-L surface (L filter: Gaussian, L nesting index = 127 µm, manage end effects)

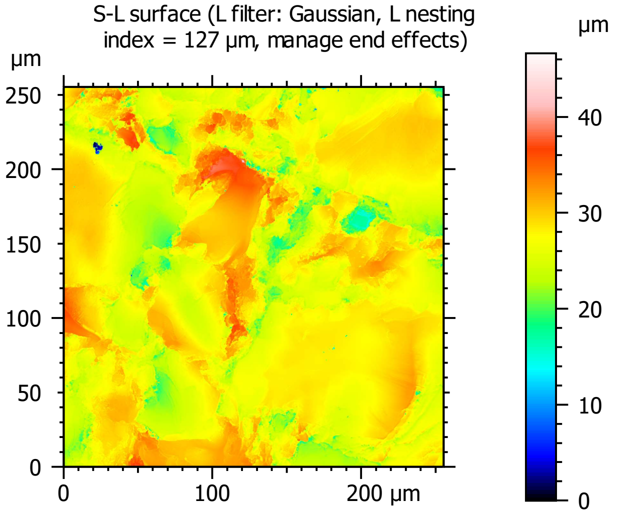

S-L surface (L filter: Gaussian, L nesting index = 127 µm, manage end effects)

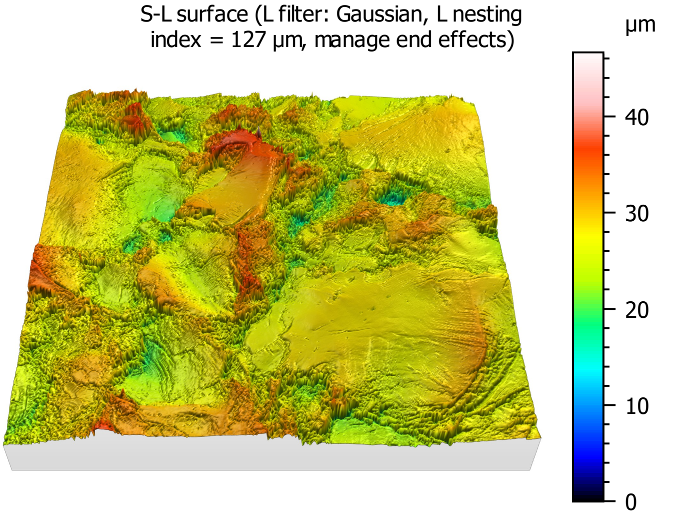

ISO 25178		
**Height Parameters**		
Sq	3.09	µm
Ssk	-0.321	
Sku	5.38	
Sp	19.4	µm
Sv	27.3	µm
Sz	46.6	µm
Sa	2.35	µm
**Functional Parameters**		
Smr	0.000167	%
Smc	3.48	µm
Sxp	6.54	µm
**Spatial Parameters**		
Sal	16.5	µm
Str	0.704	
Std	73.0	°
**Hybrid Parameters**		
Sdq	4.14	
Sdr	127	%
**Functional Parameters (Vol...**		
Vm	0.185	µm³/µm²
Vv	3.66	µm³/µm²
Vmp	0.185	µm³/µm²
Vmc	2.55	µm³/µm²
Vvc	3.25	µm³/µm²
Vvv	0.411	µm³/µm²
**Functional Parameters (Str...**		
Sk	4.17	µm
Spk	3.03	µm
Svk	3.43	µm
Smr1	12.5	%
Smr2	84.4	%
Spq	*****	
Svq	*****	
Smq	*****	

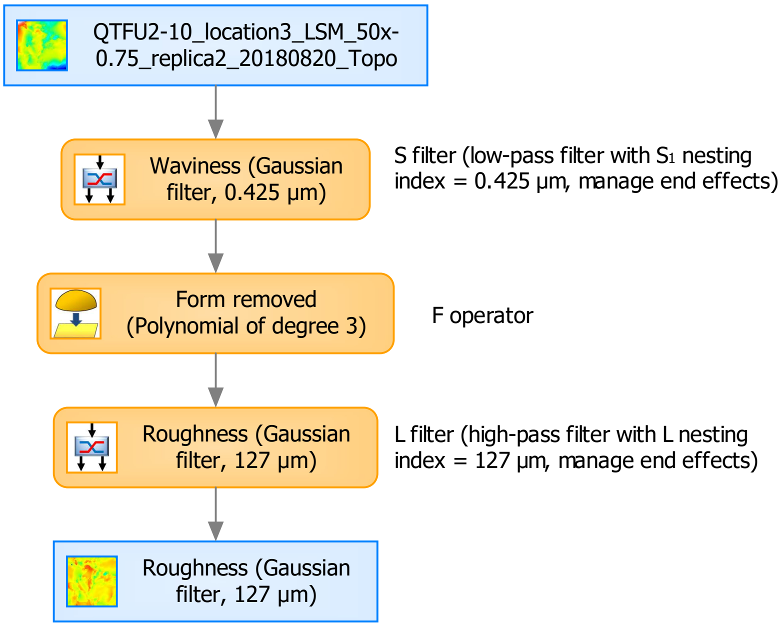

Quartzite QTFU2-10
50x objective, NA = 0.75 or 0.95

Identity card - QTFU2-10_location3_LSM_5...		
Name:	QTFU2-10_location3_LSM_50x-0.75_replica3_20...	
Created on:	8/20/2018 3:26:18 PM	
**Axis:**	**X**	
Length:	255	µm
Size:	3000	points
Spacing:	85.2	nm
**Axis:**	**Y**	
Length:	255	µm
Size:	3000	points
Spacing:	85.2	nm
**Axis:**	**Z**	
Length:	63.4	µm
Size:	65532	digits
Spacing:	0.967	nm
NMP ratio:	0.00 % (0 Pts)	

Extracted surface

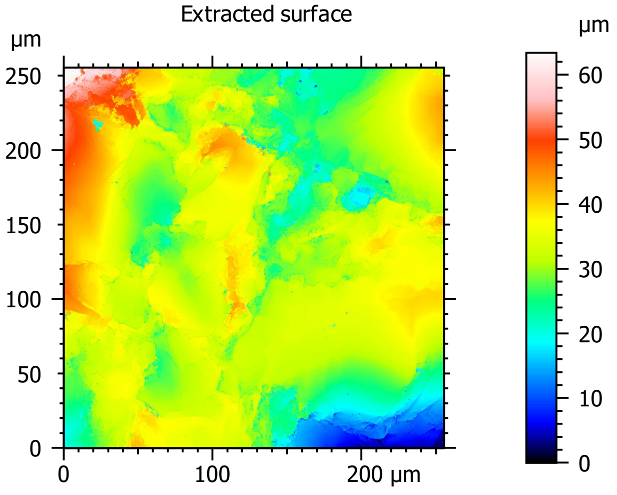

Primary surface (S Filter: Gaussian, S1 nesting index = 0.425 µm, manage end effects)

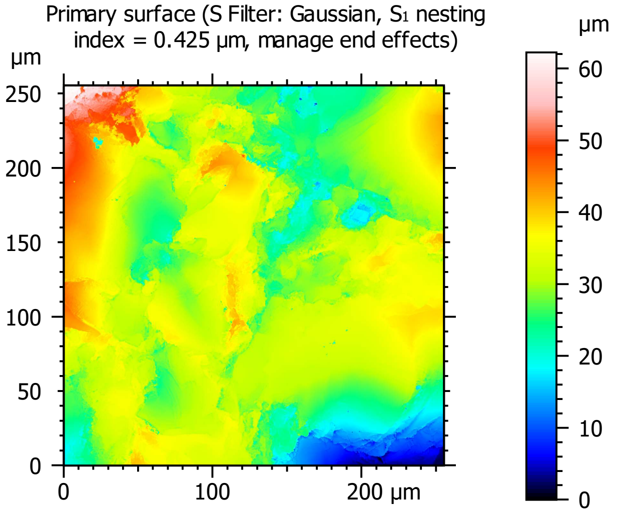

SF surface (F operator: polynomial of degree 3)

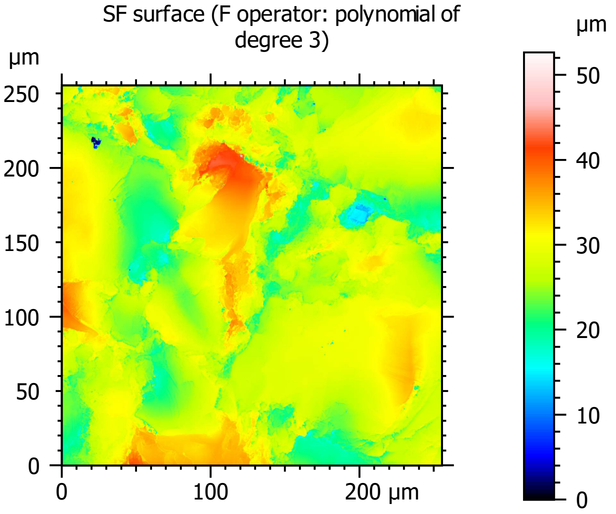

S-L surface (L filter: Gaussian, L nesting index = 127 µm, manage end effects)

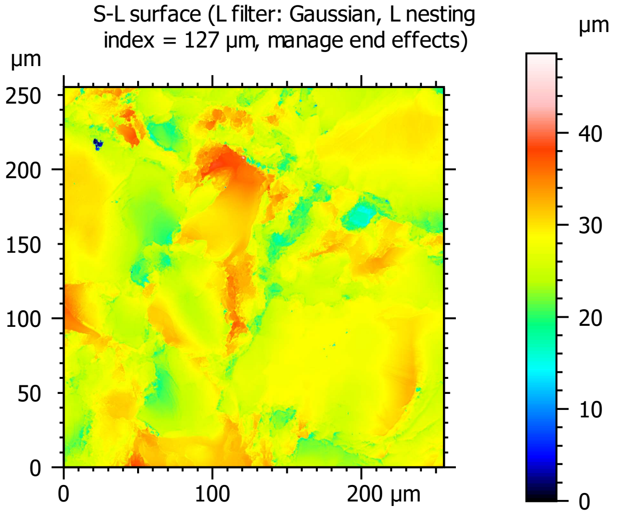

S-L surface (L filter: Gaussian, L nesting index = 127 µm, manage end effects)

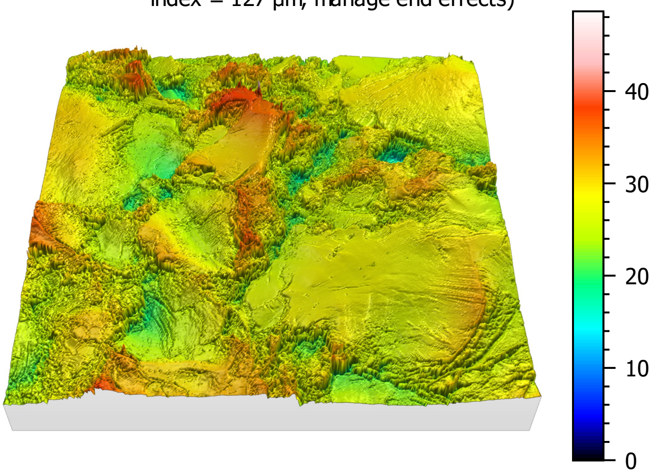

ISO 25178		
**Height Parameters**		
Sq	3.14	µm
Ssk	-0.283	
Sku	5.17	
Sp	21.3	µm
Sv	27.3	µm
Sz	48.6	µm
Sa	2.39	µm
**Functional Parameters**		
Smr	0.0001	%
Smc	3.53	µm
Sxp	6.64	µm
**Spatial Parameters**		
Sal	16.6	µm
Str	0.668	
Std	69.2	°
**Hybrid Parameters**		
Sdq	4.17	
Sdr	128	%
**Functional Parameters (V...**		
Vm	0.188	µm³/µm²
Vv	3.72	µm³/µm²
Vmp	0.188	µm³/µm²
Vmc	2.60	µm³/µm²
Vvc	3.30	µm³/µm²
Vvv	0.413	µm³/µm²
**Functional Parameters (S...**		
Sk	4.20	µm
Spk	3.08	µm
Svk	3.42	µm
Smr1	12.8	%
Smr2	84.0	%
Spq	*****	
Svq	*****	
Smq	*****	

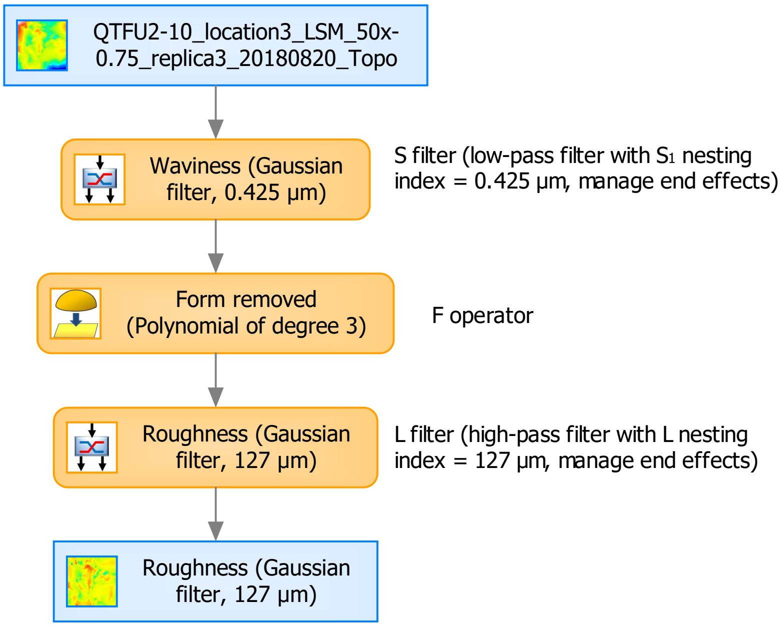

Quartzite QTFU2-10
50x objective, NA = 0.75 or 0.95

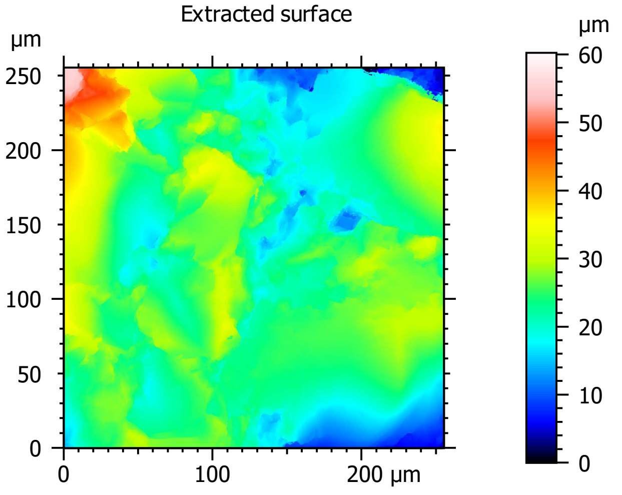

Identity card - QTFU2-10_location3_LSM_5...		
Name:	QTFU2-10_location3_LSM_50x-0.95_replica1_20...	
Created on:	8/20/2018 1:39:43 PM	
**Axis:**	**X**	
Length:	255	µm
Size:	3000	points
Spacing:	85.2	nm
**Axis:**	**Y**	
Length:	255	µm
Size:	3000	points
Spacing:	85.2	nm
**Axis:**	**Z**	
Length:	60.2	µm
Size:	65532	digits
Spacing:	0.919	nm
NMP ratio:	0.00 % (0 Pts)	

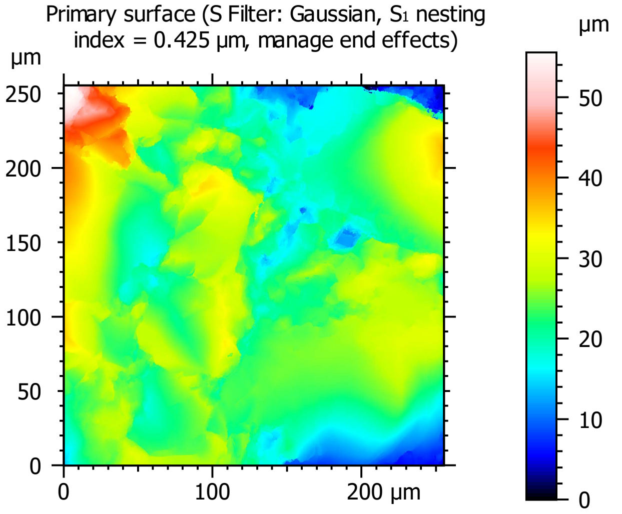

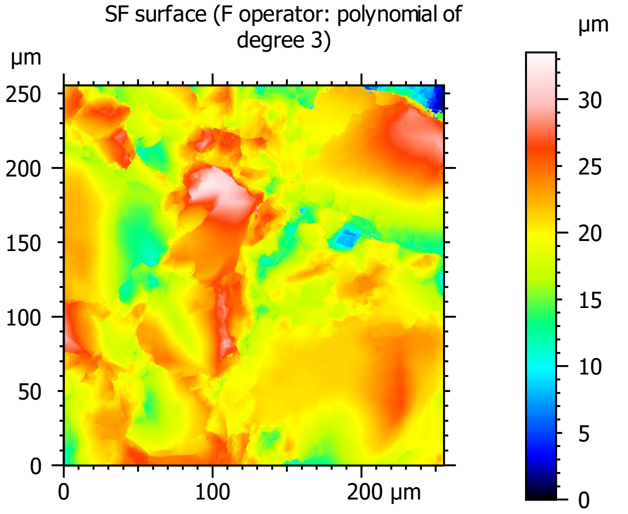

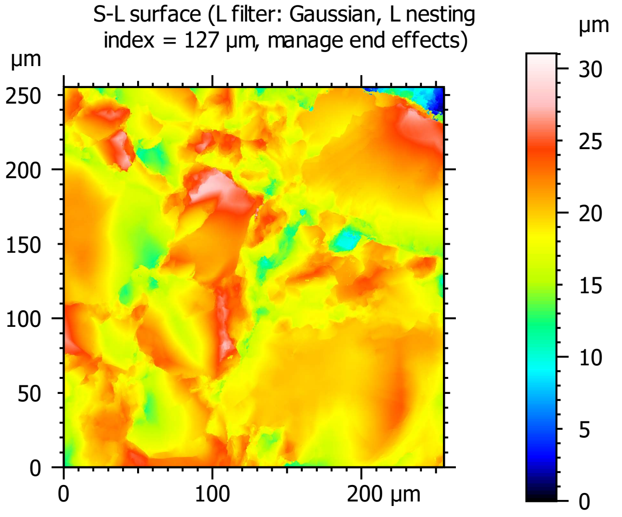

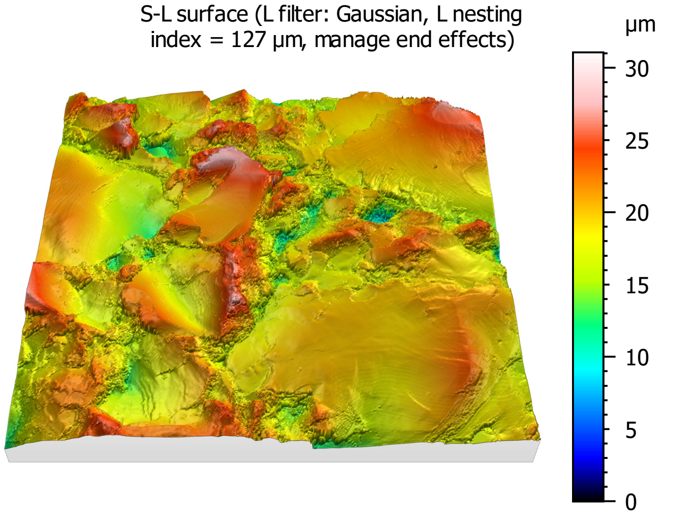

ISO 25178		
**Height Parameters**		
Sq	2.85	µm
Ssk	-0.656	
Sku	6.21	
Sp	11.7	µm
Sv	19.3	µm
Sz	31.1	µm
Sa	2.10	µm
**Functional Parameters**		
Smr	0.000256	%
Smc	3.32	µm
Sxp	5.95	µm
**Spatial Parameters**		
Sal	15.0	µm
Str	0.740	
Std	73.0	°
**Hybrid Parameters**		
Sdq	2.09	
Sdr	39.0	%
**Functional Parameters (Vol...**		
Vm	0.155	µm³/µm²
Vv	3.48	µm³/µm²
Vmp	0.155	µm³/µm²
Vmc	2.26	µm³/µm²
Vvc	3.08	µm³/µm²
Vvv	0.396	µm³/µm²
**Functional Parameters (Str...**		
Sk	3.61	µm
Spk	2.66	µm
Svk	3.08	µm
Smr1	14.9	%
Smr2	84.3	%
Spq	*****	
Svq	*****	
Smq	*****	

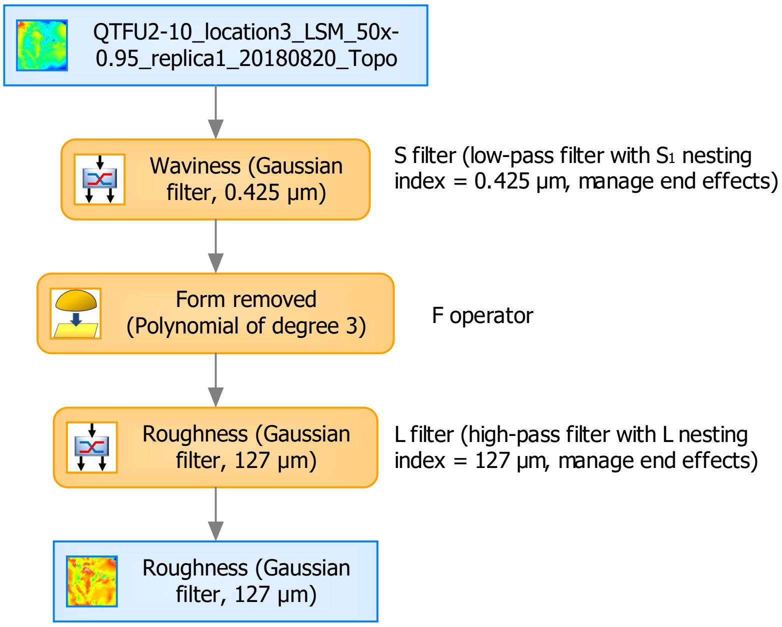

Quartzite QTFU2-10
50x objective, NA = 0.75 or 0.95

**Identity card - QTFU2-10_location3_LSM_5...**		
Name:	QTFU2-10_location3_LSM_50x-0.95_replica2_20...	
Created on:	8/20/2018 2:23:07 PM	
**Axis:**	**X**	
Length:	255	µm
Size:	3000	points
Spacing:	85.2	nm
**Axis:**	**Y**	
Length:	255	µm
Size:	3000	points
Spacing:	85.2	nm
**Axis:**	**Z**	
Length:	70.7	µm
Size:	65532	digits
Spacing:	1.08	nm
NMP ratio:	0.00 % (0 Pts)	

Extracted surface

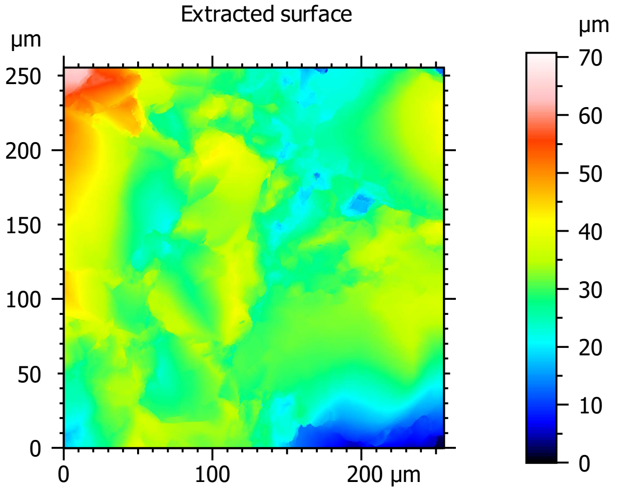

Primary surface (S Filter: Gaussian, S1 nesting index = 0.425 µm, manage end effects)

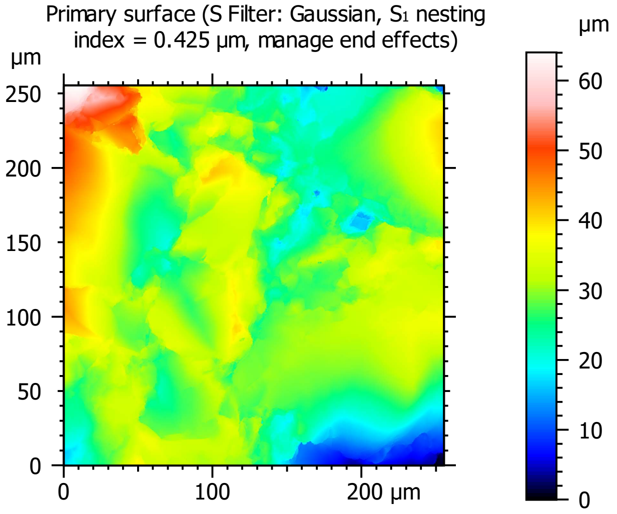

SF surface (F operator: polynomial of degree 3)

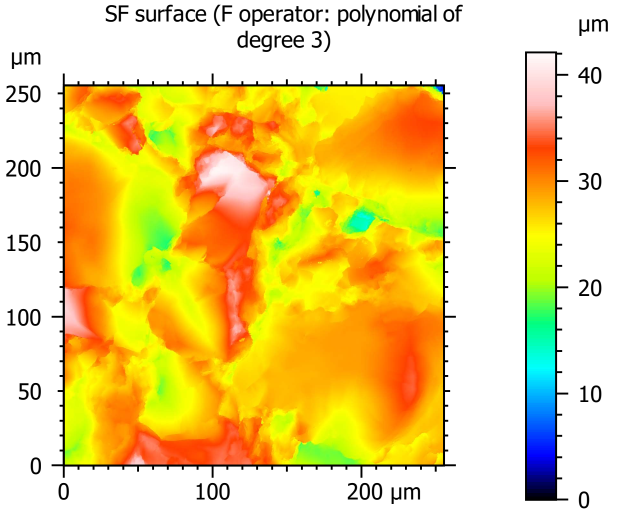

S-L surface (L filter: Gaussian, L nesting index = 127 µm, manage end effects)

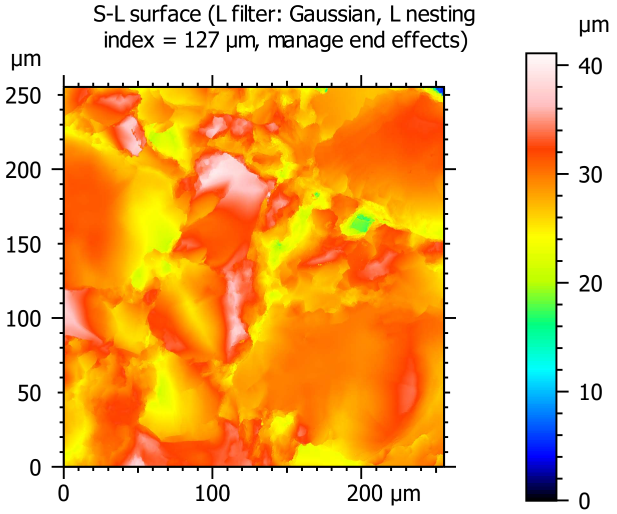

S-L surface (L filter: Gaussian, L nesting index = 127 µm, manage end effects)

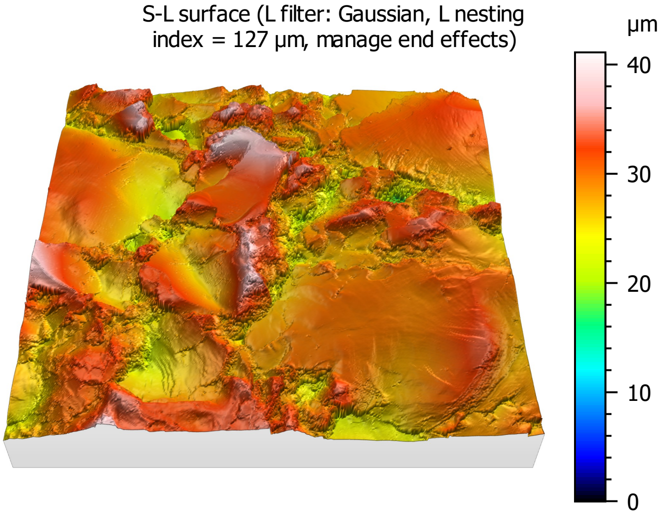

ISO 25178		
**Height Parameters**		
Sq	2.91	µm
Ssk	-0.25	
Sku	5.22	
Sp	12.0	µm
Sv	29.2	µm
Sz	41.1	µm
Sa	2.22	µm
**Functional Parameters**		
Smr	0.000137	%
Smc	3.35	µm
Sxp	6.02	µm
**Spatial Parameters**		
Sal	16.7	µm
Str	0.645	
Std	51.5	°
**Hybrid Parameters**		
Sdq	1.91	
Sdr	40.7	%
**Functional Parameters (Vol...**		
Vm	0.176	µm³/µm²
Vv	3.52	µm³/µm²
Vmp	0.176	µm³/µm²
Vmc	2.42	µm³/µm²
Vvc	3.15	µm³/µm²
Vvv	0.372	µm³/µm²
**Functional Parameters (Str...**		
Sk	3.92	µm
Spk	2.73	µm
Svk	2.83	µm
Smr1	13.0	%
Smr2	84.0	%
Spq	*****	
Svq	*****	
Smq	*****	

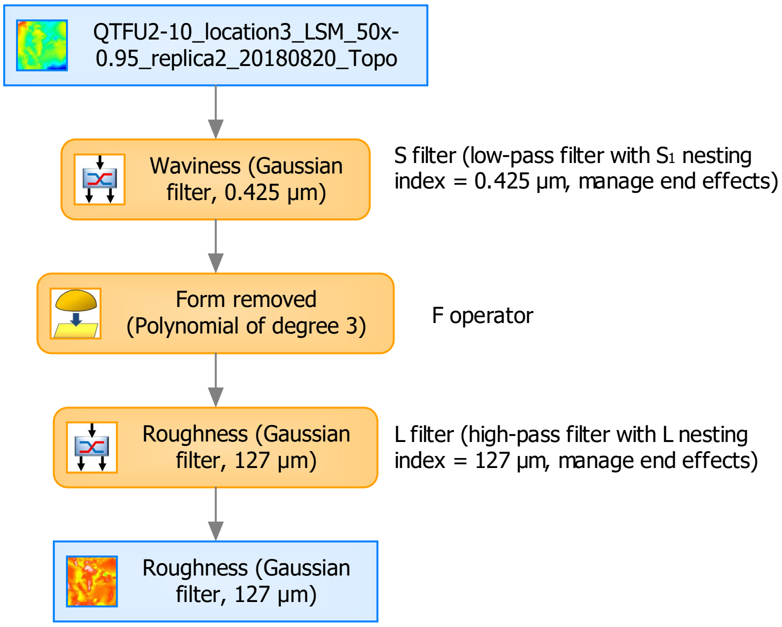

Quartzite QTFU2-10
50x objective, NA = 0.75 or 0.95

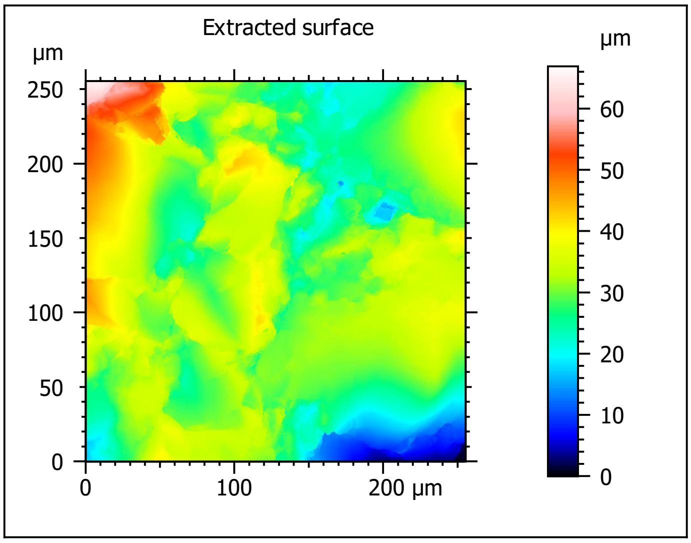

Identity card - QTFU2-10_location3_LSM_5...		
Name:	QTFU2-10_location3_LSM_50x-0.95_replica3_20...	
Created on:	8/20/2018 3:06:11 PM	
**Axis:**	**X**	
Length:	255	µm
Size:	3000	points
Spacing:	85.2	nm
**Axis:**	**Y**	
Length:	255	µm
Size:	3000	points
Spacing:	85.2	nm
**Axis:**	**Z**	
Length:	66.9	µm
Size:	65532	digits
Spacing:	1.02	nm
NMP ratio:	0.00 % (0 Pts)	

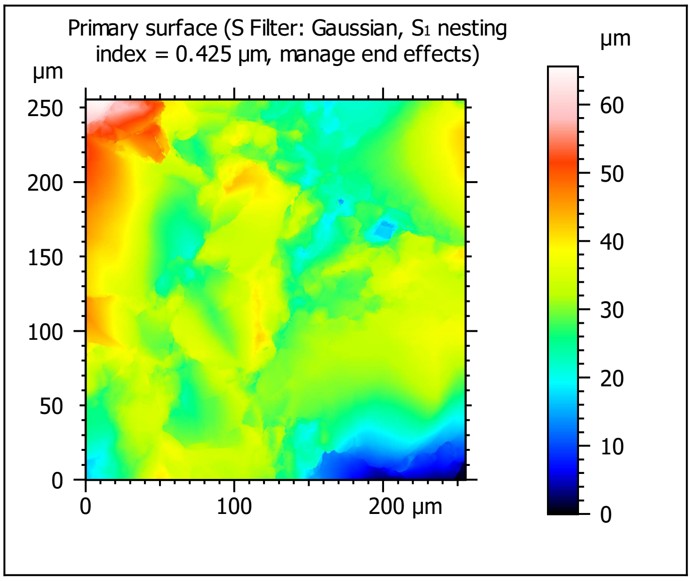

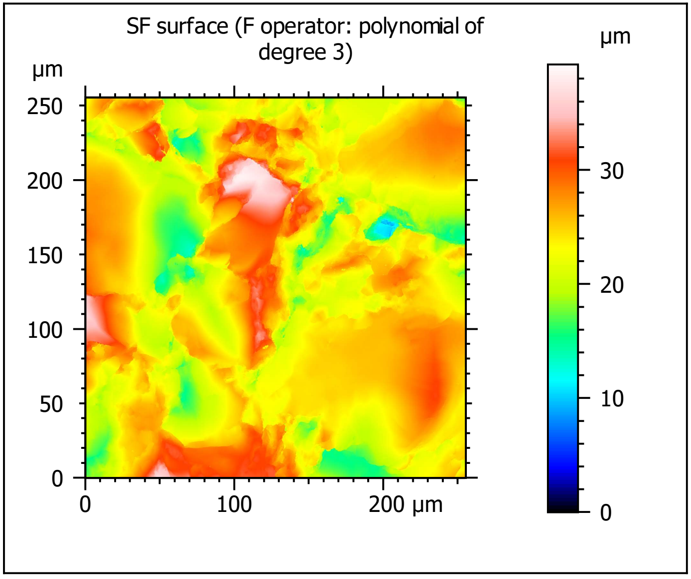

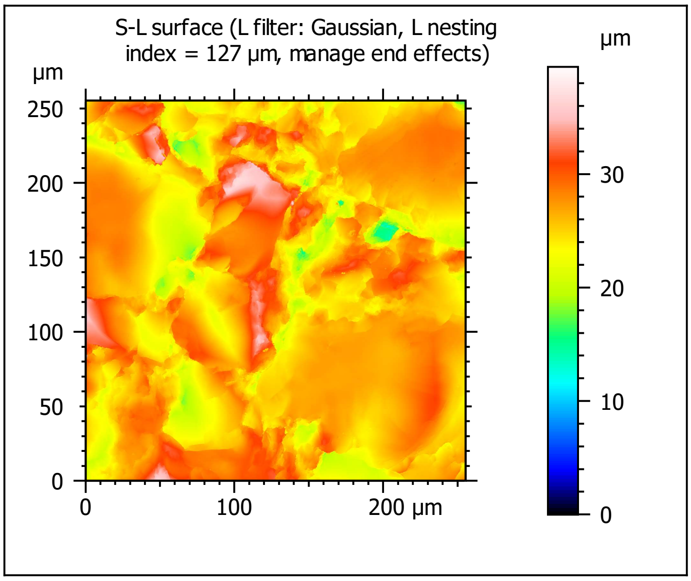

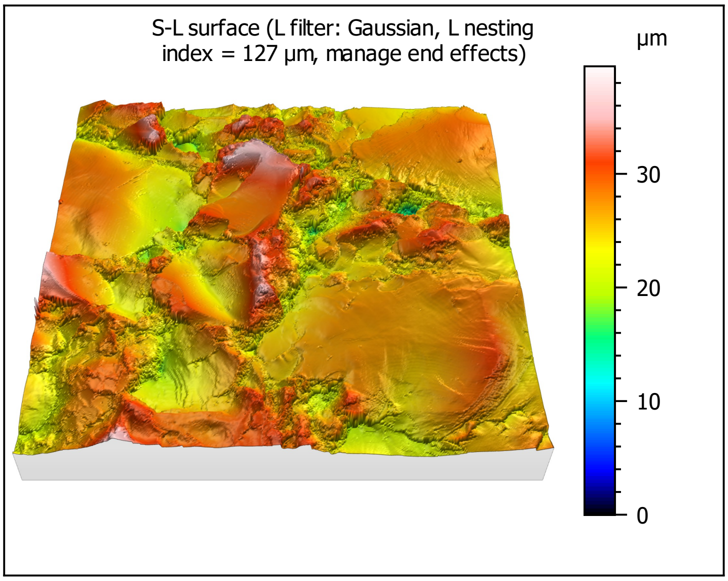

ISO 25178		
**Height Parameters**		
Sq	2.94	µm
Ssk	-0.0327	
Sku	3.88	
Sp	13.2	µm
Sv	26.2	µm
Sz	39.5	µm
Sa	2.27	µm
**Functional Parameters**		
Smr	0.00224	%
Smc	3.42	µm
Sxp	6.10	µm
**Spatial Parameters**		
Sal	17.0	µm
Str	0.595	
Std	51.5	°
**Hybrid Parameters**		
Sdq	1.89	
Sdr	41.1	%
**Functional Parameters (Vol...**		
Vm	0.183	µm³/µm²
Vv	3.61	µm³/µm²
Vmp	0.183	µm³/µm²
Vmc	2.51	µm³/µm²
Vvc	3.24	µm³/µm²
Vvv	0.365	µm³/µm²
**Functional Parameters (Str...**		
Sk	3.96	µm
Spk	2.85	µm
Svk	2.86	µm
Smr1	13.0	%
Smr2	84.0	%
Spq	*****	
Svq	*****	
Smq	*****	

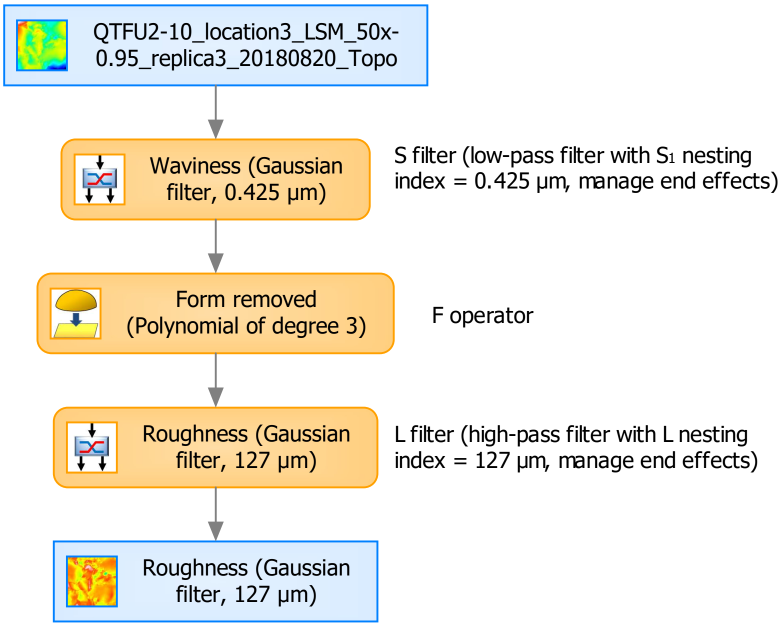

Roughness standard *Ra* = 0.4 um
50x objective, NA = 0.75 or 0.95, 4x1 tiles

Extracted

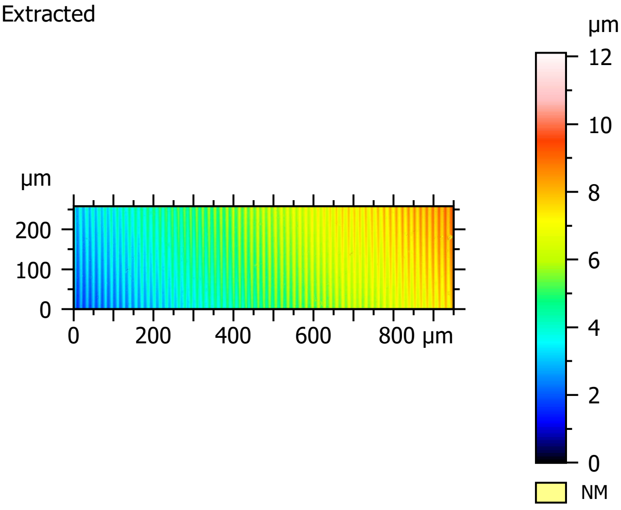

**Identity card - roughnessStandard0.4um_locat...**		
Name:	roughnessStandard0.4um_location1_LSM_50x-0.75_...	
Created on:	8/15/2018 12:52:07 PM	
**Axis:**	**X**	
Length:	950	µm
Size:	7617	points
Spacing:	0.125	µm
**Axis:**	**Y**	
Length:	258	µm
Size:	2069	points
Spacing:	0.125	µm
**Axis:**	**Z**	
Length:	12.1	µm
Size:	65532	digits
Spacing:	0.185	nm
NMP ratio:	0.989 % (155932 Pts)	

Levelled surface (least square method by subtraction)

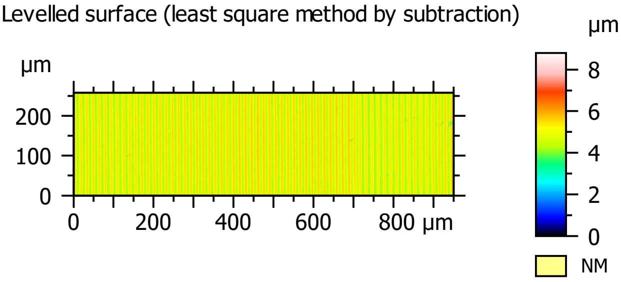

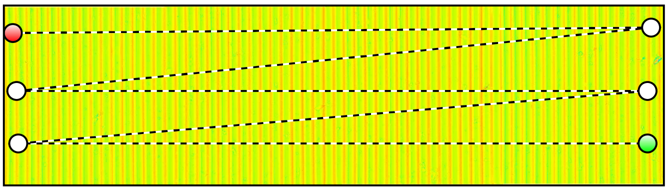

Extracted profile

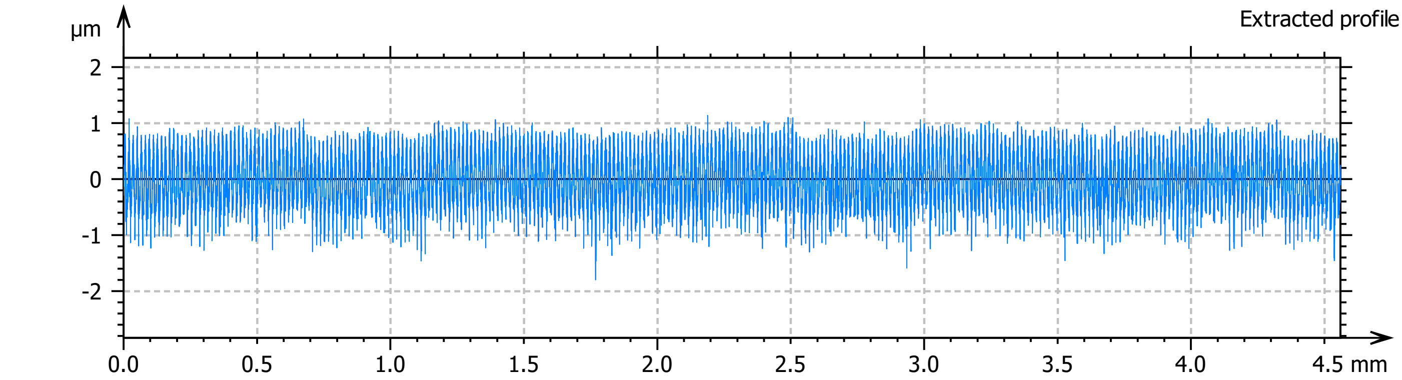

Extracted profile

**Parameters**	Value	Unit
Length	4.56	mm

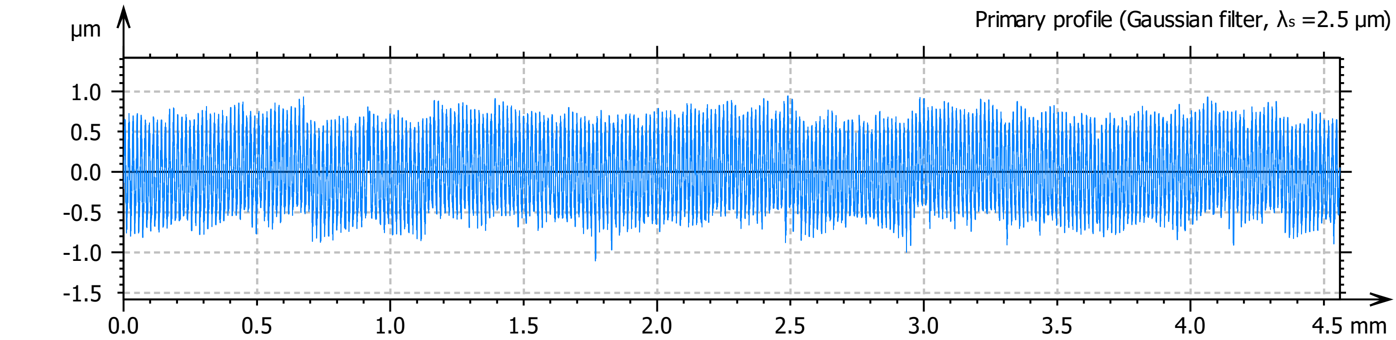

Primary profile (Gaussian filter, λ_s =2.5 µm)

**Parameters**	Value	Unit
Length	4.56	mm

ISO 4287				
**Amplitude parameters - Roughness profile**				
Ra	0.396	µm	*Gaussian filter, 0.8 mm*	*Arithmetic mean deviation of the roughness profile.*

**Parameters**	Value	Unit	Tolerance	
Ra - Amplitude parameters - Roughness profile - ISO 4287	0.396	µm	0.400 µm ± 0.050 µm	✓

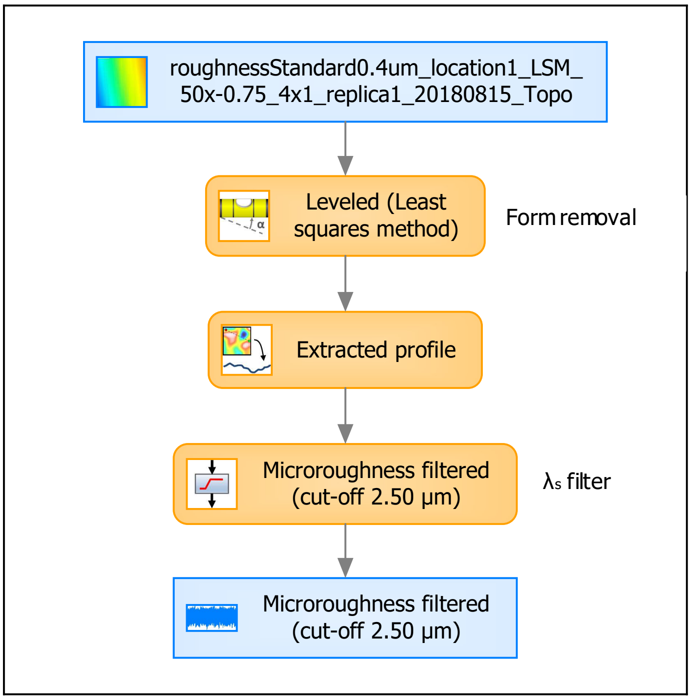

Roughness standard *Ra* = 0.4 um
50x objective, NA = 0.75 or 0.95, 4x1 tiles

Identity card - roughnessStandard0.4um_locat...

Name:	roughnessStandard0.4um_location1_LSM_50x-0.75_...	
Created on:	8/15/2018 1:11:25 PM	
**Axis:**	**X**	
Length:	950	µm
Size:	7617	points
Spacing:	0.125	µm
**Axis:**	**Y**	
Length:	258	µm
Size:	2069	points
Spacing:	0.125	µm
**Axis:**	**Z**	
Length:	11.9	µm
Size:	65532	digits
Spacing:	0.181	nm
NMP ratio:	0.989 % (155932 Pts)	

Extracted

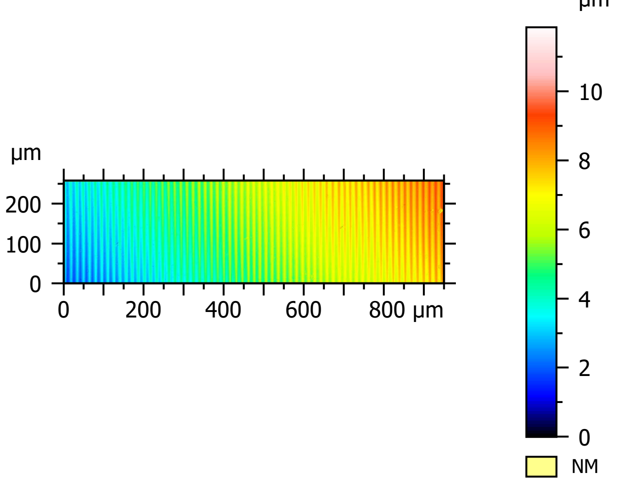

Levelled surface (least square method by subtraction)

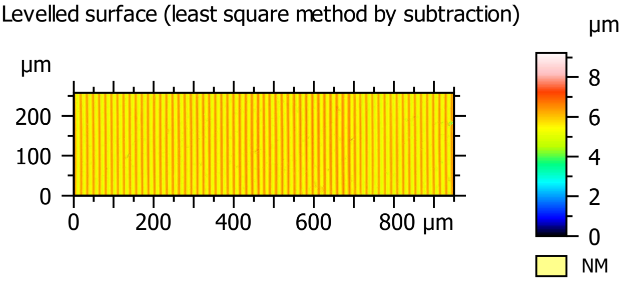

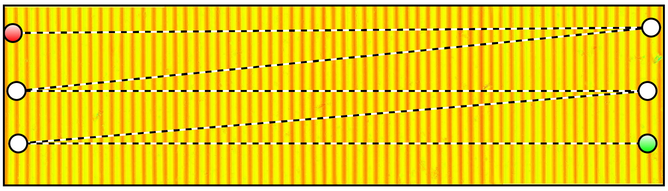

Extracted profile

Extracted profile

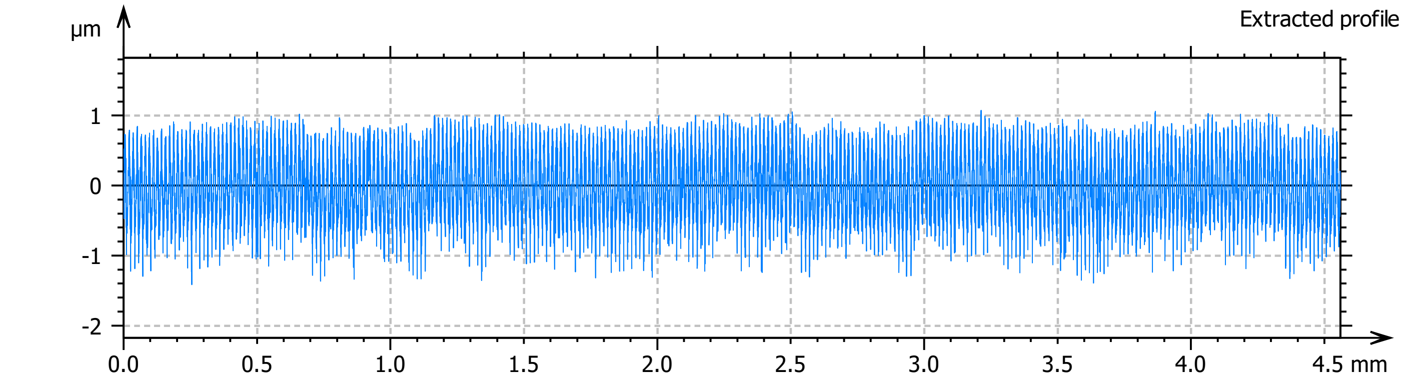

**Parameters**	Value	Unit
Length	4.56	mm

Primary profile (Gaussian filter, λ_s =2.5 µm)

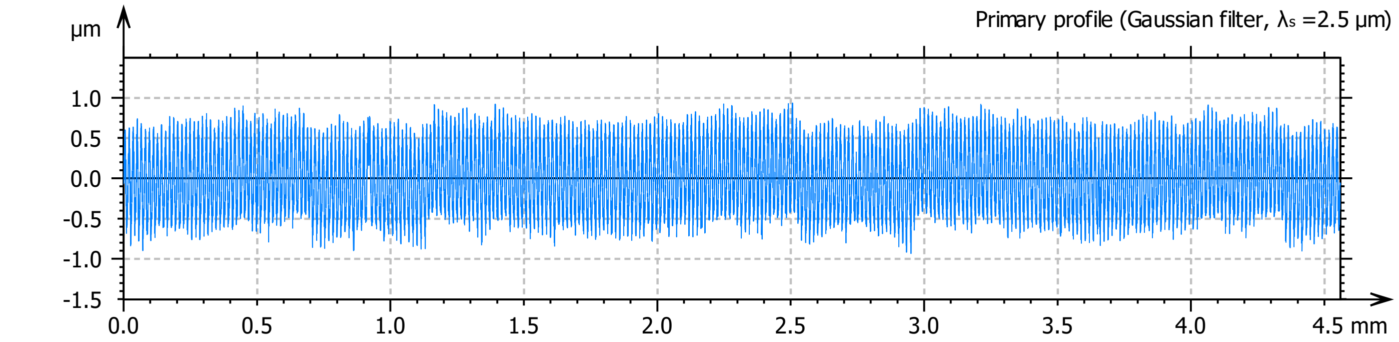

**Parameters**	Value	Unit
Length	4.56	mm

ISO 4287				
**Amplitude parameters - Roughness profile**				
Ra	0.395	µm	*Gaussian filter, 0.8 mm*	*Arithmetic mean deviation of the roughness profile.*

**Parameters**	Value	Unit	Tolerance	
Ra - Amplitude parameters - Roughness profile - ISO 4287	0.395	µm	0.400 µm ± 0.050 µm	✓

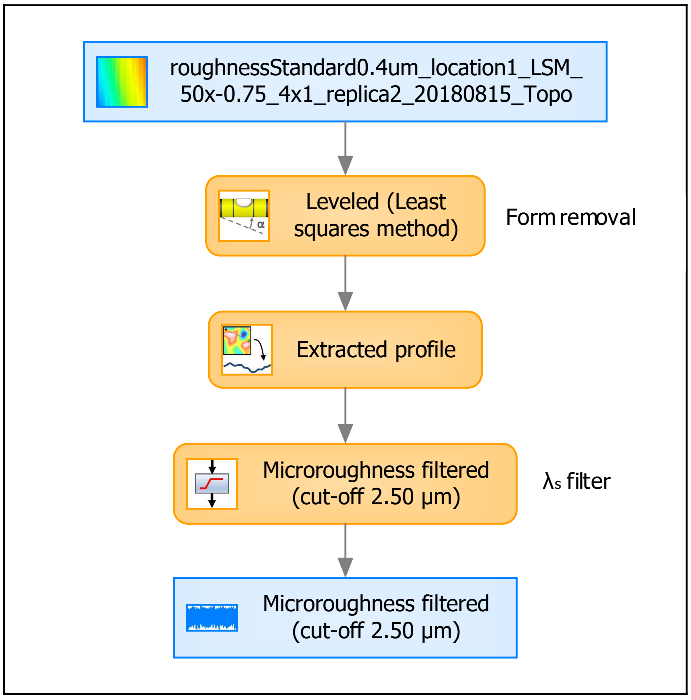

Roughness standard *Ra* = 0.4 um
50x objective, NA = 0.75 or 0.95, 4x1 tiles

Extracted

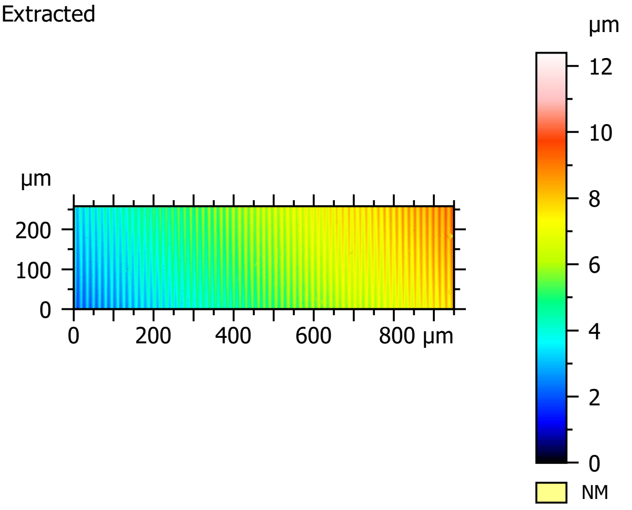

Identity card - roughnessStandard0.4um_locat...		
Name:	roughnessStandard0.4um_location1_LSM_50x-0.75_...	
Created on:	8/15/2018 1:30:08 PM	
**Axis:**	**X**	
Length:	950	µm
Size:	7617	points
Spacing:	0.125	µm
**Axis:**	**Y**	
Length:	258	µm
Size:	2069	points
Spacing:	0.125	µm
**Axis:**	**Z**	
Length:	12.4	µm
Size:	65532	digits
Spacing:	0.189	nm
NMP ratio:	0.989 % (155932 Pts)	

Levelled surface (least square method by subtraction)

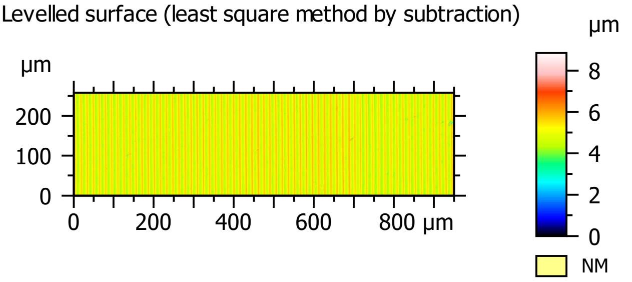

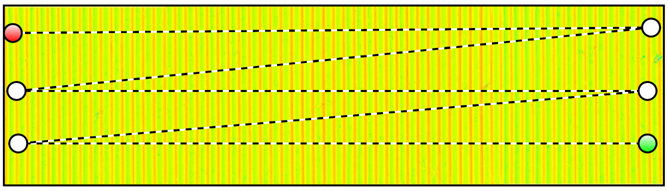

Extracted profile

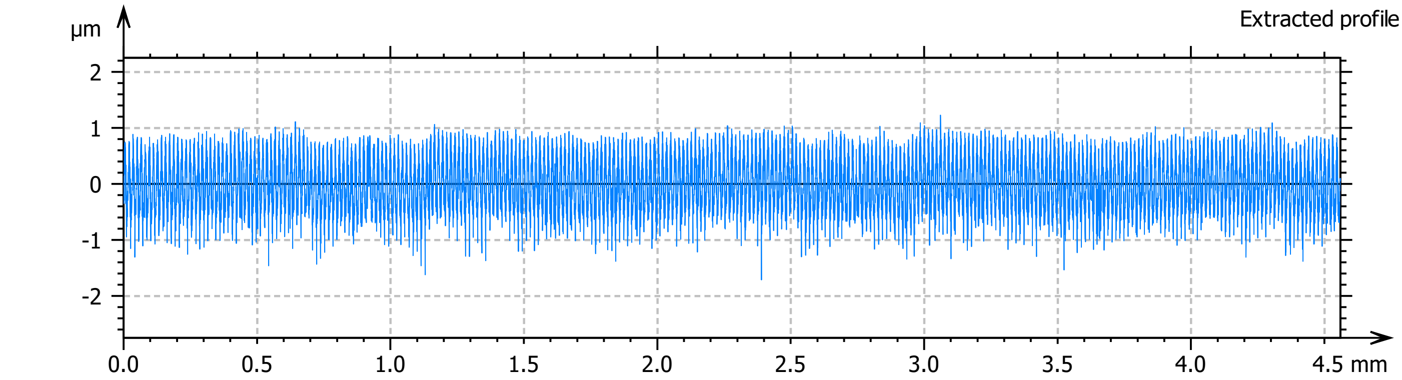

**Parameters**	Value	Unit
Length	4.56	mm

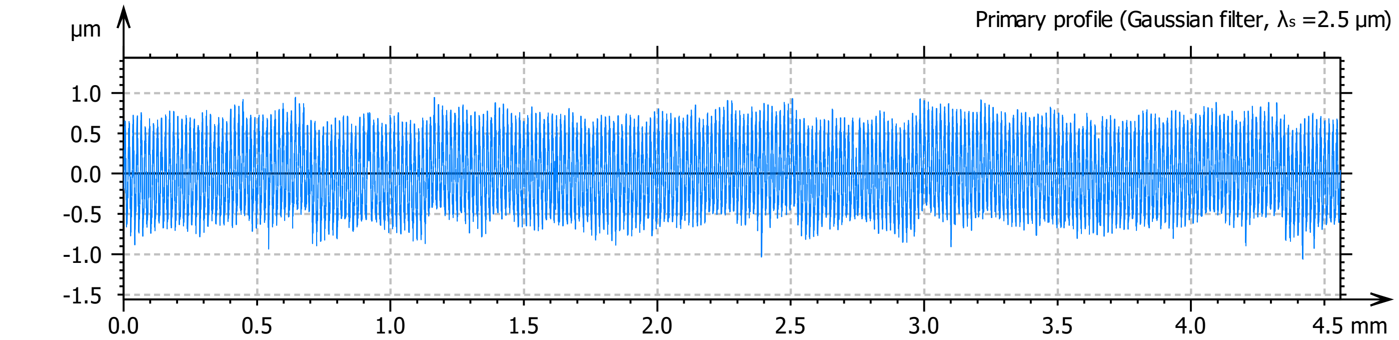

**Parameters**	Value	Unit
Length	4.56	mm

ISO 4287				
**Amplitude parameters - Roughness profile**				
Ra	0.396	µm	*Gaussian filter, 0.8 mm*	*Arithmetic mean deviation of the roughness profile.*

**Parameters**	Value	Unit	Tolerance	
Ra - Amplitude parameters - Roughness profile - ISO 4287	0.396	µm	0.400 µm ± 0.050 µm	✓

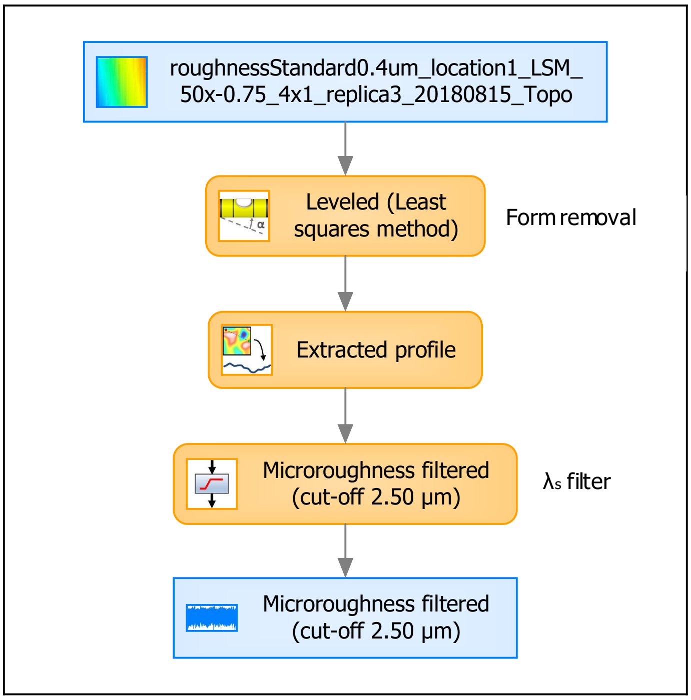

Roughness standard *Ra* = 0.4 um
50x objective, NA = 0.75 or 0.95, 4x1 tiles

**Identity card - roughnessStandard0.4um_locat...**		
Name:	roughnessStandard0.4um_location1_LSM_50x-0.95_...	
Created on:	8/15/2018 12:35:19 PM	
**Axis:**	**X**	
Length:	944	µm
Size:	7569	points
Spacing:	0.125	µm
**Axis:**	**Y**	
Length:	258	µm
Size:	2069	points
Spacing:	0.125	µm
**Axis:**	**Z**	
Length:	10.5	µm
Size:	65531	digits
Spacing:	0.160	nm
NMP ratio:	0.987 % (154588 Pts)	

Extracted

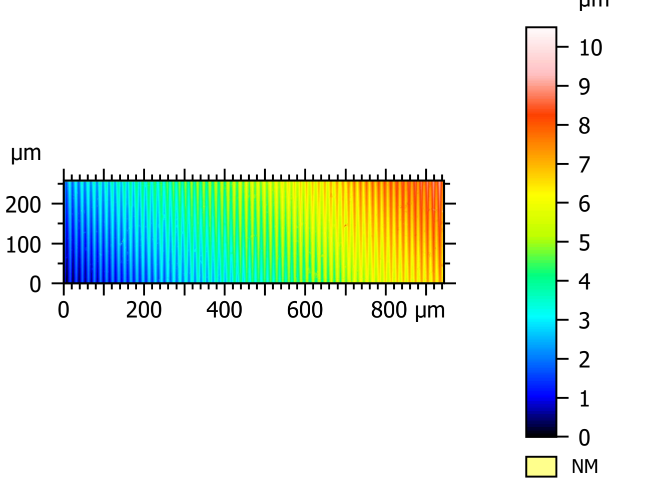

Levelled surface (least square method by subtraction)

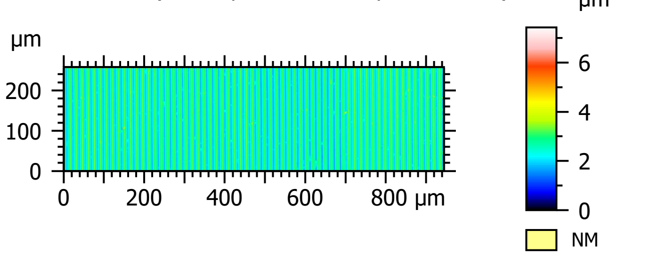

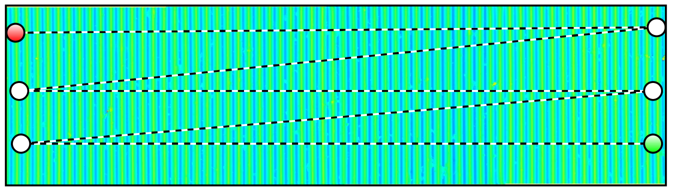

Extracted profile

Extracted profile

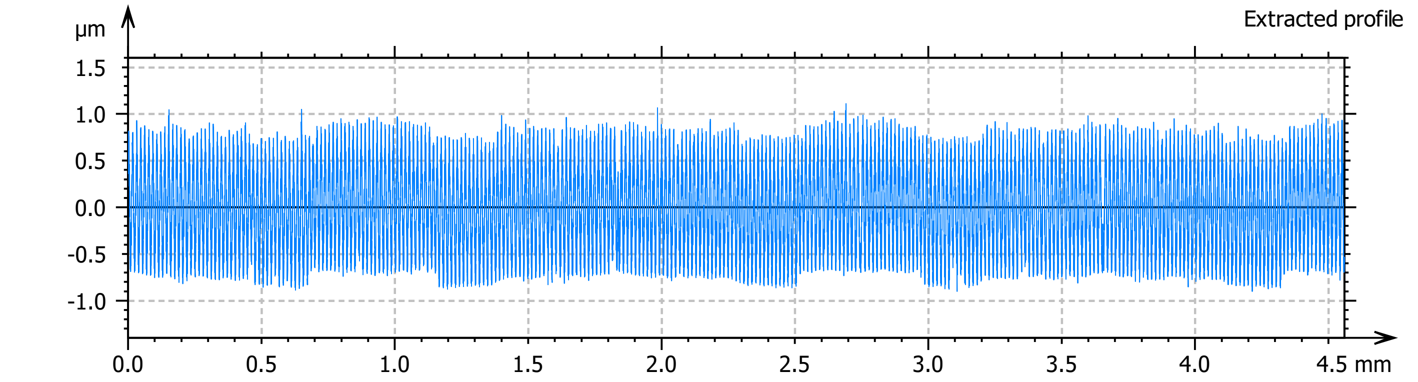

**Parameters**	Value	Unit
Length	4.56	mm

Primary profile (Gaussian filter, λ_s =2.5 µm)

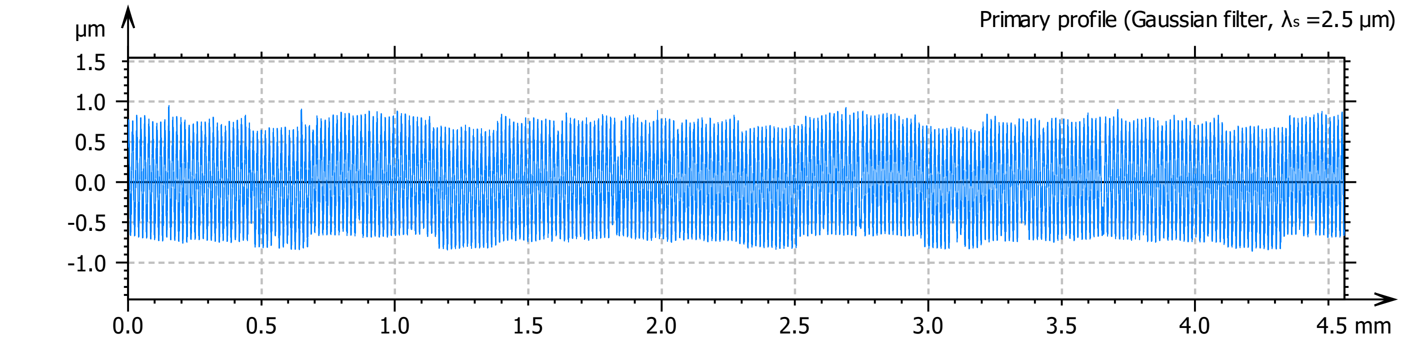

**Parameters**	Value	Unit
Length	4.56	mm

ISO 4287				
**Amplitude parameters - Roughness profile**				
Ra	0.418	µm	*Gaussian filter, 0.8 mm*	*Arithmetic mean deviation of the roughness profile.*

**Parameters**	Value	Unit	Tolerance	
Ra - Amplitude parameters - Roughness profile - ISO 4287	0.418	µm	0.400 µm ± 0.050 µm	✓

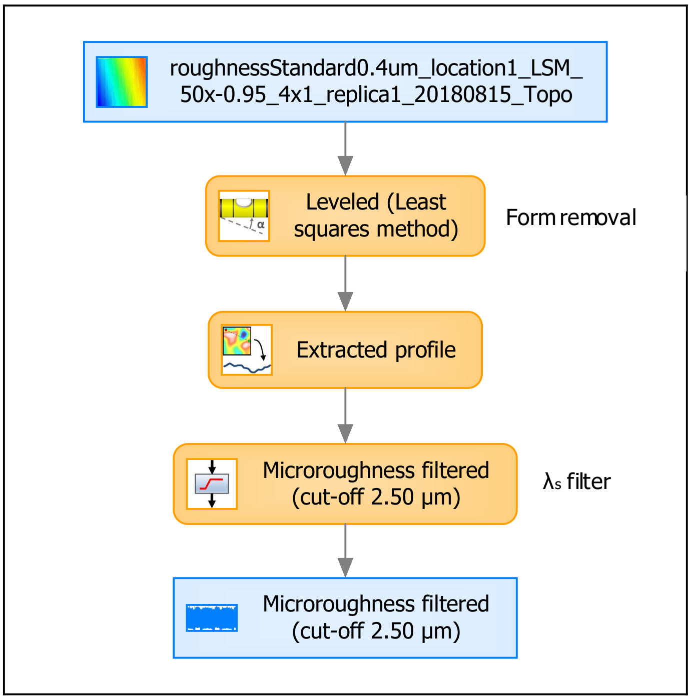

Roughness standard *Ra* = 0.4 um
50x objective, NA = 0.75 or 0.95, 4x1 tiles

**Identity card - roughnessStandard0.4um_locat...**		
Name:	roughnessStandard0.4um_location1_LSM_50x-0.95_...	
Created on:	8/15/2018 1:02:18 PM	
**Axis:**	**X**	
Length:	944	µm
Size:	7569	points
Spacing:	0.125	µm
**Axis:**	**Y**	
Length:	258	µm
Size:	2069	points
Spacing:	0.125	µm
**Axis:**	**Z**	
Length:	11.5	µm
Size:	65532	digits
Spacing:	0.176	nm
NMP ratio:	0.987 % (154588 Pts)	

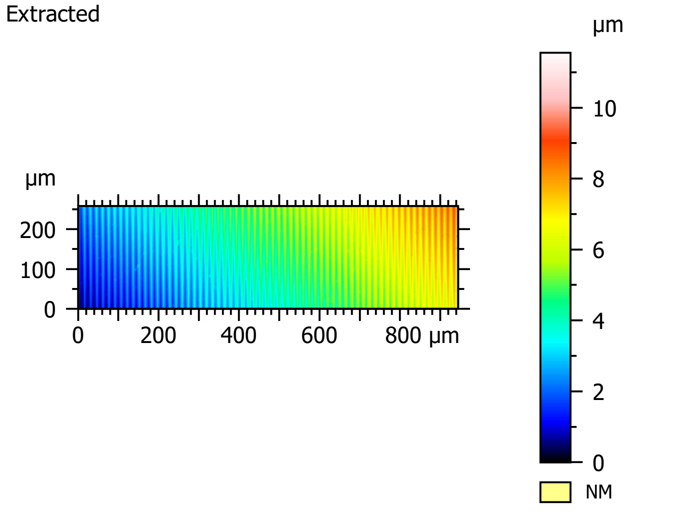

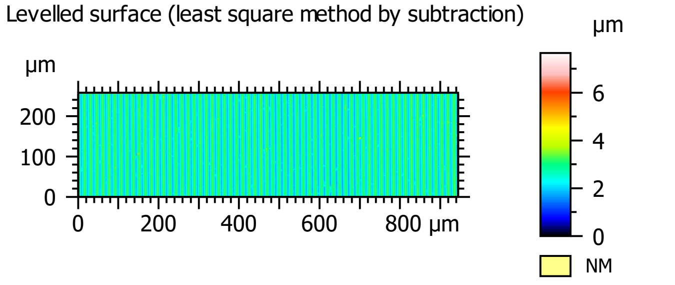

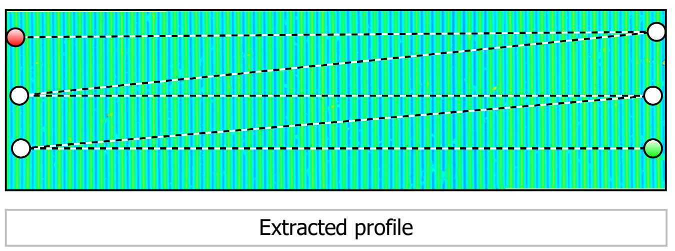

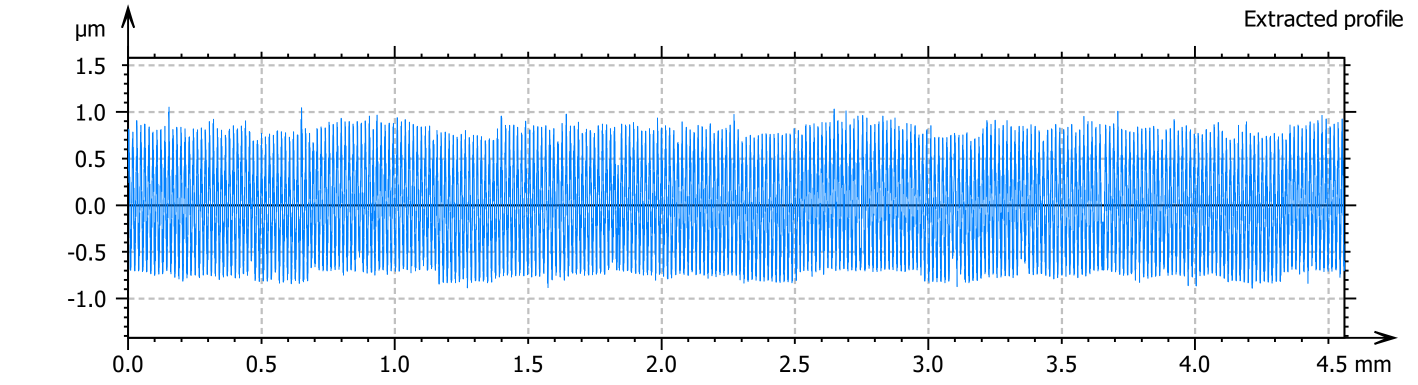

**Parameters**	Value	Unit
Length	4.56	mm

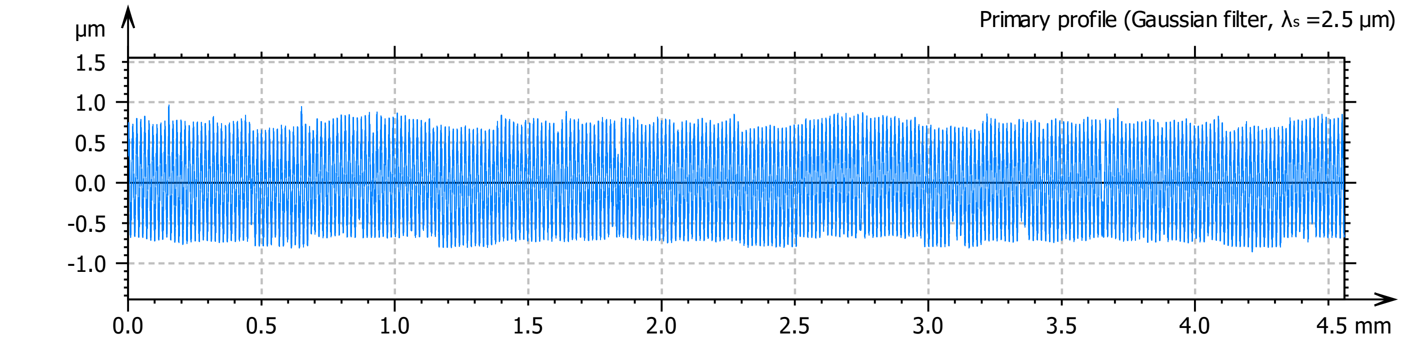

**Parameters**	Value	Unit
Length	4.56	mm

ISO 4287				
**Amplitude parameters - Roughness profile**				
Ra	0.415	µm	*Gaussian filter, 0.8 mm*	*Arithmetic mean deviation of the roughness profile.*

**Parameters**	Value	Unit	Tolerance	
Ra - Amplitude parameters - Roughness profile - ISO 4287	0.415	µm	0.400 µm ± 0.050 µm	✓

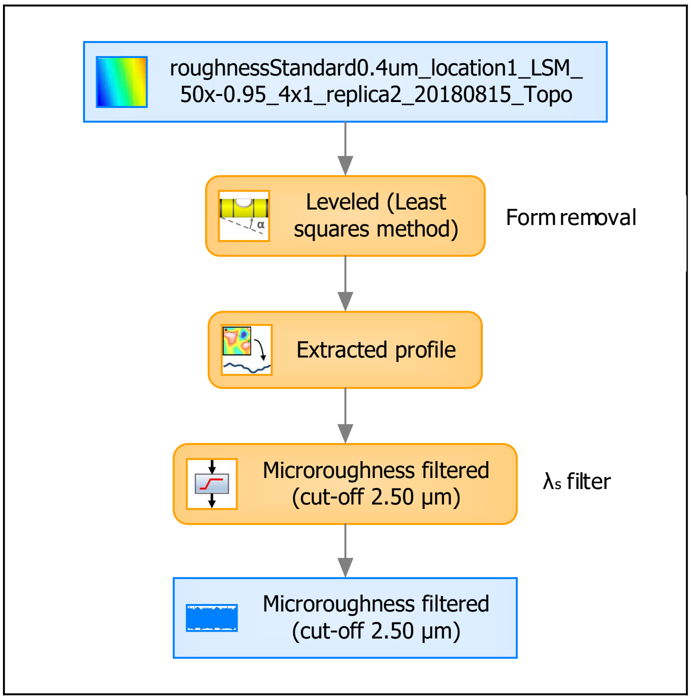

Roughness standard *Ra* = 0.4 um
50x objective, NA = 0.75 or 0.95, 4x1 tiles

**Identity card - roughnessStandard0.4um_locat...**		
Name:	roughnessStandard0.4um_location1_LSM_50x-0.95_...	
Created on:	8/15/2018 1:21:05 PM	
**Axis:**	**X**	
Length:	944	µm
Size:	7569	points
Spacing:	0.125	µm
**Axis:**	**Y**	
Length:	258	µm
Size:	2069	points
Spacing:	0.125	µm
**Axis:**	**Z**	
Length:	11.3	µm
Size:	65531	digits
Spacing:	0.173	nm
NMP ratio:	0.987 % (154588 Pts)	

Extracted

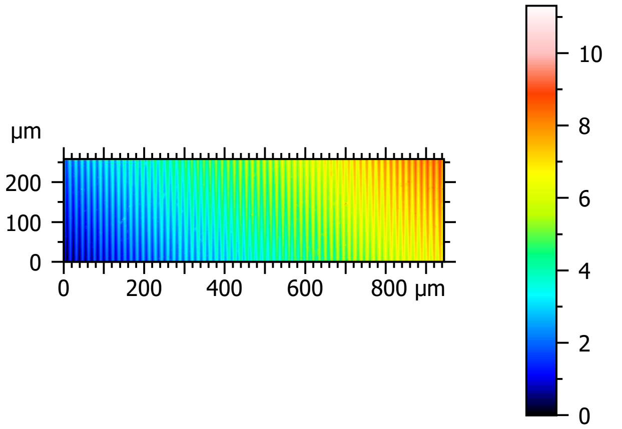

Levelled surface (least square method by subtraction)

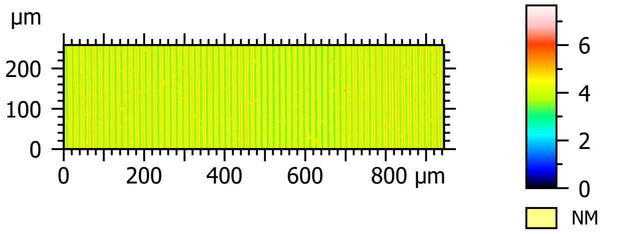

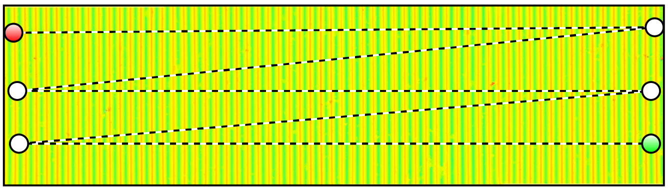

Extracted profile

Extracted profile

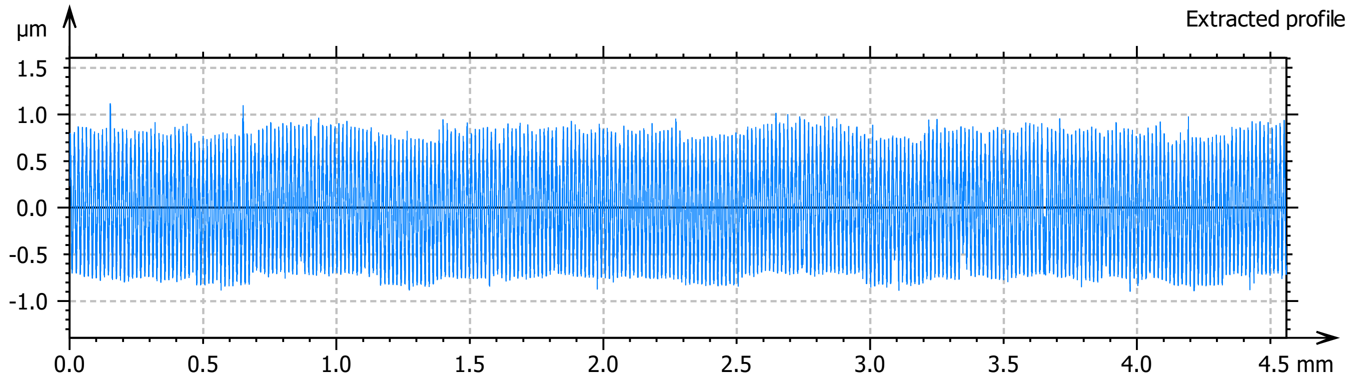

**Parameters**	Value	Unit
Length	4.56	mm

Primary profile (Gaussian filter, λ_s =2.5 µm)

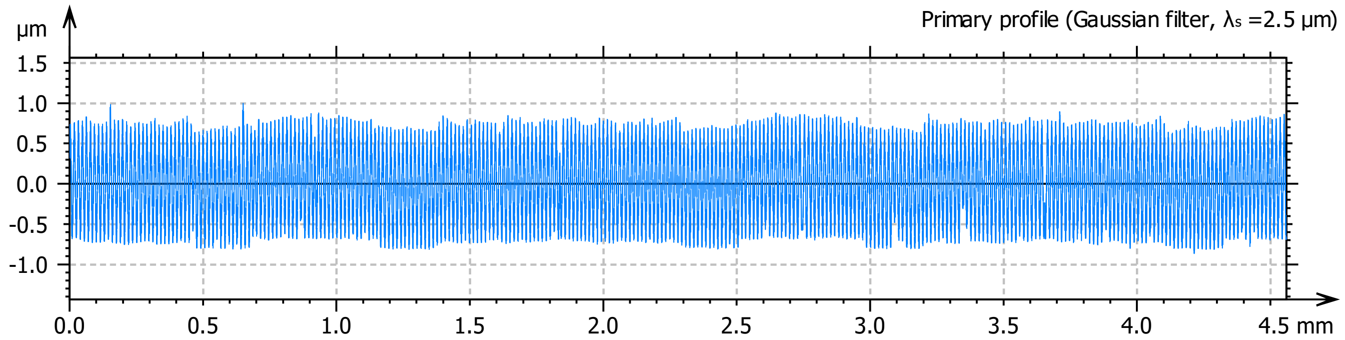

**Parameters**	Value	Unit
Length	4.56	mm

ISO 4287				
**Amplitude parameters - Roughness profile**				
Ra	0.417	µm	*Gaussian filter, 0.8 mm*	*Arithmetic mean deviation of the roughness profile.*

**Parameters**	Value	Unit	Tolerance	
Ra - Amplitude parameters - Roughness profile - ISO 4287	0.417	µm	0.400 µm ± 0.050 µm	✓

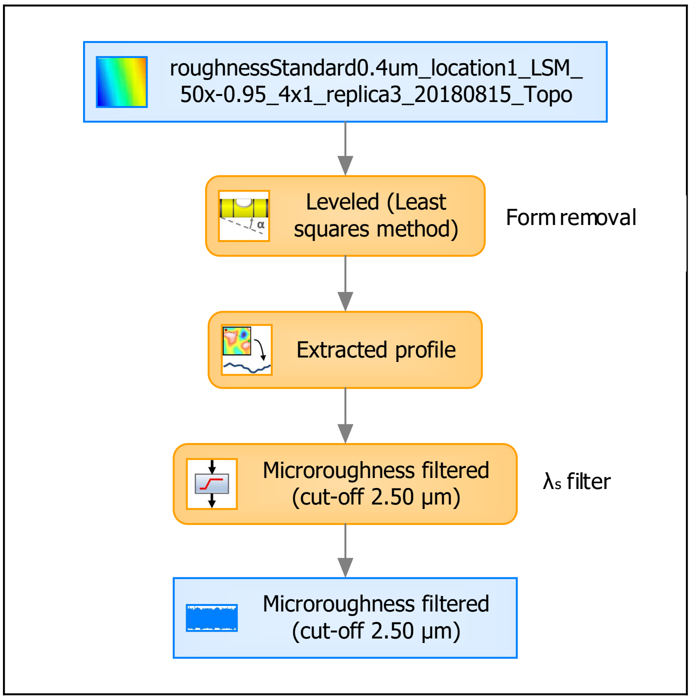

Roughness standard *Ra* = 0.4 um
50x objective, NA = 0.75 or 0.95, 4x1 tiles

Extracted

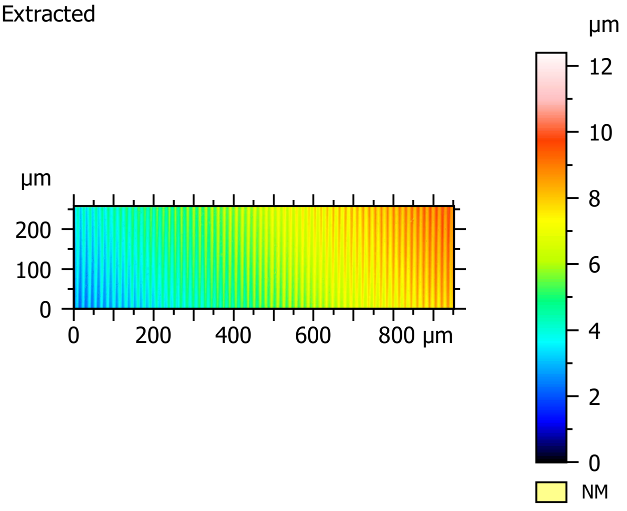

Levelled surface (least square method by subtraction)

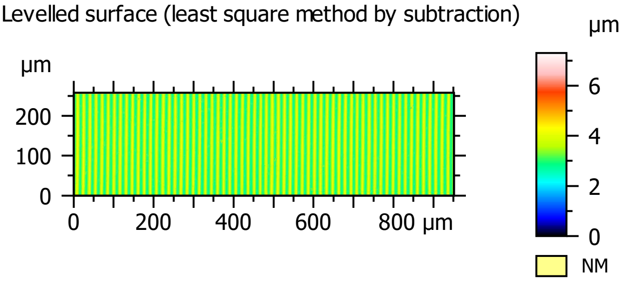

Identity card - roughnessStandard0.4um_locat...		
Name:	roughnessStandard0.4um_location2_LSM_50x-0.75_...	
Created on:	8/16/2018 1:50:21 PM	
**Axis:**	**X**	
Length:	951	µm
Size:	7625	points
Spacing:	0.125	µm
**Axis:**	**Y**	
Length:	258	µm
Size:	2069	points
Spacing:	0.125	µm
**Axis:**	**Z**	
Length:	12.4	µm
Size:	65532	digits
Spacing:	0.189	nm
NMP ratio:	0.990 % (156156 Pts)	

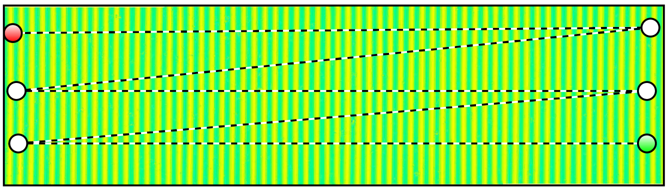

Extracted profile

Extracted profile

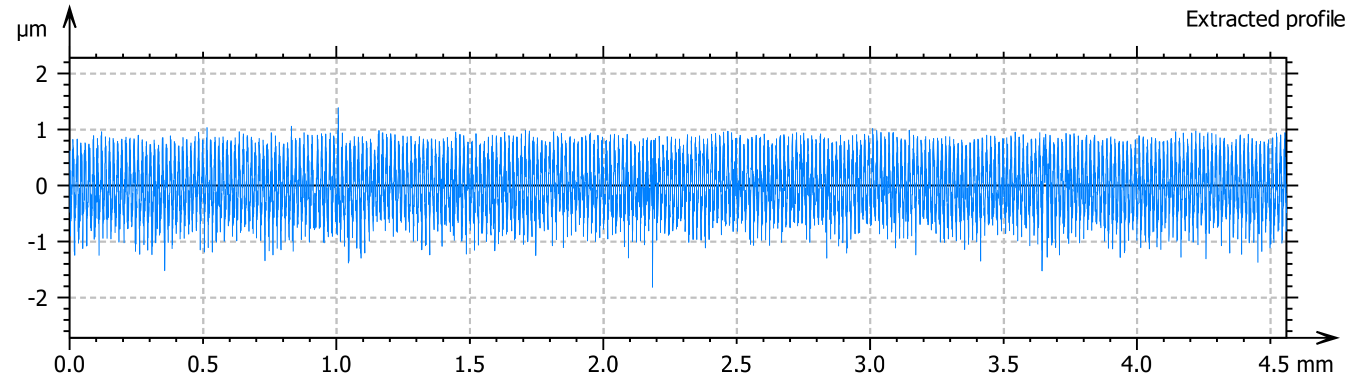

**Parameters**	Value	Unit
Length	4.56	mm

Primary profile (Gaussian filter, λ_s =2.5 µm)

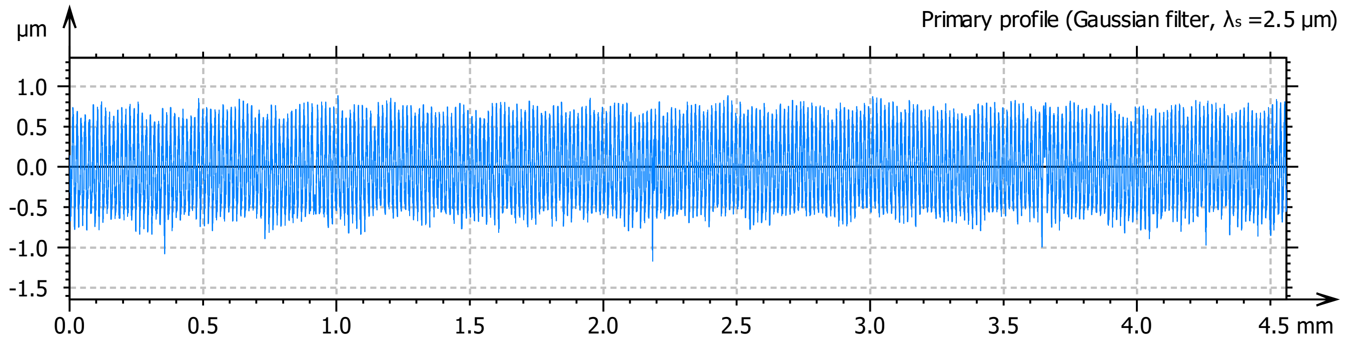

**Parameters**	Value	Unit
Length	4.56	mm

ISO 4287				
**Amplitude parameters - Roughness profile**				
Ra	0.396	µm	*Gaussian filter, 0.8 mm*	*Arithmetic mean deviation of the roughness profile.*

**Parameters**	Value	Unit	Tolerance	
Ra - Amplitude parameters - Roughness profile - ISO 4287	0.396	µm	0.400 µm ± 0.050 µm	✓

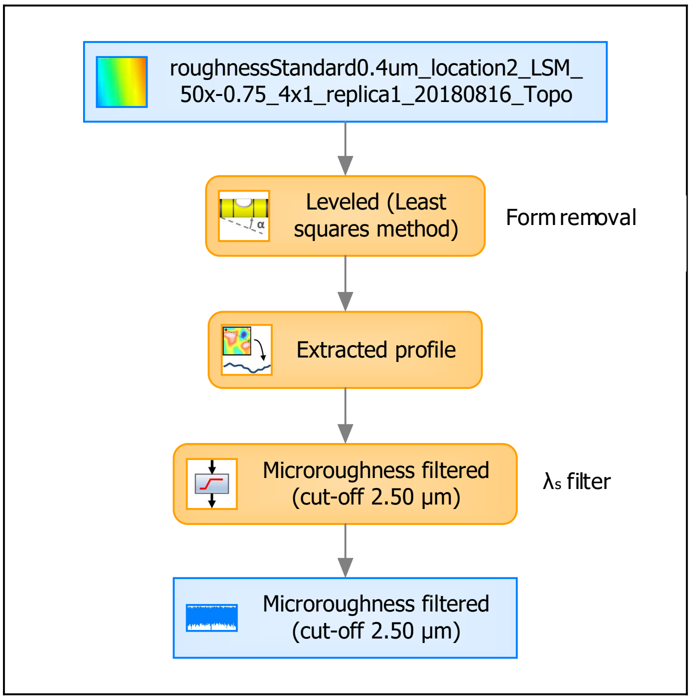

Roughness standard *Ra* = 0.4 um
50x objective, NA = 0.75 or 0.95, 4x1 tiles

Extracted

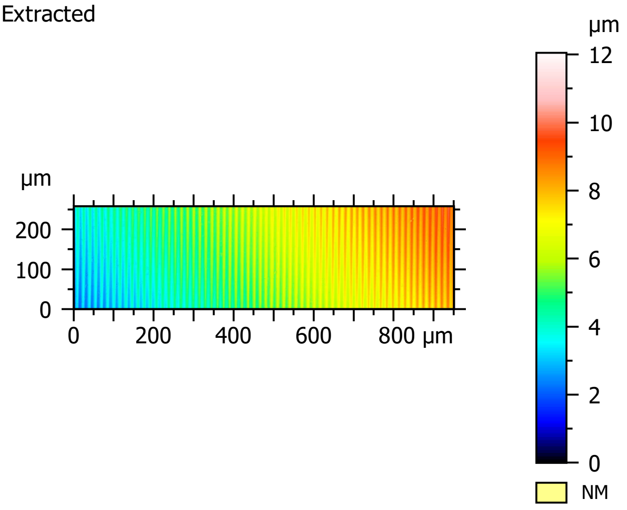

Identity card - roughnessStandard0.4um_locat...		
Name:	roughnessStandard0.4um_location2_LSM_50x-0.75_...	
Created on:	8/16/2018 2:05:37 PM	
**Axis:**	**X**	
Length:	951	µm
Size:	7622	points
Spacing:	0.125	µm
**Axis:**	**Y**	
Length:	258	µm
Size:	2069	points
Spacing:	0.125	µm
**Axis:**	**Z**	
Length:	12.0	µm
Size:	65532	digits
Spacing:	0.184	nm
NMP ratio:	0.990 % (156072 Pts)	

Levelled surface (least square method by subtraction)

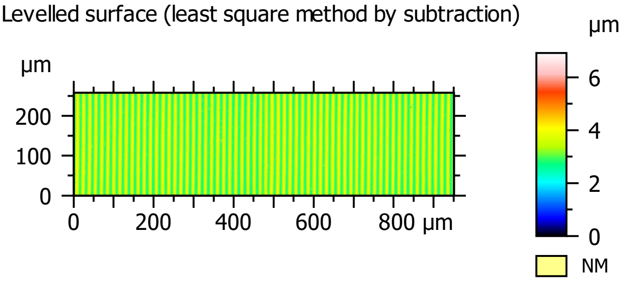

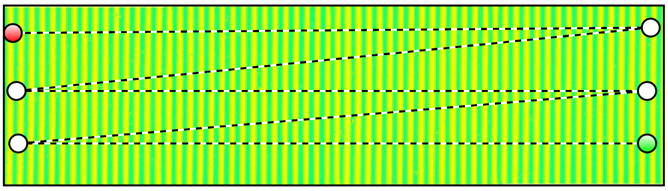

Extracted profile

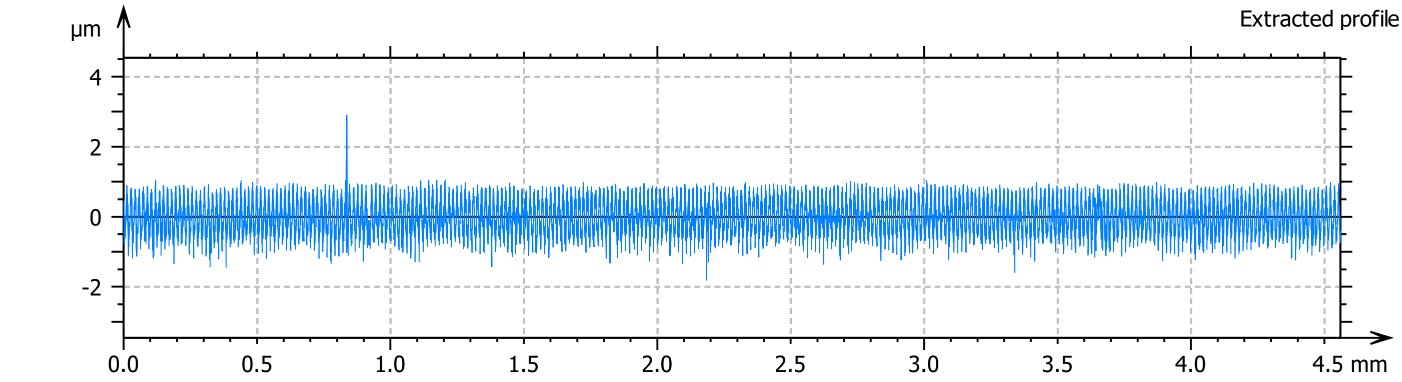

**Parameters**	Value	Unit
Length	4.56	mm

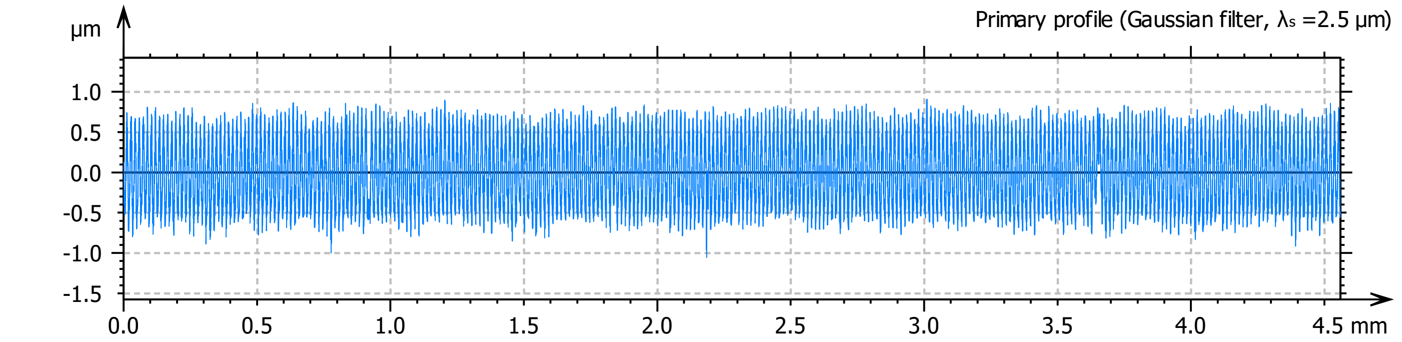

**Parameters**	Value	Unit
Length	4.56	mm

ISO 4287				
**Amplitude parameters - Roughness profile**				
Ra	0.398	µm	*Gaussian filter, 0.8 mm*	*Arithmetic mean deviation of the roughness profile.*

**Parameters**	Value	Unit	Tolerance	
Ra - Amplitude parameters - Roughness profile - ISO 4287	0.398	µm	0.400 µm ± 0.050 µm	✓

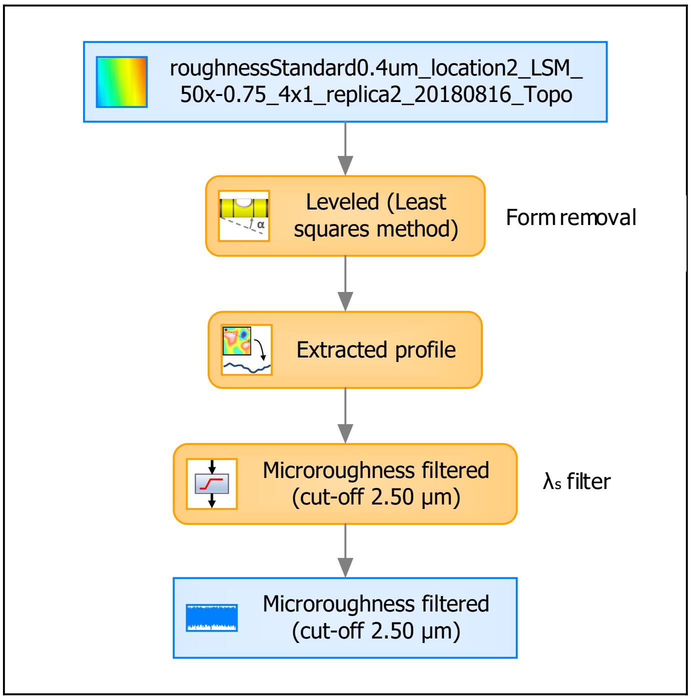

Roughness standard *Ra* = 0.4 um
50x objective, NA = 0.75 or 0.95, 4x1 tiles

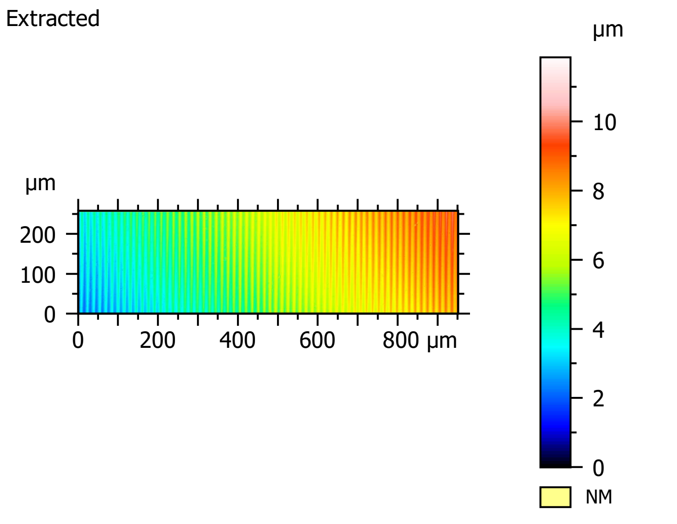

Identity card - roughnessStandard0.4um_locat...		
Name:	roughnessStandard0.4um_location2_LSM_50x-0.75_...	
Created on:	8/16/2018 2:21:48 PM	
**Axis:**	**X**	
Length:	951	µm
Size:	7625	points
Spacing:	0.125	µm
**Axis:**	**Y**	
Length:	258	µm
Size:	2069	points
Spacing:	0.125	µm
**Axis:**	**Z**	
Length:	11.9	µm
Size:	65531	digits
Spacing:	0.181	nm
NMP ratio:	0.990 % (156156 Pts)	

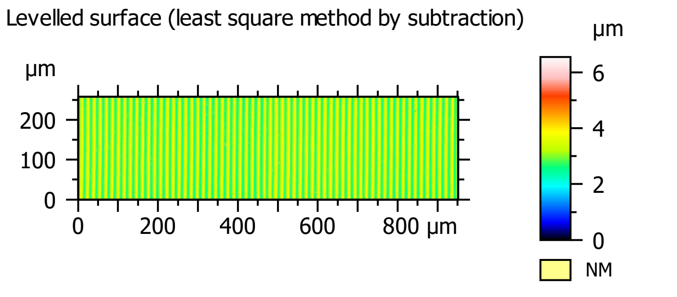

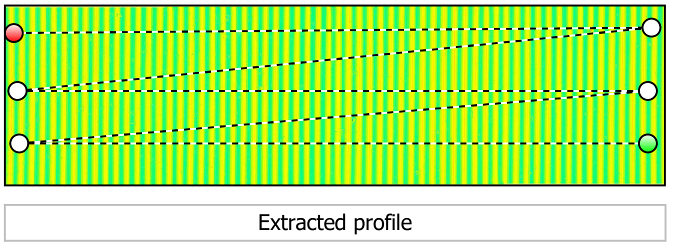

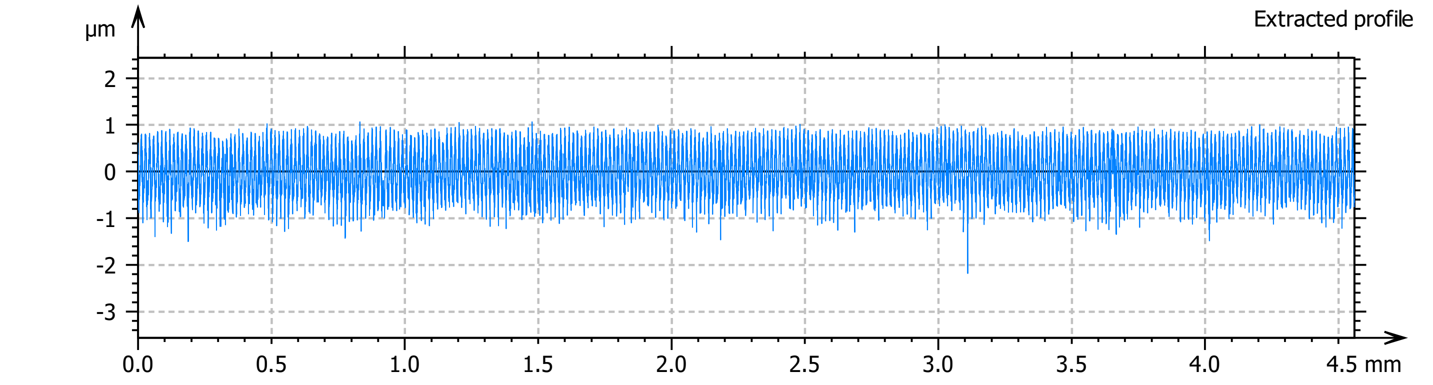

**Parameters**	Value	Unit
Length	4.56	mm

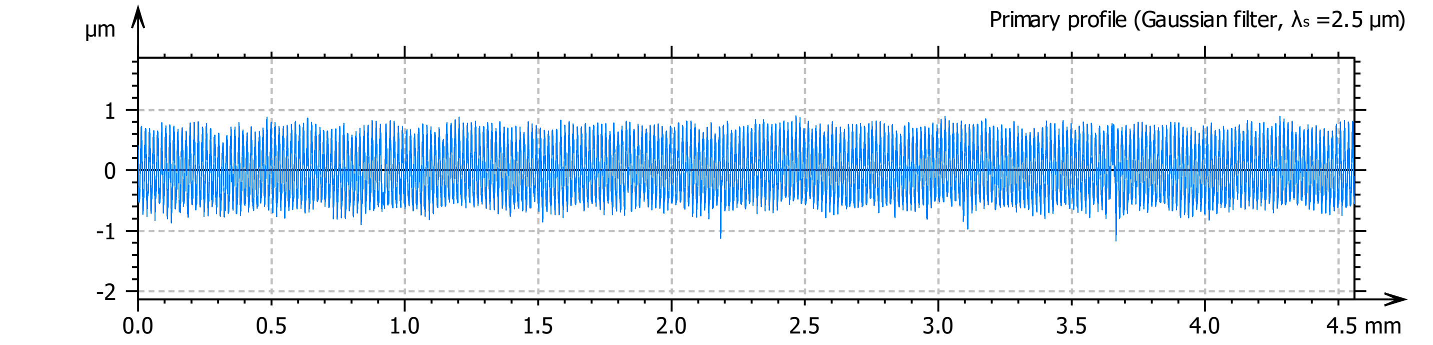

**Parameters**	Value	Unit
Length	4.56	mm

ISO 4287				
**Amplitude parameters - Roughness profile**				
Ra	0.398	µm	*Gaussian filter, 0.8 mm*	*Arithmetic mean deviation of the roughness profile.*

**Parameters**	Value	Unit	Tolerance	
Ra - Amplitude parameters - Roughness profile - ISO 4287	0.398	µm	0.400 µm ± 0.050 µm	✓

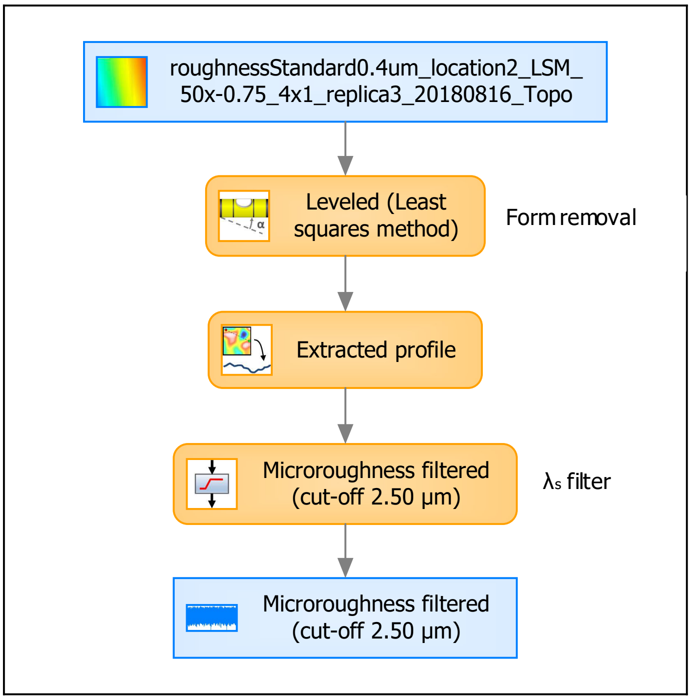

Roughness standard *Ra* = 0.4 um
50x objective, NA = 0.75 or 0.95, 4x1 tiles

Extracted

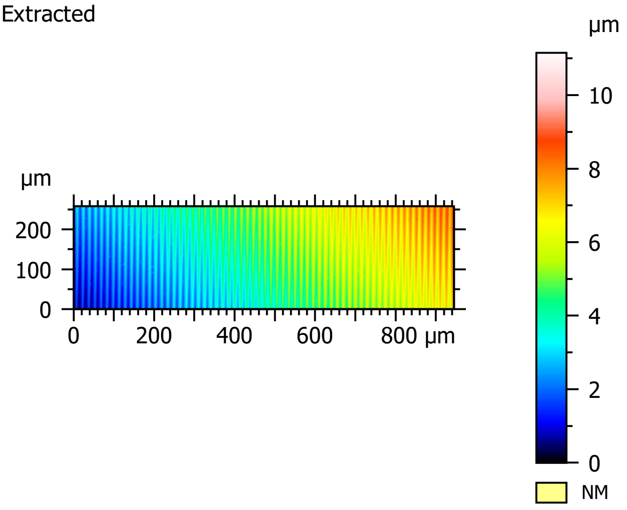

**Identity card - roughnessStandard0.4um_locat...**		
Name:	roughnessStandard0.4um_location2_LSM_50x-0.95_...	
Created on:	8/16/2018 1:40:35 PM	
**Axis:**	**X**	
Length:	945	µm
Size:	7574	points
Spacing:	0.125	µm
**Axis:**	**Y**	
Length:	258	µm
Size:	2069	points
Spacing:	0.125	µm
**Axis:**	**Z**	
Length:	11.2	µm
Size:	65531	digits
Spacing:	0.170	nm
NMP ratio:	0.987 % (154728 Pts)	

Levelled surface (least square method by subtraction)

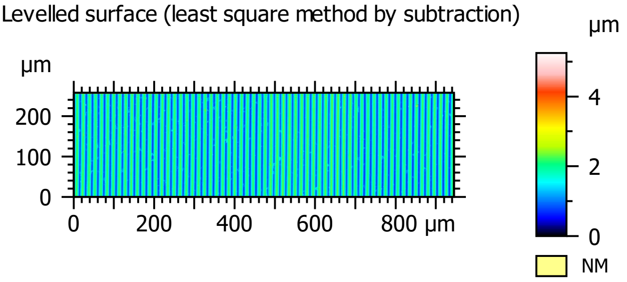

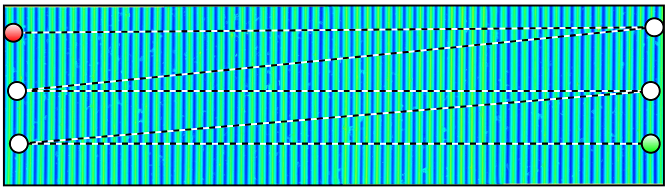

Extracted profile

Extracted profile

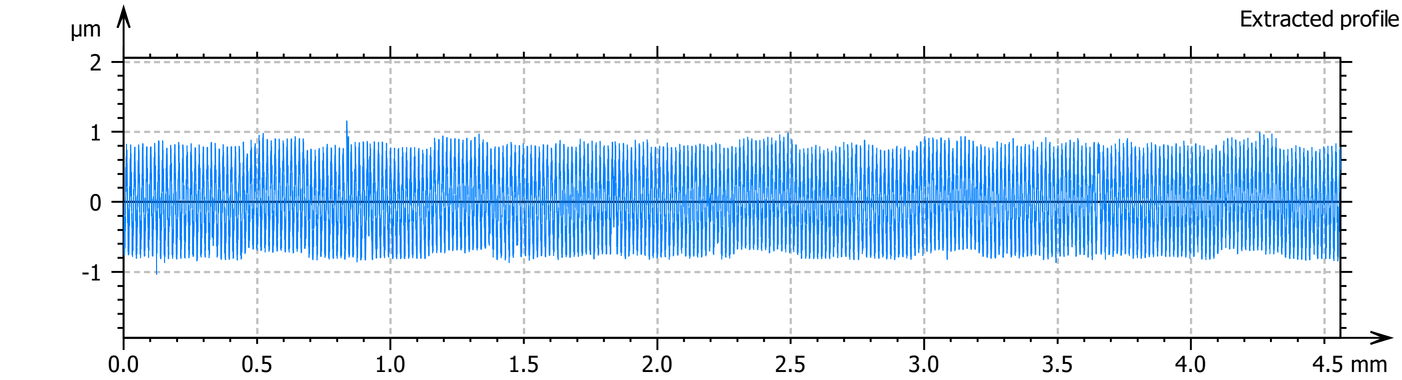

**Parameters**	Value	Unit
Length	4.56	mm

Primary profile (Gaussian filter, λ_s =2.5 µm)

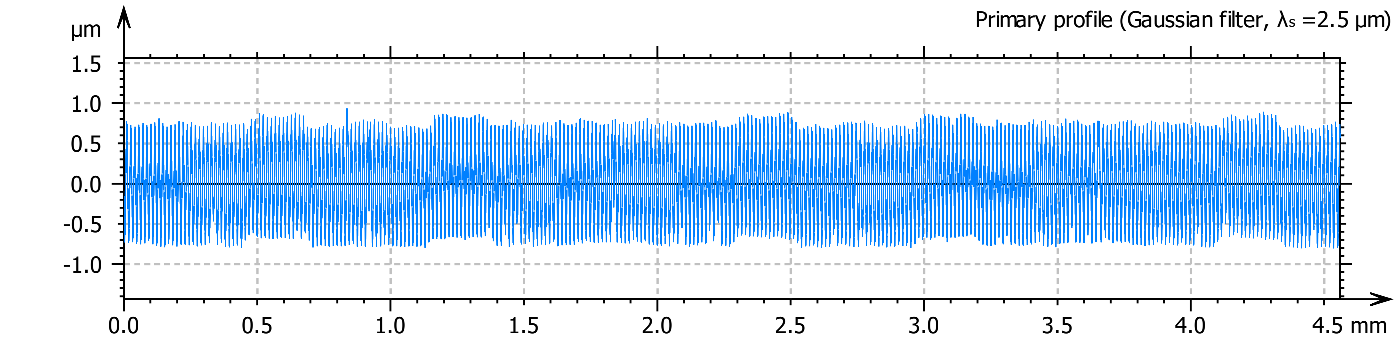

**Parameters**	Value	Unit
Length	4.56	mm

ConfoMap ST 7.4.8633

ISO 4287				
**Amplitude parameters - Roughness profile**				
Ra	0.419	µm	*Gaussian filter, 0.8 mm*	*Arithmetic mean deviation of the roughness profile.*

**Parameters**	Value	Unit	Tolerance	
Ra - Amplitude parameters - Roughness profile - ISO 4287	0.419	µm	0.400 µm ± 0.050 µm	✓

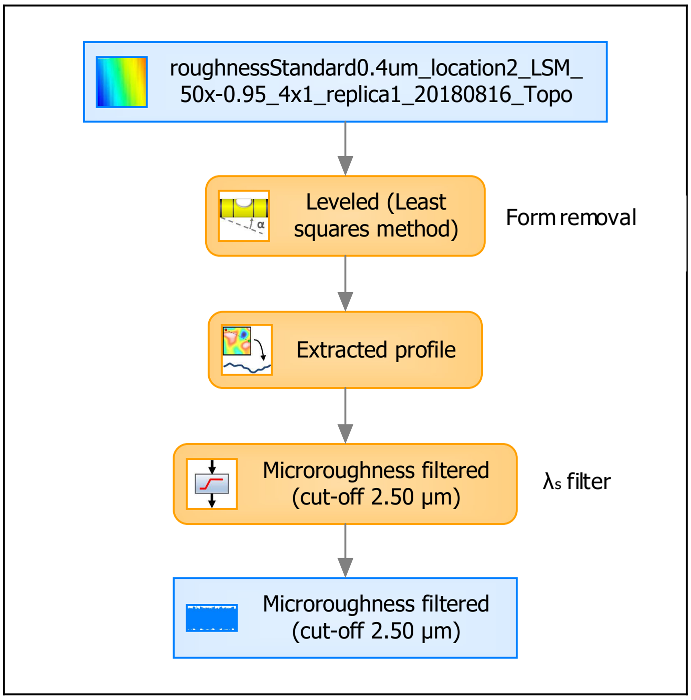

Roughness standard *Ra* = 0.4 um
50x objective, NA = 0.75 or 0.95, 4x1 tiles

**Identity card - roughnessStandard0.4um_locat...**		
Name:	roughnessStandard0.4um_location2_LSM_50x-0.95_...	
Created on:	8/16/2018 1:58:03 PM	
**Axis:**	**X**	
Length:	945	µm
Size:	7577	points
Spacing:	0.125	µm
**Axis:**	**Y**	
Length:	258	µm
Size:	2069	points
Spacing:	0.125	µm
**Axis:**	**Z**	
Length:	11.0	µm
Size:	65531	digits
Spacing:	0.168	nm
NMP ratio:	0.988 % (154812 Pts)	

Extracted

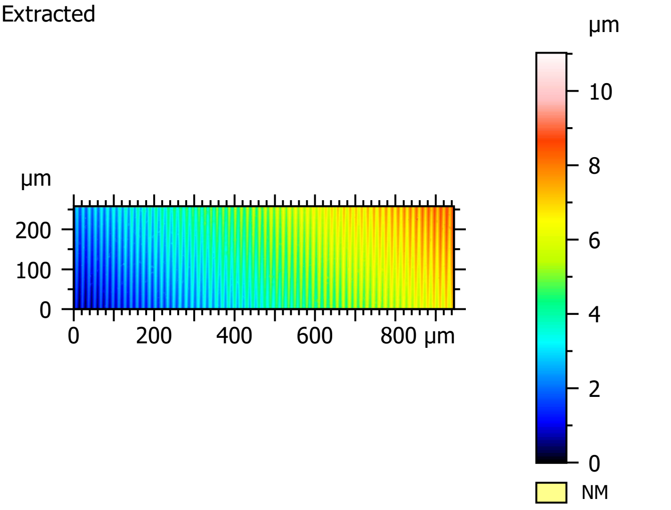

Levelled surface (least square method by subtraction)

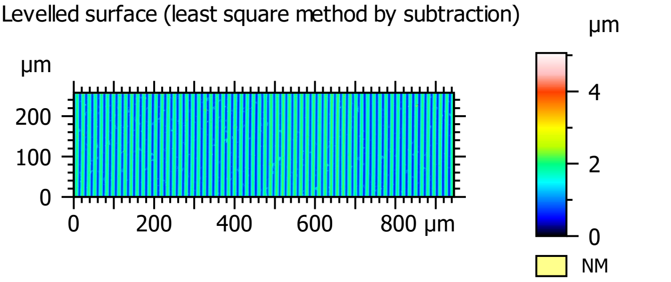

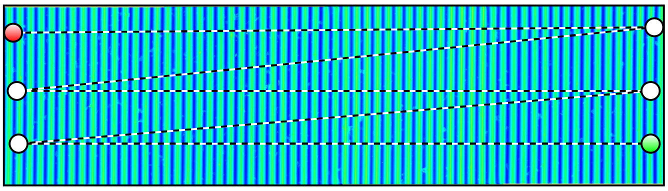

Extracted profile

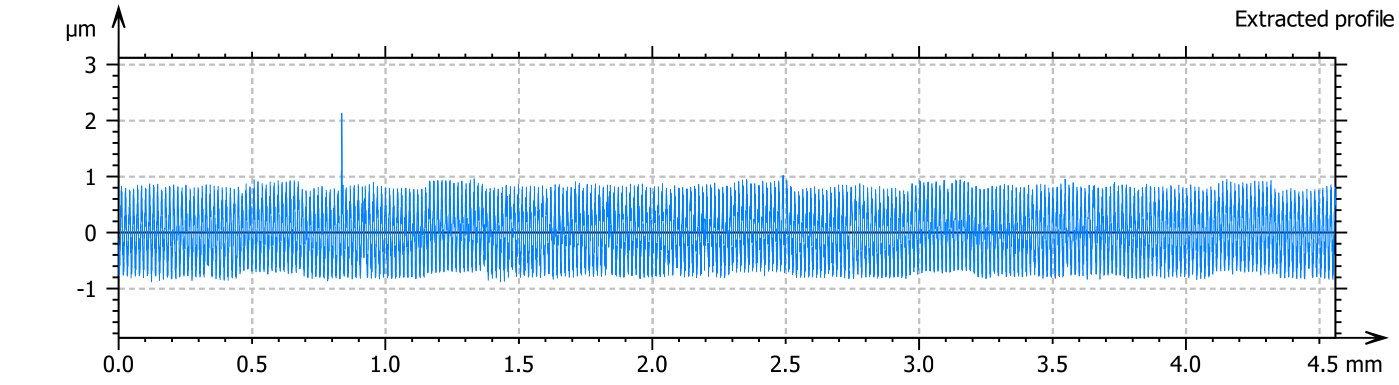

**Parameters**	Value	Unit
Length	4.56	mm

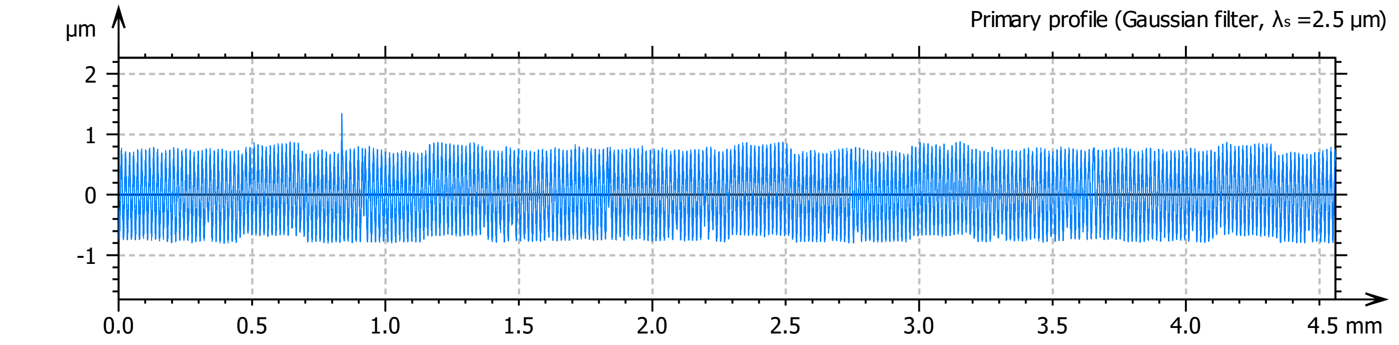

**Parameters**	Value	Unit
Length	4.56	mm

ISO 4287

Amplitude parameters - Roughness profile

Ra	0.420	µm	*Gaussian filter, 0.8 mm*	*Arithmetic mean deviation of the roughness profile.*

**Parameters**	Value	Unit	Tolerance	
Ra - Amplitude parameters - Roughness profile - ISO 4287	0.420	µm	0.400 µm ± 0.050 µm	✓

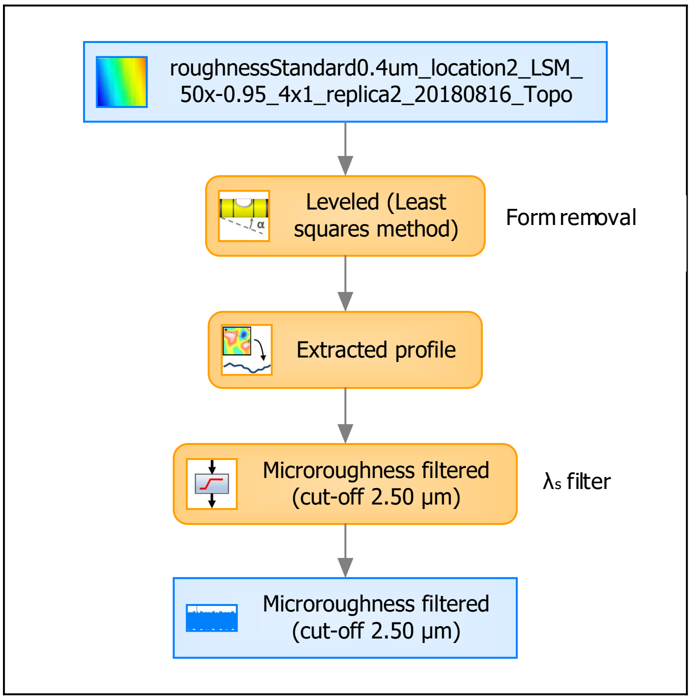

Roughness standard *Ra* = 0.4 um
50x objective, NA = 0.75 or 0.95, 4x1 tiles

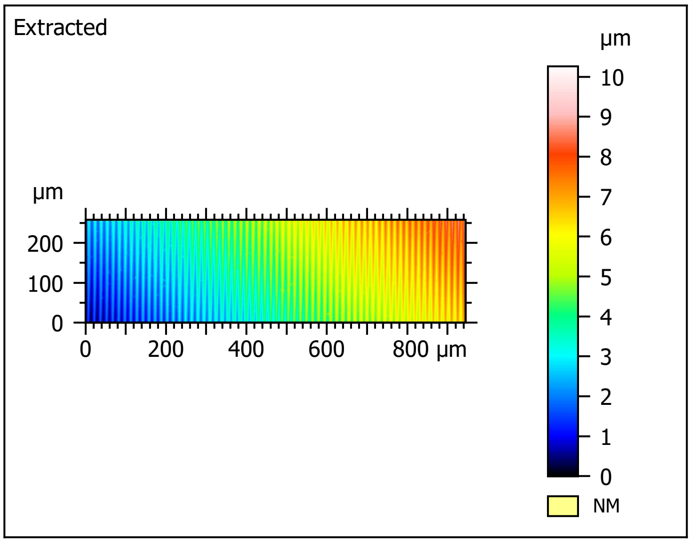

Identity card - roughnessStandard0.4um_locat...		
Name:	roughnessStandard0.4um_location2_LSM_50x-0.95_...	
Created on:	8/16/2018 2:13:32 PM	
**Axis:**	**X**	
Length:	945	µm
Size:	7574	points
Spacing:	0.125	µm
**Axis:**	**Y**	
Length:	258	µm
Size:	2069	points
Spacing:	0.125	µm
**Axis:**	**Z**	
Length:	10.3	µm
Size:	65532	digits
Spacing:	0.157	nm
NMP ratio:	0.987 % (154728 Pts)	

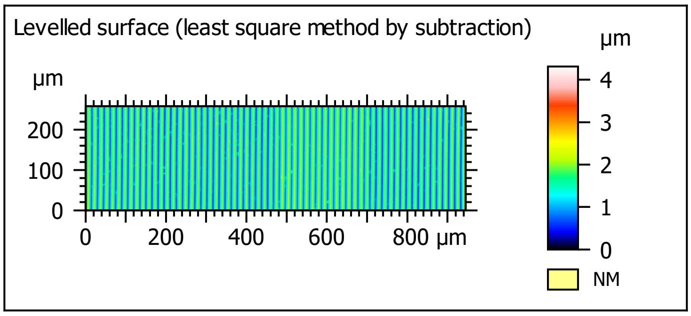

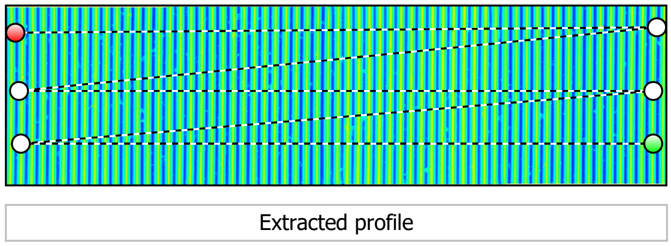

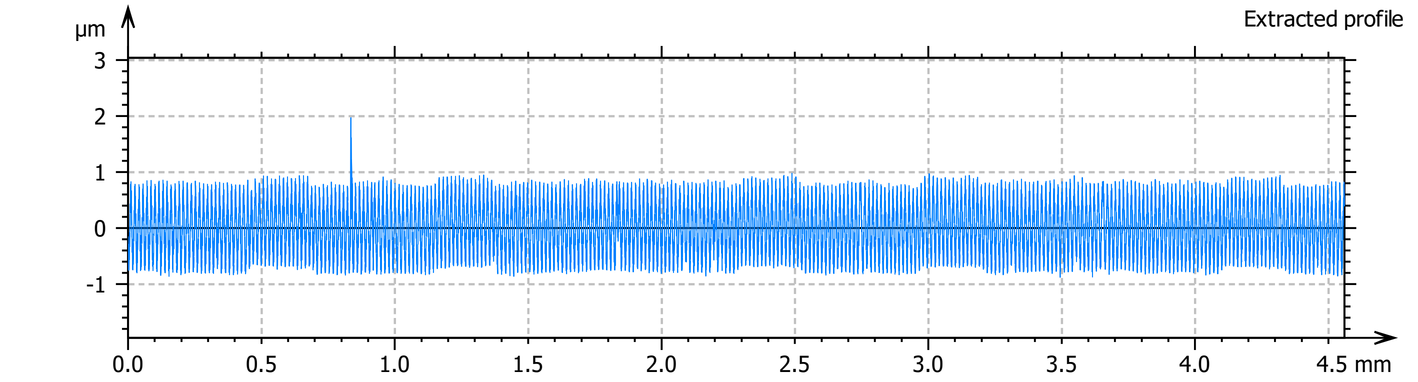

**Parameters**	Value	Unit
Length	4.56	mm

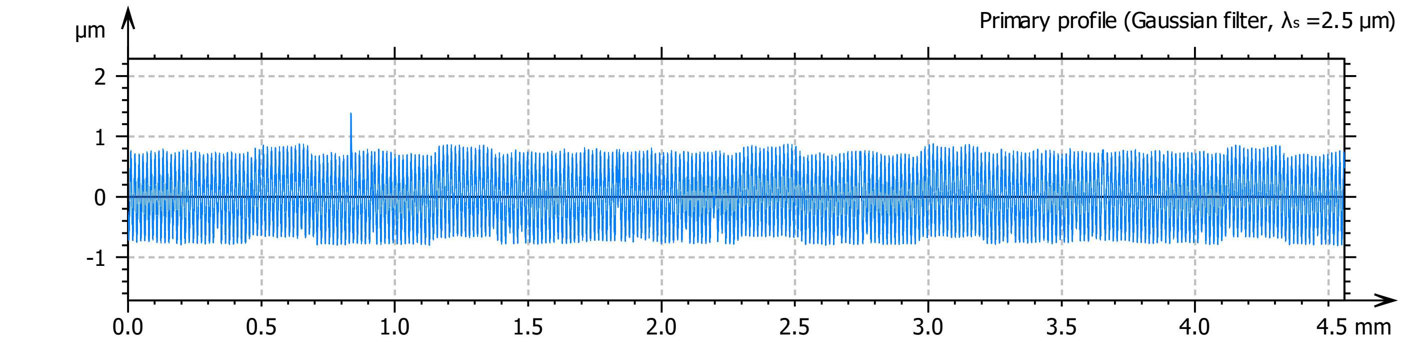

**Parameters**	Value	Unit
Length	4.56	mm

ISO 4287				
**Amplitude parameters - Roughness profile**				
Ra	0.418	µm	*Gaussian filter, 0.8 mm*	*Arithmetic mean deviation of the roughness profile.*

**Parameters**	Value	Unit	Tolerance	
Ra - Amplitude parameters - Roughness profile - ISO 4287	0.418	µm	0.400 µm ± 0.050 µm	✓

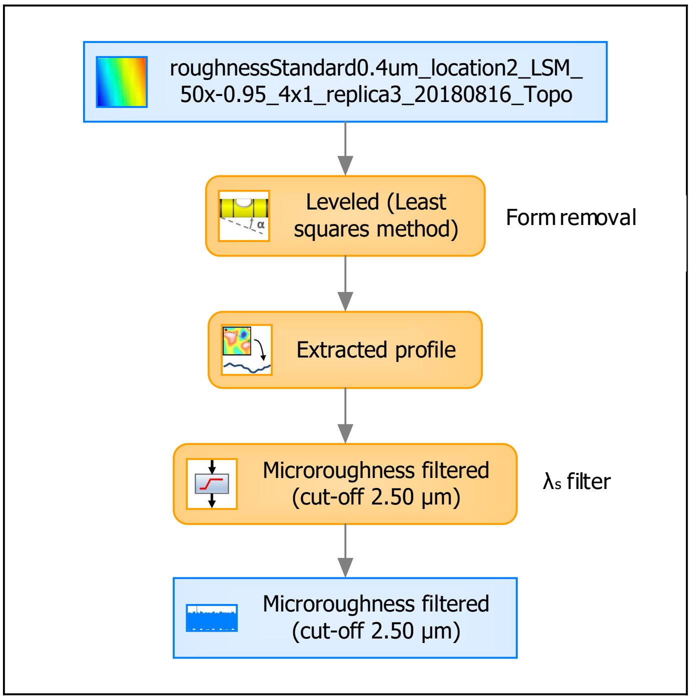

Roughness standard *Ra* = 0.4 um
50x objective, NA = 0.75 or 0.95, 4x1 tiles

Extracted

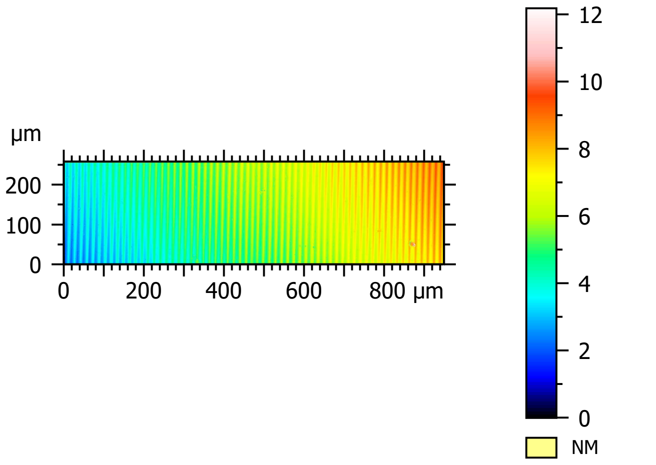

Identity card - roughnessStandard0.4um_locat...		
Name:	roughnessStandard0.4um_location3_LSM_50x-0.75_...	
Created on:	8/17/2018 2:05:20 PM	
**Axis:**	**X**	
Length:	950	µm
Size:	7611	points
Spacing:	0.125	µm
**Axis:**	**Y**	
Length:	258	µm
Size:	2069	points
Spacing:	0.125	µm
**Axis:**	**Z**	
Length:	12.2	µm
Size:	65531	digits
Spacing:	0.186	nm
NMP ratio:	0.989 % (155764 Pts)	

Levelled surface (least square method by subtraction)

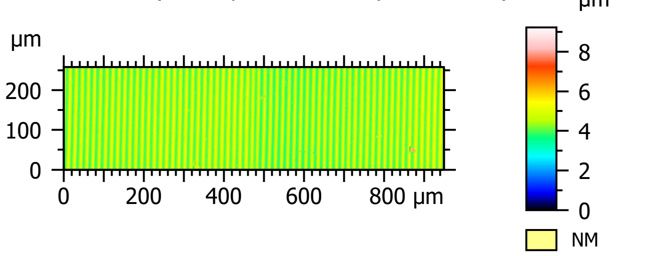

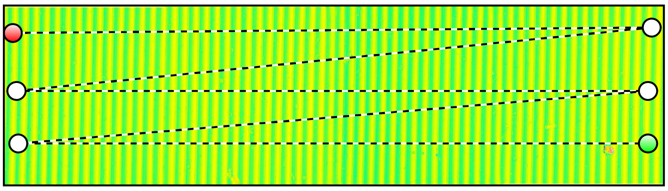

Extracted profile

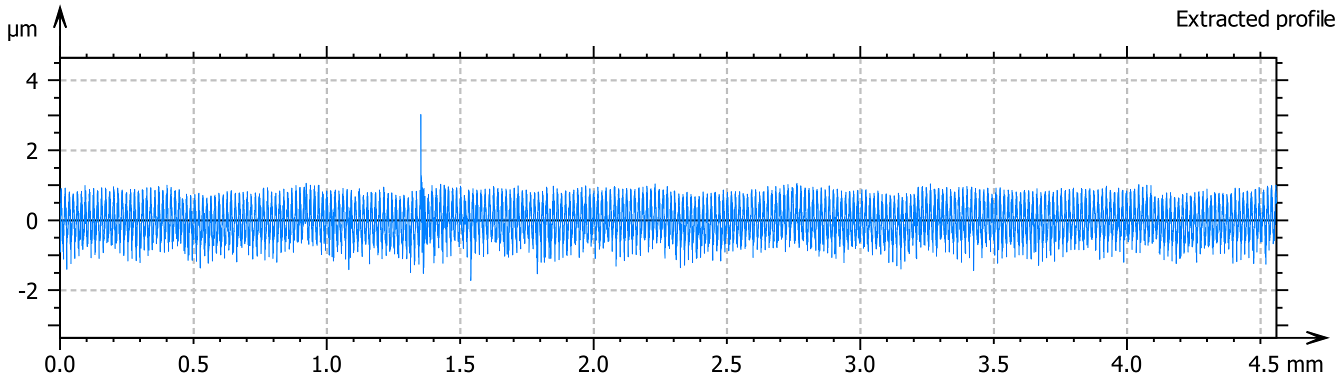

**Parameters**	Value	Unit
Length	4.56	mm

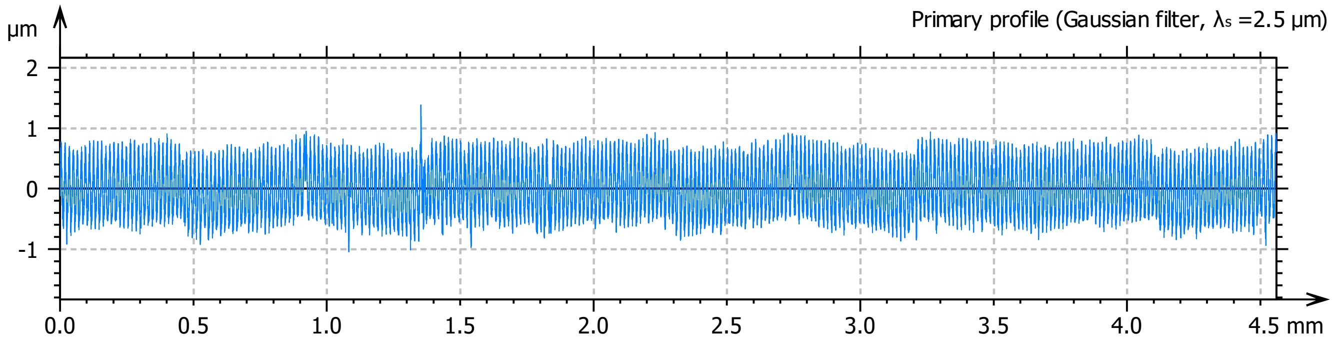

**Parameters**	Value	Unit
Length	4.56	mm

ISO 4287				
**Amplitude parameters - Roughness profile**				
Ra	0.397	µm	*Gaussian filter, 0.8 mm*	*Arithmetic mean deviation of the roughness profile.*

**Parameters**	Value	Unit	Tolerance	
Ra - Amplitude parameters - Roughness profile - ISO 4287	0.397	µm	0.400 µm ± 0.050 µm	✓

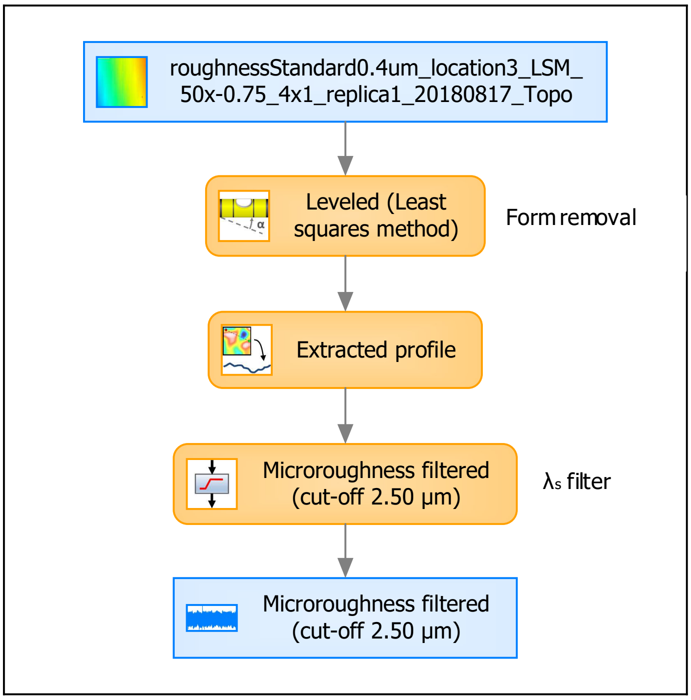

Roughness standard *Ra* = 0.4 um
50x objective, NA = 0.75 or 0.95, 4x1 tiles

Extracted

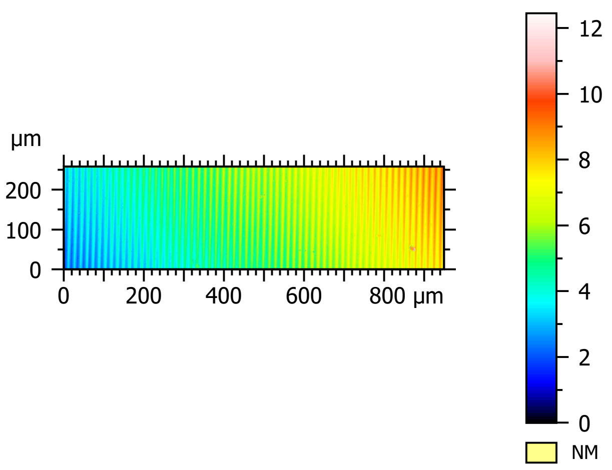

**Identity card - roughnessStandard0.4um_locat...**		
Name:	roughnessStandard0.4um_location3_LSM_50x-0.75_...	
Created on:	8/17/2018 2:24:37 PM	
**Axis:**	**X**	
Length:	950	µm
Size:	7611	points
Spacing:	0.125	µm
**Axis:**	**Y**	
Length:	258	µm
Size:	2069	points
Spacing:	0.125	µm
**Axis:**	**Z**	
Length:	12.4	µm
Size:	65532	digits
Spacing:	0.190	nm
NMP ratio:	0.989 % (155764 Pts)	

Levelled surface (least square method by subtraction)

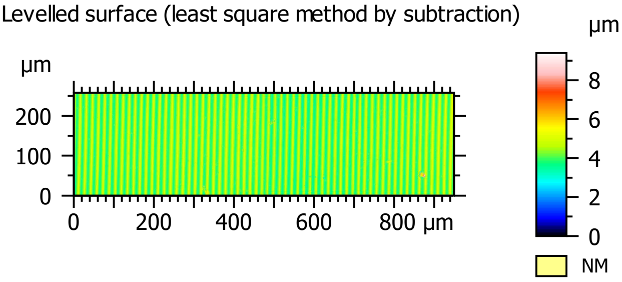

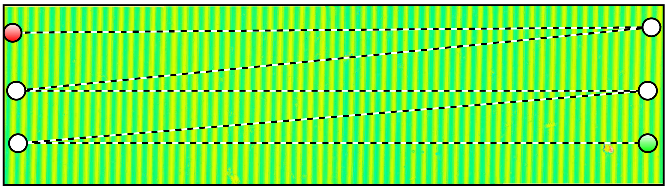

Extracted profile

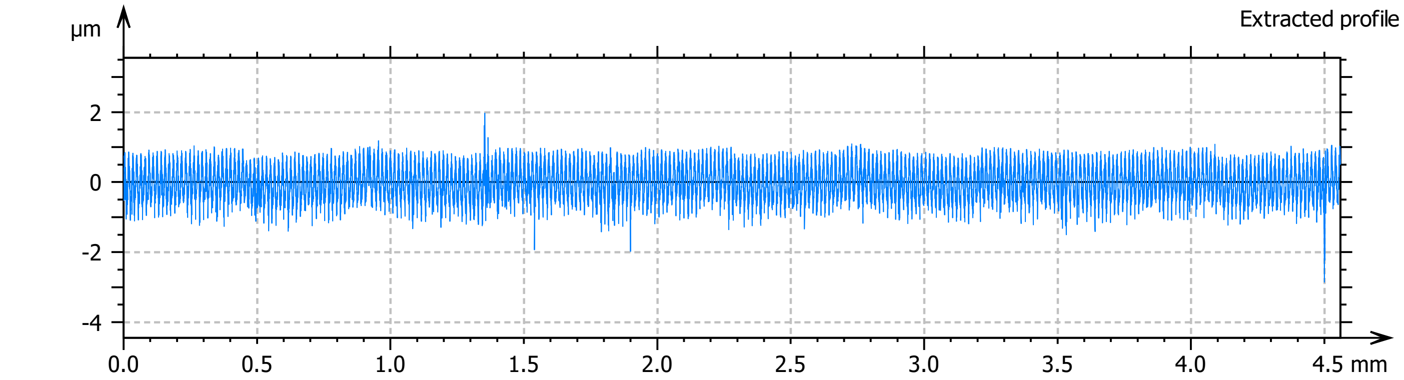

Extracted profile

**Parameters**	Value	Unit
Length	4.56	mm

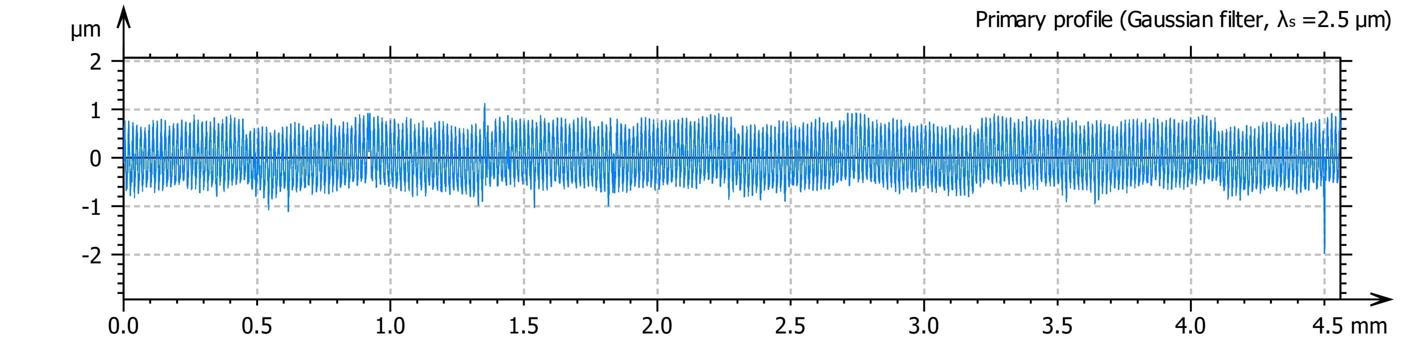

Primary profile (Gaussian filter, λ_s =2.5 µm)

**Parameters**	Value	Unit
Length	4.56	mm

ISO 4287				
**Amplitude parameters - Roughness profile**				
Ra	0.398	µm	*Gaussian filter, 0.8 mm*	*Arithmetic mean deviation of the roughness profile.*

**Parameters**	Value	Unit	Tolerance	
Ra - Amplitude parameters - Roughness profile - ISO 4287	0.398	µm	0.400 µm ± 0.050 µm	✓

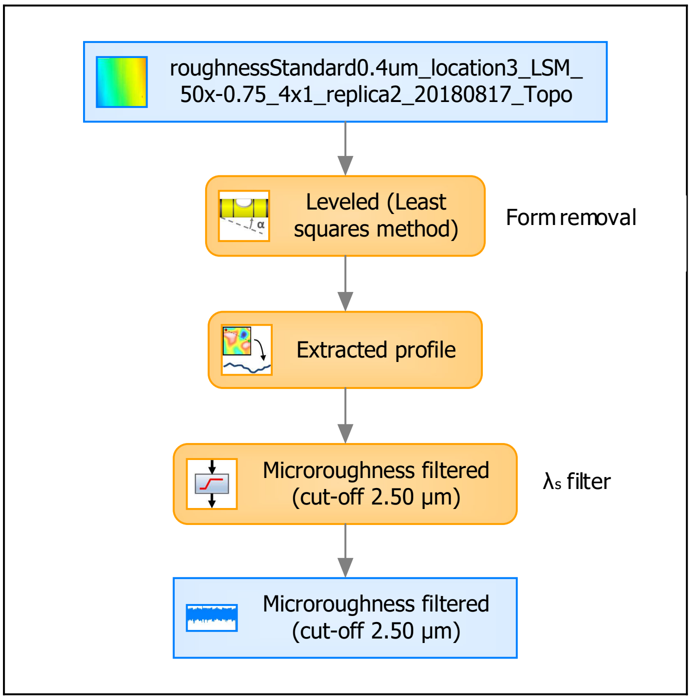

Roughness standard *Ra* = 0.4 um
50x objective, NA = 0.75 or 0.95, 4x1 tiles

Extracted

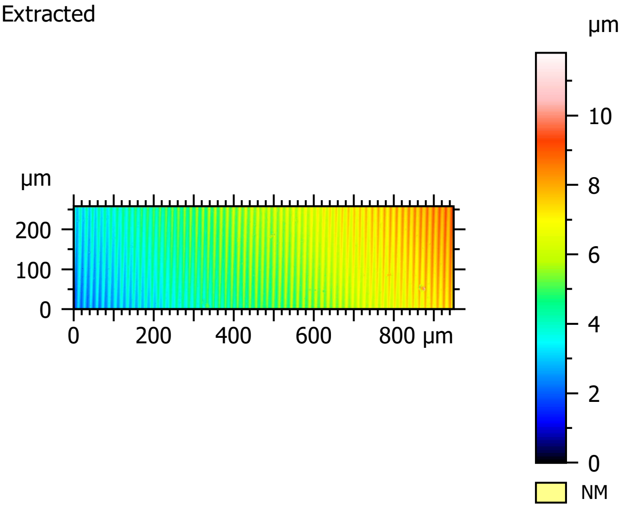

Levelled surface (least square method by subtraction)

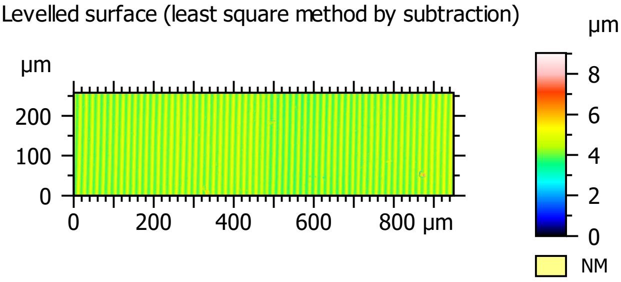

Identity card - roughnessStandard0.4um_locat...		
Name:	roughnessStandard0.4um_location3_LSM_50x-0.75_...	
Created on:	8/17/2018 2:41:54 PM	
**Axis:**	**X**	
Length:	950	µm
Size:	7611	points
Spacing:	0.125	µm
**Axis:**	**Y**	
Length:	258	µm
Size:	2069	points
Spacing:	0.125	µm
**Axis:**	**Z**	
Length:	11.8	µm
Size:	65532	digits
Spacing:	0.180	nm
NMP ratio:	0.989 % (155764 Pts)	

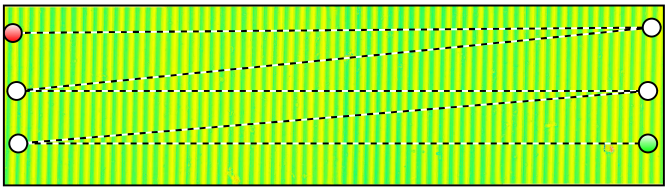

Extracted profile

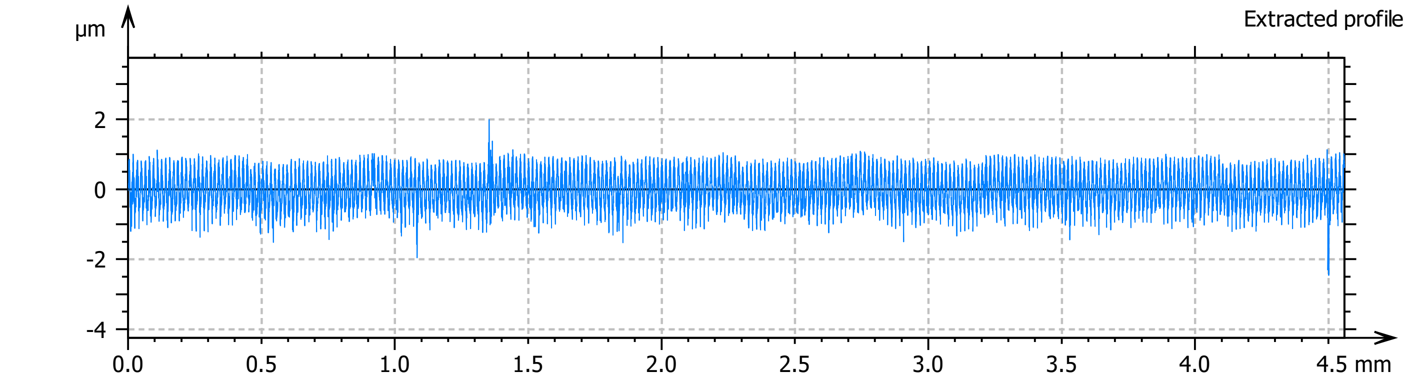

**Parameters**	Value	Unit
Length	4.56	mm

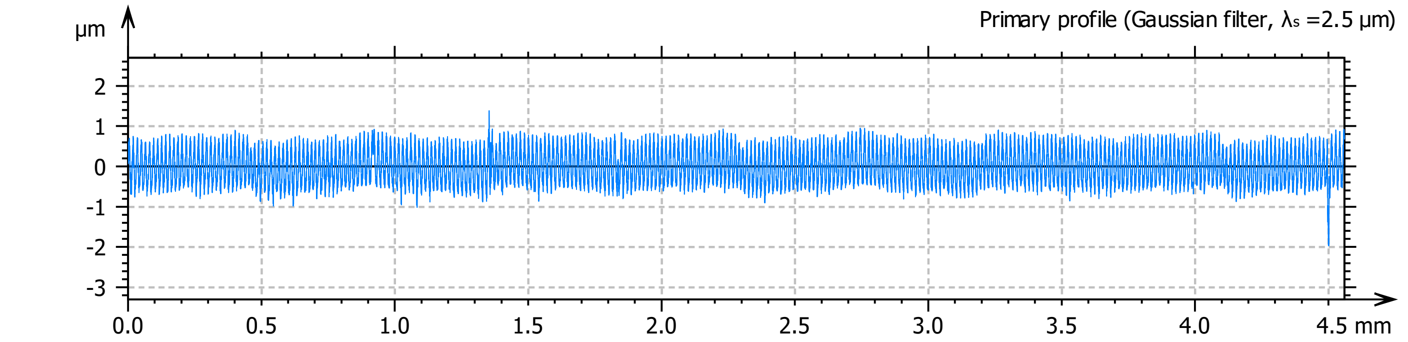

**Parameters**	Value	Unit
Length	4.56	mm

ISO 4287				
**Amplitude parameters - Roughness profile**				
Ra	0.398	µm	*Gaussian filter, 0.8 mm*	*Arithmetic mean deviation of the roughness profile.*

**Parameters**	Value	Unit	Tolerance	
Ra - Amplitude parameters - Roughness profile - ISO 4287	0.398	µm	0.400 µm ± 0.050 µm	✓

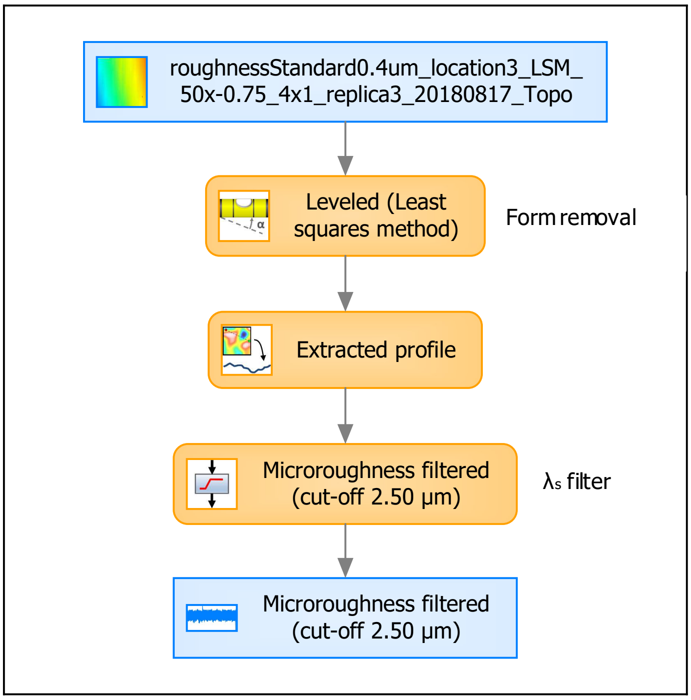

Roughness standard *Ra* = 0.4 um
50x objective, NA = 0.75 or 0.95, 4x1 tiles

Identity card - roughnessStandard0.4um_locat...		
Name:	roughnessStandard0.4um_location3_LSM_50x-0.95_...	
Created on:	8/17/2018 1:55:40 PM	
**Axis:**	**X**	
Length:	944	µm
Size:	7563	points
Spacing:	0.125	µm
**Axis:**	**Y**	
Length:	258	µm
Size:	2069	points
Spacing:	0.125	µm
**Axis:**	**Z**	
Length:	10.4	µm
Size:	65531	digits
Spacing:	0.158	nm
NMP ratio:	0.987 % (154420 Pts)	

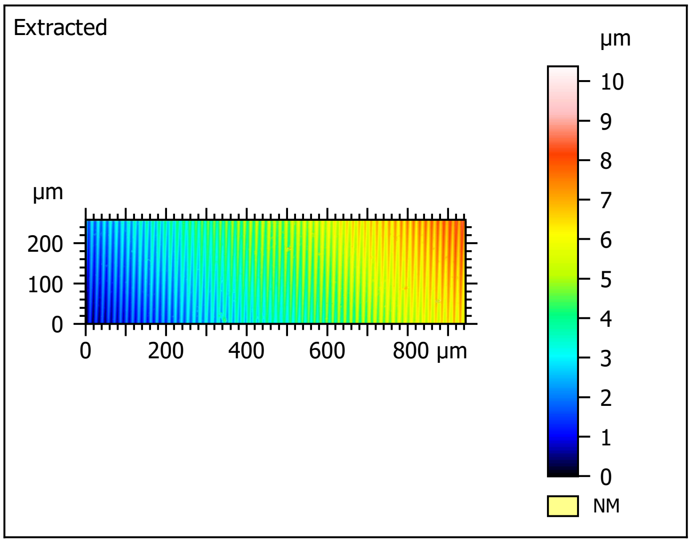

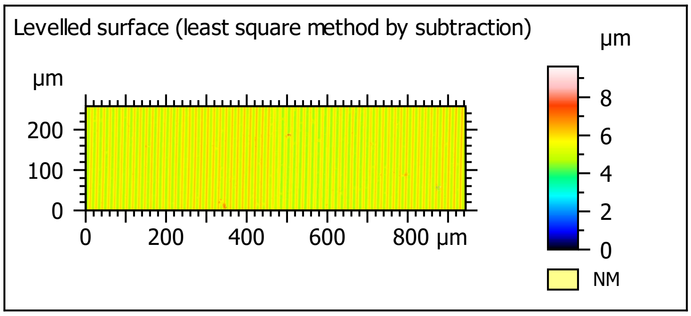

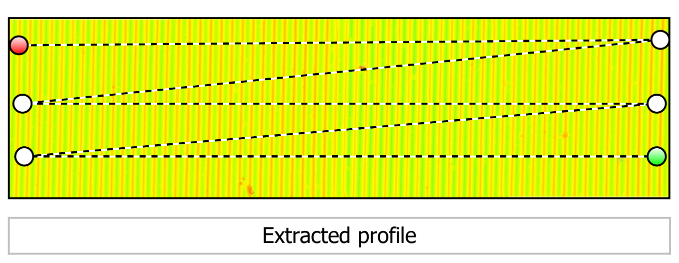

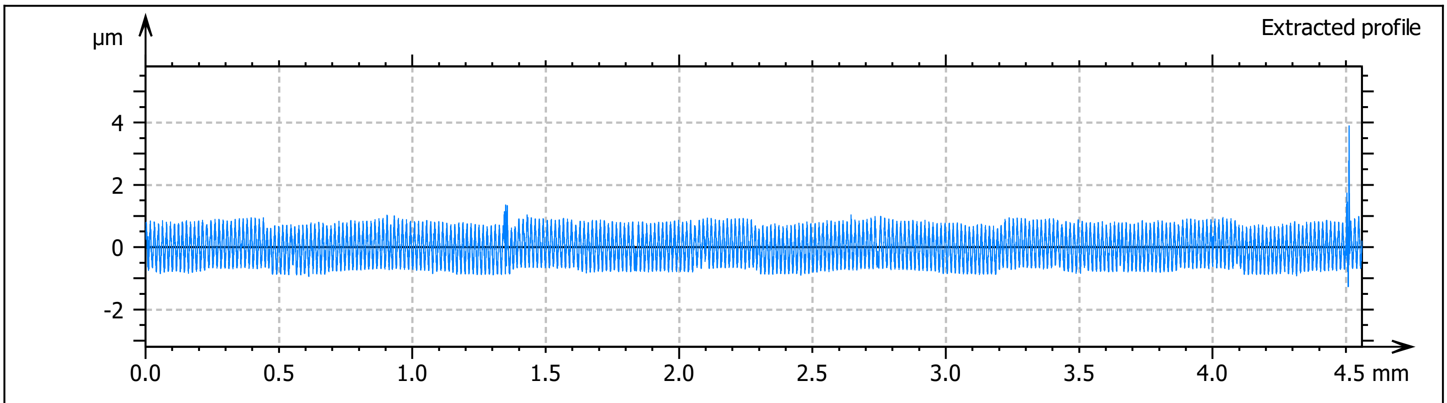

**Parameters**	Value	Unit
Length	4.56	mm

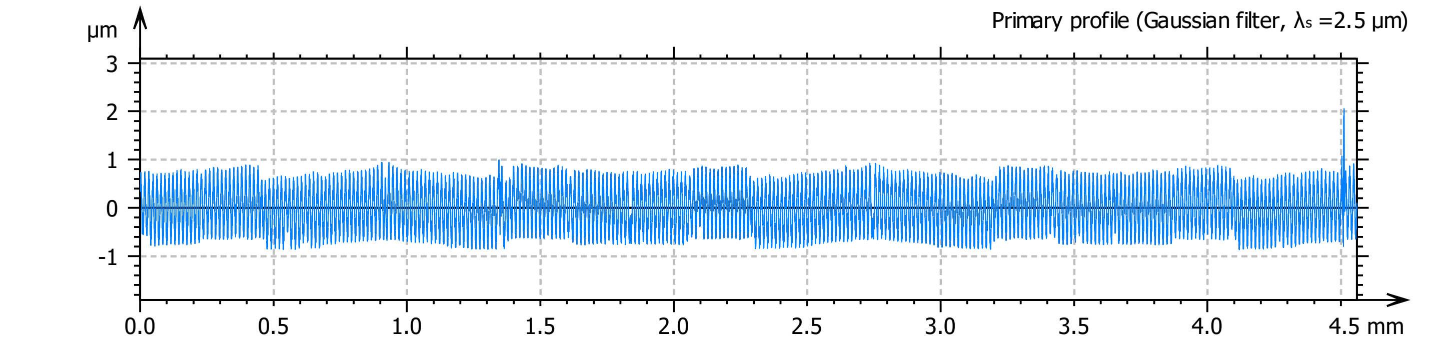

**Parameters**	Value	Unit
Length	4.56	mm

ISO 4287				
**Amplitude parameters - Roughness profile**				
Ra	0.420	µm	*Gaussian filter, 0.8 mm*	*Arithmetic mean deviation of the roughness profile.*

**Parameters**	Value	Unit	Tolerance	
Ra - Amplitude parameters - Roughness profile - ISO 4287	0.420	µm	0.400 µm ± 0.050 µm	✓

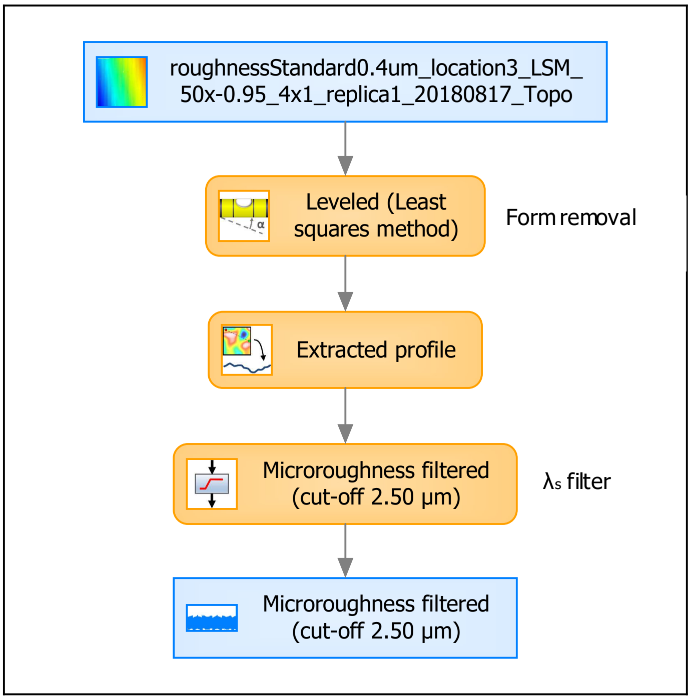

Roughness standard *Ra* = 0.4 um
50x objective, NA = 0.75 or 0.95, 4x1 tiles

**Identity card - roughnessStandard0.4um_locat...**		
Name:	roughnessStandard0.4um_location3_LSM_50x-0.95_...	
Created on:	8/17/2018 2:14:19 PM	
**Axis:**	**X**	
Length:	944	µm
Size:	7563	points
Spacing:	0.125	µm
**Axis:**	**Y**	
Length:	258	µm
Size:	2069	points
Spacing:	0.125	µm
**Axis:**	**Z**	
Length:	10.6	µm
Size:	65532	digits
Spacing:	0.162	nm
NMP ratio:	0.987 % (154420 Pts)	

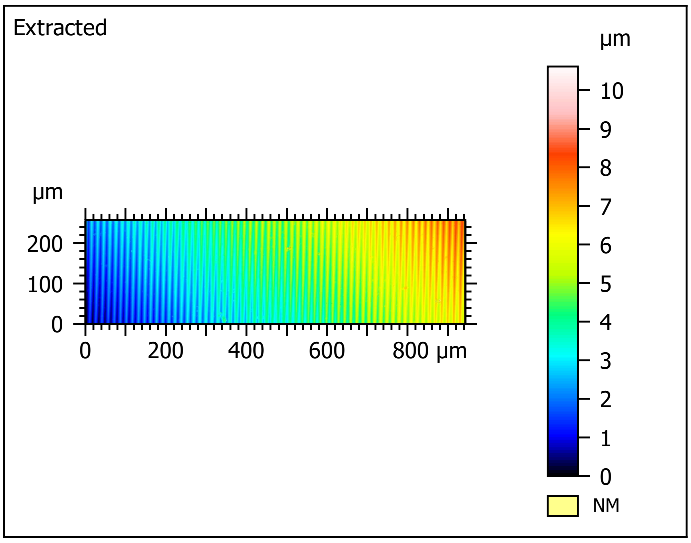

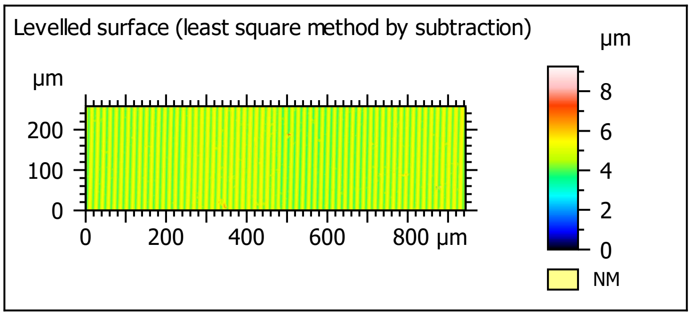

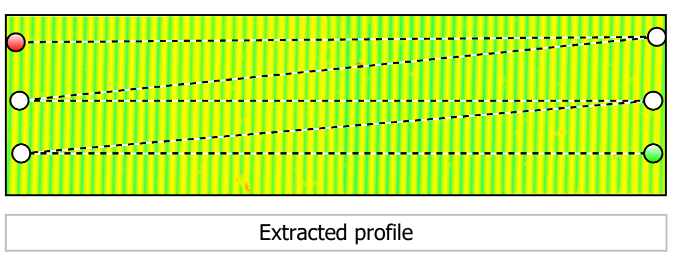

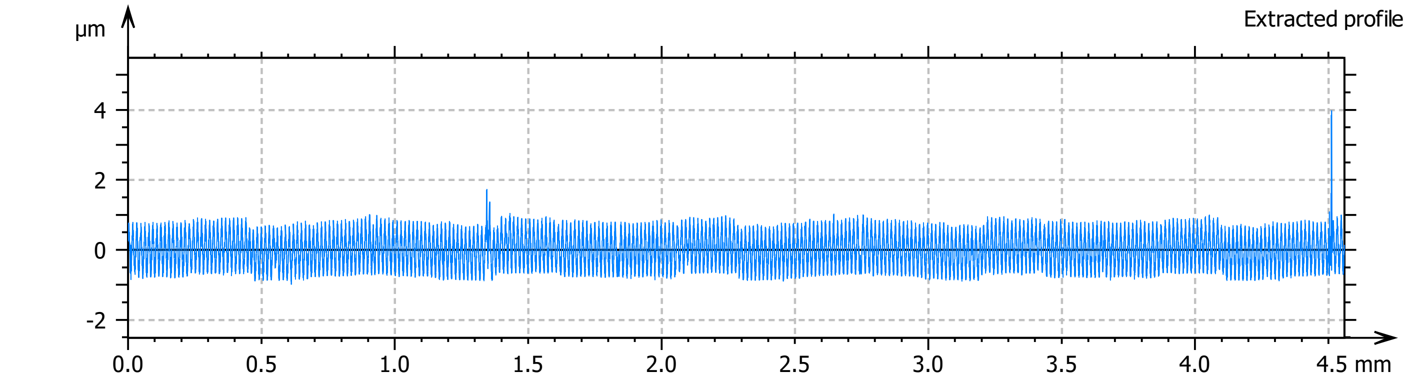

**Parameters**	Value	Unit
Length	4.56	mm

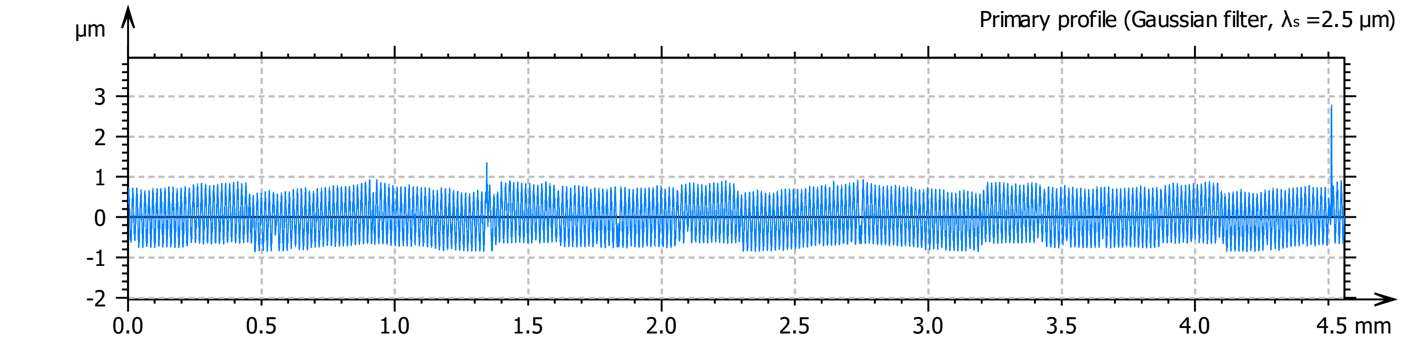

**Parameters**	Value	Unit
Length	4.56	mm

ISO 4287				
**Amplitude parameters - Roughness profile**				
Ra	0.418	µm	*Gaussian filter, 0.8 mm*	*Arithmetic mean deviation of the roughness profile.*

**Parameters**	Value	Unit	Tolerance	
Ra - Amplitude parameters - Roughness profile - ISO 4287	0.418	µm	0.400 µm ± 0.050 µm	✓

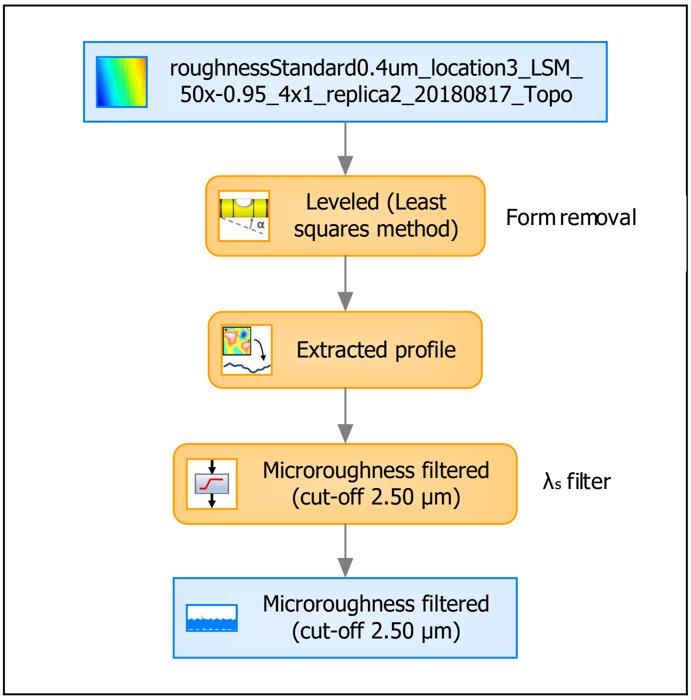

Roughness standard *Ra* = 0.4 um
50x objective, NA = 0.75 or 0.95, 4x1 tiles

Extracted

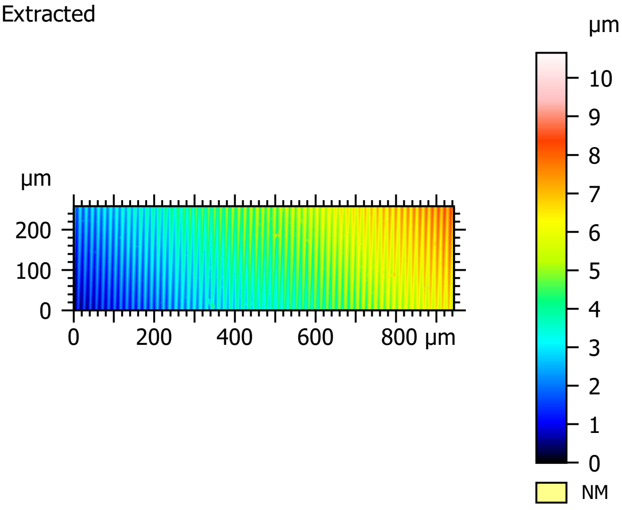

**Identity card - roughnessStandard0.4um_locat...**		
Name:	roughnessStandard0.4um_location3_LSM_50x-0.95_...	
Created on:	8/17/2018 2:33:55 PM	
**Axis:**	**X**	
Length:	944	µm
Size:	7563	points
Spacing:	0.125	µm
**Axis:**	**Y**	
Length:	258	µm
Size:	2069	points
Spacing:	0.125	µm
**Axis:**	**Z**	
Length:	10.7	µm
Size:	65532	digits
Spacing:	0.163	nm
NMP ratio:	0.987 % (154420 Pts)	

Levelled surface (least square method by subtraction)

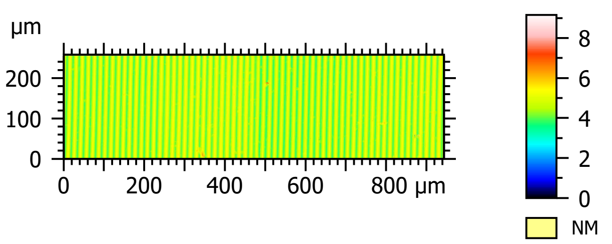

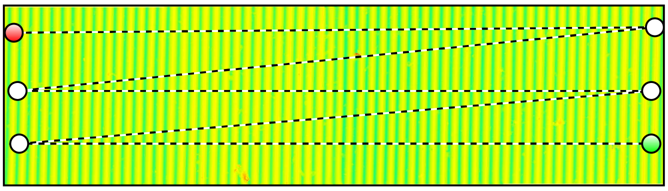

Extracted profile

Extracted profile

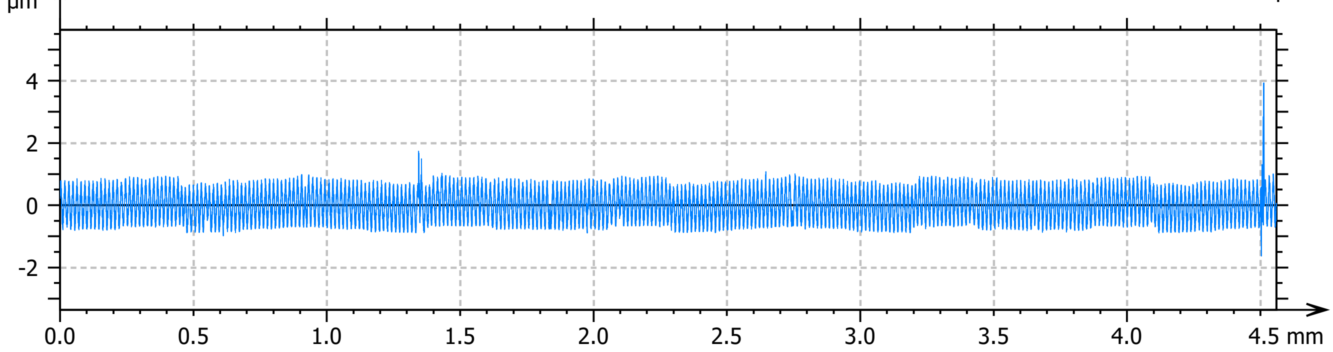

**Parameters**	Value	Unit
Length	4.56	mm

Primary profile (Gaussian filter, λ_s =2.5 µm)

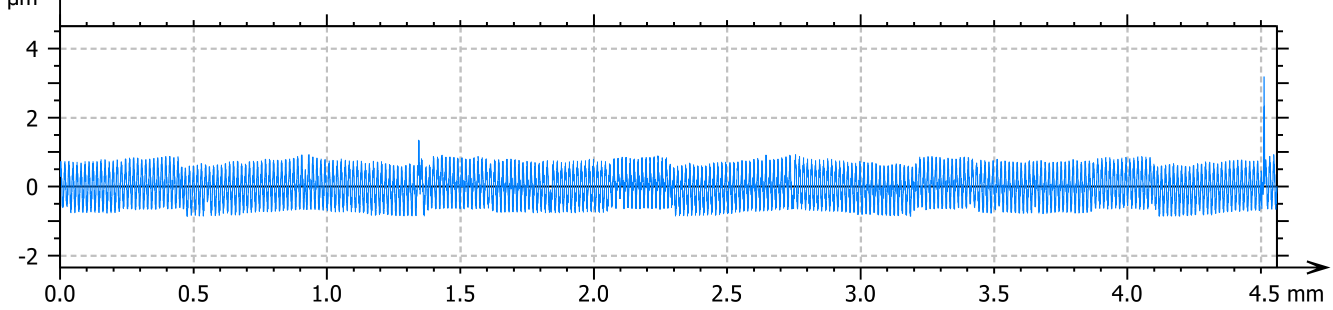

**Parameters**	Value	Unit
Length	4.56	mm

ISO 4287				
**Amplitude parameters - Roughness profile**				
Ra	0.418	µm	*Gaussian filter, 0.8 mm*	*Arithmetic mean deviation of the roughness profile.*

**Parameters**	Value	Unit	Tolerance	
Ra - Amplitude parameters - Roughness profile - ISO 4287	0.418	µm	0.400 µm ± 0.050 µm	✓

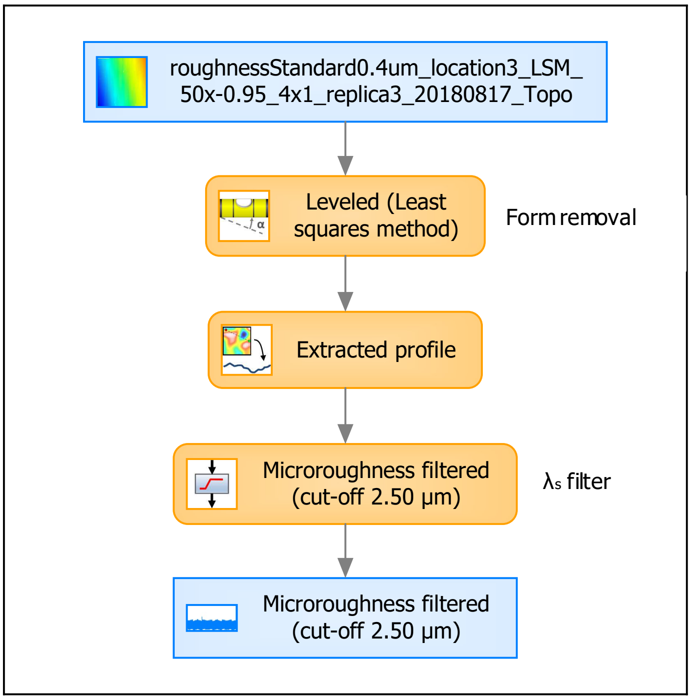

Supplement: Supplementary file 2 — Supplementary Material 2 [file 41598_2019_42713_MOESM2_ESM.pdf]
