# Supplementary material for: The effect of numerical aperture on quantitative use-wear studies and its implication on reproducibility
Source: Sci Rep. 2019 Apr 19;9:6313. doi: 10.1038/s41598-019-42713-w (PMC6474883; doi:10.1038/s41598-019-42713-w)
Supplement: Supplementary file 3 — Supplementary Material 3 [file 41598_2019_42713_MOESM3_ESM.pdf]

The effect of numerical aperture on quantitative use-wear studies and its implication on reproducibility

Ivan Calandra, Lisa Schunk, Konstantin Bob, Walter Gneisinger, Antonella Pedergnana, Eduardo Paixao, Andreas Hildebrandt, Joao Marreiros

Scientific Reports

Supplementary Material 3. Raw data, and RStudio project, R markdown scripts and HTML outputs of the statistical procedures.

All files are available on Zenodo (DOI: https://doi.org/10.5281/zenodo.2594755).

‘Acquisition paper.Rproj’: RStudio project of the analysis.

‘*.csv’: CSV files of raw data used for scripts #1 (one file per sample); scripts #2-3 used data in *.Rbin format created during script #1 (section 5.3).

They should be placed in a folder ‘Data’ within the analysis directory. Note that the ‘last status change’ (= ‘creation’ on Windows) and last access times from these files are different from the files used in the analysis (see section 3 of script #1) because it is a copy of the latter; the modification date/time shows that these files are identical to the ones used for the analysis.

Scripts #1 (Rmd scripts + HTML outputs): Read data from ConfoMap’s batch processing (in CSV format) into R, format the data and save the formatted data (one file per sample).

Script #2 (Rmd scripts + HTML outputs): Calculate descriptive statistics for each location on the sample and objective (one file per sample).

Script #3 (Rmd scripts + HTML outputs): Scatter plots for all ISO 25178-2 parameters and for ISO 4287 *Ra* for each objective (one file per sample).
