# Supplementary material for: The effect of numerical aperture on quantitative use-wear studies and its implication on reproducibility
Source: Sci Rep. 2019 Apr 19;9:6313. doi: 10.1038/s41598-019-42713-w (PMC6474883; doi:10.1038/s41598-019-42713-w)
Supplement: Supplementary file 4 — Supplementary Material 4 [file 41598_2019_42713_MOESM4_ESM.pdf]

The effect of numerical aperture on quantitative use-wear studies and its implication on reproducibility

Ivan Calandra, Lisa Schunk, Konstantin Bob, Walter Gneisinger, Antonella Pedergnana, Eduardo Paixao, Andreas Hildebrandt, Joao Marreiros

Scientific Reports

Supplementary Material 4. Python script and results of the Bayesian Multi-factor ANOVA.

All files are available on Zenodo (DOI: https://doi.org/10.5281/zenodo.2594746).

'analysisRestrictedModel.py': Python script of the analysis procedure.

'*_Auto.pdf': Energy plots of Hamiltonian Monte Carlo for the 26 ISO 25178-2 parameters and for ISO 4287 *Ra*.

'*_Contrasts.pdf': Contrast plots between 50×/0.75 and 50×/0.95 objectives for the 26 ISO 25178-2 parameters and for ISO 4287 *Ra*. See also Fig. 3 for *Sa*, *Sdr*, *Sku*, *Sq*, *Std*, *Str*, *Sxp* and *Vmc* and Fig. 5b for *Ra*.

'*_HistoMatrix.pdf': Histograms of posterior for the 26 ISO 25178-2 parameters and for ISO 4287 *Ra*.

'*_Trace.pdf': Trace plots for the 26 ISO 25178-2 parameters and for ISO 4287 *Ra*.
